# Iron‐Catalyzed C−H Activation with Propargyl Acetates: Mechanistic Insights into Iron(II) by Experiment, Kinetics, Mössbauer Spectroscopy, and Computation

**DOI:** 10.1002/anie.201904110

**Published:** 2019-07-30

**Authors:** Jiayu Mo, Thomas Müller, João C. A. Oliveira, Serhiy Demeshko, Franc Meyer, Lutz Ackermann

**Affiliations:** ^1^ Institut für Organische und Biomolekulare Chemie Georg-August-Universität Göttingen Tammannstraße 2 37077 Göttingen Germany; ^2^ Institut für Anorganische Chemie Georg-August-Universität Göttingen Tammannstraße 4 37077 Göttingen Germany

**Keywords:** C−H activation, density functional calculations, iron, Mössbauer spectroscopy, reaction mechanisms

## Abstract

An iron‐catalyzed C−H/N−H alkyne annulation was realized by using a customizable clickable triazole amide under exceedingly mild reaction conditions. A unifying mechanistic approach combining experiment, spectroscopy, kinetics, and computation provided strong support for facile C−H activation by a ligand‐to‐ligand hydrogen transfer (LLHT) mechanism. Combined Mössbauer spectroscopic analysis and DFT calculations were indicative of high‐spin iron(II) species as the key intermediates in the C−H activation manifold.

## Graphical abstract

**All for one**: A unified mechanistic approach reveals the importance of high‐spin iron(II) for facile C−H activation in expedient propargylic acetate annulations. Experimental, spectroscopic, and computational methods provide insights into the reaction mechanism and the catalytically relevant species.

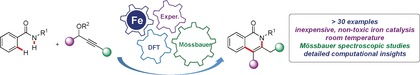

The use of iron complexes for molecular catalysis is highly attractive because iron is inexpensive, of low toxicity, and the most abundant 3d metal in the earth's crust.^1^ While considerable advances have been accomplished in iron‐catalyzed cross‐coupling chemistry,^2^ major challenges continue to be associated with these coupling reactions. Particularly, the need for substrate prefunctionalization and the formation of stoichiometric amounts of undesired by‐products translate into a strong demand for more step‐ and atom‐economic strategies. As a consequence, earth‐abundant^3^ iron‐catalyzed C−H activation^4^ has emerged as an increasingly powerful tool for the sustainable transformation of otherwise inert C−H bonds. While organometallic‐iron‐catalyzed C−H functionalizations have gained significant momentum during the last years,^5^ our mechanistic understanding of these processes continues to be rather poor, reflecting the highly complex nature of iron catalysis. In sharp contrast, and within our program on sustainable iron‐catalyzed C−H activation,^6^ we have now obtained key mechanistic insights into unprecedented iron(II)‐catalyzed C−H/N−H functionalizations with propargyl acetates for the first time, which we report herein. Thus, a unified mechanistic approach involving a combination of experiment, Mössbauer spectroscopy, and computation provides key insights into the importance of high‐spin iron(II) complexes as the crucial intermediates of the C−H functionalization manifold (Figure 1).

**Figure 1:**
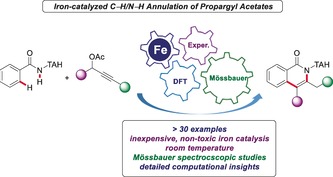
Iron(II)‐catalyzed C−H activation/annulations of propargyl acetates.

We initiated our studies by probing various reaction conditions for the envisioned iron‐catalyzed C−H/N−H activation of benzamide **1 a** with propargylic acetate **2 a** (Table 1 a, and Table S1 in the Supporting Information). Preliminary studies revealed that a variety of iron(II) and iron(III) precatalysts enabled efficient alkyne annulation, delivering the desired isoquinolone **3 aa** in high yields, even at ambient temperature (entries 1–5). The reaction did not occur in the absence of an iron catalyst (entry 6), or with alternative ligands, such as dcpe, dppp, dppf, or Xantphos (entries 7–10). The biomass‐derived solvent 2‐MeTHF proved to be a viable reaction medium (entry 11). A Job plot analysis regarding the metal‐to‐ligand ratio indicated a ratio of 1:1 ([Fe]/ligand) to give optimal results (Table S2).

**Table 1:** Optimization of the iron‐catalyzed C−H alkyne annulation.^[a]^ 
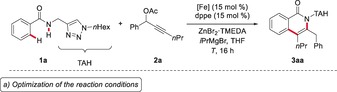

Entry	[Fe]	Ligand	*T* [°C]	Yield [%]
1	Fe(acac)_3_	dppe	65	51
2	Fe(acac)_3_	dppe	40	73
3	Fe(acac)_3_	dppe	23	64^[b]^
4	FeCl_3_	dppe	23	69
5	FeCl_2_	dppe	23	81
6	–	dppe	23	–
7	FeCl_2_	dcpe	23	–
8	FeCl_2_	dppp	23	<5
9	FeCl_2_	dppf	23	–
10	FeCl_2_	Xantphos	23	–
11	FeCl_2_	dppe	23	56^[c]^
12	FeCl_2_	dppe	23	58^[d]^
13	FeCl_2_	dppe	23	31^[e]^
14	Fe(acac)_2_	dppe	23	60^[f]^
**15**	**FeCl_2_**	dppe	**23**	**85^[f]^**
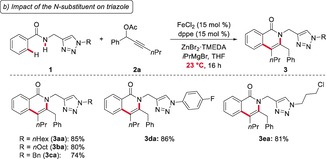				

[a] Reaction conditions: **1 a** (0.30 mmol), **2 a** (0.90 mmol), [Fe] (15 mol %), ligand (15 mol %), ZnBr_2_⋅TMEDA (0.60 mmol), *i*PrMgBr (0.90 mmol), THF (0.80 mL), *T*, 16 h; yields of isolated product are given. [b] 24 h. [c] 2‐MeTHF as the solvent. [d] 4 h. [e] FeCl_2_ (5.0 mol %). [f] Alkyne **2 a** (2.0 equiv). acac=acetylacetonate, dppe=1,2‐bis(diphenylphosphino)ethane, TMEDA=*N*,*N*,*N′*,*N′*‐tetramethylethylenediamine, dppp=1,3‐bis(diphenylphosphino)propane.

Next, we probed the impact of the N‐substitution pattern of the click‐triazole moiety on the iron‐catalyzed C−H/N−H activation (Table 1 b). Various methylene‐tethered triazoles **1** delivered the desired isoquinolones **3 aa**–**3 ea** in high yields, and the reaction tolerates alkyl, benzyl, and aryl groups, and even a reactive alkyl chloride (**1 d**).

Having identified optimized reaction conditions, we then explored the versatility of the iron‐catalyzed C−H activation with a variety of TAH‐benzamides **1** (Scheme 1 a). Here, numerous arenes with halogen substituents, such as chloro, bromo, or even iodo moieties, delivered the desired products **3** with high levels of chemoselectivity. Likewise, tertiary amines (**1 o**) and thioethers (**1 n**) were suitable substrates and efficiently delivered the corresponding isoquinolinones **3 na** and **3 oa**. Thiophene **1 w** also proved to be a viable substrate, while the olefin **1 y** gave the desired pyridone product **3 ya**.

**Scheme 1:**
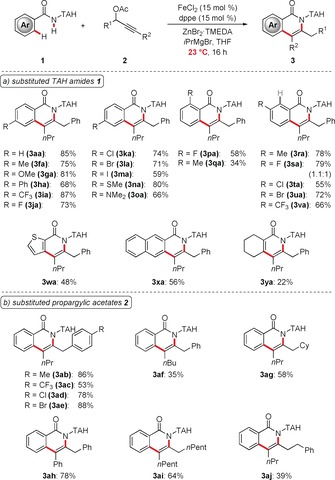
Versatility of the iron‐catalyzed C−H/N−H functionalizations with a) TAH amides **1** and b) propargylic acetates **2**.

The versatile iron catalyst further proved applicable to various propargyl acetates **2**, furnishing the corresponding isoquinolones **3 ab**–**3 aj** (Scheme 1 b). Different propargyl acetates **2** with electron‐donating or electron‐withdrawing groups were efficiently converted. Furthermore, the reaction fully tolerated synthetically meaningful aryl halides.

Given the efficacy of the novel iron‐catalyzed C−H activation, we became interested in delineating its mode of action. To this end, we tested the effect of the leaving group on the alkyne coupling partner **2**, and aliphatic carboxylates were found to be inherently more reactive (Scheme 2 a). Furthermore, intermolecular competition experiments highlighted the improved performance of electron‐deficient arenes **1** (Scheme 2 b), which can be rationalized by a ligand‐to‐ligand hydrogen transfer (LLHT) mechanism.^7^ In sharp contrast, the effect exerted by the electronic nature of alkynes **2** was less pronounced (Scheme 2 b). An additional asset is the possibility to remove the TAH group in a traceless fashion (Figure S3 in the Supporting Information).

**Scheme 2:**
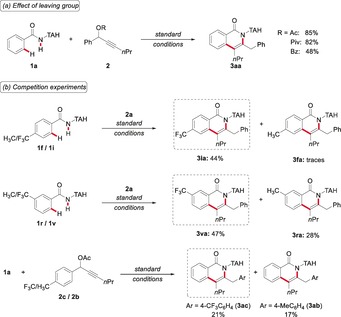
Competition experiments and effect of the leaving group in alkyne **2**.

A Hammett plot analysis of the initial rates of the iron‐catalyzed C−H activation with a range of substrates **2** showed a change in the slope (*k*_X_/*k*_H_), which can, among others, be explained by a change in the rate‐determining step (Scheme 3).^8^ C−D/N−H functionalization with the isotopically labeled substrates [D]_5_‐**1 e** and [D]_5_‐**1 a**, either by independent or competition experiments, showed no kinetic isotope effect (*k*_H_/*k*_D_=1.1), which is indicative of a facile C−H cleavage (Scheme 4 a, b). Further, the deuterium‐labeled substrate [D]_5_‐**1 a** underwent considerable H/D scrambling in the second *ortho* position of the product [D]_*n*_‐**3 aa**, again supporting a reversible C−H activation event (Scheme 4 c).

**Scheme 3:**
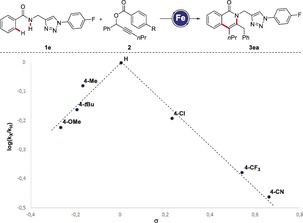
Hammett plot analysis with alkynes **2**.

**Scheme 4:**
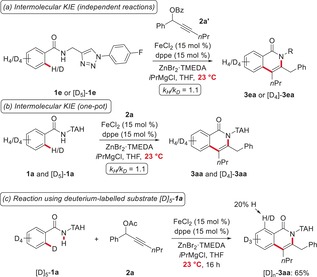
Kinetic isotope effect studies.

As to the catalyst's mode of action, detailed Mössbauer spectroscopic studies were performed to reveal the oxidation state of the iron species. Our observations provided strong support for the presence of high‐spin iron(II) intermediates (Table 2).^9^

**Table 2:** Mössbauer parameters of reaction mixtures. Frozen solutions measured at 80 K.

Entry	Reaction	Valence of iron/spin state	*δ* [mm s^−1^]	Δ*E*_Q_ [mm s^−1^]	Rel. int. [%]
1	^57^FeCl_2_+THF	+2^HS^	1.26	3.05	100
					
2	entry 1+MeMgBr	+1.49c	0.29	0.88	100
					
3	entry 2+ZnBr_2_⋅TMEDA	+2^HS^ +2^HS^	1.01 1.36	2.69 2.56	69 31
					
4	entry 3+dppe	+2^HS^ +2^HS^ +2^HS^	0.92 0.98 1.24	1.42 2.57 2.68	23 40 37
					
5	entry 4+**1 a**	n.a.^[a]^ +2^HS^ +2^HS^	0.26 1.14 1.00	1.01 2.45 3.17	43 36 21
					
6	entry 5+**2 a**	+2^HS^ +2^HS^	1.00 0.95	2.94 2.29	48 52

[a] n.a.=not assigned as the parameters are not specific.

The reaction mechanism was subsequently probed through detailed computational studies, by means of DFT at the PW6B95‐D3BJ/def2‐TZVP+SMD(THF)//TPSS‐D3BJ/def2‐SVP level of theory.^10^ Because of the nature of iron(II) complexes, the reaction path was explored by taking into consideration the potential energy surfaces of three spin states, singlet (low‐spin), triplet (intermediate‐spin), and quintet (high‐spin).

The overall C−H/N−H functionalization can be dissected into four elementary steps, namely a) C−H activation, b) alkyne migratory insertion, c) β‐O‐elimination, and d) allene migratory insertion (Figure 2). After proto‐demetalation, the latter delivers the desired isoquinolone product **3 aa**. Thus, computational analysis of the C−H activation was in line with a LLHT mechanism via **TS(0**‐**1)**, with an activation energy of 22.4 kcal mol^−1^. In contrast, a potential β‐hydride elimination pathway was shown to be significantly higher in energy (27.0 kcal mol^−1^; Figure S13 in the Supporting Information).^10^ The alkyne migratory insertion was proven to be facile via **TS(2**‐**3)**, with an activation energy of 23.6 kcal mol^−1^, followed by the exergonic formation of an allene‐coordinated intermediate, **I‐4**, with an activation energy of 8.1 kcal mol^−1^. This will undergo intramolecular migratory insertion into the N−Fe bond giving the isoquinolone‐derived complex **I‐5**. Here, evidence for a spin‐crossover, multi‐state scenario was not obtained.^11^ However, in the alkyne migratory insertion step, the quintet‐ and singlet‐state potential energy surfaces appear to be close in energy within less than 1 kcal mol^−1^.

**Figure 2:**
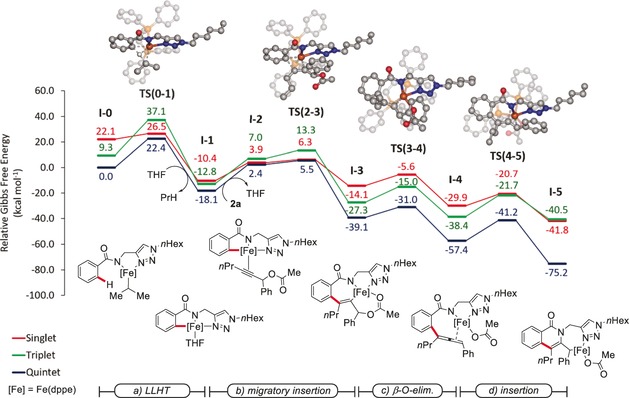
Computed Gibbs free energies (Δ*G*_298.15_) in kcal mol^−1^ for the iron‐catalyzed C−H/N−H annulation of propargyl acetate. All values include dispersion corrections. In the computed transition‐state structures, non‐relevant hydrogen atoms were omitted for clarity.

Based on our detailed experimental and computational mechanistic studies, we propose the iron(II)‐catalyzed C−H/N−H annulation sequence to be initiated by facile C−H activation through LLHT to generate the cyclometalated iron species **A** (Scheme 5). After coordination of substrate **2 a**, intermediate **B** undergoes fast migratory insertion to deliver complex **C**, which then forms the energetically favorable allene intermediate complex **D** in an exergonic pathway by cleavage of the C−O bond of the acetate leaving group. Thereafter, insertion of the allene moiety into the N−Fe bond forms the annulated iron complex **E**. Finally, proto‐demetalation releases the desired isoquinolone product **3 aa** and regenerates the active iron catalyst.

**Scheme 5:**
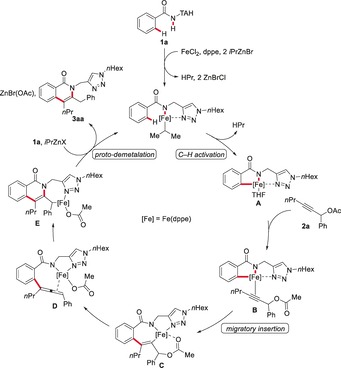
Proposed catalytic cycle.

In summary, we have reported on the realization and detailed mechanistic rationalization of an unprecedented C−H activation/annulation strategy with propargyl acetates enabled by a catalyst based on earth‐abundant iron. The C−H functionalization proceeded efficiently at ambient temperature and in the absence of external oxidants. The versatile iron catalyst provided expedient access to differently substituted isoquinolones. The first unifying mechanistic approach featuring a combination of experimental studies, kinetics, Mössbauer spectroscopy, and computation provided detailed mechanistic insight into the catalyst's mode of action, highlighting high‐spin iron(II) intermediates towards fast C−H activation.

## Conflict of interest

The authors declare no conflict of interest.

## Supporting information

As a service to our authors and readers, this journal provides supporting information supplied by the authors. Such materials are peer reviewed and may be re‐organized for online delivery, but are not copy‐edited or typeset. Technical support issues arising from supporting information (other than missing files) should be addressed to the authors.

Supplementary
