# Supplementary material for: Iron‐Catalyzed C−H Activation with Propargyl Acetates: Mechanistic Insights into Iron(II) by Experiment, Kinetics, Mössbauer Spectroscopy, and Computation
Source: Angew Chem Int Ed Engl. 2019 Jul 30;58(37):12874–8. doi: 10.1002/anie.201904110 (PMC7187192; doi:10.1002/anie.201904110)
Supplement: Supplementary file 1 — Supplementary [file ANIE-58-12874-s001.pdf]

Angewandte Chemie
Eine Zeitschrift der Gesellschaft Deutscher Chemiker

Supporting Information

Iron-Catalyzed C–H Activation with Propargyl Acetates: Mechanistic Insights into Iron(II) by Experiment, Kinetics, Mössbauer Spectroscopy, and Computation

*Jiayu Mo[+], Thomas Müller[+], João C. A. Oliveira[+], Serhiy Demeshko, Franc Meyer, and Lutz Ackermann**

anie_201904110_sm_miscellaneous_information.pdf

Table of Contents

General Remarks..S3

Optimization Studies for the Iron-Catalyzed C–H/N–H Annulation......................................S4

General Procedure for the Iron-Catalyzed C–H/N–H Annulation..S6

Characterization Data of Products **3** S7

Competition Experiments S29

Hammett-Plot Analysis for *para*-Substituted Propargyl Benzoates S31

Intermolecular Kinetic Isotope Effect (KIE) Measurement by Independent Reactions S33

Intermolecular KIE Measurement by One-Pot Reaction S34

Reaction Using Deuterium-Labelled Substrate $[D]_5$-**1a** S35

Deprotonation of Substrate **1a** by PhZnCl...S36

Reaction Using Propargyl Acetate **2k**...S37

Removal of TAH...S38

Mössbauer Spectroscopy – Analytical Data S39

Computational Studies S46

References...S91

NMR spectra S92

General Remarks

All reactions were carried out in Schlenk tubes under a N_2 atmosphere using pre-dried glassware. THF was dried using a solvent purification system (SPS) from MBraun SPS-800. All starting materials were synthesized according to previously described methods.[1-2] Alkynes **2** were synthesized according to known procedures.[3] PhZnCl was synthesized according to a known procedure.[4] $^{57}FeCl_2$ was synthesized from ^{57}Fe-enriched metal powder (95%, Isoflex) according to the literature.[5] *i*PrMgBr (3.0 M in 2-MeTHF) and MeMgBr (3.0 M in Et_2O) were purchased from Sigma-Aldrich. Other chemicals were obtained from commercial sources and were used without further purification. Yields refer to isolated compounds, estimated to be >95% pure as determined by ^{1}H-NMR. TLC was performed on Merck TLC Silica Gel 60 F_{254} with detection under UV light at 254 nm. Chromatographic separations were carried out on Merck Geduran SI-60 (0.040–0.063 mm, 230–400 mesh ASTM). All IR spectra were recorded on a Bruker FT-IR alpha-P device. EI-MS was recorded on Jeol *AccuTOF* at 70 eV; ESI-MS was recorded on Bruker Daltonik *micrOTOF* and *maXi*. Melting points (M.p.) were measured on Stuart™ melting point apparatus SMP3, values are uncorrected. Nuclear magnetic resonance (NMR) spectroscopy was performed at 300, 400, 500 or 600 MHz (^{1}H-NMR), 75, 101 or 126 MHz (^{13}C-NMR, APT) and 282, 376 or 470 MHz (^{19}F-NMR) on Bruker *Avance III HD 300, Avance III 300, Avance III 400, Avance III HD 500*, Varian *Unity-300, Inova 500* and *Inova 600* instruments. Chemical shifts (δ) are provided in *ppm* and are referenced to the residual solvent signal.

Stainless steel electrodes (Type 304, 10 mm × 15 mm × 0.25 mm; obtained from abcr, Germany) and RVC electrodes (5 mm × 10 mm × 6 mm, SIGRACELL® GFA 6 EA, obtained from SGL Carbon, Wiesbaden, Germany) were connected using stainless steel adapters. Electrocatalysis was conducted using an AXIOMET AX-3003P potentiostat in constant current mode.

Mössbauer spectra were recorded with a ^{57}Co source in a Rh matrix using an alternating constant acceleration *Wissel* Mössbauer spectrometer operated in the transmission mode and equipped with a *Janis* closed-cycle helium cryostat. Isomer shifts are given relative to iron metal at ambient temperature. Simulation of the experimental data was performed with the *Mfit* program[6] using *Lorentzian* line doublets.

Optimization Studies for the Iron-Catalyzed C–H/N–H Annulation

Table S-1. Optimization Studies for the Iron-Catalyzed C–H/N–H Annulation.[a]

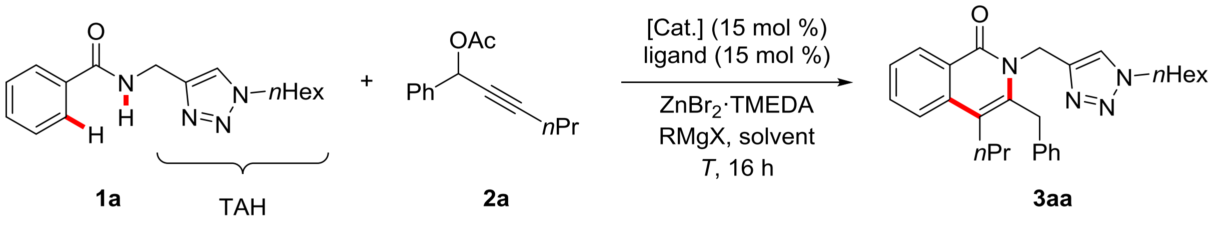

Entry	[Cat.]	ligand	RMgX	solvent	T [°C]	Yield [%]
1	$Fe(acac)_3$	dppe	*i*PrMgBr	THF	65	51
2	$Fe(acac)_3$	dppe	*i*PrMgBr	THF	40	73
3	$FeCl_3$	dppe	*i*PrMgBr	THF	23	69
4	$FeCl_2$	dppe	*i*PrMgBr	THF	23	81
5	$Fe(dbm)_3$	dppe	*i*PrMgBr	THF	23	58
6	---	dppe	*i*PrMgBr	THF	23	---
7	$Fe(acac)_3$	dppe	*i*PrMgBr	2-Me-THF	23	60
8	$FeCl_2$	dppe	*i*PrMgBr	2-Me-THF	23	56
9	$FeCl_2$	dppe	*i*PrMgBr	THF	40	80
10	$FeCl_2$	dppe	*i*PrMgBr	THF	60	75
11	$FeCl_2$	xantphos	*i*PrMgBr	THF	23	---
12	$FeCl_2$	dppf	*i*PrMgBr	THF	23	---
13	$FeCl_2$	dppp	*i*PrMgBr	THF	23	traces
14	$FeCl_2$	dcpe	*i*PrMgBr	THF	23	---
15	$FeCl_2$	*t*BuPhP(O)H	*i*PrMgBr	THF	23	---
16	$FeCl_2$	1,10-phen	*i*PrMgBr	THF	23	---
17	$FeCl_2$	dppe	*i*PrMgBr	THF	23	35[b]
18	$FeCl_2$	dppe	*i*PrMgBr	THF	23	58[c]
19	$FeCl_2$	dppe	*i*PrMgBr	THF	23	42[d]
20	$FeCl_2$	dppe	*i*PrMgBr	THF	23	33[e]
21	$FeCl_2$	dppe	*i*PrMgBr	THF	23	31[f]
22	$FeCl_2$	dppe	*i*PrMgBr	THF	23	85[g]
23	$Fe(acac)_2$	dppe	*i*PrMgBr	THF	23	60[g]
24	$FeCl_2$	dppe	*i*PrMgBr	THF	23	63[h]
25	$FeCl_2$	dppe	*i*PrMgBr	THF	23	25[i]
26	$FeCl_2$	dppe	*i*PrMgBr	THF	23	21[j]
27	$FeCl_2$	dppe	CyMgCl	THF	23	86[k]

[a] Reaction conditions: **1a** (0.30 mmol), **2a** (0.90 mmol), [Cat.] (15 mol %), ligand (15 mol %), $ZnBr_2$·TMEDA (0.60 mmol), *i*PrMgBr (3.0 M in 2-Me-THF, 0.90 mmol), solvent (0.80 mL), *T*, 16 h; yields of isolated product, [b] 1 h, [c] 4 h, [d] $FeCl_2$ (10 mol %), dppe (10 mol %), [e] $FeCl_2$ (7.5 mol %), dppe (7.5 mol %), [f] $FeCl_2$ (5.0 mol %), dppe (5.0 mol %), [g] 2.0 equiv **2a**, [h] 1.1 equiv **2a**, [i] *i*PrMgBr (3 M in THF, 0.60 mmol, 2.0 equiv), [j] $ZnBr_2$·TMEDA (0.30 mmol, 1.0 equiv), *i*PrMgBr (3 M in THF, 0.45 mmol, 1.5 equiv), [k] CyMgCl (0.9 M in THF, 0.90 mmol).

To a stirred solution of **1a** (0.30 mmol, 1.0 equiv), $ZnBr_2$·TMEDA (205 mg, 0.60 mmol, 2.0 equiv) and ligand (0.045 mmol, 15 mol %) in THF (0.40 mL), RMgX (0.90 mmol, 3.0 equiv) was added in one portion and the reaction mixture was stirred for 5 min at ambient temperature. Then, [Fe] (0.045 mmol, 15 mol %) was added in a single portion. After stirring the solution for additional 5 min, alkyne **2a** (130 mg, 0.90 mmol, 3.0 equiv) was added as a solution in THF (0.40 mL). Then, the mixture was placed in a pre-heated oil bath at the temperatures indicated above. After stirring for 16 h, sat. aqueous NH_4Cl (3.0 mL) was added and the reaction mixture was extracted with CH_2Cl_2 (3 × 15 mL). The combined organic extracts were dried over Na_2SO_4, filtered and concentrated. The crude product was purified by column chromatography (*n*hexane/EtOAc).

Table S-2: Job-plot analysis regarding the optimal metal-to-ligand ratio.

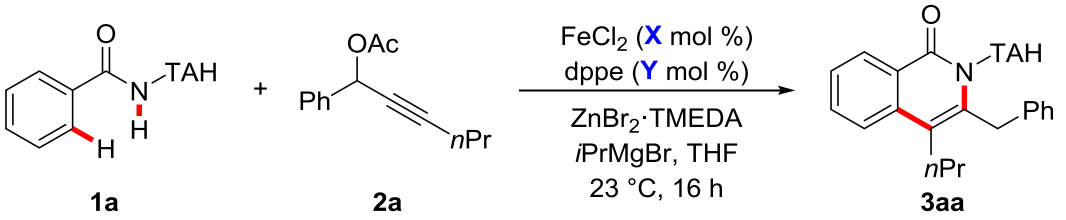

Entry	X	Y	[Fe]:L ratio	Yield / %
**1**	15	7.5	2:1 (1:0.5)	74
**2**	15	15	1:1	85
**3**	15	30	1:2	70
**4**	15	60	1:4	72

[a] Reaction conditions: **1a** (0.30 mmol), **2a** (0.60 mmol), $FeCl_2$ (**X** mol %), dppe (**Y** mol %), $ZnBr_2$·TMEDA (2.0 equiv), *i*PrMgBr (3.0 M in 2-Me-THF, 0.90 mmol), THF (0.80 mL), 23 °C, 16 h.

To a stirred solution of **1a** (0.30 mmol, 1.0 equiv), $ZnBr_2$·TMEDA (205 mg, 0.60 mmol, 2.0 equiv) and dppe in THF (0.40 mL), *i*PrMgBr (3.0 M in THF, 0.90 mmol, 3.0 equiv) was added in one portion and the reaction mixture was stirred for 5 min at ambient temperature. Then, $FeCl_2$ was added in a single portion. After stirring the solution for additional 5 min, alkyne **2a** (130 mg, 0.90 mmol, 2.0 equiv) was added as a solution in THF (0.40 mL). Then, the mixture was stirred at ambient temperature. After stirring for 16 h, sat. aqueous NH_4Cl (3.0 mL) was added and the reaction mixture was extracted with CH_2Cl_2 (3 × 15 mL). The combined organic extracts were dried over Na_2SO_4, filtered and concentrated. The crude product was purified by column chromatography (*n*hexane/EtOAc).

General Procedure for the Iron-Catalyzed C–H/N–H Annulation.

To a stirred solution of **1** (0.30 mmol, 1.0 equiv), $ZnBr_2$·TMEDA (205 mg, 0.60 mmol, 2.0 equiv) and dppe (17.9 mg, 0.045 mmol, 15 mol %) in THF (0.40 mL), *i*PrMgBr (3.0 M in THF, 300 μL, 0.90 mmol, 3.0 equiv) was added in one portion and the reaction mixture was stirred for 5 min at ambient temperature. Then, $FeCl_2$ (5.7 mg, 0.045 mmol,15 mol %) was added in a single portion. After stirring the solution for additional 5 min, alkyne **2** (0.60 mmol, 2.0 equiv) was added as a solution in THF (0.40 mL). Then, the mixture was stirred at ambient temperature. After stirring for 16 h, sat. aqueous NH_4Cl (3.0 mL) was added and the reaction mixture was extracted with CH_2Cl_2 (3 × 15 mL). The combined organic extracts were dried over Na_2SO_4, filtered and concentrated. The crude product was purified by column chromatography (*n*hexane/EtOAc).

Characterization Data of Products 3

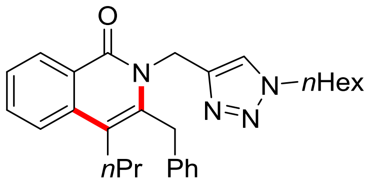

3-Benzyl-2-[(1-*n*-hexyl-1*H*-1,2,3-triazol-4-yl)methyl]-4-*n*-propylisoquinolin-1(2*H*)-one (3aa): The general procedure was followed using**1a** (85.9 mg, 0.30 mmol) and alkyne **2a** (130 mg, 0.60 mmol). Purification by column chromatography (*n*hexane/EtOAc = 3/2) yielded **3aa** (112 mg, 85%) as a white solid. **M.p.** = 97–98 °C. **^{1}H NMR** (600 MHz, $CDCl_3$): δ = 8.49 (d, J = 8.0 Hz, 1H), 7.74–7.68 (m, 3H), 7.48 (t, J = 7.3 Hz, 1H), 7.33 (t, J = 7.7 Hz, 2H), 7.28–7.24 (m, 3H), 5.19 (br s, 2H), 4.61 (s, 2H), 4.26 (t, J = 7.3 Hz, 2H), 2.77–2.71 (m, 2H), 1.89–1.82 (m, 2H), 1.61–1.53 (m, 2H), 1.32–1.23 (m, 6H), 0.99 (t, J = 7.3 Hz, 3H), 0.84 (t, J = 6.7 Hz, 3H). **^{13}C{H} NMR** (126 MHz, $CDCl_3$): δ = 162.7 (C_q), 144.2 (C_q), 137.4 (C_q), 137.1 (C_q), 136.7 (C_q), 132.4 (CH), 129.1 (CH), 128.3 (CH), 128.0 (CH), 126.9 (CH), 126.2 (CH), 125.3 (C_q), 124.0 (CH), 123.1 (CH), 116.4 (C_q), 50.5 (CH_2), 40.0 (CH_2), 35.0 (CH_2), 31.3 (CH_2), 30.4 (CH_2), 30.3 (CH_2), 26.3 (CH_2), 23.6 (CH_2), 22.6 (CH_2), 14.6 (CH_3), 14.1 (CH_3). **IR** (ATR): 3122, 2952, 2931, 1650, 1610, 1313, 1063, 776, 732, 715 cm^{-1}. **MS** (EI) *m/z* (relative intensity): 442 (72) $[M]^+$, 329 (32), 276 (86), 248 (100), 242 (47), 112 (64). **HR-MS** (EI) *m/z* calcd for $C_{28}H_{34}N_4O$ $[M]^+$ 442.2733, found 442.2722.

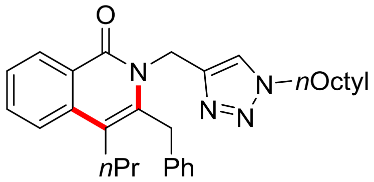

3-Benzyl-2-[(1-*n*-octyl-1*H*-1,2,3-triazol-4-yl)methyl]-4-*n*-propylisoquinolin-1(2*H*)-one (3ba): The general procedure was followed using **1b** (94.3 mg, 0.30 mmol) and alkyne **2a** (130 mg, 0.60 mmol). Purification by column chromatography (*n*hexane/EtOAc = 3/2) yielded **3ba** (113 mg, 80%) as an off-white solid. **M.p.** = 112–113 °C. **^{1}H NMR** (600 MHz, $CDCl_3$): δ = 8.49 (dd, J = 8.0, 0.9 Hz, 1H), 7.74–7.68 (m, 3H), 7.48 (ddd, J = 8.0, 6.7, 1.4 Hz, 1H), 7.33 (dd, J = 7.6, 7.6 Hz, 2H), 7.27–7.24 (m, 3H), 5.19 (br s, 2H), 4.61 (s, 2H), 4.26 (t, J = 7.3 Hz, 2H), 2.76–2.71 (m, 2H), 1.90–1.81 (m, 2H), 1.62–1.53 (m, 2H), 1.32–1.18 (m, 10H), 0.99 (t, J = 7.3 Hz, 3H), 0.85 (t, J = 7.1 Hz, 3H). **^{13}C{H} NMR** (126 MHz, $CDCl_3$): δ = 162.7 (C_q), 144.2 (C_q), 137.4 (C_q), 137.1 (C_q), 136.7 (C_q), 132.4 (CH), 129.1 (CH), 128.3 (CH), 128.0

(CH), 126.9 (CH), 126.2 (CH), 125.3 (C_q), 124.1 (CH), 123.1 (CH), 116.4 (C_q), 50.6 (CH_2), 40.0 (CH_2), 35.0 (CH_2), 31.9 (CH_2), 30.4 (CH_2), 30.3 (CH_2), 29.2 (CH_2), 29.1 (CH_2), 26.7 (CH_2), 23.6 (CH_2), 22.8 (CH_2), 14.6 (CH_3), 14.3 (CH_3). **IR** (ATR): 3153, 2924, 1640, 1592, 1333, 1044, 773, 751, 714, 698 cm^{-1}. **MS** (EI) *m/z* (relative intensity): 470 (90) $[M]^+$, 329 (36), 276 (100), 270 (44), 248 (99), 140 (74). **HR-MS** (ESI) *m/z* calcd for $C_{30}H_{39}N_4O$ $[M+H]^+$ 471.3118, found 471.3114.

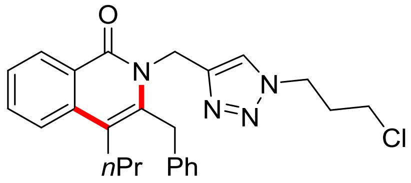

3-Benzyl-2-{[1-(3-chloropropyl)-1*H*-1,2,3-triazol-4-yl]methyl}-4-*n*-propylisoquinolin-1(2*H*)-one (3ca): The general procedure was followed using **1c** (83.6 mg, 0.30 mmol) and alkyne **2a** (130 mg, 0.60 mmol). Purification by column chromatography (*n*hexane/EtOAc = 3/2) yielded **3ca** (106 mg, 81%) as a yellow oil. **^{1}H NMR** (600 MHz, $CDCl_3$): δ = 8.48 (d, J = 7.7 Hz, 1H), 7.78 (s, 1H), 7.74–7.68 (m, 2H), 7.50–7.47 (m, 1H), 7.36-7.32 (m, 2H), 7.27–7.25 (m, 3H), 5.19 (br s, 2H), 4.60 (s, 2H), 4.46 (t, J = 6.7 Hz, 2H), 3.51 (t, J = 6.1 Hz, 2H), 2.76–2.72 (m, 2H), 2.37–2.33 (m, 2H), 1.61–1.54 (m, 2H), 1.00 (t, J = 7.3 Hz, 3H). **^{13}C{H} NMR** (126 MHz, $CDCl_3$): δ = 162.7 (C_q), 144.3 (C_q), 137.3 (C_q), 137.0 (C_q), 136.7 (C_q), 132.5 (CH), 129.1 (CH), 128.3 (CH), 127.9 (CH), 126.9 (CH), 126.2 (CH), 125.3 (C_q), 124.7 (CH), 123.2 (CH), 116.5 (C_q), 47.3 (CH_2), 41.4 (CH_2), 39.9 (CH_2), 35.0 (CH_2), 32.7 (CH_2), 30.5 (CH_2), 23.6 (CH_2), 14.62 (CH_3). **IR** (ATR): 2957, 2870, 1639, 1590, 1337, 1314, 1045, 770, 710, 698 cm^{-1}. **MS** (EI) *m/z* (relative intensity): 434 (50) $[^{35}Cl, M]^+$, 329 (39), 276 (68), 248 (100), 234 (66), 91 (43). **HR-MS** (ESI) *m/z* calcd for $C_{25}H_{28}{}^{35}ClN_4O$ $[M+H]^+$ 435.1946, found 435.1944.

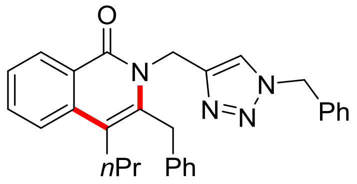

3-Benzyl-2-[(1-benzyl-1*H*-1,2,3-triazol-4-yl)methyl]-4-*n*-propylisoquinolin-1(2*H*)-one (3da): The general procedure was followed using **1d** (57.7 mg, 0.20 mmol) and alkyne **2a** (86.5 mg, 0.40 mmol). Purification by column chromatography (*n*hexane/EtOAc = 3/2) yielded **3da** (65.0 mg, 74%) as a white solid. **M.p.** = 156–157 °C. **^{1}H NMR** (300 MHz,

$CDCl_3$): δ = 8.48 (d, J = 7.8 Hz, 1H), 7.77–7.67 (m, 3H), 7.53–7.46 (m, 1H), 7.40–7.32 (m, 5H), 7.31–7.24 (m, 5H), 5.47 (s, 2H), 5.19 (br s, 2H), 4.64 (s, 2H), 2.80–2.72 (m, 2H), 1.67–1.52 (m, 2H), 1.02 (t, J = 7.3 Hz, 3H). **^{13}C{H} NMR** (126 MHz, $CDCl_3$): δ = 162.7 (C_q), 144.7 (C_q), 137.3 (C_q), 137.1 (C_q), 136.7 (C_q), 134.5 (C_q), 132.4 (CH), 129.1 (CH), 128.7 (CH), 128.7 (CH), 128.3 (CH), 128.2 (CH), 127.9 (CH), 126.9 (CH), 126.1 (CH), 125.3 (C_q), 124.2 (CH), 123.1 (CH), 116.4 (C_q), 54.3 (CH_2), 39.9 (CH_2), 35.0 (CH_2), 30.4 (CH_2), 23.6 (CH_2), 14.6 (CH_3). **IR** (ATR): 3134, 2958, 2644, 2594, 1340, 1054, 804, 713, 693, 454 cm^{-1}. **MS** (EI) *m/z* (relative intensity): 448 (19) [M]$^+$, 329 (23), 276 (22), 248 (43), 91 (100), 43 (70). **HR-MS** (ESI) *m/z* calcd for $C_{29}H_{29}N_4O$ [M+H]$^+$ 449.2336, found 449.2332.

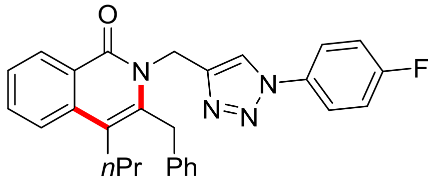

3-Benzyl-2-{[1-(4-fluorophenyl)-1*H*-1,2,3-triazol-4-yl]methyl}-4-*n*-propylisoquinolin-1(2*H*)-one(3ea): The general procedure was followed using **1e** (88.9 mg, 0.30 mmol) and alkyne **2a** (130 mg, 0.60 mmol). Purification by column chromatography (*n*hexane/EtOAc = 3/2) yielded **3ea** (117 mg, 86%) as a white solid. **M.p.** = 94–96 °C. **^{1}H NMR** (600 MHz, $CDCl_3$): δ = 8.49 (d, J = 8.1 Hz, 1H), 8.17 (s, 1H), 7.75–7.70 (m, 2H), 7.70–7.67 (m, 2H), 7.51–7.48 (m, 1H), 7.36 (dd, J = 7.6, 7.6 Hz, 2H), 7.30–7.26 (m, 3H), 7.19–7.15 (m, 2H), 5.27 (br s, 2H), 4.64 (s, 2H), 2.78–2.73 (m, 2H), 1.63–1.54 (m, 2H), 1.01 (t, J = 7.3 Hz, 3H). **^{13}C{H} NMR** (126 MHz, $CDCl_3$): δ = 162.8 (C_q), 162.4 (d, $^1J_{C-F}$ = 248.8 Hz, C_q), 145.1 (C_q), 137.3 (C_q), 136.9 (C_q), 136.7 (C_q), 133.3 (d, $^4J_{C-F}$ = 2.8 Hz, C_q), 132.5 (CH), 129.2 (CH), 128.3 (CH), 128.0 (CH), 127.0 (CH), 126.3 (CH), 125.2 (C_q), 123.2 (CH), 122.6 (CH), 122.5 (d, $^3J_{C-F}$ = 8.6 Hz, CH), 116.7 (d, $^2J_{C-F}$ = 23.2 Hz, CH), 116.7 (C_q), 39.9 (CH_2), 35.1 (CH_2), 30.5 (CH_2), 23.7 (CH_2), 14.6 (CH_3). **^{19}F NMR** (282 MHz, $CDCl_3$): δ = (-112.24)–(-112.34) (m). **IR** (ATR): 2957, 1640, 1590, 1514, 1337, 1232, 1041, 836, 769, 703 cm^{-1}. **MS** (EI) *m/z* (relative intensity): 452 (41) [M]$^+$, 424 (50), 276 (40), 248 (100), 148 (71), 43 (81). **HR-MS** (ESI) *m/z* calcd for $C_{28}H_{26}FN_4O$ [M+H]$^+$ 453.2085, found 453.2082.

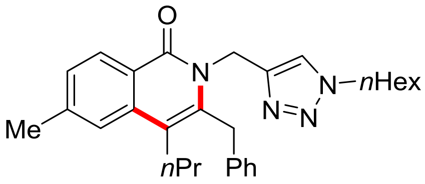

3-Benzyl-2-[(1-*n*-hexyl-1*H*-1,2,3-triazol-4-yl)methyl]-6-methyl-4-*n*-propylisoquinolin-1(2*H*)-one (3fa): The general procedure was followed using **1f** (90.1 mg, 0.30 mmol) and alkyne **2a** (130 mg, 0.60 mmol). Purification by column chromatography (*n*hexane/EtOAc = 3/2) yielded **3fa** (103 mg, 75%) as a white solid. **M.p.** = 93–94 °C. **^{1}H NMR** (600 MHz, $CDCl_3$): δ = 8.37 (d, J = 8.2 Hz, 1H), 7.72 (s, 1H), 7.48 (s, 1H), 7.34–7.30 (m, 3H), 7.27–7.24 (m, 3H), 5.17 (br s, 2H), 4.59 (br s, 2H), 4.25 (t, J = 7.4 Hz, 2H), 2.74–2.68 (m, 2H), 2.52 (s, 3H), 1.88–1.82 (m, 2H), 1.61–1.52 (m, 2H), 1.31–1.23 (m, 6H), 1.00 (t, J = 7.3 Hz, 3H), 0.84 (t, J = 7.0 Hz, 3H). **^{13}C{H} NMR** (126 MHz, $CDCl_3$): δ = 162.7 (C_q), 144.4 (C_q), 142.8 (C_q), 137.5 (C_q), 137.2 (C_q), 136.9 (C_q), 129.1 (CH), 128.3 (CH), 1280 (CH), 127.8 (CH), 126.9 (CH), 124.0 (CH), 123.1 (C_q), 122.9 (CH), 116.2 (C_q), 50.5 (CH_2), 39.9 (CH_2), 35.0 (CH_2), 31.3 (CH_2), 30.4 (CH_2), 30.3 (CH_2), 26.4 (CH_2), 23.6 (CH_2), 22.6 (CH_2), 22.5 (CH_3), 14.6 (CH_3), 14.1 (CH_3). **IR** (ATR): 3133, 2958, 2917, 1649, 1614, 1336, 1055, 830, 723, 467 cm^{-1}. **MS** (EI) *m/z* (relative intensity): 456 (100) $[M]^+$, 343 (45), 290 (98), 262 (89), 242 (37), 112 (51). **HR-MS** (EI) *m/z* calcd for $C_{29}H_{36}N_4O$ $[M]^+$ 456.2889, found 456.2883.

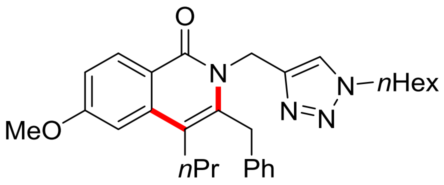

3-Benzyl-2-[(1-*n*-hexyl-1*H*-1,2,3-triazol-4-yl)methyl]-6-methoxy-4-*n*-propylisoquinolin-1(2*H*)-one (3ga): The general procedure was followed using **1g** (94.9 mg, 0.30 mmol) and alkyne **2a** (130 mg, 0.60 mmol). Purification by column chromatography (*n*hexane/EtOAc = 3/2) yielded **3ga** (115 mg, 81%) as a white solid. **M.p.** = 108–110 °C. **^{1}H NMR** (600 MHz, $CDCl_3$): δ = 8.42–8.40 (m, 1H), 7.72 (s, 1H), 7.35–7.31 (m, 2H), 7.27–7.23 (m, 3H), 7.09–7.05 (m, 2H), 5.16 (br s, 2H), 4.58 (br s, 2H), 4.25 (t, J = 7.3 Hz, 2H), 3.93 (s, 3H), 2.71–2.67 (m, 2H), 1.88–1.82 (m, 2H), 1.61–1.52 (m, 2H), 1.31–1.24 (m, 6H), 0.99 (t, J = 7.3 Hz, 3H), 0.84 (t, J = 7.0 Hz, 3H). **^{13}C{H} NMR** (126 MHz, $CDCl_3$): δ = 162.9 (C_q), 162.4 (C_q), 144.4 (C_q), 138.7 (C_q), 137.8 (C_q), 137.4 (C_q), 130.4 (CH), 129.1 (CH), 128.0 (CH), 126.9 (CH), 124.0 (CH), 119.3 (C_q), 115.9 (C_q), 114.7 (CH), 105.3 (CH), 55.6 (CH_3),

50.5 (CH_2), 39.8 (CH_2), 35.1 (CH_2), 31.3 (CH_2), 30.6 (CH_2), 30.3 (CH_2), 26.4 (CH_2), 23.4 (CH_2), 22.6 (CH_2), 14.7 (CH_3), 14.1 (CH_3). **IR** (ATR): 2950, 2929, 1646, 1612, 1597, 1235, 1031, 840, 791, 724 cm^{-1}. **MS** (ESI) *m/z* (relative intensity): 967 (30) $[2M+Na]^+$, 945 (13) $[2M+H]^+$, 495 (17) $[M+Na]^+$, 473 (100) $[M+H]^+$. **HR-MS** (ESI) *m/z* calcd for $C_{29}H_{37}N_4O_2$ $[M+H]^+$ 473.2911, found 473.2902.

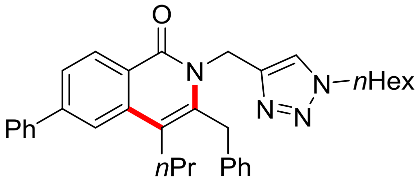

3-Benzyl-2-[(1-*n*-hexyl-1*H*-1,2,3-triazol-4-yl)methyl]-6-phenyl-4-*n*-propylisoquinolin-1(2*H*)-one (3ha): The general procedure was followed using **1h** (109 mg, 0.30 mmol) and alkyne **2a** (130 mg, 0.60 mmol). Purification by column chromatography (*n*hexane/EtOAc = 3/2) yielded **3ha** (106 mg, 68%) as a pale yellow solid. **M.p.** = 90–92 °C. **^{1}H NMR** (600 MHz, $CDCl_3$): δ = 8.55 (d, J = 8.3 Hz, 1H), 7.89 (d, J = 1.6 Hz, 1H), 7.75 (s, 1H), 7.72 (dd, J = 8.3, 1.6 Hz, 1H), 7.70–7.67 (m, 2H), 7.54–7.50 (m, 2H), 7.45–7.42 (m, 1H), 7.37–7.33 (m, 2H), 7.30–7.25 (m, 3H), 5.21 (br s, 2H), 4.64 (br s, 2H), 4.27 (t, J = 7.3 Hz, 2H), 2.83–2.79 (m, 2H), 1.90–1.83 (m, 2H), 1.68–1.58 (m, 2H), 1.33–1.24 (m, 6H), 1.01 (t, J = 7.3 Hz, 3H), 0.85 (t, J = 7.0 Hz, 3H). **^{13}C{H} NMR** (126 MHz, $CDCl_3$): δ = 162.6 (C_q), 145.3 (C_q), 144.2 (C_q), 140.9 (C_q), 137.6 (C_q), 137.3 (C_q), 137.1 (C_q), 129.1 (CH), 129.1 (CH), 128.9 (CH), 128.2 (CH), 128.0 (CH), 127.6 (CH), 126.9 (CH), 125.5 (CH), 124.2 (C_q), 124.0 (CH), 121.6 (CH), 116.5 (C_q), 50.5 (CH_2), 40.0 (CH_2), 35.1 (CH_2), 31.3 (CH_2), 30.4 (CH_2), 30.3 (CH_2), 26.4 (CH_2), 23.7 (CH_2), 22.6 (CH_2), 14.7 (CH_3), 14.1 (CH_3). **IR** (ATR): 2954, 2928, 2868, 1640, 1614, 1452, 1314, 1046, 761, 696 cm^{-1}. **MS** (ESI) *m/z* (relative intensity): 1059 (63) $[2M+Na]^+$, 841 (16), 519 (100) $[M+H]^+$, 323 (73). **HR-MS** (ESI) *m/z* calcd for $C_{34}H_{39}N_4O$ $[M+H]^+$ 519.3118, found 519.3104.

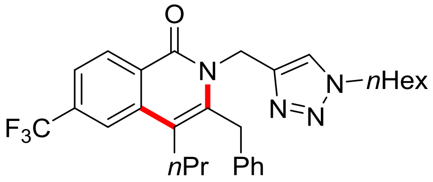

3-Benzyl-2-[(1-*n*-hexyl-1*H*-1,2,3-triazol-4-yl)methyl]-4-*n*-propyl-6-(trifluoromethyl)iso-quinolin-1(2H)-one (3ia): The general procedure was followed using **1i** (106 mg, 0.30 mmol) and alkyne **2a** (130 mg, 0.60 mmol). Purification by column chromatography

(*n*hexane/EtOAc = 3/2) yielded **3ia** (133 mg, 87%) as a white solid. **M.p.** = 133–134 °C. **^{1}H NMR** (600 MHz, $CDCl_3$): δ = 8.59 (d, J = 8.4 Hz, 1H), 7.96 (s, 1H), 7.73 (s, 1H), 7.67 (dd, J = 8.4, 1.3 Hz, 1H), 7.35 (dd, J = 7.6, 7.6 Hz, 2H), 7.29–7.24 (m, 3H), 5.19 (br s, 2H), 4.66 (br s, 2H), 4.27 (t, J = 7.3 Hz, 2H), 2.79–2.74 (m, 2H), 1.90–1.83 (m, 2H), 1.62–1.53 (m, 2H), 1.32–1.24 (m, 6H), 1.01 (t, J = 7.3 Hz, 3H), 0.84 (t, J = 7.0 Hz, 3H). **^{13}C{H} NMR** (126 MHz, $CDCl_3$): δ = 161.9 (C_q), 143.7 (C_q), 139.0 (C_q), 136.9 (C_q), 136.8 (C_q), 134.0 (q, $^2J_{C\text{–}F}$ = 32.1 Hz, C_q), 129.4 (CH), 129.2 (CH), 127.9 (CH), 127.3 (C_q), 127.1 (CH), 124.1 (CH), 124.0 (q, $^1J_{C\text{–}F}$ = 272.7 Hz, C_q), 122.1 (q, $^3J_{C\text{–}F}$ = 3.1 Hz, CH), 120.5 (q, $^3J_{C\text{–}F}$ = 4.0 Hz, CH), 116.2 (C_q), 50.6 (CH_2), 40.2 (CH_2), 35.1 (CH_2), 31.3 (CH_2), 30.3 (CH_2), 30.3 (CH_2), 26.3 (CH_2), 23.6 (CH_2), 22.6 (CH_2), 14.5 (CH_3), 14.1 (CH_3). **^{19}F NMR** (282 MHz, $CDCl_3$): δ = -62.93 (s). **IR** (ATR): 2955, 2873, 1651, 1360, 1315, 1125, 1074, 795, 724, 696 cm^{-1}. **MS** (ESI) *m/z* (relative intensity): 1043 (31) $[2M+Na]^+$, 511 (100) $[M+H]^+$. **HR-MS** (ESI) *m/z* calcd for $C_{29}H_{34}F_3N_4O$ $[M+H]^+$ 511.2679, found 511.2678.

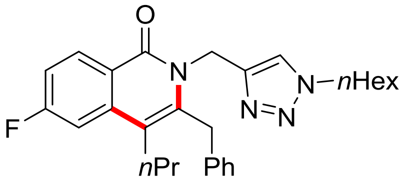

3-Benzyl-6-fluoro-2-[(1-*n*-hexyl-1*H*-1,2,3-triazol-4-yl)methyl]-4-*n*-propylisoquinolin-1(2*H*)-one (3ja): The general procedure was followed using **1j** (91.3 mg, 0.30 mmol) and alkyne **2a** (130 mg, 0.60 mmol). Purification by column chromatography (*n*hexane/EtOAc = 3/2) yielded **3ja** (100 mg, 73%) as a white solid. **M.p.** = 135–137 °C. **^{1}H NMR** (600 MHz, $CDCl_3$): δ = 8.48 (dd, J = 8.9, 6.1 Hz, 1H), 7.72 (s, 1H), 7.36–7.30 (m, 3H), 7.28–7.24 (m, 3H), 7.18 (ddd, J = 8.4, 8.4, 2.3 Hz, 1H), 5.16 (br s, 2H), 4.61 (br s, 2H), 4.26 (t, J = 7.3 Hz, 2H), 2.70–2.64 (m, 2H), 1.89–1.82 (m, 2H), 1.59–1.51 (m, 2H), 1.32–1.24 (m, 6H), 0.99 (t, J = 7.3 Hz, 3H), 0.84 (t, J = 6.8 Hz, 3H). **^{13}C{H} NMR** (126 MHz, $CDCl_3$): δ = 165.6 (d, $^1J_{C\text{–}F}$ = 250.9 Hz, C_q), 162.0 (C_q), 144.0 (C_q), 139.2 (d, $^3J_{C\text{–}F}$ = 9.7 Hz, C_q), 138.7 (C_q), 137.1 (C_q), 131.5 (d, $^3J_{C\text{–}F}$ = 10.1 Hz, CH), 129.2 (CH), 127.9 (CH), 127.0 (CH), 124.0 (CH), 121.9 (C_q), 115.9 (d, $^4J_{C\text{–}F}$ = 3.3 Hz, C_q), 114.8 (d, $^2J_{C\text{–}F}$ = 23.4 Hz, CH), 108.4 (d, $^2J_{C\text{–}F}$ = 22.8 Hz, CH), 50.5 (CH_2), 39.9 (CH_2), 35.1 (CH_2), 31.3 (CH_2), 30.6 (CH_2), 30.3 (CH_2), 26.4 (CH_2), 23.4 (CH_2), 22.6 (CH_2), 14.6 (CH_3), 14.1 (CH_3). **^{19}F NMR** (282 MHz, $CDCl_3$): δ = (-105.94)–(-106.06) (m). **IR** (ATR): 2952, 2929, 2868, 1650, 1601, 1342, 1165, 787, 724, 699 cm^{-1}. **MS** (ESI) *m/z* (relative intensity): 943 (100) $[2M+Na]^+$, 921 (8) $[2M+H]^+$, 483 (36)

[M+Na]$^+$, 461 (94) [M+H]$^+$.**HR-MS** (ESI) *m/z* calcd for $C_{28}H_{34}FN_4O$ [M+H]$^+$ 461.2711, found 461.2705.

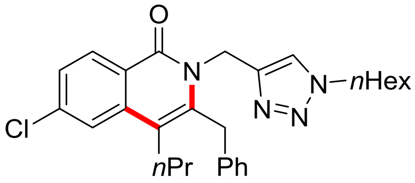

3-Benzyl-6-chloro-2-[(1-*n*-hexyl-1*H*-1,2,3-triazol-4-yl)methyl]-4-*n*-propylisoquinolin-1(2*H*)-one (3ka): The general procedure was followed using **1k** (96.2 mg, 0.30 mmol) and alkyne **2a** (130 mg, 0.60 mmol). Purification by column chromatography (*n*hexane/EtOAc = 3/2) yielded **3ka** (106 mg, 74%) as a pale yellow solid. **M.p.** = 146–147 °C. **^{1}H NMR** (600 MHz, $CDCl_3$): δ = 8.40 (d, *J* = 8.6 Hz, 1H), 7.71 (s, 1H), 7.66 (d, *J* = 1.9 Hz, 1H), 7.42 (dd, *J* = 8.6, 1.9 Hz, 1H), 7.34 (dd, *J* = 7.6, 7.6 Hz, 2H), 7.28–7.23 (m, 3H), 5.16 (br s, 2H), 4.61 (s, 2H), 4.26 (t, *J* = 7.3 Hz, 2H), 2.71–2.66 (m, 2H), 1.89–1.83 (m, 2H), 1.59–1.52 (m, 2H), 1.31–1.25 (m, 6H), 1.00 (t, *J* = 7.3 Hz, 3H), 0.84 (t, *J* = 7.0 Hz, 3H). **^{13}C{H} NMR** (126 MHz, $CDCl_3$): δ = 162.1 (C_q), 143.9 (C_q), 139.1 (C_q), 138.8 (C_q), 138.1 (C_q), 137.0 (C_q), 130.1 (CH), 129.2 (CH), 127.9 (CH), 127.0 (CH), 126.6 (CH), 124.0 (CH), 123.6 (C_q), 122.7 (CH), 115.6 (C_q), 50.5 (CH_2), 40.0 (CH_2), 35.1 (CH_2), 31.3 (CH_2), 30.4 (CH_2), 30.3 (CH_2), 26.4 (CH_2), 23.5 (CH_2), 22.6 (CH_2), 14.6 (CH_3), 14.1 (CH_3). **IR** (ATR): 3134, 2949, 2918, 1648, 1600, 1335, 1055, 831, 787, 723 cm^{-1}. **MS** (EI) *m/z* (relative intensity): 476 (83) [^{35}Cl, M]$^+$, 310 (89), 282 (89), 242 (80), 112 (100), 85 (88). **HR-MS** (EI) *m/z* calcd for $C_{28}H_{33}{}^{35}ClN_4O$[M]$^+$ 476.2343, found 476.2343.

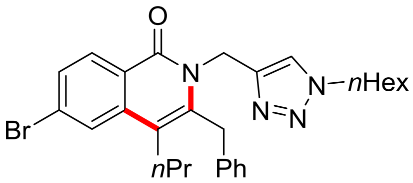

3-Benzyl-6-bromo-2-[(1-*n*-hexyl-1*H*-1,2,3-triazol-4-yl)methyl]-4-*n*-propylisoquinolin-1(2*H*)-one (3la): The general procedure was followed using **1l** (110 mg, 0.30 mmol) and alkyne **2a** (130 mg, 0.60 mmol). Purification by column chromatography (*n*hexane/EtOAc = 3/2) yielded **3la** (112 mg, 71%) as a white solid. **M.p.** = 144–145 °C. **^{1}H NMR** (600 MHz, $CDCl_3$): δ = 8.32 (d, *J* = 8.6 Hz, 1H), 7.83 (d, *J* = 1.6 Hz, 1H), 7.71 (s, 1H), 7.57 (dd, *J* = 8.6, 1.6 Hz, 1H), 7.34 (dd, *J* = 7.6, 7.6 Hz, 2H), 7.27–7.23 (m, 3H), 5.15 (br s, 2H), 4.61 (br s, 2H), 4.26 (t, *J* = 7.3 Hz, 2H), 2.71–2.65 (m, 2H), 1.89–1.81 (m, 2H),

1.59–1.51 (m, 2H), 1.32–1.23 (m, 6H), 1.00 (t, $J = 7.3$ Hz, 3H), 0.84 (t, $J = 6.8$ Hz, 3H). **^{13}C{H} NMR** (126 MHz, $CDCl_3$): $\delta = 162.2$ (C_q), 143.9 (C_q), 138.8 (C_q), 138.3 (C_q), 137.0 (C_q), 130.1 (CH), 129.4 (CH), 129.2 (CH), 127.9 (CH),127.9 (C_q), 127.0 (CH), 125.9 (CH), 124.0 (CH), 123.9 (C_q), 115.5 (C_q), 50.5 (CH_2), 40.0 (CH_2), 35.1 (CH_2), 31.3 (CH_2), 30.3 (CH_2), 30.3 (CH_2), 26.3 (CH_2), 23.5 (CH_2), 22.6 (CH_2), 14.6 (CH_3), 14.1 (CH_3). **IR** (ATR): 3134, 2949, 2917, 1647, 1598, 1377, 1055, 821, 786, 722 cm^{-1}. **MS** (EI) *m/z* (relative intensity): 522 (55) [^{81}Br, M]$^+$, 354 (54), 326 (58), 242 (80), 112 (100), 85 (75). **HR-MS** (EI) *m/z* calcd for $C_{28}H_{33}{}^{81}BrN_4O$ [M]$^+$ 522.1817, found 522.1803.

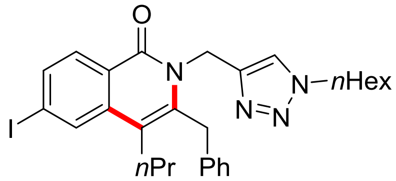

3-Benzyl-2-[(1-*n*-hexyl-1H-1,2,3-triazol-4-yl)methyl]-6-iodo-4-*n*-propylisoquinolin-1(2H)-one (3ma): The general procedure was followed using **1m** (124 mg, 0.30 mmol) and alkyne **2a** (130 mg, 0.60 mmol). Purification by column chromatography (*n*hexane/EtOAc = 3/2) yielded **3ma** (100 mg, 59%) as a white solid. **M.p.** = 131–133 °C. **^{1}H NMR** (300 MHz, $CDCl_3$): $\delta = 8.15$ (d, $J = 8.5$ Hz, 1H), 8.06 (s, 1H), 7.78 (d, $J = 8.5$ Hz, 1H), 7.71 (s, 1H), 7.38–7.30 (m, 2H), 7.29–7.21 (m, 3H), 5.15 (br s, 2H), 4.60 (s, 2H), 4.26 (t, $J = 7.3$ Hz, 2H), 2.72–2.63 (m, 2H), 1.92–1.79 (m, 2H), 1.63–1.48 (m, 2H), 1.33–1.21 (m, 6H), 0.99 (t, $J = 7.3$ Hz, 3H), 0.84 (t, $J = 6.6$ Hz, 3H). **^{13}C{H} NMR** (126 MHz, $CDCl_3$): $\delta = 162.4$ (C_q), 143.9 (C_q), 138.6 (C_q), 138.3 (C_q), 137.0 (C_q), 135.1 (CH), 132.3 (CH), 129.9 (CH), 129.2 (CH), 127.9 (CH), 127.0 (CH), 124.4 (C_q), 124.0 (CH), 115.2 (C_q), 100.8 (C_q), 50.5 (CH_2), 40.1 (CH_2), 35.1 (CH_2), 31.3 (CH_2), 30.3 (CH_2), 30.2 (CH_2), 26.4 (CH_2), 23.6 (CH_2), 22.6 (CH_2), 14.6 (CH_3), 14.1 (CH_3). **IR** (ATR): 2949, 2915, 1647, 1596, 1583, 1377, 1174, 1055, 786, 722 cm^{-1}. **MS** (ESI) *m/z* (relative intensity): 1159 (64) [2M+Na]$^+$, 591 (31) [M+Na]$^+$, 569 (100) [M+H]$^+$, 263 (41). **HR-MS** (ESI) *m/z* calcd for $C_{28}H_{34}IN_4O$[M+H]$^+$ 569.1772, found 569.1771.

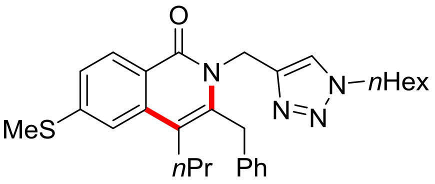

3-Benzyl-2-[(1-*n*-hexyl-1*H*-1,2,3-triazol-4-yl)methyl]-6-(methylthio)-4-*n*-propyliso-quinolin-1(2*H*)-one (3na): The general procedure was followed using **1n** (99.7 mg, 0.30 mmol) and alkyne **2a** (130 mg, 0.60 mmol). Purification by column chromatography (*n*hexane/EtOAc = 3/2) yielded **3na** (118 mg, 80%) as a white solid. **M.p.** = 111–112 °C. **^{1}H NMR** (600 MHz, $CDCl_3$): δ = 8.36 (d, J = 8.5 Hz, 1H), 7.73 (s, 1H), 7.46 (d, J = 1.6 Hz, 1H), 7.35–7.32 (m, 3H), 7.27–7.24 (m, 3H), 5.17 (br s, 2H), 4.60 (s, 2H), 4.26 (t, J = 7.3 Hz, 2H), 2.73–2.69 (m, 2H), 2.58 (s, 3H), 1.89–1.82 (m, 2H), 1.61–1.54 (m, 2H), 1.31–1.25 (m, 6H), 1.00 (t, J = 7.3 Hz, 3H), 0.85 (t, J = 6.9 Hz, 3H). **^{13}C{H} NMR** (126 MHz, $CDCl_3$): δ = 162.4 (C_q), 144.7 (C_q), 144.1 (C_q), 138.0 (C_q), 137.2 (C_q), 137.1 (C_q), 129.1 (CH), 128.6 (CH), 127.9 (CH), 126.9 (CH), 124.1 (CH), 123.9 (CH), 122.2 (C_q), 118.7 (CH), 115.7 (C_q), 50.6 (CH_2), 39.9 (CH_2), 35.0 (CH_2), 31.3 (CH_2), 30.4 (CH_2), 30.3 (CH_2), 26.3 (CH_2), 23.5 (CH_2), 22.6 (CH_2), 15.3 (CH_3), 14.6 (CH_3), 14.1 (CH_3). **IR** (ATR): 3125, 2949, 2921, 1645, 1601, 1587, 1178, 834, 787, 722 cm^{-1}. **MS** (EI) *m/z* (relative intensity): 488 (100) $[M]^+$, 375 (50), 322 (81), 294 (74), 242 (42), 112 (72). **HR-MS** (ESI) *m/z* calcd for $C_{29}H_{37}N_4OS$ $[M+H]^+$ 489.2683, found 489.2680.

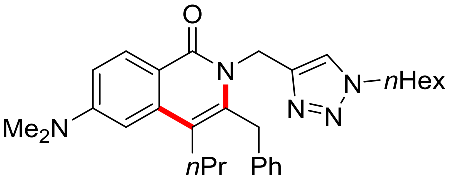

3-Benzyl-6-(dimethylamino)-2-[(1-*n*-hexyl-1*H*-1,2,3-triazol-4-yl)methyl]-4-*n*-propyliso-quinolin-1(2*H*)-one (3oa): The general procedure was followed using **1o** (95.8 mg, 0.30 mmol) and alkyne **2a** (130 mg, 0.60 mmol). Purification by column chromatography (*n*hexane/EtOAc = 3/2) yielded **3oa** (95.8 mg, 66%) as an off-white solid. **M.p.** = 118–120 °C. **^{1}H NMR** (600 MHz, $CDCl_3$): δ = 8.33 (d, J = 9.0 Hz, 1H), 7.71 (s, 1H), 7.34–7.30 (m, 2H), 7.27–7.22 (m, 3H), 6.95 (dd, J = 9.0, 1.8 Hz, 1H), 6.78 (s, 1H), 5.15 (br s, 2H), 4.54 (s, 2H), 4.24 (t, J = 7.4 Hz, 2H), 3.11 (s, 6H), 2.71–2.66 (m, 2H), 1.88–1.81 (m, 2H), 1.62–1.55 (m, 2H), 1.31–1.24 (m, 6H), 0.98 (t, J = 7.4 Hz, 3H), 0.84 (t, J = 7.0 Hz, 3H). **^{13}C{H} NMR** (126 MHz, $CDCl_3$): δ = 162.5 (C_q), 152.7 (C_q), 144.8 (C_q), 138.2 (C_q), 137.6 (C_q), 137.3 (C_q), 129.7 (CH), 129.0 (CH), 128.0 (CH), 126.7 (CH), 123.9 (CH), 115.9 (C_q), 112.6 (CH), 112.6

(CH), 103.0 (C_q), 50.5 (CH_2), 40.6 (CH_3), 40.6 (CH_3), 39.6 (CH_2), 35.1 (CH_2), 31.3 (CH_2), 30.6 (CH_2), 30.3 (CH_2), 26.3 (CH_2), 23.2 (CH_2), 22.6 (CH_2), 14.8 (CH_3), 14.1 (CH_3). **IR** (ATR): 2952, 2927, 1633, 1609, 1579, 1395, 1187, 826, 792, 698 cm^{-1}. **MS** (EI) *m/z* (relative intensity): 485 (57) $[M]^+$, 457 (38), 372 (100), 319 (63), 291 (83), 91 (34). **HR-MS** (ESI) *m/z* calcd for $C_{30}H_{40}N_5O$ $[M+H]^+$ 486.3227, found 486.3224.

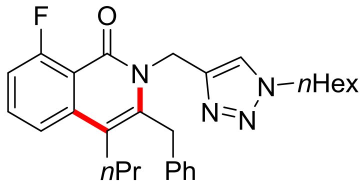

3-Benzyl-8-fluoro-2-[(1-*n*-hexyl-1*H*-1,2,3-triazol-4-yl)methyl]-4-*n*-propylisoquinolin-1(2*H*)-one (3pa): The general procedure was followed using **1p** (91.3 mg, 0.30 mmol) and alkyne **2a** (130 mg, 0.60 mmol). Purification by column chromatography (*n*hexane/EtOAc = 3/2) yielded **3pa** (80.2 mg, 58%) as a white solid. **M.p.** = 133–135 °C. **^{1}H NMR** (600 MHz, $CDCl_3$): δ = 7.83 (s, 1H), 7.61 (ddd, J = 8.1, 8.1, 5.1 Hz, 1H), 7.48 (d, J = 8.3 Hz, 1H), 7.36–7.32 (m, 2H), 7.28–7.24 (m, 3H), 7.11 (dd, J = 11.5, 8.0 Hz, 1H), 5.12 (br s, 2H), 4.65 (br s, 2H), 4.26 (t, J = 7.3 Hz, 2H), 2.73–2.67 (m, 2H), 1.89–1.82 (m, 2H), 1.59–1.50 (m, 2H), 1.32–1.24 (m, 6H), 0.98 (t, J = 7.3 Hz, 3H), 0.84 (t, J = 7.0 Hz, 3H). **^{13}C{H} NMR** (126 MHz, $CDCl_3$): δ = 162.9 (d, $^1J_{C-F}$ = 262.9 Hz, C_q), 160.0 (d, $^3J_{C-F}$ = 4.8 Hz, C_q), 143.9 (C_q), 139.6 (C_q), 138.7 (C_q), 137.1 (C_q), 133.0 (d, $^3J_{C-F}$ = 10.3 Hz, CH), 129.1 (CH), 127.9 (CH), 127.0 (CH), 124.5 (CH), 118.9 (d, $^4J_{C-F}$ = 4.4 Hz, CH), 115.5 (d, $^4J_{C-F}$ = 1.9 Hz, C_q), 114.6 (d, $^3J_{C-F}$ = 4.1 Hz, C_q), 113.0 (d, $^2J_{C-F}$ = 22.1 Hz, CH), 50.5 (CH_2), 39.8 (CH_2), 35.1 (CH_2), 31.3 (CH_2), 30.9 (CH_2), 30.3 (CH_2), 26.4 (CH_2), 23.4 (CH_2), 22.6 (CH_2), 14.6 (CH_3), 14.1 (CH_3). **^{19}F NMR** (282 MHz, $CDCl_3$): δ = -110.53 (dd, J = 11.7, 5.1 Hz). **IR** (ATR): 3130, 2957, 2928, 1645, 1596, 1306, 1051, 811, 768, 708 cm^{-1}. **MS** (EI) *m/z* (relative intensity): 460 (55) $[M]^+$, 347 (24), 294 (100), 266 (63), 242 (28), 112 (34). **HR-MS** (EI) *m/z* calcd for $C_{28}H_{33}FN_4O[M]^+$460.2638 found 460.2620.

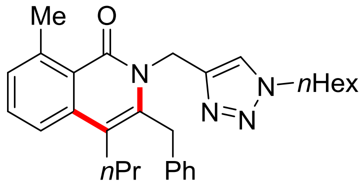

3-Benzyl-2-[(1-*n*-hexyl-1*H*-1,2,3-triazol-4-yl)methyl]-8-methyl-4-*n*-propylisoquinolin-1(2*H*)-one (3qa): The general procedure was followed using **1q** (90.1 mg, 0.30 mmol) and

alkyne **2a** (130 mg, 0.60 mmol). Purification by column chromatography (*n*hexane/EtOAc = 3/2) yielded **3qa** (46.3 mg, 34%) as a pale yellow solid. **M.p.** = 101–102 °C. **^{1}H NMR** (600 MHz, $CDCl_3$): δ = 7.71 (s, 1H), 7.57 (d, *J* = 8.1 Hz, 1H), 7.54–7.51 (m, 1H), 7.35–7.32 (m, 2H), 7.27–7.23 (m, 4H), 5.14 (br s, 2H), 4.56 (br s, 2H), 4.27 (t, *J* = 7.3 Hz, 2H), 2.97 (s, 3H), 2.73–2.67 (m, 2H), 1.89–1.82 (m, 2H), 1.59–1.52 (m, 2H), 1.31–1.25 (m, 6H), 0.98 (t, *J* = 7.3 Hz, 3H), 0.85 (t, *J* = 7.0 Hz, 3H). **^{13}C{H} NMR** (126 MHz, $CDCl_3$): δ = 163.4 (C_q), 144.6 (C_q), 142.1 (C_q), 138.3 (C_q), 137.5 (C_q), 137.1 (C_q), 131.5 (CH), 129.5 (CH), 129.1 (CH), 128.0 (CH), 126.8 (CH), 124.0 (C_q), 123.9 (CH), 121.3 (CH), 116.0 (C_q), 50.5 (CH_2), 40.0 (CH_2), 35.1 (CH_2), 31.3 (CH_2), 30.9 (CH_2), 30.3 (CH_2), 26.4(CH_2), 24.9 (CH_3), 23.4 (CH_2), 22.6 (CH_2), 14.6 (CH_3), 14.1 (CH_3). **IR** (ATR): 2956, 2928, 2869, 1770, 1644, 1600, 1305, 1049, 785, 697 cm^{-1}. **MS** (EI) *m/z* (relative intensity): 456 (96) [M]$^+$, 343 (22), 290 (100), 262 (91), 242 (42), 112 (50). **HR-MS** (EI) *m/z* calcd for $C_{29}H_{36}N_4O$ [M]$^+$ 456.2889, found 456.2896.

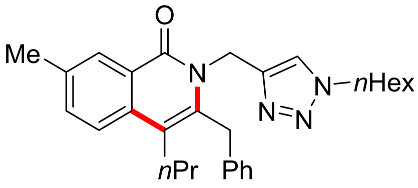

3-Benzyl-2-[(1-*n*-hexyl-1*H*-1,2,3-triazol-4-yl)methyl]-7-methyl-4-*n*-propylisoquinolin-1(2*H*)-one (3ra): The general procedure was followed using **1r** (90.1 mg, 0.30 mmol) and alkyne **2a** (130 mg, 0.60 mmol). Purification by column chromatography (*n*hexane/EtOAc = 3/2) yielded **3ra** (107 mg, 78%) as a white solid. **M.p.** = 136–138 °C. **^{1}H NMR** (600 MHz, $CDCl_3$): δ = 8.29 (s, 1H), 7.73 (s, 1H), 7.62 (d, *J* = 8.4 Hz, 1H), 7.52 (dd, *J* = 8.4, 1.9 Hz, 1H), 7.35–7.31 (m, 2H), 7.27–7.23 (m, 3H), 5.18 (br s, 2H), 4.59 (br s, 2H), 4.25 (t, *J* = 7.3 Hz, 2H), 2.74–2.69 (m, 2H), 2.50 (s, 3H), 1.88–1.81 (m, 2H), 1.59–1.52 (m, 2H), 1.32–1.24 (m, 6H), 0.98 (t, *J* = 7.3 Hz, 3H), 0.84 (t, *J* = 6.9 Hz, 3H). **^{13}C{H} NMR** (126 MHz, $CDCl_3$): δ = 162.7 (C_q), 144.3 (C_q), 137.6 (C_q), 136.1 (C_q), 136.1 (C_q), 134.5 (C_q), 133.9 (CH), 129.1 (CH), 128.0 (CH), 127.8 (CH), 126.8 (CH), 125.2 (C_q), 124.0 (CH), 123.2 (CH), 116.4 (C_q), 50.5 (CH_2), 40.0 (CH_2), 34.9 (CH_2), 31.3 (CH_2), 30.5 (CH_2), 30.3 (CH_2), 26.4 (CH_2), 23.7 (CH_2), 22.6 (CH_2), 21.4 (CH_3), 14.6 (CH_3), 14.1 (CH_3). **IR** (ATR): 2949, 2867, 1649, 1600, 1341, 1057, 841, 797, 724, 465 cm^{-1}. **MS** (EI) *m/z* (relative intensity): 456 (100) [M]$^+$, 290 (97), 262 (94), 242 (39), 215 (36), 112 (48). **HR-MS** (EI) *m/z* calcd for $C_{29}H_{36}N_4O$ [M]$^+$ 456.2889, found 456.2889.

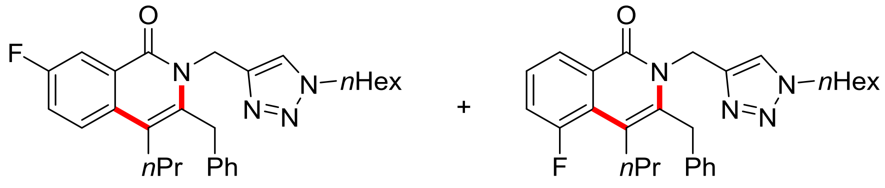

3sa **3sa'**

The general procedure was followed using **1s** (91.3 mg, 0.30 mmol) and alkyne **2a** (130 mg, 0.60 mmol). Purification by column chromatography (*n*hexane/EtOAc = 3/2) yielded **3sa** (51.5 mg, 37%) and **3sa'** (58.2 mg, 42%) as white solids.

3-Benzyl-7-fluoro-2-[(1-*n*-hexyl-1*H*-1,2,3-triazol-4-yl)methyl]-4-*n*-propylisoquinolin-1(2*H*)-one (3sa): M.p. = 141–143 °C. **^{1}H NMR** (600 MHz, $CDCl_3$): δ = 8.11 (dd, J = 9.3, 2.9 Hz, 1H), 7.73 (s, 1H), 7.71 (dd, J = 9.1, 5.0 Hz, 1H), 7.42 (ddd, J = 9.1, 8.1, 2.9 Hz, 1H), 7.34 (dd, J = 7.5, 7.5 Hz, 2H), 7.28–7.25 (m, 3H), 5.17 (br s, 2H), 4.61 (br s, 2H), 4.27 (t, J = 7.3 Hz, 2H), 2.75–2.70 (m, 2H), 1.89–1.82 (m, 2H), 1.59–1.51 (m, 2H), 1.32–1.24 (m, 6H), 0.99 (t, J = 7.3 Hz, 3H), 0.84 (t, J = 7.0 Hz, 3H). **^{13}C{H} NMR** (126 MHz, $CDCl_3$): δ = 161.9 (d, $^4J_{C\text{-}F}$ = 3.4 Hz, C_q), 161.0 (d, $^1J_{C–F}$ = 246.7 Hz, C_q), 144.0 (C_q), 137.3 (C_q), 136.4 (d, $^4J_{C–F}$ = 2.4 Hz, C_q), 133.4 (C_q), 129.1 (CH), 127.9 (CH), 127.0 (CH), 126.9 (d, $^3J_{C–F}$ = 7.9 Hz, C_q), 125.7 (d, $^3J_{C–F}$ = 7.6 Hz, CH), 124.0 (CH), 121.1 (d, $^2J_{C–F}$ = 23.3 Hz, CH), 116.0 (C_q), 113.2 (d, $^2J_{C–F}$ = 22.4 Hz, CH), 50.5 (CH_2), 40.1 (CH_2), 34.9 (CH_2), 31.3 (CH_2), 30.6 (CH_2), 30.3 (CH_2), 26.4 (CH_2), 23.7 (CH_2), 22.6 (CH_2), 14.6 (CH_3), 14.1 (CH_3). **^{19}F NMR** (282 MHz, $CDCl_3$): δ = -114.57 (ddd, J = 9.3, 8.1, 5.0 Hz). **IR** (ATR): 2954, 2920, 1648, 1600, 1493, 1346, 1057, 825, 725, 542 cm^{-1}. **MS** (EI) *m/z* (relative intensity): 460 (83) [M]$^+$, 294 (92), 266 (100), 242 (61), 112 (62), 85 (48). **HR-MS** (EI) *m/z* calcd for $C_{28}H_{33}FN_4O$ [M]$^+$ 460.2638, found 460.2639.

3-Benzyl-5-fluoro-2-[(1-*n*-hexyl-1*H*-1,2,3-triazol-4-yl)methyl]-4-*n*-propylisoquinolin-1(2*H*)-one (3sa'): M.p. = 142–144 °C. **^{1}H NMR** (600 MHz, $CDCl_3$): δ = 8.32 (dd, J = 7.9, 1.4 Hz, 1H), 7.73 (s, 1H), 7.41 (ddd, J = 7.9, 7.9, 4.6 Hz, 1H), 7.38–7.33 (m, 3H), 7.29–7.26 (m, 3H), 5.17 (br s, 2H), 4.61 (br s, 2H), 4.27 (t, J = 7.3 Hz, 2H), 2.81 (s, 2H), 1.89–1.82 (m, 2H), 1.60–1.52 (m, 2H), 1.33–1.24 (m, 6H), 0.97 (t, J = 7.3 Hz, 3H), 0.85 (t, J = 7.0 Hz, 3H). **^{13}C{H} NMR** (126 MHz, $CDCl_3$): δ = 161.6 (d, $^4J_{C–F}$ = 3.0 Hz, C_q), 158.5 (d, $^1J_{C–F}$ = 252.6 Hz, C_q), 143.9 (C_q), 138.0 (C_q), 137.2 (C_q), 129.1 (CH), 127.9 (CH), 127.8 (d, $^3J_{C–F}$ = 3.9 Hz, C_q), 127.0 (CH), 126.6 (d, $^3J_{C–F}$ = 9.1 Hz, CH), 126.3 (d, $^3J_{C–F}$ = 9.9 Hz, C_q), 124.5 (d, $^4J_{C–F}$ = 3.5 Hz, CH), 124.0 (CH), 119.4 (d, $^2J_{C–F}$ = 25.1 Hz, CH), 114.4 (d, $^4J_{C–F}$ = 5.8 Hz, C_q), 50.5 (CH_2), 40.4 (CH_2), 34.6 (CH_2), 32.4 (d, $^4J_{C–F}$ = 12.2 Hz, CH_2), 31.3 (CH_2), 30.3 (CH_2),

26.4 (CH_2), 24.6 (d, $^5J_{C\text{–}F}$ = 3.7 Hz, CH_2), 22.6 (CH_2), 14.6 (CH_3), 14.1 (CH_3). **^{19}F NMR** (282 MHz, $CDCl_3$): δ = -114.13 (ddd, J = 4.2, 2.9, 1.6 Hz). **IR** (ATR): 2957, 2930, 1649, 1594, 1228, 1063, 1007, 782, 755, 726 cm^{-1}. **MS** (EI) *m/z* (relative intensity): 460 (75) $[M]^+$, 294 (82), 266 (100), 242 (58), 112 (61), 85 (47). **HR-MS** (EI) *m/z* calcd for $C_{28}H_{33}FN_4O[M]^+$460.2638, found 460.2641.

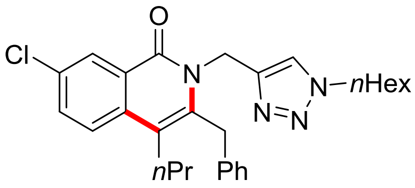

3-Benzyl-7-chloro-2-[(1-*n*-hexyl-1*H*-1,2,3-triazol-4-yl)methyl]-4-*n*-propylisoquinolin-1(2*H*)-one (3ta): The general procedure was followed using **1t** (96.2 mg, 0.30 mmol) and alkyne **2a** (130 mg, 0.60 mmol). Purification by column chromatography (*n*hexane/EtOAc = 3/2) yielded **3ta** (78.9 mg, 55%) as an off-white solid. **M.p.** = 138–139 °C. **^{1}H NMR** (600 MHz, $CDCl_3$): δ = 8.45 (d, J = 2.3 Hz, 1H), 7.72 (s, 1H), 7.66–7.61 (m, 2H), 7.36–7.32 (m, 2H), 7.28–7.23 (m, 3H), 5.17 (br s, 2H), 4.61 (s, 2H), 4.26 (t, J = 7.3 Hz, 2H), 2.73–2.69 (m, 2H), 1.89–1.83 (m, 2H), 1.58–1.51 (m, 2H), 1.31–1.25 (m, 6H), 0.98 (t, J = 7.3 Hz, 3H), 0.85 (t, J = 7.0 Hz, 3H). **^{13}C{H} NMR** (126 MHz, $CDCl_3$): δ = 161.7 (C_q), 143.9 (C_q), 137.6 (C_q), 137.1 (C_q), 135.1 (C_q), 132.8 (CH), 132.2 (C_q), 129.2 (CH), 127.9 (CH), 127.7 (CH), 127.0 (CH), 126.4 (C_q), 125.0 (CH), 124.1 (CH), 116.0 (C_q), 50.5 (CH_2), 40.1 (CH_2), 35.0 (CH_2), 31.3 (CH_2), 30.5 (CH_2), 30.3 (CH_2), 26.4 (CH_2), 23.6 (CH_2), 22.6 (CH_2), 14.6 (CH_3), 14.1 (CH_3). **IR** (ATR): 3134, 2953, 2923, 1648, 1595, 1304, 1055, 823, 724, 669 cm^{-1}. **MS** (EI) *m/z* (relative intensity): 476 (61) $[^{35}Cl, M]^+$, 310 (80), 282 (86), 242 (71), 112 (97), 69 (100). **HR-MS** (EI) *m/z* calcd for $C_{28}H_{33}{}^{35}ClN_4O[M]^+$ 476.2343, found 476.2345.

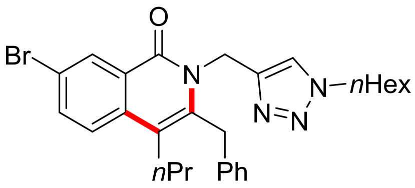

3-Benzyl-7-bromo-2-[(1-*n*-hexyl-1*H*-1,2,3-triazol-4-yl)methyl]-4-*n*-propylisoquinolin-1(2*H*)-one (3ua): The general procedure was followed using **1u** (110 mg, 0.30 mmol) and alkyne **2a** (130 mg, 0.60 mmol). Purification by column chromatography (*n*hexane/EtOAc = 3/2) yielded **3ua** (113 mg, 72%) as a white solid. **M.p.** = 143–144 °C. **^{1}H NMR** (600 MHz, $CDCl_3$): δ = 8.61 (d, J = 2.2 Hz, 1H), 7.76 (dd, J = 8.8, 2.2 Hz, 1H), 7.72

(s, 1H), 7.58 (d, *J* = 8.8 Hz, 1H), 7.34 (dd, *J* = 7.5, 7.5 Hz, 2H), 7.28–7.23 (m, 3H), 5.17 (br s, 2H), 4.61 (br s, 2H), 4.26 (t, *J* = 7.3 Hz, 2H), 2.73–2.68 (m, 2H), 1.89–1.82 (m, 2H), 1.58–1.49 (m, 2H), 1.32–1.25 (m, 6H), 0.98 (t, *J* = 7.3 Hz, 3H), 0.85 (t, *J* = 7.0 Hz, 3H). **^{13}C{H} NMR** (126 MHz, $CDCl_3$): δ = 161.7 (C_q), 143.9 (C_q), 137.9 (C_q), 137.1 (C_q), 135.6 (CH), 135.5 (C_q), 130.9 (CH), 129.2 (CH), 128.0 (CH), 127.1 (CH), 126.7 (C_q), 125.2 (CH), 124.2 (CH), 120.1 (C_q), 116.1 (C_q), 50.5 (CH_2), 40.1 (CH_2), 34.9 (CH_2), 31.2 (CH_2), 30.3 (CH_2), 30.2 (CH_2), 26.3 (CH_2), 23.5 (CH_2), 22.5 (CH_2), 14.5 (CH_3), 14.0 (CH_3). **IR** (ATR): 2949, 2916, 1647, 1596, 1583, 1377, 1055, 813, 786, 722 cm^{-1}. **MS** (ESI) *m/z* (relative intensity): 1065 (44) [^{81}Br, 2M+Na]$^+$, 523 (100) [^{81}Br, M+H]$^+$. **HR-MS** (EI) *m/z* calcd for $C_{28}H_{34}{}^{81}BrN_4O$ [M+H]$^+$ 523.1892, found 523.1890.

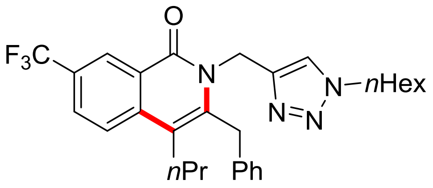

3-Benzyl-2-[(1-*n*-hexyl-1*H*-1,2,3-triazol-4-yl)methyl]-4-*n*-propyl-7-(trifluoromethyl)isoquinolin-1(2*H*)-one (3va): The general procedure was followed using **1v** (106 mg, 0.30 mmol) and alkyne **2a** (130 mg, 0.60 mmol). Purification by column chromatography (*n*hexane/EtOAc = 3/2) yielded **3va** (102 mg, 66%) as a white solid. **M.p.** = 116–117 °C. **^{1}H NMR** (300 MHz, $CDCl_3$): δ = 8.79 (s, 1H), 7.93–7.82 (m, 2H), 7.77 (s, 1H), 7.42–7.34 (m, 2H), 7.32–7.25 (m, 3H), 5.21 (br s, 2H), 4.69 (s, 2H), 4.30 (t, *J* = 7.3 Hz, 2H), 2.83–2.73 (m, 2H), 1.95–1.82 (m, 2H), 1.66–1.52 (m, 2H), 1.35–1.25 (m, 6H), 1.03 (t, *J* = 7.3 Hz, 3H), 0.87 (t, *J* = 6.7 Hz, 3H). **^{13}C{H} NMR** (126 MHz, $CDCl_3$): δ = 162.1 (C_q), 143.7 (C_q), 139.9 (C_q), 139.1 (C_q), 136.8 (C_q), 129.2 (CH), 128.4 (q, $^3J_{C–F}$ = 3.2 Hz, CH), 128.2 (C_q) 127.9 (CH), 127.1 (CH), 126.1 (q, $^3J_{C–F}$ = 3.6 Hz, CH), 125.0 (C_q), 124.2 (CH), 124.1 (CH), 124.1 (q, $^1J_{C–F}$ = 271.5 Hz, C_q), 116.0 (C_q), 50.6 (CH_2), 40.1 (CH_2), 35.2 (CH_2), 31.3 (CH_2), 30.4 (CH_2), 30.3 (CH_2), 26.4 (CH_2), 23.6 (CH_2), 22.6 (CH_2), 14.6 (CH_3), 14.1 (CH_3). **^{19}F NMR** (282 MHz, $CDCl_3$). δ = -62.38 (s). **IR** (ATR): 2931, 2872, 1655, 1622, 1323, 1297, 1130, 1030, 830, 714 cm^{-1}. **MS** (EI) *m/z* (relative intensity): 510 (63) [M]$^+$, 344 (72), 316 (76), 242 (57), 112 (88), 85 (70), 43 (100). **HR-MS** (ESI) *m/z* calcd for $C_{29}H_{34}F_3N_4O$ [M+H]$^+$ 511.2679, found 511.2676.

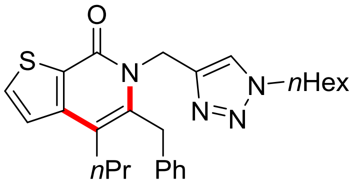

5-Benzyl-6-[(1-*n*-hexyl-1*H*-1,2,3-triazol-4-yl)methyl]-4-*n*-propylthieno[2,3-c]pyridin-7(6*H*)-one 3wa): The general procedure was followed using **1w** (87.7 mg, 0.30 mmol) and alkyne **2a** (130 mg, 0.60 mmol). Purification by column chromatography (*n*hexane/EtOAc = 3/2) yielded **3wa** (64.6 mg, 48%) as an off-white solid. **M.p.** = 100–102 °C. **^{1}H NMR** (600 MHz, $CDCl_3$): δ = 7.76 (s, 1H), 7.70 (d, J = 5.3 Hz, 1H), 7.35–7.31 (m, 2H), 7.29 (d, J = 5.3 Hz, 1H), 7.27–7.25 (m, 1H), 7.24–7.21 (m, 2H), 5.19 (br s, 2H), 4.60 (s, 2H), 4.26 (t, J = 7.4 Hz, 2H), 2.72–2.67 (m, 2H), 1.88–1.82 (m, 2H), 1.59–1.52 (m, 2H), 1.31–1.24 (m, 6H), 0.95 (t, J = 7.3 Hz, 3H), 0.85 (t, J = 6.9 Hz, 3H). **^{13}C{H} NMR** (126 MHz, $CDCl_3$): δ = 158.9 (C_q), 146.2 (C_q), 144.0 (C_q), 138.4 (C_q), 137.4 (C_q), 133.1 (CH), 129.1 (CH), 128.7 (C_q), 127.9 (CH), 126.9 (CH), 124.2 (CH), 123.3 (CH), 116.4 (C_q), 50.5 (CH_2), 39.7 (CH_2), 34.5 (CH_2), 32.3 (CH_2), 31.3 (CH_2), 30.3 (CH_2), 26.4 (CH_2), 24.0 (CH_2), 22.6 (CH_2), 14.5 (CH_3), 14.1 (CH_3). **IR** (ATR): 2930, 2866, 1646, 1576, 1450, 1131, 787, 718, 694, 457 cm^{-1}. **MS** (ESI) *m/z* (relative intensity): 919 (81) $[2M+Na]^+$, 897 (13) $[2M+H]^+$, 471 (45) $[M+Na]^+$, 449 (100) $[M+H]^+$. **HR-MS** (ESI) *m/z* calcd for $C_{26}H_{33}N_4OS$ $[M+H]^+$ 449.2370, found 449.2377.

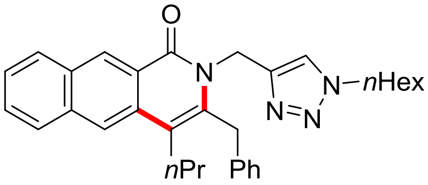

3-Benzyl-2-[(1-*n*-hexyl-1*H*-1,2,3-triazol-4-yl)methyl]-4-*n*-propylbenzo[g]isoquinolin-1(2*H*)-one (3xa): The general procedure was followed using **1x** (101 mg, 0.30 mmol) and alkyne **2a** (130 mg, 0.60 mmol). Purification by column chromatography (*n*hexane/EtOAc = 3/2) yielded **3xa** (82.4 mg, 56%) as a yellow solid. **M.p.** = 155–157 °C. **^{1}H NMR** (600 MHz, $CDCl_3$): δ = 9.09 (s, 1H), 8.15 (s, 1H), 8.05 (d, J = 8.3 Hz, 1H), 7.98 (d, J = 8.3 Hz, 1H), 7.75 (s, 1H), 7.59 (ddd, J = 8.1, 6.9, 1.0 Hz, 1H), 7.51 (ddd, J = 7.9, 6.9, 1.0 Hz, 1H), 7.36–7.30 (m, 4H), 7.28–7.25 (m, 1H), 5.21 (br s, 2H), 4.63 (s, 2H), 4.26 (t, J = 7.3 Hz, 2H), 2.89–2.83 (m, 2H), 1.89–1.82 (m, 2H), 1.71–1.63 (m, 2H), 1.31–1.24 (m, 6H), 1.06 (t, J = 7.3 Hz, 3H), 0.84 (t, J = 7.0 Hz, 3H). **^{13}C{H} NMR** (126 MHz, $CDCl_3$): δ = 163.3 (C_q), 144.3 (C_q), 137.5 (C_q), 135.8 (C_q), 135.5 (C_q), 132.8 (C_q), 131.4 (C_q), 129.4 (CH), 129.2

(CH), 129.1 (CH), 128.1 (CH), 128.1 (CH), 128.0 (CH), 126.9 (CH), 126.0 (CH), 124.0 (CH), 124.0 (C_q), 121.6 (CH), 116.3 (C_q), 50.6 (CH_2), 39.8 (CH_2), 35.1 (CH_2), 31.3 (CH_2), 30.7 (CH_2), 30.3 (CH_2), 26.3 (CH_2), 23.5 (CH_2), 22.6 (CH_2), 14.7 (CH_3), 14.1 (CH_3). **IR** (ATR): 2926, 1641, 1619, 1380, 1218, 1047, 882, 795, 728, 477 cm^{-1}. **MS** (EI) *m/z* (relative intensity): 492 (69) $[M]^+$, 401 (17), 326 (75), 298 (69), 112 (33), 91 (53). **HR-MS** (EI) *m/z* calcd for $C_{32}H_{36}N_4O$ $[M]^+$ 492.2889, found 492.2889.

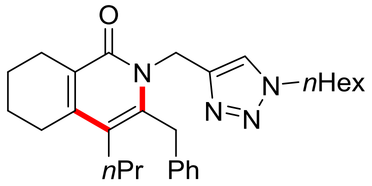

3-Benzyl-2-[(1-*n*-hexyl-1*H*-1,2,3-triazol-4-yl)methyl]-4-*n*-propyl-5,6,7,8-tetrahydroiso-quinolin-1(2H)-one (3ya): The general procedure was followed using **1y** (87.1 mg, 0.30 mmol) and alkyne **2a** (130 mg, 0.60 mmol). Purification by column chromatography (*n*hexane/EtOAc = 3/2) yielded **3ya** (29.7 mg, 22%) as a yellow oil. **^{1}H NMR** (600 MHz, $CDCl_3$): δ = 7.75 (s, 1H), 7.34–7.30 (m, 2H), 7.25–7.22 (m, 1H), 7.18 (d, J = 7.3 Hz, 2H), 5.08 (br s, 2H), 4.50 (s, 2H), 4.25 (t, J = 7.4 Hz, 2H), 2.58 (q, J = 6.5 Hz, 4H), 2.37–2.32 (m, 2H), 1.88–1.82 (m, 2H), 1.79–1.72 (m, 4H), 1.41–1.34 (m, 2H), 1.31–1.26 (m, 6H), 0.90 (t, J = 7.3 Hz, 3H), 0.85 (t, J = 7.0 Hz, 3H). **^{13}C{H} NMR** (126 MHz, $CDCl_3$): δ = 162.2 (C_q), 147.0 (C_q), 143.9 (C_q), 139.3 (C_q), 137.4 (C_q), 129.0 (CH), 127.9 (CH), 126.8 (CH), 125.7 (C_q), 124.3 (CH), 119.7 (C_q), 50.5 (CH_2), 40.0 (CH_2), 34.7 (CH_2), 31.3 (CH_2), 30.3 (CH_2), 30.3 (CH_2), 27.1 (CH_2), 26.3 (CH_2), 24.7 (CH_2), 24.0 (CH_2), 22.7 (CH_2), 22.6 (CH_2), 22.1 (CH_2), 14.6 (CH_3), 14.1 (CH_3). **IR** (ATR): 2951, 2923, 1644, 1591, 1484, 1302, 1302, 1056, 821, 723 cm^{-1}. **MS** (EI) *m/z* (relative intensity): 446 (57) $[M]^+$, 418 (35), 333 (95), 280 (100), 252 (40), 91 (46). **HR-MS** (EI) *m/z* calcd for $C_{28}H_{38}N_4O$ $[M]^+$ 446.3046, found 446.3045.

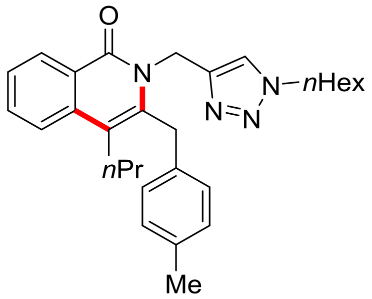

2-[(1-*n*-Hexyl-1*H*-1,2,3-triazol-4-yl)methyl]-3-(4-methylbenzyl)-4-*n*-propylisoquinolin-1(2*H*)-one (3ab): The general procedure was followed using **1a** (85.9 mg, 0.30 mmol) and

alkyne **2b** (138 mg, 0.60 mmol). Purification by column chromatography (*n*hexane/EtOAc = 3/2) yielded **3ab** (118 mg, 86%) as a colorless oil. **^{1}H NMR** (300 MHz, $CDCl_3$): δ = 8.48 (d, J = 7.8 Hz, 1H), 7.75–7.66 (m, 3H), 7.48 (ddd, J = 8.1, 6.0, 2.1 Hz, 1H), 7.14 (s, 4H), 5.19 (br s, 2H), 4.56 (s, 2H), 4.26 (t, J = 7.3 Hz, 2H), 2.77–2.69 (m, 2H), 2.33 (s, 3H), 1.91–1.80 (m, 2H), 1.64–1.50 (m, 2H), 1.33–1.22 (m, 6H), 0.99 (t, J = 7.3 Hz, 3H), 0.84 (t, J = 6.8 Hz, 3H). **^{13}C{H} NMR** (75 MHz, $CDCl_3$): δ = 162.8 (C_q), 144.3 (C_q), 137.4 (C_q), 136.9 (C_q), 136.6 (C_q), 134.3 (C_q), 132.5 (CH), 129.8 (CH), 128.3 (CH), 127.9 (CH), 126.2 (CH), 125.3 (C_q), 124.1 (CH), 123.2 (CH), 116.4 (C_q), 50.5 (CH_2), 39.9 (CH_2), 34.5 (CH_2), 31.2 (CH_2), 30.4 (CH_2), 30.2 (CH_2), 26.3 (CH_2), 23.6 (CH_2), 22.5 (CH_2), 21.2 (CH_3), 14.5 (CH_3), 14.0 (CH_3). **IR** (ATR): 2955, 2928, 2869, 1641, 1610, 1590, 1336, 1047, 772, 729 cm^{-1}. **MS** (EI) *m/z* (relative intensity): 456 (100) $[M]^+$, 343 (24), 290 (88), 262 (50), 256 (84), 112 (53), 85 (50). **HR-MS** (EI) *m/z* calcd for $C_{29}H_{36}N_4O$ $[M]^+$ 456.2889, found 456.2885.

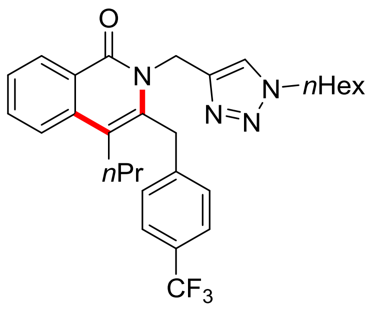

2-[(1-*n*-Hexyl-1*H*-1,2,3-triazol-4-yl)methyl]-4-*n*-propyl-3-[4-(trifluoromethyl)benzyl]iso-quinolin-1(2*H*)-one 3ac): The general procedure was followed using **1a** (85.9 mg, 0.30 mmol) and alkyne **2c** (171 mg, 0.60 mmol). Purification by column chromatography (*n*hexane/EtOAc = 3/2) yielded **3ac** (81.3 mg, 53%) as sticky yellow oil. **^{1}H NMR** (300 MHz, $CDCl_3$): δ = 8.49 (d, J = 8.0 Hz, 1H), 7.77–7.70 (m, 3H), 7.60 (d, J = 8.0 Hz, 2H), 7.50 (ddd, J = 8.0, 5.0, 3.2 Hz, 1H), 7.39 (d, J = 8.0 Hz, 2H), 5.13 (br s, 2H), 4.69 (s, 2H), 4.26 (t, J = 7.3 Hz, 2H), 2.76–2.67 (m, 2H), 1.91–1.80 (m, 2H), 1.60–1.49 (m, 2H), 1.32–1.22 (m, 6H), 1.00 (t, J = 7.2 Hz, 3H), 0.84 (t, J = 6.8 Hz, 3H). **^{13}C{H} NMR** (126 MHz, $CDCl_3$): δ = 162.6 (C_q), 143.9 (C_q), 141.7 (C_q), 136.6 (C_q), 136.1 (C_q), 132.6 (CH), 129.4 (q, $^{2}J_{C–F}$ = 32.4 Hz, C_q), 128.3 (CH), 128.3 (CH), 126.5 (CH), 126.0 (q, $^{3}J_{C–F}$ = 3.7 Hz, CH), 125.4 (C_q), 124.2 (q, $^{1}J_{C–F}$ = 271.5 Hz, C_q), 124.2 (CH), 123.2 (CH), 116.8 (C_q), 50.6 (CH_2), 40.0 (CH_2), 34.9 (CH_2), 31.3 (CH_2), 30.5 (CH_2), 30.3 (CH_2), 26.3 (CH_2), 23.6 (CH_2), 22.6 (CH_2), 14.6 (CH_3), 14.1 (CH_3). **^{19}F NMR** (282 MHz, $CDCl_3$): δ = -62.47 (s). **IR** (ATR): 2957, 2931, 1643, 1611, 1592, 1322, 1162, 1121, 1066, 772 cm^{-1}. **MS** (ESI) *m/z* (relative intensity): 1043 (86) $[2M+Na]^+$, 901

(5), 777 (3), 511 (100) [M+H]$^+$. **HR-MS** (ESI) *m/z* calcd for $C_{29}H_{34}F_3N_4O$ [M+H]$^+$511.2679, found 511.2674.

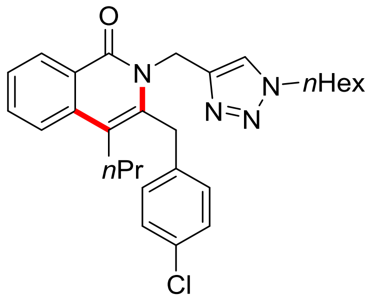

3-(4-Chlorobenzyl)-2-[(1-*n*-hexyl-1*H*-1,2,3-triazol-4-yl)methyl]-4-*n*-propylisoquinolin-1(2*H*)-one (3ad): The general procedure was followed using **1a** (85.9 mg, 0.30 mmol) and alkyne **2d** (150 mg, 0.60 mmol). Purification by column chromatography (*n*hexane/EtOAc = 3/2) yielded **3ad** (111 mg, 78%) as a yellow oil. **^{1}H NMR** (600 MHz, $CDCl_3$): δ = 8.48 (d, J = 7.9 Hz, 1H), 7.74 (s, 1H), 7.73–7.68 (m, 2H), 7.49 (ddd, J = 8.1, 6.0, 2.1 Hz, 1H), 7.30 (d, J = 8.4 Hz, 2H), 7.19 (d, J = 8.4 Hz, 2H), 5.15 (br s, 2H), 4.58 (s, 2H), 4.26 (t, J = 7.3 Hz, 2H), 2.73–2.68 (m, 2H), 1.88–1.82 (m, 2H), 1.59–1.52 (m, 2H), 1.31–1.24 (m, 6H), 0.99 (t, J = 7.3 Hz, 3H), 0.84 (t, J = 6.9 Hz, 3H). **^{13}C{H} NMR** (126 MHz, $CDCl_3$): δ = 162.7 (C_q), 144.0 (C_q), 136.6 (C_q), 136.6 (C_q), 135.9 (C_q), 132.8 (C_q), 132.5 (CH), 129.3 (CH), 129.2 (CH), 128.3 (CH), 126.3 (CH), 125.3 (C_q), 124.1 (CH), 123.2 (CH), 116.6 (C_q), 50.6 (CH_2), 39.9 (CH_2), 34.4 (CH_2), 31.3 (CH_2), 30.4 (CH_2), 30.3 (CH_2), 26.3(CH_2), 23.6 (CH_2), 22.6 (CH_2), 14.6 (CH_3), 14.1 (CH_3). **IR** (ATR): 3120, 2956, 2927, 1643, 1589, 1489, 1340, 1091, 769,482 cm^{-1}. **MS** (ESI) *m/z* (relative intensity): 975 (61) [2M+Na]$^+$, 833 (5), 727 (5), 477 (100) [M+H]$^+$. **HR-MS** (ESI) *m/z* calcd for $C_{28}H_{34}{}^{35}ClN_4O$ [M+H]$^+$ 477.2416, found 477.2410.

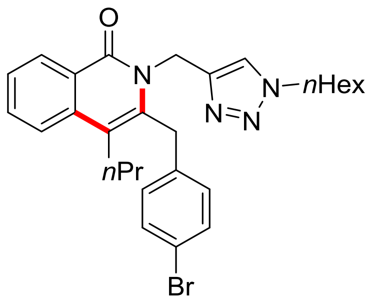

3-(4-Bromobenzyl)-2-[(1-*n*-hexyl-1*H*-1,2,3-triazol-4-yl)methyl]-4-*n*-propylisoquinolin-1(2*H*)-one (3ae): The general procedure was followed using **1a** (85.9 mg, 0.30 mmol) and alkyne **2e** (177 mg, 0.60 mmol). Purification by column chromatography

(*n*hexane/EtOAc = 3/2) yielded **3ae** (137 mg, 88%) as a white solid. **M.p.** = 51–53 °C. **^{1}H NMR** (300 MHz, $CDCl_3$): δ = 8.48 (d, J = 8.0 Hz, 1H), 7.77–7.66 (m, 3H), 7.54–7.41 (m, 3H), 7.14 (d, J = 8.0 Hz, 2H), 5.14 (br s, 2H), 4.56 (s, 2H), 4.26 (t, J = 7.3 Hz, 2H), 2.76–2.64 (m, 2H), 1.92–1.79 (m, 2H), 1.63–1.48 (m, 2H), 1.34–1.21 (m, 6H), 0.99 (t, J = 7.3 Hz, 3H), 0.84 (t, J = 6.0 Hz, 3H). **^{13}C{H} NMR** (75 MHz, $CDCl_3$): δ = 162.8 (C_q), 144.1 (C_q), 136.7 (C_q), 136.6 (C_q), 136.5 (C_q), 132.6 (CH), 132.3 (CH), 129.8 (CH), 128.4 (CH), 126.4 (CH), 125.4 (C_q), 124.2 (CH), 123.2 (CH), 120.9 (C_q), 116.6 (C_q), 50.5 (CH_2), 39.9 (CH_2), 34.4 (CH_2), 31.2 (CH_2), 30.4 (CH_2), 30.2 (CH_2), 26.3 (CH_2), 23.6 (CH_2), 22.5 (CH_2), 14.5 (CH_3), 14.0 (CH_3). **IR** (ATR): 2954, 2928, 2869, 1641, 1590, 1486, 1010, 770, 727, 699 cm^{-1}. **MS** (EI) *m/z* (relative intensity): 522 (67) [^{81}Br, M]$^+$, 356 (47), 320 (50), 246 (100), 112 (61), 85 (60). **HR-MS** (EI) *m/z* calcd for $C_{28}H_{33}{}^{81}BrN_4O$ [M]$^+$ 522.1824, found 522.1829.

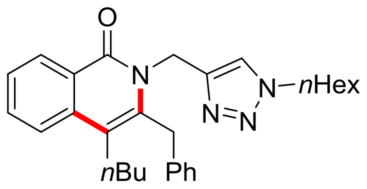

3-Benzyl-4-*n*-butyl-2-[(1-*n*-hexyl-1*H*-1,2,3-triazol-4-yl)methyl]isoquinolin-1(2*H*)-one (3af): The general procedure was followed using **1a** (85.9 mg, 0.30 mmol) and alkyne **2f** (138 mg, 0.60 mmol). Purification by column chromatography (*n*hexane/EtOAc = 3/2) yielded **3af** (48.5 mg, 35%) as an off-white solid. **M.p.** = 97–99 °C. **^{1}H NMR** (400 MHz, $CDCl_3$): δ = 8.49 (d, J = 7.9 Hz, 1H), 7.75–7.68 (m, 3H), 7.48 (ddd, J = 8.1, 6.4, 1.7 Hz, 1H), 7.36–7.31 (m, 2H), 7.29–7.23 (m, 3H), 5.19 (br s, 2H), 4.61 (s, 2H), 4.26 (t, J = 7.3 Hz, 2H), 2.79–2.72 (m, 2H), 1.90–1.82 (m, 2H), 1.57–1.48 (m, 2H), 1.46–1.36 (m, 2H), 1.32–1.24 (m, 6H), 0.91 (t, J = 7.2 Hz, 3H), 0.85 (t, J = 6.9 Hz, 3H). **^{13}C{H} NMR** (101 MHz, $CDCl_3$): δ = 162.8 (C_q), 144.3 (C_q), 137.5 (C_q), 137.1 (C_q), 136.8 (C_q), 132.5 (CH), 129.2 (CH), 128.4 (CH), 128.1 (CH), 127.0 (CH), 126.2 (CH), 125.4 (C_q), 124.1 (CH), 123.2 (CH), 116.6 (C_q), 50.5 (CH_2), 39.9 (CH_2), 34.9 (CH_2), 32.5 (CH_2), 31.2 (CH_2), 30.2 (CH_2), 28.0 (CH_2), 26.3 (CH_2), 23.2 (CH_2), 22.5 (CH_2), 14.0 (CH_3), 14.0 (CH_3). **IR** (ATR): 2954, 2928, 2858, 1650, 1597, 1313, 1031, 764, 714, 696 cm^{-1}. **MS** (ESI) *m/z* (relative intensity): 935 (80) [2M+Na]$^+$, 779 (6), 489 (20) [M+Na]$^+$, 457 (100) [M+H]$^+$. **HR-MS** (ESI) *m/z* calcd for $C_{29}H_{37}N_4O$ [M+H]$^+$ 457.2962, found 457.2953.

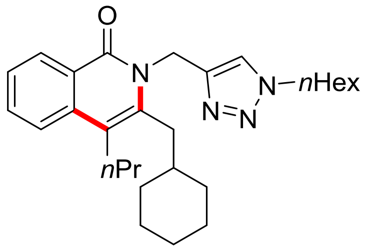

3-(Cyclohexylmethyl)-2-[(1-*n*-hexyl-1*H*-1,2,3-triazol-4-yl)methyl]-4-*n*-propylisoquinolin-1(2*H*)-one (3ag): The general procedure was followed using **1a** (85.9 mg, 0.30 mmol) and alkyne **2g** (133 mg, 0.60 mmol). Purification by column chromatography (*n*hexane/EtOAc = 3/2) yielded **3ag** (78.2 mg, 58%) as a white solid. **M.p.** = 126–128 °C. **^{1}H NMR** (600 MHz, $CDCl_3$): δ = 8.44 (d, J = 8.0 Hz, 1H), 7.65–7.63 (m, 3H), 7.42 (ddd, J = 8.0, 4.9, 3.2 Hz, 1H), 5.45 (br s, 2H), 4.23 (t, J = 7.3 Hz, 2H), 2.97 (s, 2H), 2.76–2.70 (m, 2H), 1.88–1.80 (m, 4H), 1.77–1.72 (m, 2H), 1.69–1.61 (m, 2H), 1.56–1.48 (m, 2H), 1.28–1.23 (m, 6H), 1.23–1.13 (m, 5H), 1.00 (t, J = 7.3 Hz, 3H), 0.83 (t, J = 6.9 Hz, 3H). **^{13}C{H} NMR** (126 MHz, $CDCl_3$): δ = 162.8 (C_q), 144.4 (C_q), 138.5 (C_q), 136.7 (C_q), 132.2 (CH), 128.2 (CH), 125.7 (CH), 124.9 (C_q), 123.7 (CH), 123.1 (CH), 115.5 (C_q), 50.5 (CH_2), 40.2 (CH_2), 39.4 (CH), 36.3 (CH_2), 33.4 (CH_2), 31.3 (CH_2), 30.4 (CH_2), 30.3 (CH_2), 26.8 (CH_2), 26.5 (CH_2), 26.3 (CH_2), 23.5 (CH_2), 22.6 (CH_2), 14.6 (CH_3), 14.1(CH_3). **IR** (ATR): 2925, 2851, 1639, 1608, 1588, 1451, 1382, 1043, 768, 699 cm^{-1}. **MS** (EI) *m/z* (relative intensity): 448 (79) $[M]^+$, 366 (72), 282 (100), 254 (55), 200 (56), 172 (70). **HR-MS** (ESI) *m/z* calcd for $C_{28}H_{41}N_4O$ $[M+H]^+$ 449.3275, found 449.3274.

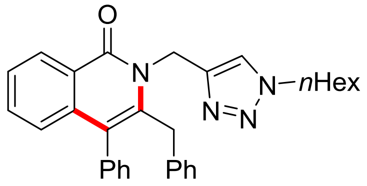

3-Benzyl-2-[(1-*n*-hexyl-1*H*-1,2,3-triazol-4-yl)methyl]-4-phenylisoquinolin-1(2*H*)-one (3ah): The general procedure was followed using **1a** (85.9 mg, 0.30 mmol) and alkyne **2h** (150 mg, 0.60 mmol). Purification by column chromatography (*n*hexane/EtOAc = 3/2) yielded **3ah** (112 mg, 78%) as a yellow oil. **^{1}H NMR** (300 MHz, $CDCl_3$): δ = 8.55 (d, J = 7.9 Hz, 1H), 7.65 (s, 1H), 7.61–7.46 (m, 3H), 7.45–7.41 (m, 2H), 7.32–7.27 (m, 2H), 7.26–7.12 (m, 4H), 7.09–7.00 (m, 2H), 5.12 (br s, 2H), 4.28 (t, J = 7.3 Hz, 2H), 3.85 (s, 2H), 1.94–1.82 (m, 2H), 1.36–1.26 (m, 6H), 0.88 (t, J = 6.8 Hz, 3H). **^{13}C{H} NMR** (126 MHz, $CDCl_3$): δ = 162.4 (C_q), 143.9 (C_q), 142.3 (C_q), 140.3 (C_q), 136.8 (C_q), 134.3 (C_q), 132.5 (CH), 129.9 (CH), 129.3 (CH), 128.8 (CH), 128.4 (CH), 128.2 (CH), 127.8 (CH), 126.6 (CH), 126.0 (CH), 125.8 (C_q),

124.5(CH), 123.6 (CH), 113.2 (C_q), 50.4 (CH_2), 42.4 (CH_2), 34.8 (CH_2), 31.3 (CH_2), 30.3 (CH_2), 26.3 (CH_2), 22.6 (CH_2), 14.1 (CH_3). **IR** (ATR): 2929, 2857, 1644, 1589, 1485, 1320, 1047, 762, 696, 524 cm^{-1}. **MS** (EI) *m/z* (relative intensity): 476 (96) $[M]^+$, 377 (25), 363 (53), 311 (100), 234 (46), 232 (44). **HR-MS** (ESI) *m/z* calcd for $C_{31}H_{33}N_4O$ $[M+H]^+$ 477.2649, found 477.2648.

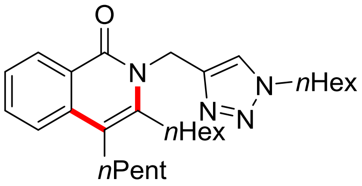

3-*n*-Hexyl-2-[(1-*n*-hexyl-1*H*-1,2,3-triazol-4-yl)methyl]-4-*n*-pentylisoquinolin-1(2*H*)-one (3ai): The general procedure was followed using **1a** (85.9 mg, 0.30 mmol) and alkyne **2i** (143 mg, 0.60 mmol). Purification by column chromatography (CH_2Cl_2/MeOH = 95/5) yielded **3ai** (88.6 mg, 64%) as a colorless oil. **^{1}H NMR** (600 MHz, $CDCl_3$): δ = 8.44 (d, J = 8.0 Hz, 1H), 7.71 (s, 1H), 7.66–7.61 (m, 2H), 7.42 (ddd, J = 8.0, 6.0, 2.0 Hz, 1H), 5.40 (br s, 2H), 4.25 (t, J = 7.3 Hz, 2H), 3.08–2.96 (m, 2H), 2.71–2.66 (m, 2H), 1.87–1.81 (m, 2H), 1.68–1.62 (m, 2H), 1.58–1.51 (m, 4H), 1.46–1.34 (m, 8H), 1.28–1.23 (m, 6H), 0.94–0.90 (m, 6H), 0.84 (t, J = 6.9 Hz, 3H). **^{13}C{H} NMR** (126 MHz, $CDCl_3$): δ = 162.7 (C_q), 144.4 (C_q), 140.2 (C_q), 136.9 (C_q), 132.3 (CH), 128.2 (CH), 125.7 (CH), 124.9 (C_q), 123.9 (CH), 122.8 (CH), 114.5 (C_q), 50.5 (CH_2), 40.0 (CH_2), 32.5 (CH_2), 31.8 (CH_2), 31.3 (CH_2), 30.3 (CH_2), 30.3 (CH_2), 30.1 (CH_2), 29.7 (CH_2), 29.7 (CH_2), 27.9 (CH_2), 26.3 (CH_2), 22.8 (CH_2), 22.7 (CH_2), 22.6 (CH_2), 14.3 (CH_3), 14.3 (CH_3), 14.1 (CH_3). **IR** (ATR): 2953, 2923, 2856, 1642, 1589, 1463, 1337, 1046, 772, 730 cm^{-1}. **MS** (ESI) *m/z* (relative intensity): 951 (100) $[2M+Na]^+$, 929 (31), 895 (9), 781 (10), 465 (92) $[M+H]^+$, 317 (15). **HR-MS** (ESI) *m/z* calcd for $C_{29}H_{45}N_4O$ $[M+H]^+$465.3588, found 465.3575.

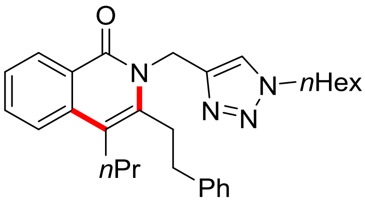

2-[(1-*n*-Hexyl-1*H*-1,2,3-triazol-4-yl)methyl]-3-phenethyl-4-*n*-propylisoquinolin-1(2*H*)-one (3aj): The general procedure was followed using **1a** (85.9 mg, 0.30 mmol) and alkyne **2j** (138 mg, 0.60 mmol). Purification by column chromatography (*n*hexane/EtOAc = 3/2) yielded

3aj (53.8 mg, 39%) as an off-white solid. **M.p.** = 106–108 °C. **^{1}H NMR** (600 MHz, $CDCl_3$): δ = 8.47 (d, J = 7.8 Hz, 1H), 7.72 (s, 1H), 7.68–7.64 (m, 2H), 7.45 (ddd, J = 8.1, 6.2, 1.9 Hz, 1H), 7.36–7.32 (m, 4H), 7.27–7.23 (m, 1H), 5.46 (br s, 2H), 4.25 (t, J = 7.3 Hz, 2H), 3.39 (s, 2H), 3.00–2.96 (m, 2H), 2.71–2.67 (m, 2H), 1.88–1.82 (m, 2H), 1.62–1.54 (m, 2H), 1.30–1.24 (m, 6H), 1.04 (t, J = 7.3 Hz, 3H), 0.84 (t, J = 7.0 Hz, 3H). **^{13}C{H} NMR** (126 MHz, $CDCl_3$): δ = 162.7 (C_q), 144.3 (C_q), 140.3 (C_q), 139.0 (C_q), 136.8 (C_q), 132.3 (CH), 128.8 (CH), 128.4 (CH), 128.3 (CH), 126.6 (CH), 125.9 (CH), 125.0 (C_q), 123.9 (CH), 123.0 (CH), 115.1 (C_q), 50.5 (CH_2), 40.1 (CH_2), 36.1 (CH_2), 31.5 (CH_2), 31.3 (CH_2), 30.3 (CH_2), 30.0 (CH_2), 26.3 (CH_2), 23.8 (CH_2), 22.6 (CH_2), 14.7 (CH_3), 14.1 (CH_3). **IR** (ATR): 2958, 2928, 2859, 1632, 1581, 1327, 1057, 772, 749, 694 cm^{-1}. **MS** (ESI) *m/z* (relative intensity): 935 (100) $[2M+Na]^+$, 913 (22) $[2M+H]^+$, 479 (36) $[M+Na]^+$, 457 (89) $[M+H]^+$. **HR-MS** (ESI) *m/z* calcd for $C_{29}H_{37}N_4O$ $[M+H]^+$ 457.2962, found 457.2946.

Competition Experiments

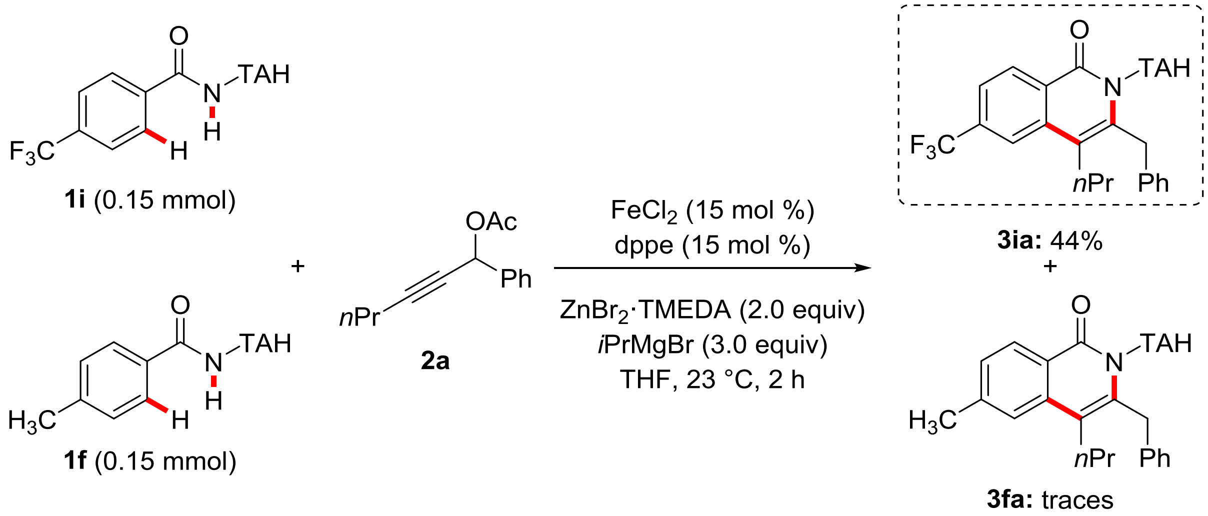

To a stirred solution of **1f** (45.1 mg, 0.15 mmol), **1i** (53.2 mg, 0.15 mmol), $ZnBr_2$·TMEDA (205 mg, 0.60 mmol) and dppe (17.9 mg, 15 mol %) in THF (0.4 mL), *i*PrMgBr (3.0 M in 2-MeTHF, 300 µL, 0.90 mmol) was added in one portion and the reaction mixture was stirred for 5 min at ambient temperature. Then, $FeCl_2$ (5.7 mg, 15 mol %) was added in a single portion. After stirring for additional 5 min, a solution of alkyne **2a** (130 mg, 0.60 mmol) in THF (0.40 mL) was added in one portion. The reaction mixture was stirred at ambient temperature. After 2 h, sat. aqueous NH_4Cl (3.0 mL) was added and the reaction mixture was extracted with CH_2Cl_2 (3 × 15 mL). The combined organic extracts were dried over Na_2SO_4, filtered and concentrated. Purification by column chromatography (*n*hexane/EtOAc = 3/2) yielded **3ia** (66.7 mg, 44%) as the sole product.

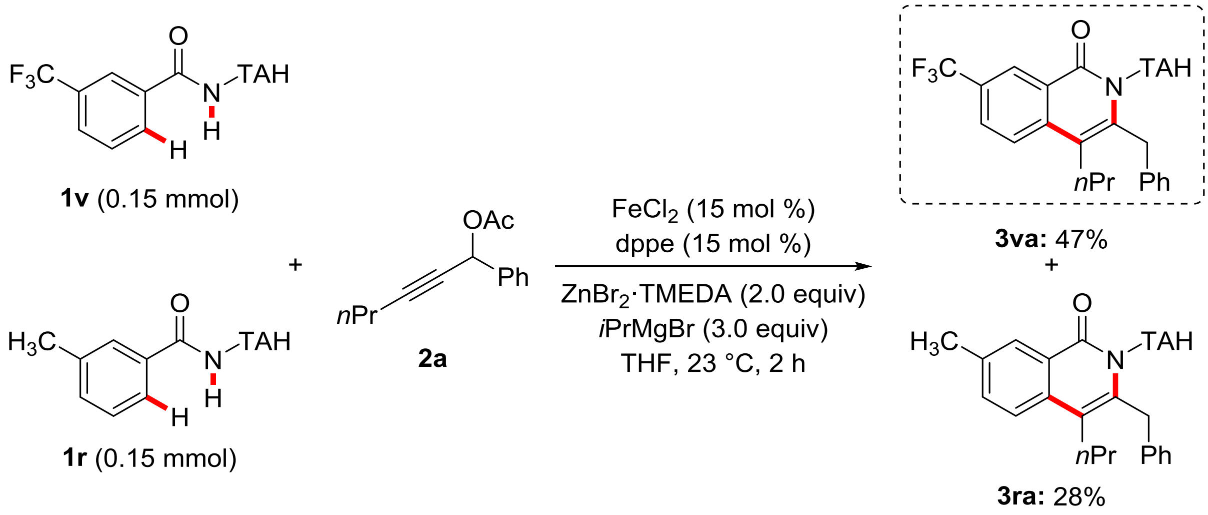

To a stirred solution of **1r** (45.1 mg, 0.15 mmol), **1v** (53.2 mg, 0.15 mmol), $ZnBr_2$·TMEDA (205 mg, 0.60 mmol) and dppe (17.9 mg, 15 mol %) in THF (0.4 mL), *i*PrMgBr (3.0 M in

2-MeTHF, 300 µL, 0.90 mmol) was added in one portion and the reaction mixture was stirred for 5 min at ambient temperature. Then, $FeCl_2$ (5.7 mg, 15 mol %) was added in a single portion. After stirring for additional 5 min, a solution of alkyne **2a** (130 mg, 0.60 mmol) in THF (0.40 mL) was added in one portion. The reaction mixture was stirred at ambient temperature. After 2 h, sat. aqueous NH_4Cl (3.0 mL) was added and the reaction mixture was extracted with CH_2Cl_2 (3 × 15 mL). The combined organic extracts were dried over Na_2SO_4, filtered and concentrated. Purification by column chromatography (*n*hexane/EtOAc = 3/2) yielded **3va** (71.9 mg, 47%) and **3ra** (37.9 mg, 28%).

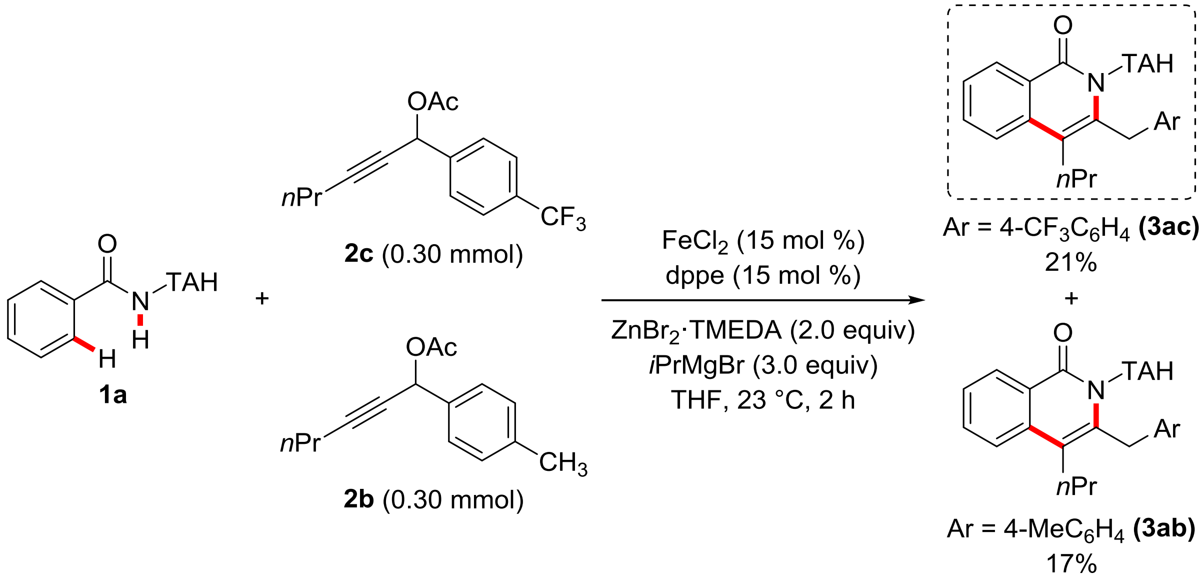

To a stirred solution of **1a** (85.9 mg, 0.30 mmol), $ZnBr_2$·TMEDA (205 mg, 0.60 mmol) and dppe (17.9 mg, 15 mol %) in THF (0.40 mL),*i*PrMgBr (3.0 M in 2-MeTHF, 300 µL, 0.90 mmol) was added in one portion and the reaction mixture was stirred for 5 min at ambient temperature. Then, $FeCl_2$ (5.7 mg, 15 mol %) was added in a single portion. After stirring for additional 5 min, a solution of alkyne **2b** (69.0 mg, 0.30 mmol) and **2c** (85.3 mg, 0.30 mmol) in THF (0.40 mL) was added in one portion. The reaction mixture was stirred at ambient temperature. After 2 h, sat. aqueous NH_4Cl (3.0 mL) was added and the reaction mixture was extracted with CH_2Cl_2 (3 × 15 mL). The combined organic extracts were dried over Na_2SO_4, filtered and concentrated. Purification by column chromatography (*n*hexane/EtOAc = 3/2) afforded a mixture of both products. The yields of **3ac** (21%) and **3ab** (17%) were determined by ^{1}H-NMR spectroscopy with 1,3,5-trimethoxybenzene as internal standard.

Hammett-Plot Analysis for *para*-Substituted Propargyl Benzoates

To a stirred solution of **1e** (296 mg, 1.00 mmol), $ZnBr_2$·TMEDA (683 mg, 2.00 mmol) and dppe (59.8 mg, 0.15 mmol, 15 mol %) in THF (1.3 mL), *i*PrMgBr (3.0 M in 2-MeTHF, 1.0 mL, 3.00 mmol) was added in one portion and the reaction mixture was stirred for 5 min at ambient temperature. Then, $FeCl_2$ (19.0 mg, 0.15 mmol, 15 mol %) was added in a single portion. After stirring for additional 5 min, a solution of propargyl benzoate **2** (2.00 mmol, 2.0 equiv) and 1-fluorononane (73.1 mg, 0.50 mmol, 0.5 equiv) as internal standard in THF (1.3 mL) was added in one portion. The reaction mixture was stirred at ambient temperature. After the times indicated below an aliquot of 0.1 mL was taken, filtered over a short plug of silica and diluted with $CDCl_3$ (0.7 mL). The yields of product were determined by ^{19}F NMR using 1-fluoro nonane as internal standard.

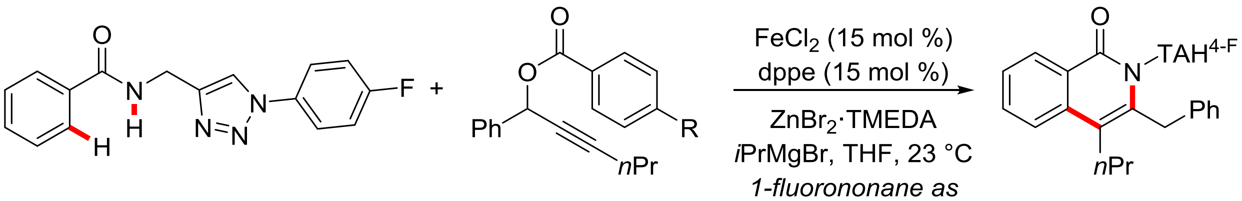

1e **2** **3ea**

Time [min]	5	10	20	30	45	60	90
**4-OMe [%]**	1.00	1.50	3.00	3.50	4.50	5.50	9.00
**4-tBu [%]**	0.99	1.49	2.48	3.71	4.95	6.68	9.65
**4-Me [%]**	2.00	3.50	4.50	6.50	7.00	9.50	13.00
**H [%]**	3.01	4.01	5.01	6.52	9.52	11.03	15.54
**4-Cl [%]**	2.00	2.50	3.50	4.50	6.00	7.50	10.00
**4-CF_3[%]**	0.50	1.00	2.00	2.50	3.50	4.00	6.00
**4-CN [%]**	0.50	1.00	1.50	2.00	2.50	3.50	5.00

R	r [min^{-1}]	σ_p	r [mol·L^{-1}·s^{-1}]	k_X/k_H	log(k_X/k_H)
**4-OMe**	0.0887	–0.268	0,00041065	0.601355932	–0.2208684
**4-tBu**	0.1021	–0.197	0,00047269	0.69220339	–0.159766278
**4-Me**	0.1231	–0,170	0,00056991	0.834576271	–0.078533967
**H**	0.1475	0.00	0,00068287	1	0
**4-Cl**	0.0952	0.227	0,00044074	0.645423729	–0.190155072
**4-CF_3**	0.062	0.54	0,00028704	0.420338983	–0.376400331
**4-CN**	0.0511	0.66	0,00023657	0.346440678	–0.46037112

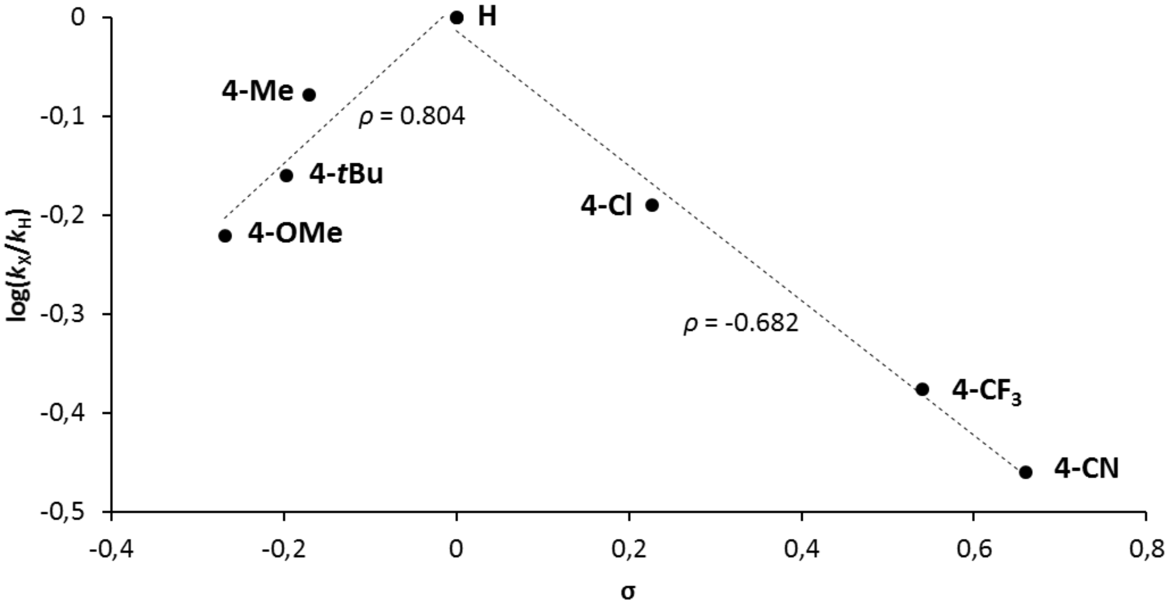

Figure S-1. Hammett plot correlation of differently *para*-substituted propargyl benzoates.

Intermolecular Kinetic Isotope Effect (KIE) Measurement by Independent Reactions

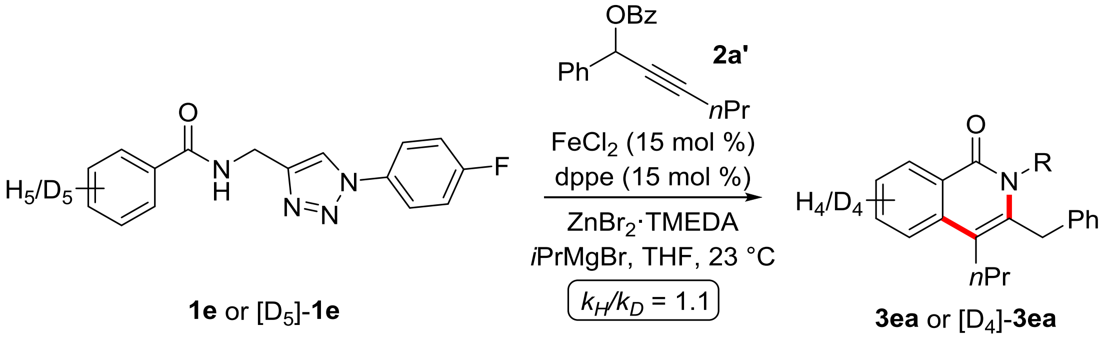

1e or [D$_5$]-**1e**   **3ea** or [D$_4$]-**3ea**

To a stirred solution of **1e** (296 mg, 1.00 mmol) or [D$_5$]-**1e** (301 mg, 1.00 mmol), $ZnBr_2$·TMEDA (683 mg, 2.00 mmol) and dppe (59.8 mg, 0.15 mmol, 15 mol %) in THF (1.3 mL), *i*PrMgBr (3.0 M in 2-MeTHF, 1.0 mL, 3.00 mmol) was added in one portion and the reaction mixture was stirred for 5 min at ambient temperature. Then, $FeCl_2$ (19.0 mg, 0.15 mmo, 15 mol %) was added in a single portion. After stirring for additional 5 min, a solution of propargyl benzoate **2a’** (557 mg, 2.0 mmol, 2.0 equiv) and 1-fluorononane (73.1 mg, 0.50 mmol, 0.5 equiv) in THF (1.3 mL) was added in one portion. The reaction mixture was stirred at ambient temperature. After the times indicated below an aliquot of 0.1 mL was taken, filtered over a short plug of silica and diluted with $CDCl_3$ (0.7 mL). The yields of product were determined *via* ^{19}F NMR using 1-fluorononane (73.1 mg, 0.5 mmol) as the internal standard.

**Time [min]**	**5**	**10**	**20**	**30**	**45**	**60**	**70**
**3ea [%]**	3.0	4.0	5.0	6.5	9.5	11.0	15.5
**[D$_4$]-3ea [%]**	2.5	4.0	6.0	7.0	9.5	12.0	14.0

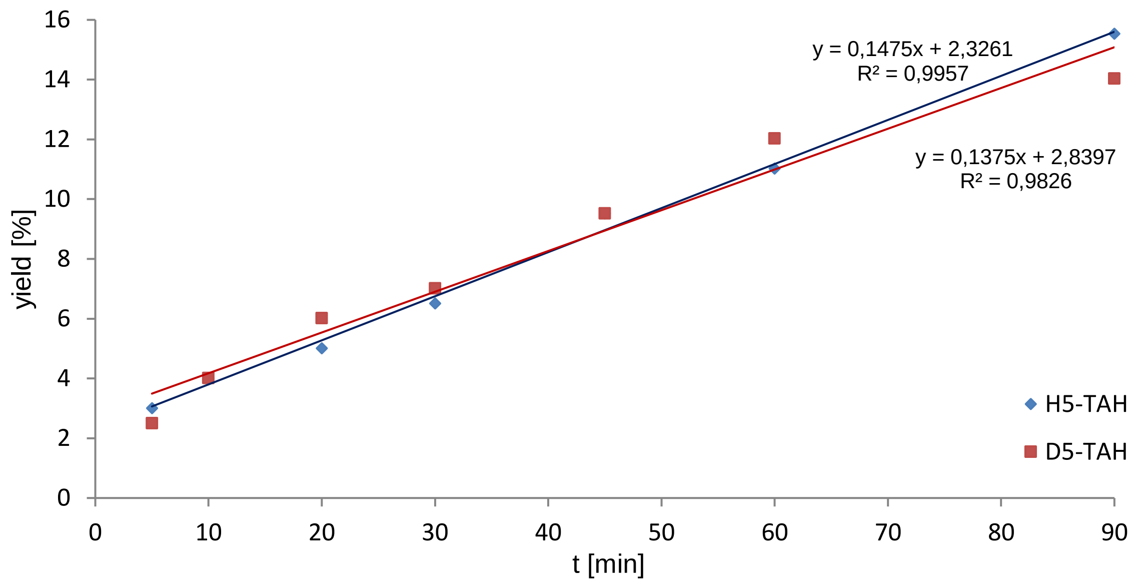

Figure S-2: Initial rates for the reaction of **1e** or $[D]_5$-**1e** with **2a'**.

Intermolecular KIE Measurement by One-Pot Reaction

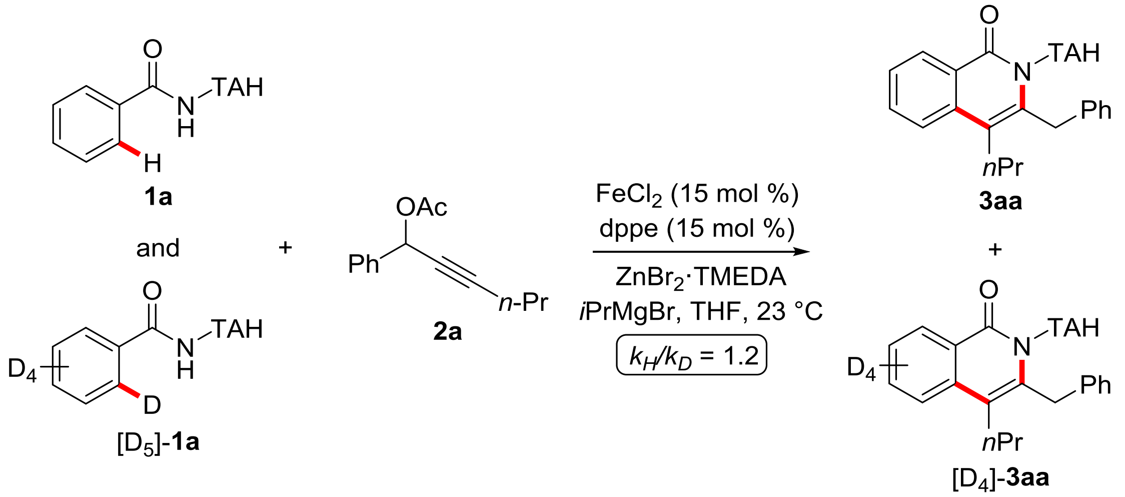

To a stirred solution of **1a** (43.0 mg, 0.15 mmol), $[D]_5$-**1a** (43.7 mg, 0.15 mmol) $ZnBr_2$·TMEDA (205 mg, 0.60 mmol) and dppe (17.9 mg, 15 mol %) in THF (0.40 mL), *i*PrMgBr (3.0 M in 2-MeTHF, 300 µL, 0.90 mmol) was added in one portion and the reaction mixture was stirred for 5 min at ambient temperature. Then, $FeCl_2$ (5.7 mg, 15 mol %) was added in a single portion. After stirring for additional 5 min, a solution of alkyne **2a** (130 mg, 0.60 mmol) in THF (0.40 mL) was added in one portion. After stirring 1 h at ambient temperature, sat. aqueous NH_4Cl (3.0 mL) was added and the reaction mixture was extracted with CH_2Cl_2 (3 × 15 mL). The combined organic extracts were dried over Na_2SO_4, filtered and concentrated under reduced pressure. Purification by column chromatography

(*n*hexane/EtOAc = 3/2) afforded a mixture of both products. The ratio of **3aa** to [D]$_4$-**3aa** was determined by ^{1}H NMR spectroscopic analysis.

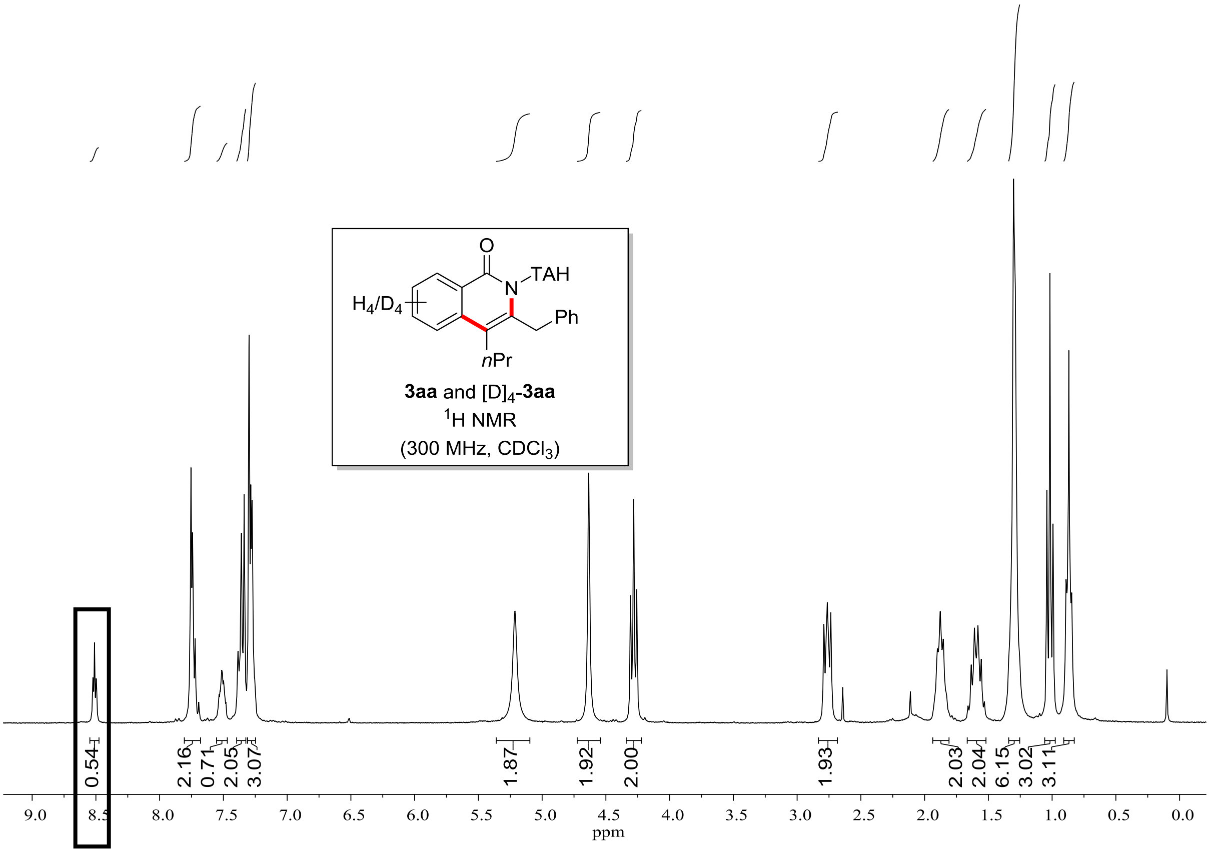

Reaction Using Deuterium-Labelled Substrate [D]$_5$-1a

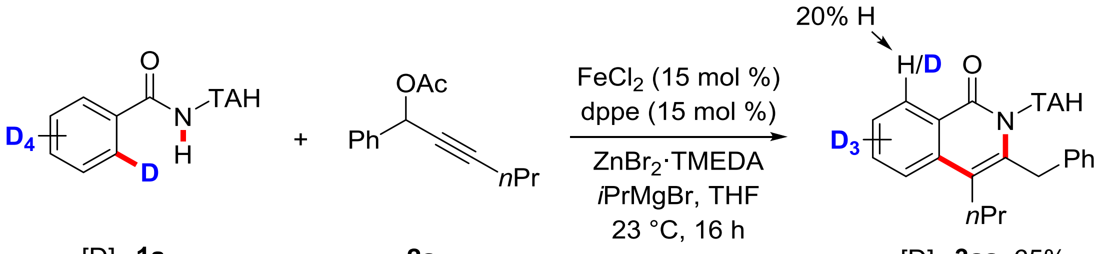

[D]$_5$-**1a** **2a** [D]$_n$-**3aa**: 65%

To a stirred solution of [D]$_5$-**1a** (87.4 mg, 0.3 mmol), $ZnBr_2$·TMEDA (205 mg, 0.60 mmol) and dppe (17.9 mg, 15 mol %) in THF (0.4 mL), *i*PrMgBr (3.0 M in 2-MeTHF, 300 μL, 0.90 mmol) was added in one portion and the reaction mixture was stirred for 5 min at ambient temperature. Then, $FeCl_2$ (5.7 mg, 15 mol %) was added in a single portion. After stirring for additional 5 min, a solution of alkyne **2a** (130 mg, 0.60 mmol) in THF (0.40 mL) was added in one portion. The reaction mixture was stirred at ambient temperature. After 16 h, sat. aqueous NH_4Cl (3.0 mL) was added and the reaction mixture was extracted with CH_2Cl_2 (3 × 15 mL). The combined organic extracts were dried over Na_2SO_4, filtered and concentrated. Purification

by column chromatography (*n*hexane/EtOAc = 3/2) yielded the annulated isoquinolone $[D]_n$-**3aa** (86.8 mg, 65%) as a white solid. The amount of deuterium incorporation was determined by ¹H NMR spectroscopy.

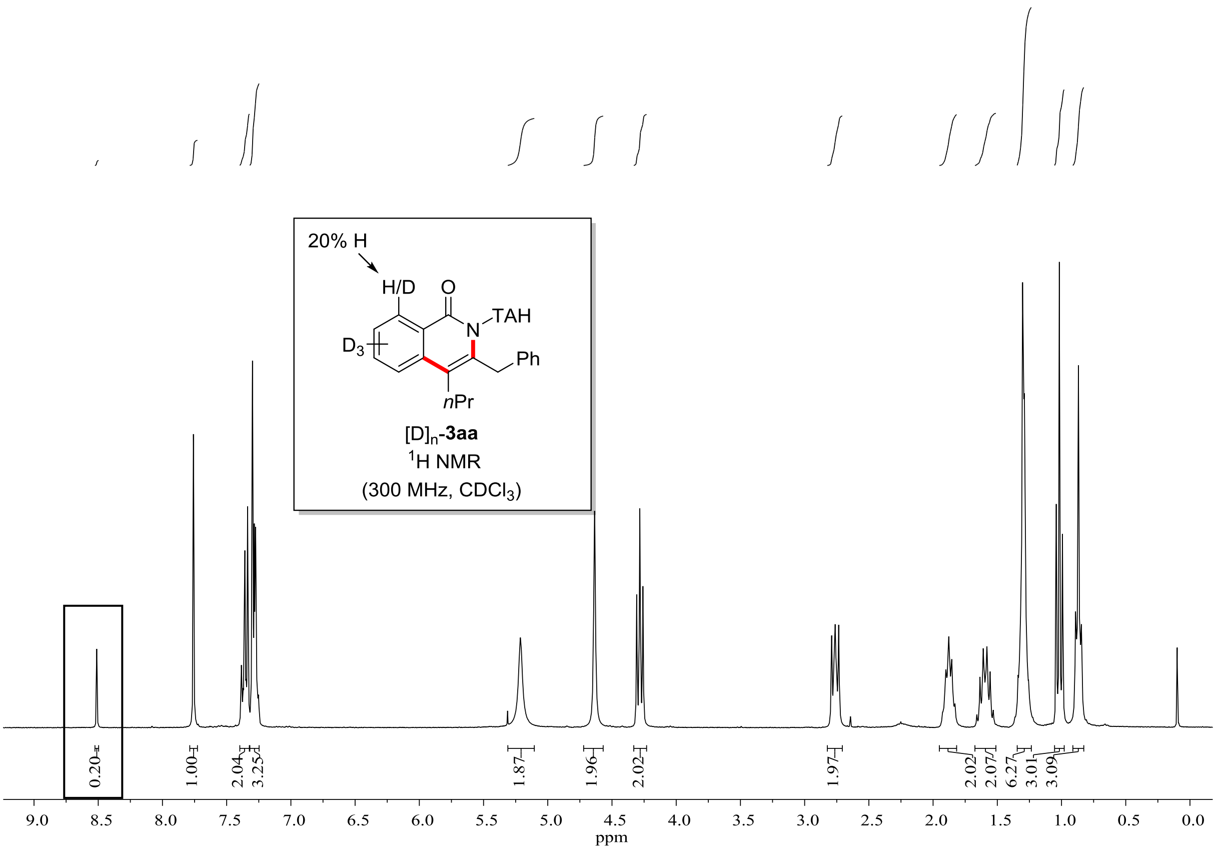

Deprotonation of Substrate 1a by PhZnCl

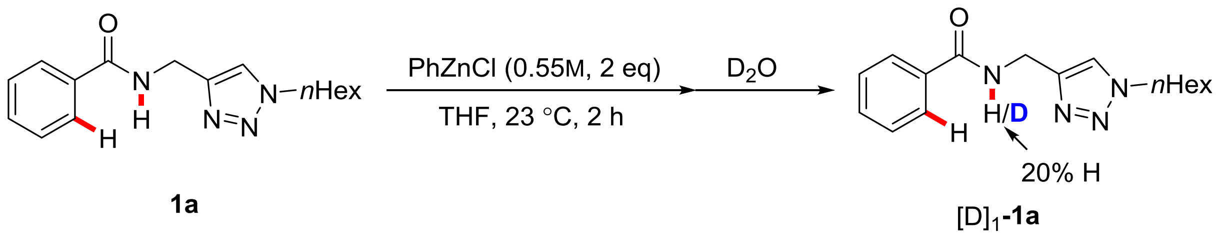

1a

$[D]_1$-**1a**

To a Schlenk tube charged with **1a** (85.9 mg, 0.30 mmol), PhZnCl (0.55 M in THF, 1.1 mL, 2.0 equiv) was added in one portion and the reaction mixture was stirred at ambient temperature. After 2 h, D_2O (2.0 mL) was added and the mixture was extracted with CH_2Cl_2 (3 × 15 mL). The combined organic extracts were dried over Na_2SO_4, filtered and concentrated under reduced pressure. Deuterium contents were determined by ¹H NMR spectroscopic analysis.

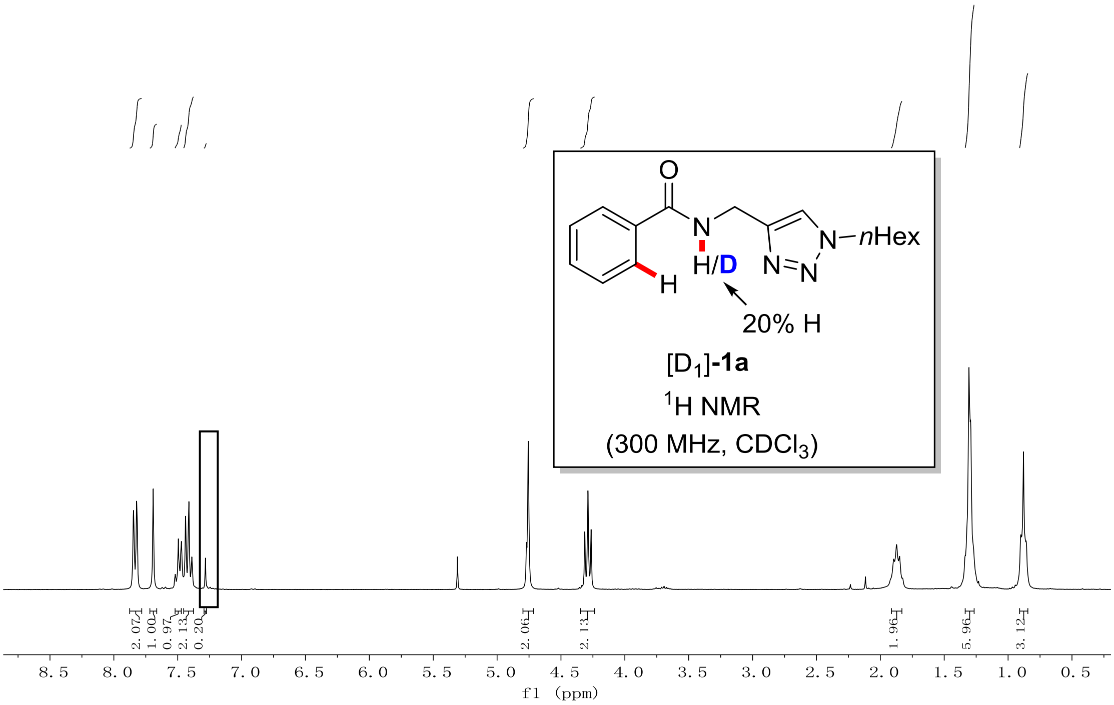

Reaction Using Propargyl Acetate 2k

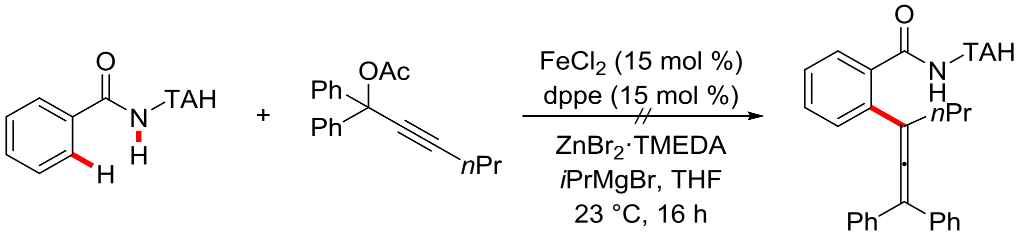

To a stirred solution of **1a** (85.9 mg, 0.30 mmol, 1.0 equiv), $ZnBr_2$·TMEDA (205 mg, 0.60 mmol, 2.0 equiv) and dppe (17.9 mg, 0.045 mmol, 15 mol %) in THF (0.40 mL), *i*PrMgBr (3.0 M in THF, 300 µL, 0.90 mmol, 3.0 equiv) was added in one portion and the reaction mixture was stirred for 5 min at ambient temperature. Then, $FeCl_2$ (5.7 mg, 0.045 mmol,15 mol %) was added in a single portion. After stirring the solution for additional 5 min, alkyne **2k** (175 mg, 0.60 mmol, 2.0 equiv) was added as a solution in THF (0.40 mL). Then, the mixture was stirred at ambient temperature. After stirring for 16 h, sat. aqueous NH_4Cl (3.0 mL) was added and the reaction mixture was extracted with CH_2Cl_2 (3 × 15 mL). The combined organic extracts were dried over Na_2SO_4, filtered and concentrated. Product **4** was not observed by ^{1}H NMR spectroscopic analysis.

Removal of TAH

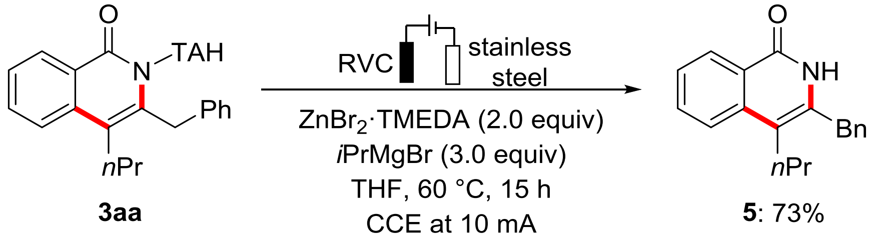

Figure S-3. Traceless removal of TAH group.

The electrochemical removal of the TAH group was carried out in an undivided cell with RVC anode (10 mm × 5 mm × 6 mm) and a steel cathode (20 mm × 10 mm × 0.25 mm). To a stirred solution of isoquinolone **3aa** (97.0 mg, 0.22 mmol) and $ZnBr_2$·TMEDA (150 mg, 0.44 mmol, 2.0 equiv) in THF (2.0 mL), *i*PrMgBr (3.0 M in 2-MeTHF, 220 µL, 0.66 mmol) was added in one portion. The electrocatalysis was performed at 60 °C with a constant current of 10.0 mA maintained for 15 h. Then, the mixture was allowed to cool to ambient temperature, and saturated aq. NH_4Cl (3.0 mL) was added. The RVC anode was washed with CH_2Cl_2 (3 × 10 mL) in an ultrasonic bath. The combined aqueous phase were extracted with CH_2Cl_2 (3 × 15 mL), dried over Na_2SO_4, filtered and concentrated under reduced pressure. Purification by column chromatography (*n*hexane/EtOAc = 3/2) yielded **5** (45.0 mg, 73%) as a white solid. **M.p.** = 194–196 °C. **^{1}H NMR** (300 MHz, $CDCl_3$): δ = 9.92 (s, 1H), 8.44 (d, *J* = 7.9 Hz, 1H), 7.72 (dd, *J* = 4.3, 2.0 Hz, 2H), 7.48 (ddd, *J* = 8.1, 5.4, 2.8 Hz, 1H), 7.38–7.18 (m, 5H), 4.08 (s, 2H), 2.81 (t, *J* = 8.1, 2H), 1.65–1.54 (m, 2H), 1.06 (t, *J* = 7.3 Hz, 3H). **^{13}C{H} NMR** (126 MHz, $CDCl_3$): δ = 163.2 (C_q), 138.3 (C_q), 137.1 (C_q), 135.5 (C_q), 132.6 (CH), 129.1 (CH), 128.9 (CH), 128.0 (CH), 127.3 (CH), 125.9 (CH), 125.6 (C_q), 123.3 (CH), 114.2 (C_q), 36.8 (CH_2), 29.0 (CH_2), 23.5 (CH_2), 14.4 (CH_3). **IR** (ATR): 3022, 2954, 2871, 2031, 1653, 1630, 1606, 758, 709, 511 cm^{-1}. **MS** (ESI) *m/z* (relative intensity): 168 (100), 278 (67) $[M+H]^+$, 577 (25) $[2M+Na]^+$. **HR-MS** (ESI) *m/z* calcd for $C_{19}H_{19}NO$ $[M+H]^+$ 278.1539, found 278.1538.

Mössbauer Spectroscopy – Analytical Data

Subspectra were assigend based on the following considerations: i) isomers shifts higher than 0.9 mm/s are characteristic of Fe(II)-HS species, for which the coordination number is likely five or higher; ii) usually, high-valent species such as Fe(IV) display very low or even negative isomer shifts, which is not the case here; iii) the quadrupole splitting of Fe(III)-LS species is expected to be high due to the valence contribution to the electrical field gradient, while the isomer shift should be quite small; such subspectra are not observed in any of the present experiments; iv) six-coordinate Fe(II)-LS and Fe(III)-HS reveal similar isomer shift and quadrupole splitting parameters, which does not allow to distinguish between these two cases based on Mössbauer spectroscopy only; v) trends of isomer shifts and quadrupole splittings for low-valent and/or low-coordinate iron compounds are mostly elusive due to the strong degree of π-backdonation; therefore, the presence of such species cannot be ruled out completely.[7]

Table S-3. Mössbauer Parameters of Reaction Mixtures

Entry	Reaction	Valence of Iron/ Spin State	δ (mm s^{-1})	ΔE_Q (mm s^{-1})	rel. int.(%)
1	$^{57}FeCl_2$ + THF	$+2^{HS}$	1.26	3.05	100
2	Entry 1 + MeMgBr	+1.4[7]	0.29	0.88	100
3	Entry 2+	$+2^{HS}$	1.01	2.69	69
	$ZnBr_2$·TMEDA	$+2^{HS}$	1.36	2.56	31
		$+2^{HS}$	0.92	1.42	23
4	Entry 3 + dppe	$+2^{HS}$	0.98	2.57	40
		$+2^{HS}$	1.24	2.68	37
		n.a.[a]	0.26	1.01	43
5	Entry 4 + **1a**	$+2^{HS}$	1.14	2.45	36
		$+2^{HS}$	1.00	3.17	21
6	Entry 5 + **2a**	$+2^{HS}$	1.00	2.94	48
		$+2^{HS}$	0.95	2.29	52

[a] n.a. = not assigned since the parameters are not specific.

Sample Preparation for Mössbauer Measurements

Entry 1. $^{57}FeCl_2$+ THF

Inside a nitrogen-filled glovebox, a suspension of $^{57}FeCl_2$ (3.2 mg, 25 μmol) in THF (0.80 mL) was stirred at ambient temperature for 5 min. Then, the solution was filtered and added into a sample holder. The sample holder was taken out of the glovebox and frozen in liquid nitrogen immediately.

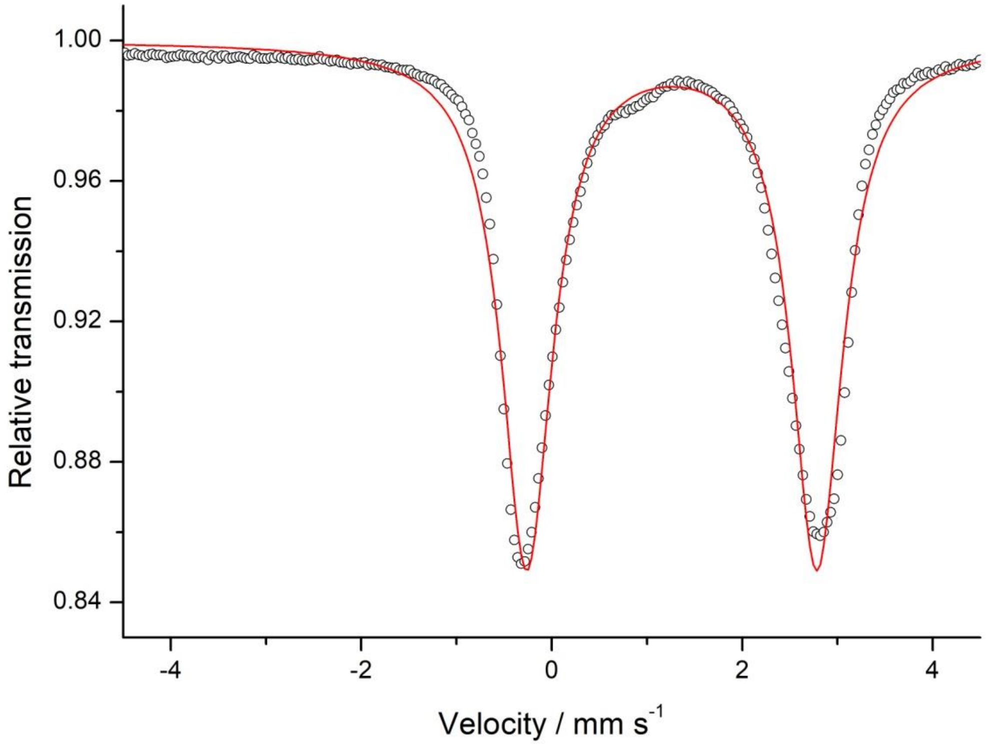

Figure S-4. Mössbauer spectrum of a frozen solution (T = 80 K) of $^{57}FeCl_2$ (31 mM) in THF together with fit (red): δ= 1.26 mm s^{-1}, ΔE_Q = 3.05 mm s^{-1}.

Entry 2. $^{57}FeCl_2$+MeMgBr + THF

Inside a nitrogen-filled glovebox, a suspension of MeMgBr (3.0 M in Et_2O, 75 μL, 9.0 equiv) in THF (17 μL) was stirred at ambient temperature for 5 min. Then, $^{57}FeCl_2$ (3.2 mg, 25 μmol) was added in a single portion. After stirring for additional 5 min, the solution was diluted to 5.0 mL by adding THF, 0.80 mL of the solution was filtered and added into a sample holder. The sample holder was taken out of the glovebox and frozen in liquid nitrogen immediately.

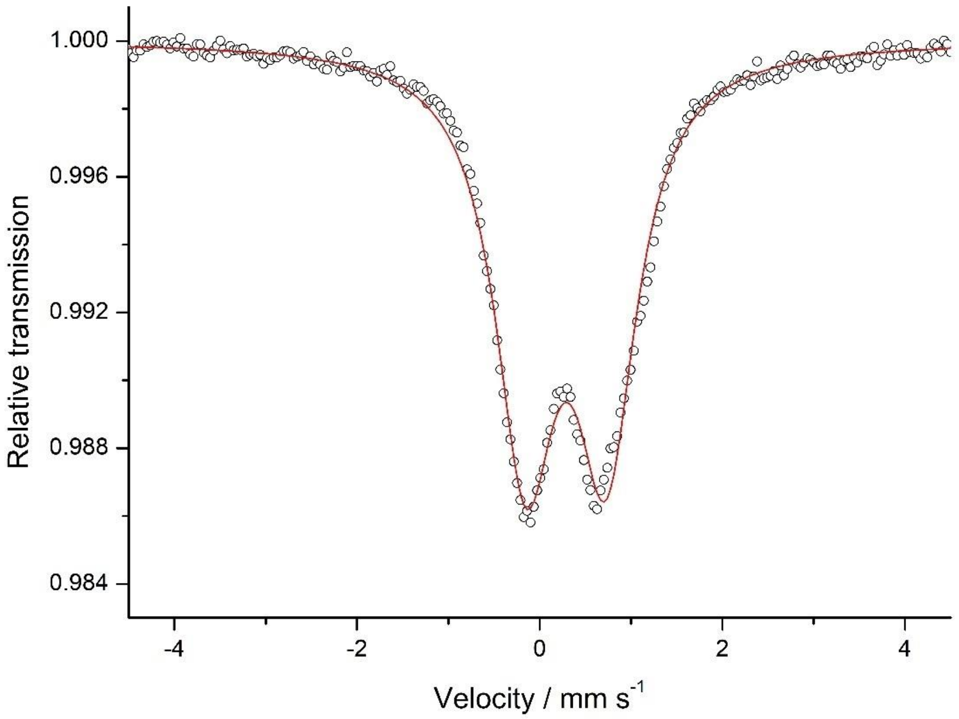

Figure S-5. Mössbauer spectrum of a frozen solution (T = 80 K) of the products formed in the reaction of $^{57}FeCl_2$ (5.0 mM) with MeMgBr (9.0 equiv) in THF together with the best fit (red): δ = 0.29 mm s^{-1}, ΔE_Q = 0.88 mm s^{-1}.

Entry 3. $^{57}FeCl_2$ +MeMgBr + $ZnBr_2$·TMEDA + THF

Inside a nitrogen-filled glovebox, to a stirred solution of $ZnBr_2$·TMEDA (51.6 mg, 6.0 equiv) in THF (17 µL), MeMgBr (3.0 M in Et_2O, 75 µL, 9.0 equiv) was added in one portion and the reaction mixture was stirred for 5 min at ambient temperature. Then, $^{57}FeCl_2$ (3.2 mg, 25 µmol) was added in a single portion. After stirring for additional 5 min, the solution was diluted to 5.0 mL by adding THF, 0.80 mL of the solution was filtered and added into a sample holder. The sample holder was taken out of the glovebox and frozen in liquid nitrogen immediately.

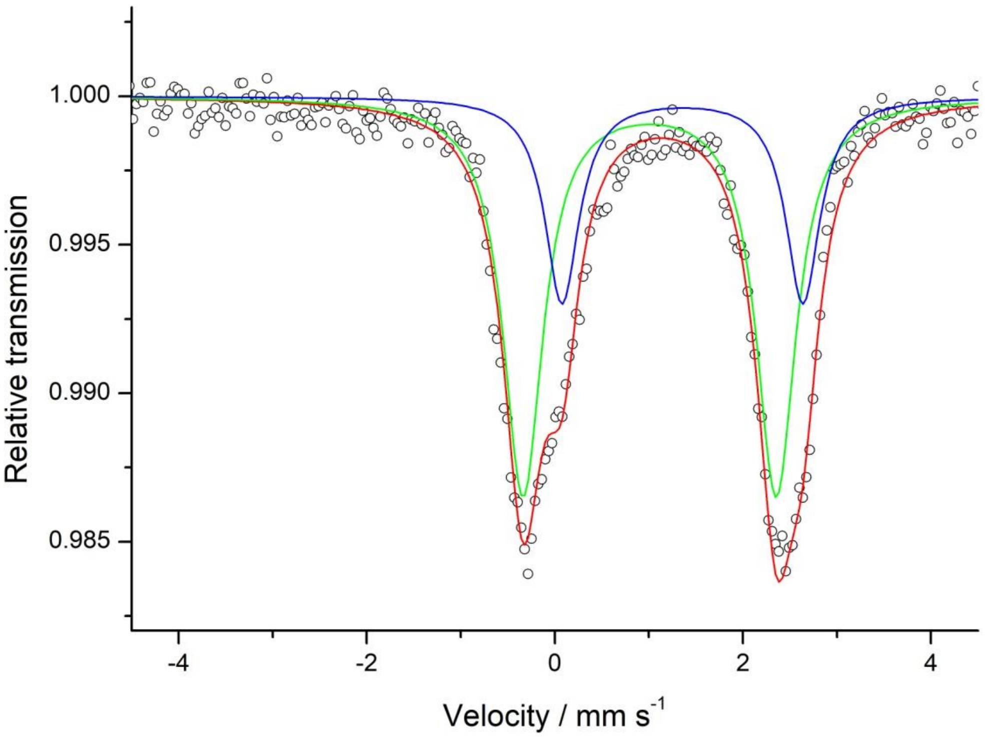

Figure S-6. Mössbauer spectrum of a frozen solution (T = 80 K) of the products formed in the reaction of $^{57}FeCl_2$ (5.0 mM) with MeMgBr (9.0 equiv) and $ZnBr_2$·TMEDA (6.0 equiv) in THF together with overall fit (red); subspectra of the fit: δ(green) = 1.01 mm s^{-1}, ΔE_Q(green) = 2.69 mm s^{-1}, rel. int. (green) = 69%; δ(blue) = 1.36 mm s^{-1}, ΔE_Q(blue) = 2.56 mm s^{-1}, rel. int. (blue) = 31%.

Entry 4. $^{57}FeCl_2$+MeMgBr + $ZnBr_2$·TMEDA + dppe + THF

Inside a nitrogen-filled glovebox, to a stirred solution of $ZnBr_2$·TMEDA (51.6 mg, 6.0 equiv) and dppe (10.0 mg, 1.0 equiv) in THF (17 µL), MeMgBr (3.0 M in Et_2O, 75 µL, 9.0 equiv) was added in one portion and the reaction mixture was stirred for 5 min at ambient temperature. Then, $^{57}FeCl_2$ (3.2 mg, 25 µmol) was added in a single portion. After stirring for additional 5 min, the solution was diluted to 5.0 mL by adding THF, 0.80 mL of the solution was filtered and added into a sample holder. The sample holder was taken out of the glovebox and frozen in liquid nitrogen immediately.

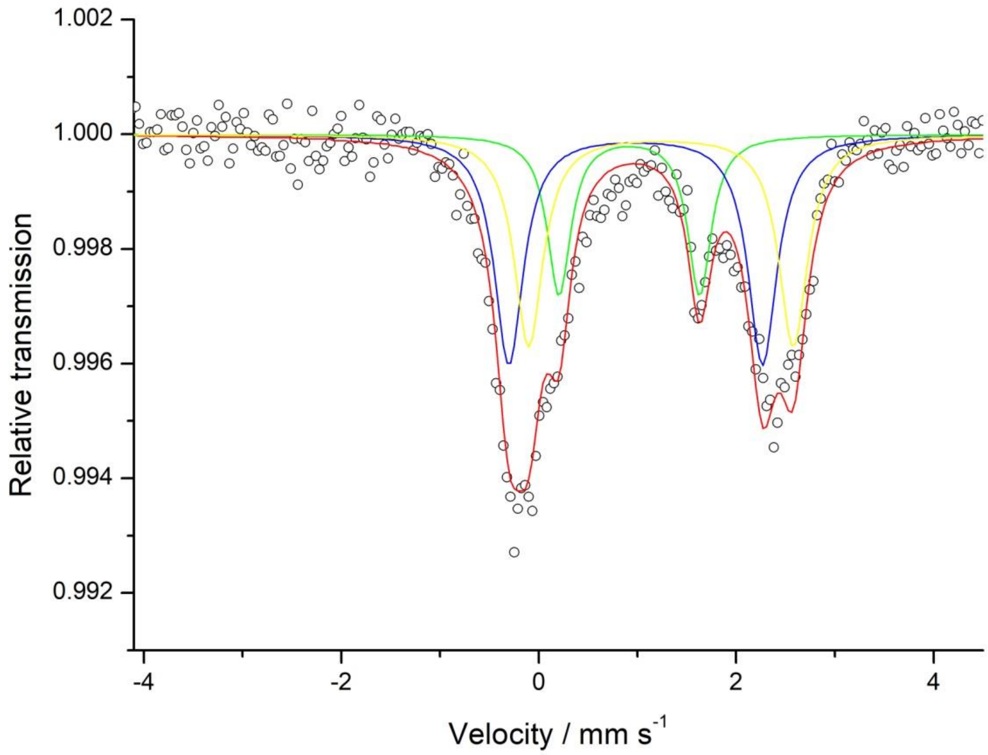

Figure S-7. Mössbauer spectrum of a frozen solution (T = 80 K) of the products formed in the reaction of $^{57}FeCl_2$ (5.0 mM) with MeMgBr (9.0 equiv), $ZnBr_2$·TMEDA (6.0 equiv) and dppe (1.0 equiv) in THF together with overall fit (red); subspectra of the fit: δ(green) = 0.92 mm s^{-1}, ΔE_Q(green) = 1.42 mm s^{-1}, rel. int. (green) = 23%; δ(blue) = 0.98 mm s^{-1}, ΔE_Q(blue) = 2.57 mm s^{-1}, rel. int. (blue) = 40%; δ(yellow) = 1.24 mm s^{-1}, ΔE_Q(yellow) = 2.68mm s^{-1}, rel. int. (yellow) = 37%.

Entry 5. $^{57}FeCl_2$ +MeMgBr + $ZnBr_2$ TMEDA + dppe + TAH-substrate (1a) + THF

Inside a nitrogen-filled glovebox, to a stirred solution of **1a** (7.2 mg, 1.0 equiv), $ZnBr_2$·TMEDA (51.6 mg, 6.0 equiv) and dppe (10.0 mg, 1.0 equiv) in THF (17 µL), MeMgBr (3.0 M in Et_2O, 75 µL, 9.0 equiv) was added in one portion and the reaction mixture was stirred for 5 min at ambient temperature. Then, $^{57}FeCl_2$ (3.2 mg, 25 µmol) was added in a single portion. After stirring for additional 5 min, the solution was diluted to 5.0 mL by adding THF, 0.80 mL of the solution was filtered and added into a sample holder. The sample holder was taken out of the glovebox and frozen in liquid nitrogen immediately.

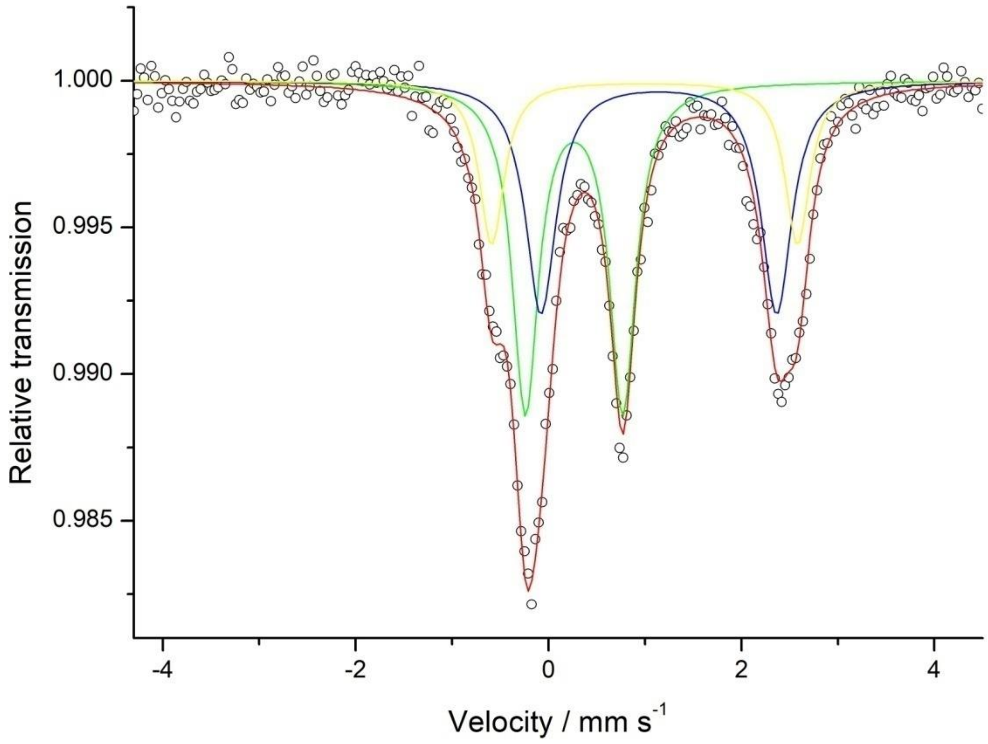

Figure S-8. Mössbauer spectrum of a frozen solution (T = 80 K) of the products formed in the reaction of $^{57}FeCl_2$ (5.0 mM) with MeMgBr (9.0 equiv), $ZnBr_2$·TMEDA (6.0equiv), dppe (1.0 equiv) and **1a** (1.0 equiv) in THF together with overall fit (red); subspectra of the fit: δ(green) = 0.26 mm s^{-1}, ΔE_Q(green) = 1.01 mm s^{-1}, rel. int. (green) = 43%; δ(blue) = 1.14 mm s^{-1}, ΔE_Q(blue) = 2.45 mm s^{-1}, rel. int. (blue) = 36%; δ(yellow) = 1.00 mm s^{-1}, ΔE_Q(yellow) = 3.17mm s^{-1}, rel. int. (yellow) = 21%.

Entry 6. $^{57}FeCl_2$ +MeMgBr + $ZnBr_2$·TMEDA + dppe + TAH-substrate (1a) + alkyne (2a)+ THF

Inside a nitrogen-filled glovebox, to a stirred solution of **1a** (7.2 mg, 1.0 equiv), $ZnBr_2$·TMEDA (51.6 mg, 6.0 equiv) and dppe (10.0mg, 1.0 equiv) in THF (17 µL), MeMgBr (3.0 M in Et_2O, 75 µL, 9.0 equiv) was added in one portion and the reaction mixture was stirred for 5 min at ambient temperature. Then, $^{57}FeCl_2$ (3.2 mg, 25 µmol) was added in a single portion. After stirring for additional 5 min, alkyne **2a** (16.0 mg, 3.0 equiv) was added as a solution in THF (17 µL). After stirring for additional 5 min, the solution was diluted to 5.0 mL by adding THF, 0.80 mL of the solution was filtered and added into a sample holder. The sample holder was taken out of the glovebox and frozen in liquid nitrogen immediately.

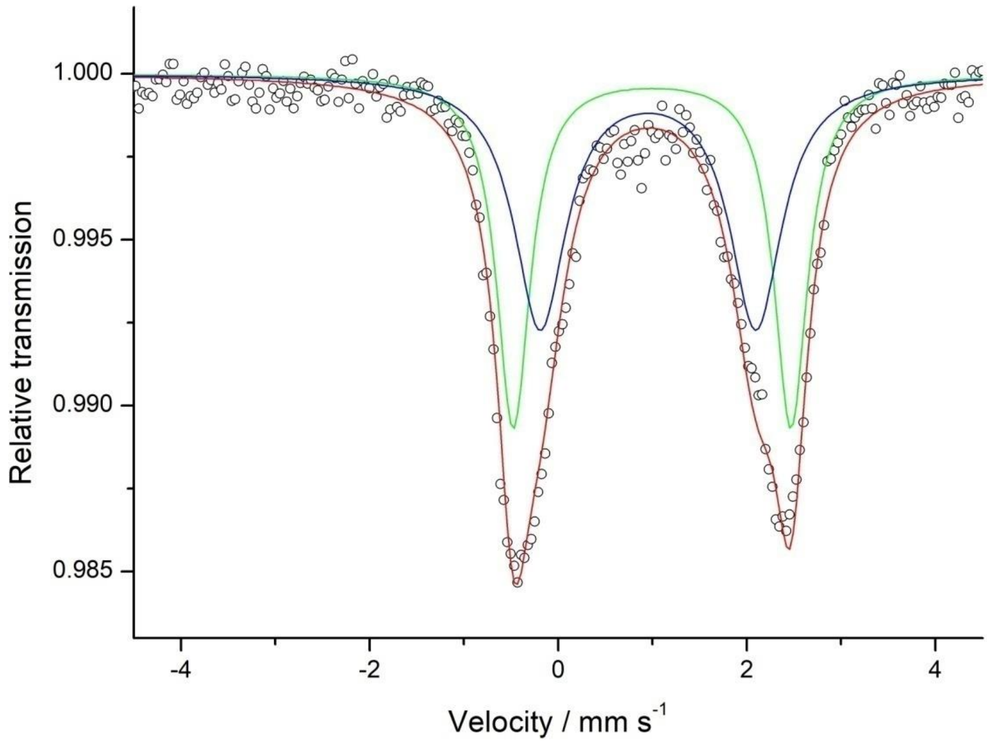

Figure S-9. Mössbauer spectrum of a frozen solution (T = 80 K) of the products formed in the reaction of $^{57}FeCl_2$ (5.0 mM) with MeMgBr (9.0 equiv), $ZnBr_2$·TMEDA (6.0 equiv), dppe (1.0 equiv), **1a** (1.0 equiv) and **2a** (3.0 equiv) in THF together with overallfit (red); subspectra of the fit: δ(green) = 1.00 mm s^{-1}, ΔE_Q(green) = 2.94 mm s^{-1}, rel. int. (green) = 48%; δ(blue) = 0.95 mm s^{-1}, ΔE_Q(blue) = 2.29 mm s^{-1}, rel. int. (blue) = 52%.

Computational Studies

All structures were optimized at the TPSS[9] level of theory in combination with D3 dispersion corrections with Becke-Johnson damping scheme (D3BJ).[10] Analytical frequency calculations were carried out at the same level of theory in order to identify all stationary points as either intermediates (no imaginary frequencies) or transition states (only one imaginary frequency), and to provide thermal and non-thermal corrections to the Gibbs free energy in gas-phase at 298.15 K. For the geometry optimizations all atoms were described with a def2-SVP basis set.[11] The electronic energy was then refined through PW6B95[12] single-point calculations on the optimized geometries in combination with dispersion corrections (D3BJ) with a def2-TZVP basis set.[11] Solvent effects were included implicitly through the use of the SMD model[13] with a dielectric constant of ε = 7.4257, which corresponds to THF.

Energies reported are based on gas-phase Gibbs free energies with def2-SVP basis set for which the electronic energies were corrected to PW6B95-D3BJ with def2-TZVP basis set and solvent effects. All calculation were performed using Gaussian 16, Revision A.03 package.[14]

3D structures images were created with *PyMOL* version 1.8.x.[15]

Table S-4. Calculated electronic energies at the PW6B95-D3BJ/def2-TZVP+SMD(THF) level of theory and Gibbs free energies with and without dispersion corrections for all structures in the present work (all in Hartree).[a]

Structure	Electronic Energy	Total Gibbs Free Energy	Total Gibbs Free Energy Without Dispersion
^{1}I-0	-2850.088390	-2849.309126	-2849.188123
^{1}TS(0-1)	-2850.077471	-2849.302210	-2849.180850
^{1}TS(0-1')	-2849.970241	-2849.203010	-2849.080815
^{1}I-1'	-2731.961818	-2731.266389	-2731.159267
^{1}TS(1'-1)	-2731.952976	-2731.260628	-2731.153066
^{1}I-1	-2963.634237	-2962.845725	-2962.722775
^{1}I-2	-3425.485098	-3424.558692	-3424.406123
^{1}TS(2-3)	-3425.479860	-3424.554893	-3424.403860
^{1}I-3	-3425.514095	-3424.587414	-3424.434835
^{1}TS(3-4)	-3425.494324	-3424.573768	-3424.422864
^{1}I-4	-3425.538529	-3424.612552	-3424.460449
^{1}TS(4-5)	-3425.522305	-3424.597802	-3424.446228
^{1}I-5	-3425.555761	-3424.631450	-3424.481946
^{3}I-0	-2850.098688	-2849.329527	-2849.208579
^{3}TS(0-1)	-2850.052698	-2849.285301	-2849.166988
^{3}TS(0-1')	-2850.041800	-2849.282840	-2849.164569
^{3}I-1'	-2731.968307	-2731.279531	-2731.174056
^{3}TS(1'-1)	-2731.918009	-2731.232909	-2731.127058
^{3}I-1	-2963.632014	-2962.849566	-2962.726490
^{3}I-2	-3425.472619	-3424.553713	-3424.405485
^{3}TS(2-3)	-3425.463192	-3424.543623	-3424.394893
^{3}I-3	-3425.528882	-3424.608351	-3424.454683
^{3}TS(3-4)	-3425.511762	-3424.588831	-3424.428863
^{3}I-4	-3425.547076	-3424.626115	-3424.473485
^{3}TS(4-5)	-3425.516554	-3424.599529	-3424.450846
^{3}I-5	-3425.579822	-3424.658255	-3424.508685
^{5}I-0	-2850.107653	-2849.344370	-2849.227811
^{5}TS(0-1)	-2850.070714	-2849.308641	-2849.192014
^{5}TS(0-1')	-2850.057460	-2849.301310	-2849.184709
^{5}I-1'	-2731.973966	-2731.291224	-2731.186809
^{5}TS(1'-1)	-2731.931748	-2731.249444	-2731.143246
^{5}I-1	-2963.636350	-2962.858093	-2962.737673
^{5}I-2	-3425.475077	-3424.561028	-3424.416059
^{5}TS(2-3)	-3425.467004	-3424.556110	-3424.412991
^{5}I-3	-3425.542376	-3424.627258	-3424.474451
^{5}TS(3-4)	-3425.527076	-3424.614203	-3424.463090
^{5}I-4	-3425.572261	-3424.656333	-3424.506469
^{5}TS(4-5)	-3425.546513	-3424.630546	-3424.480512
^{5}I-5	-3425.601713	-3424.684699	-3424.536061
Propane	-119.335433	-119.259037	-119.256545
Propene	-118.102606	-118.100557	-118.047138
H_2	-1.174126	-1.175611	-1.175582
THF	-232.830517	-232.743889	-232.739854
Alkyne	-694.691637	-694.479541	-694.462719

[a]Superscripts correspond to the spin-state of the intermediates and transition states.

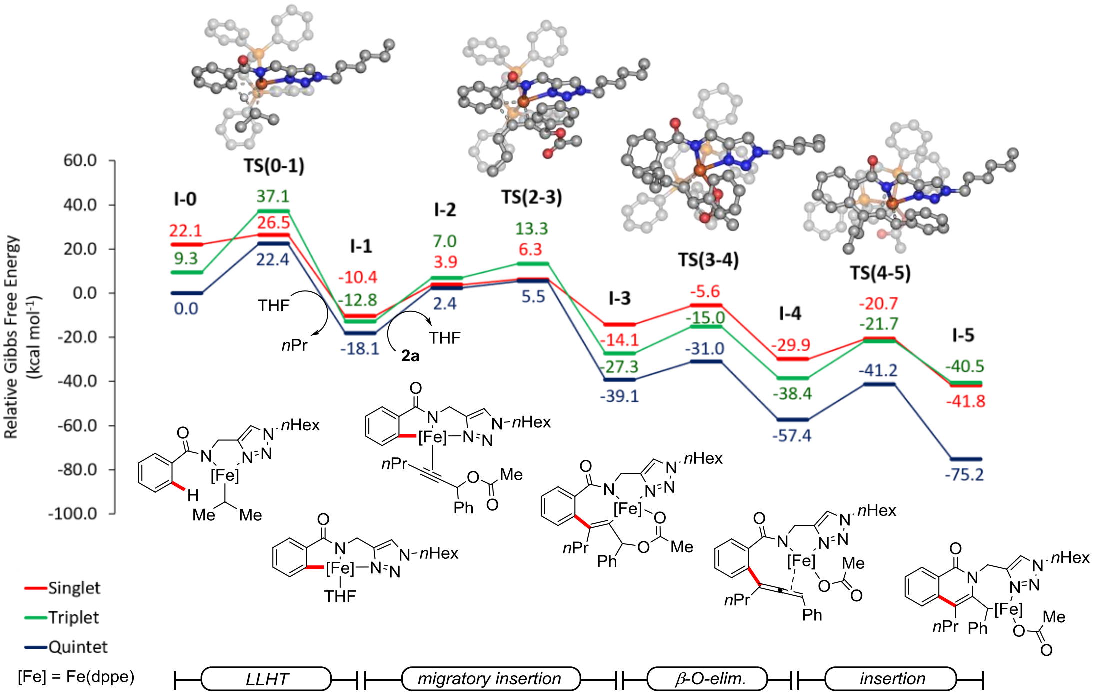

Figure S-10. Computed Gibbs free energies ($\Delta G_{298.15}$) in kcal mol^{-1} for the reaction profile for the iron-catalyzed C–H/N–H annulation of alkyne. All energy values include dispersion corrections. In the computed transition state structures non-participating H atoms were omitted for clarity.

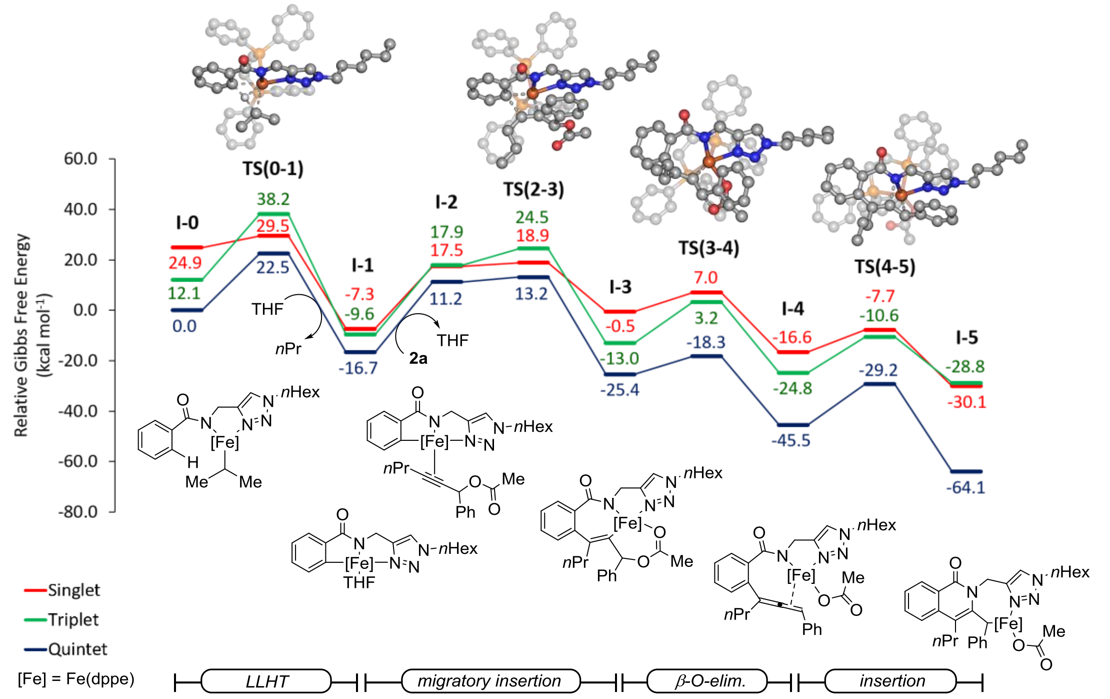

Figure S-11. Computed Gibbs free energies ($\Delta G_{298.15}$) in kcal mol^{-1} for the reaction profile for the iron-catalyzed C–H/N–H alkyne annulation of alkyne in the absence of dispersion corrections. In the computed transition state structures non-participating H atoms were omitted for clarity.

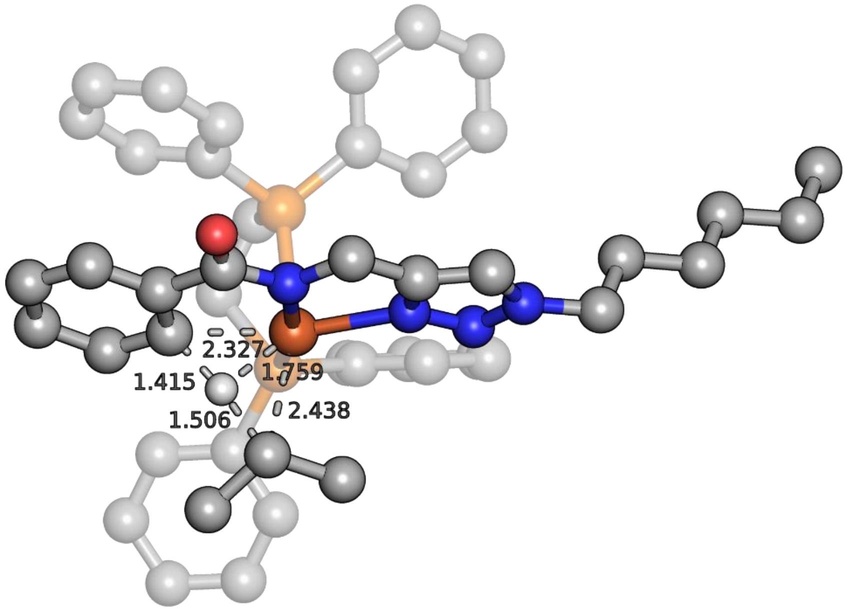

TS(0-1)

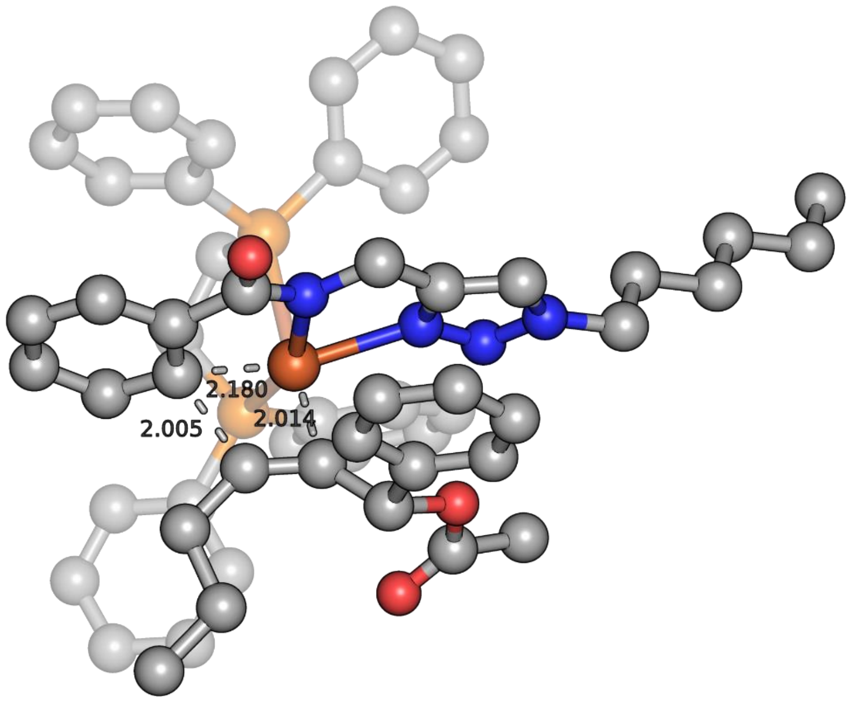

TS(2-3)

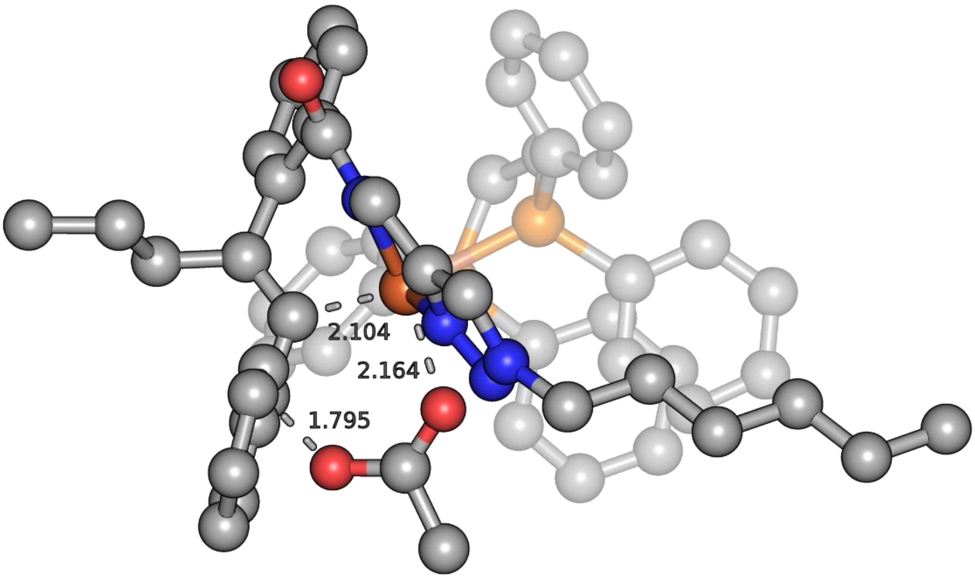

TS(3-4)

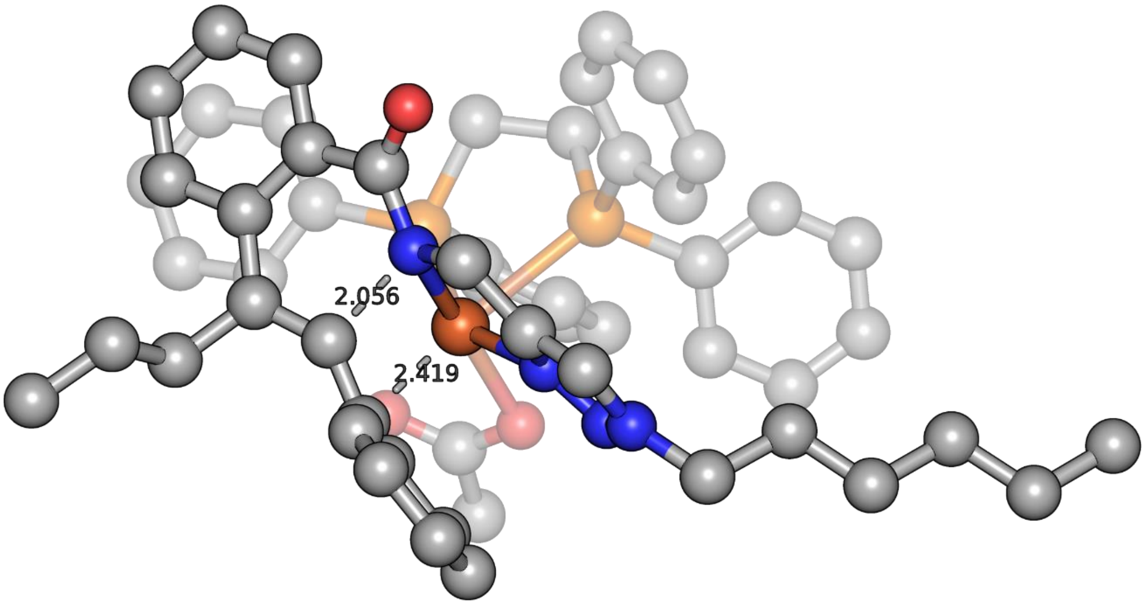

TS(4-5)

Figure S-12. Computed transition state structures for the high spin pathway. Key distances are provided in Å and non-participating H atoms were omitted for clarity.

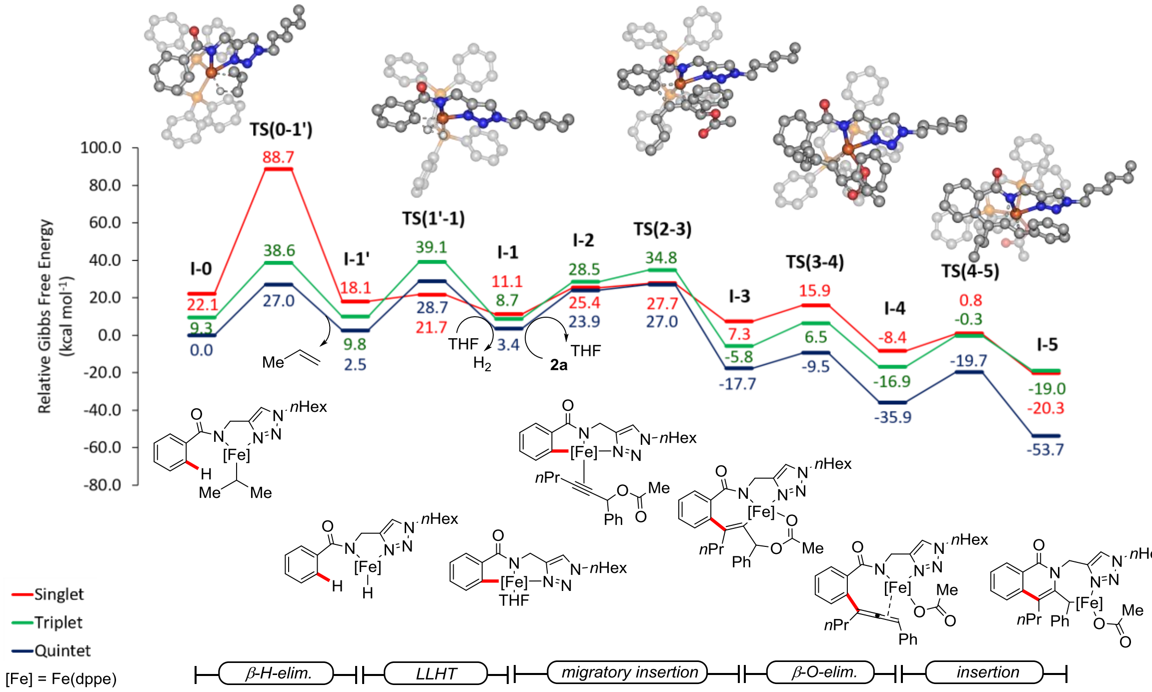

Figure S-13. Computed Gibbs free energies ($\Delta G_{298.15}$) in kcal mol^{-1} for the reaction profile corresponding to the β-H-elimination pathway for the iron-catalyzed C–H/N–H annulation of alkyne. All energy values include dispersion corrections. In the computed transition state structures non-participating H atoms were omitted for clarity.

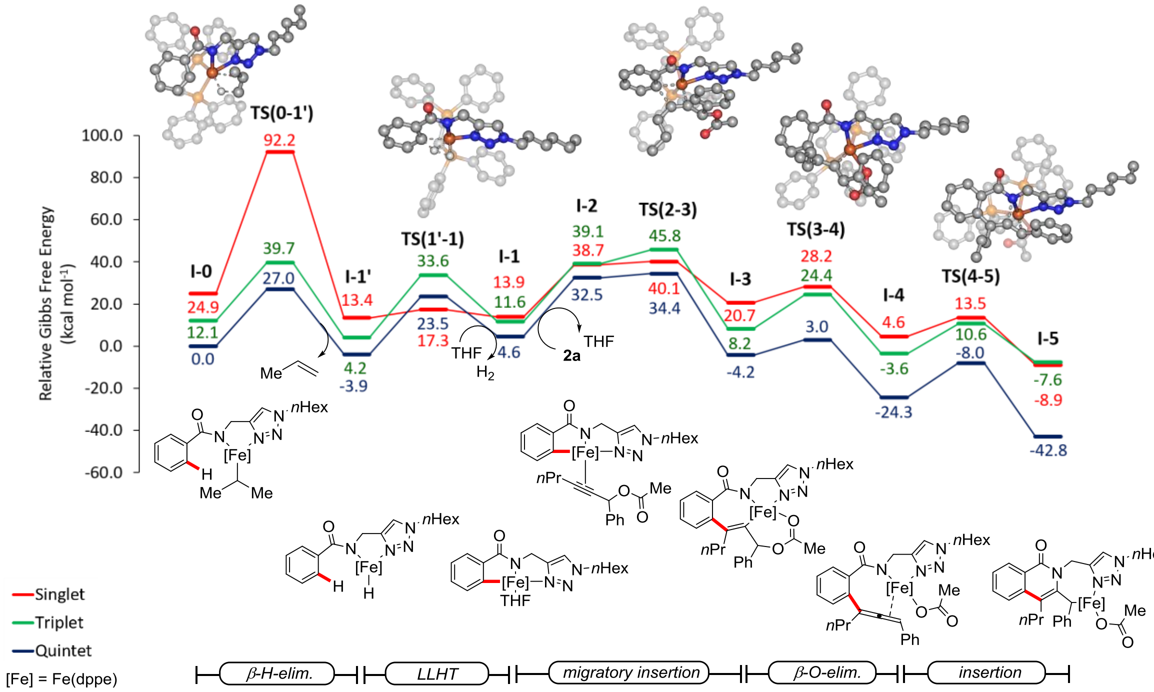

Figure S-14. Computed Gibbs free energies ($\Delta G_{298.15}$) in kcal mol^{-1} for the reaction profile corresponding to the β-H-elimination pathway for the iron-catalyzed C–H/N–H annulation of alkyne in the absence of dispersion corrections. In the computed transition state structures non-participating H atoms were omitted for clarity.

Cartesian coordinates of the optimized structures

[1]I-0
Lowest frequency = 14.1082 cm^{-1}
Charge = 0, Multiplicity = 1

105

P	1.127793	-1.125603	-1.026499
P	0.138355	1.752720	-0.640232
C	0.708297	-0.208307	-2.616900
H	1.390227	-0.507959	-3.430102
H	-0.294360	-0.563176	-2.905592
C	0.719998	1.303561	-2.382547
H	0.127779	1.848574	-3.139126
H	1.752834	1.688201	-2.429284
C	3.185006	0.698334	0.391508
C	3.914722	-0.335877	1.034894
C	3.839354	1.513875	-0.552302
C	5.264091	-0.539211	0.720532
C	5.182375	1.285351	-0.879973
H	3.306768	2.352338	-1.012433
C	5.899743	0.265432	-0.233248
H	5.781180	-1.357935	1.231277
H	5.675521	1.919253	-1.625206
H	6.952764	0.094396	-0.482154
C	3.202155	-1.297985	1.939204
N	1.863469	-1.138769	1.836059
C	1.010017	-2.077716	2.543821
H	1.140152	-3.116143	2.163521
H	1.255977	-2.126604	3.623878
C	-0.393420	-1.601107	2.322856
C	-1.647194	-1.915895	2.817840
N	-0.575072	-0.534698	1.466832
N	-1.838754	-0.178298	1.418046
N	-2.496276	-1.013739	2.236814
H	-1.991315	-2.669763	3.522731
O	3.780812	-2.175994	2.611259
Fe	1.020635	0.275167	0.767205
C	2.732609	-1.953570	-1.341851
C	3.133844	-2.994122	-0.475550
C	3.635051	-1.507973	-2.325862
C	4.392916	-3.588778	-0.607704
H	2.467567	-3.330258	0.322719
C	4.893844	-2.108421	-2.460574
H	3.369710	-0.676566	-2.985669
C	5.275532	-3.150890	-1.605014
H	4.688746	-4.376339	0.091942
H	5.584904	-1.746064	-3.229002
H	6.265063	-3.609447	-1.704888
C	-0.083113	-2.525939	-1.161643
C	0.263576	-3.855021	-1.474422
C	-1.449892	-2.206397	-1.005176
C	-0.726439	-4.839616	-1.609351
H	1.311361	-4.124697	-1.630097
C	-2.437342	-3.187133	-1.159184
H	-1.743980	-1.180240	-0.766007
C	-2.079726	-4.511476	-1.452824
H	-0.434087	-5.866923	-1.852886
H	-3.491543	-2.912813	-1.051396
H	-2.851632	-5.280120	-1.567381
C	-3.943205	-0.841515	2.384749
H	-4.263370	-1.487804	3.217734
H	-4.114361	0.208441	2.677435
C	-4.691749	-1.161873	1.087085
C	-6.162422	-0.733646	1.127568
H	-4.610773	-2.244714	0.880786
H	-4.180443	-0.631020	0.265161
C	-6.902666	-1.050486	-0.178889
H	-6.681821	-1.223494	1.975238
H	-6.213342	0.356055	1.320139
C	-8.354493	-0.552973	-0.199869
H	-6.348214	-0.597798	-1.024329
H	-6.885229	-2.144632	-0.354392
H	-8.905929	-1.002249	0.649115
H	-8.363187	0.540730	-0.023666
C	-9.082258	-0.867756	-1.511680
H	-9.117701	-1.957034	-1.695960
H	-8.568932	-0.401795	-2.372760
C	-1.697685	1.896976	-0.856848
C	-2.408363	1.138509	-1.806589
C	-2.426796	2.749704	-0.001984
C	-3.808361	1.205573	-1.881869
H	-1.876892	0.486980	-2.504111
C	-3.822376	2.819358	-0.076605
H	-1.893748	3.367791	0.725074
C	-4.522171	2.040598	-1.012094
H	-4.338241	0.606138	-2.630346
H	-4.367060	3.491084	0.596002
H	-5.614590	2.095142	-1.070986
C	0.589000	3.552257	-0.685263
C	1.607282	4.041092	0.150944
C	-0.030559	4.436349	-1.593141
C	2.007836	5.383482	0.082332
H	2.080409	3.365211	0.867661
C	0.367021	5.777570	-1.661929
H	-0.836497	4.074402	-2.241268
C	1.388446	6.254073	-0.824470
H	2.800902	5.746894	0.744344
H	-0.123412	6.454918	-2.369643
H	1.696400	7.303878	-0.877335
H	-10.121170	-0.494717	-1.496974
C	0.914605	1.368200	2.600225
C	-0.200914	2.405154	2.694782
C	2.230611	1.905480	3.174171
H	0.633870	0.521445	3.252947
H	-1.166461	1.990451	2.357830
H	-0.337590	2.754491	3.742557
H	0.012780	3.305374	2.089588
H	3.023053	1.138048	3.182653
H	2.622855	2.780252	2.619329
H	2.089212	2.242803	4.223250
H	2.336340	1.238935	0.966428

[1]TS(0-1)
Lowest frequency = -1244.4327 cm^{-1}
Charge = 0, Multiplicity = 1

105

P	1.056547	-1.189072	-1.074125

P	0.250962	1.726656	-0.713266
C	0.700545	-0.267334	-2.677460
H	1.381405	-0.611625	-3.474004
H	-0.313515	-0.577869	-2.977556
C	0.780661	1.245686	-2.460747
H	0.195881	1.806205	-3.211413
H	1.827329	1.587763	-2.528414
C	3.057052	0.515258	0.369814
C	3.830850	-0.469192	1.053290
C	3.756831	1.399901	-0.479836
C	5.217113	-0.562239	0.886810
C	5.146176	1.300113	-0.658531
H	3.222247	2.193509	-1.012109
C	5.882951	0.323769	0.029131
H	5.742506	-1.352322	1.434257
H	5.654095	1.997284	-1.335338
H	6.967462	0.252042	-0.109490
C	3.096243	-1.468693	1.894526
N	1.762489	-1.298757	1.734208
C	0.852193	-2.195513	2.419484
H	0.857447	-3.218124	1.976229
H	1.134611	-2.331383	3.483001
C	-0.506186	-1.578738	2.277514
C	-1.765656	-1.809742	2.804644
N	-0.624761	-0.477329	1.455544
N	-1.859482	-0.023309	1.458165
N	-2.556445	-0.826349	2.276412
H	-2.148199	-2.557654	3.495989
O	3.639285	-2.350403	2.589152
Fe	1.042314	0.197907	0.677604
C	2.652513	-2.043465	-1.344604
C	2.993807	-3.132779	-0.516104
C	3.622620	-1.528356	-2.225323
C	4.271064	-3.700751	-0.576119
H	2.269651	-3.522460	0.203812
C	4.899359	-2.100240	-2.286341
H	3.398899	-0.657602	-2.848754
C	5.226856	-3.186328	-1.462493
H	4.523393	-4.525048	0.097542
H	5.647646	-1.679009	-2.965633
H	6.231965	-3.619602	-1.499556
C	-0.191546	-2.552807	-1.220825
C	0.115609	-3.856351	-1.658757
C	-1.539692	-2.229935	-0.952942
C	-0.896061	-4.816939	-1.804552
H	1.149054	-4.121798	-1.897850
C	-2.549726	-3.186673	-1.114750
H	-1.800467	-1.219621	-0.625143
C	-2.230932	-4.487489	-1.532069
H	-0.637052	-5.825768	-2.144051
H	-3.590666	-2.912600	-0.914467
H	-3.019774	-5.238001	-1.651493
C	-3.992305	-0.582870	2.427083
H	-4.327669	-1.136001	3.319241
H	-4.112452	0.495726	2.621813
C	-4.770760	-0.987953	1.170575
C	-6.229721	-0.520430	1.199491
H	-4.719751	-2.085824	1.049546
H	-4.259726	-0.536980	0.301998
C	-6.991631	-0.887092	-0.081155
H	-6.751915	-0.950402	2.077281
H	-6.251511	0.578938	1.333647
C	-8.438184	-0.374230	-0.104001
H	-6.444250	-0.479194	-0.953873
H	-6.988921	-1.988114	-0.207515
H	-8.981748	-0.779659	0.771708
H	-8.432794	0.726154	0.023879
C	-9.188410	-0.738485	-1.390070
H	-9.238147	-1.834383	-1.525193
H	-8.683116	-0.316778	-2.278219
C	-1.582859	1.937168	-0.883142
C	-2.349228	1.160309	-1.772585
C	-2.255334	2.840020	-0.033579
C	-3.748381	1.264721	-1.799165
H	-1.861540	0.463470	-2.458217
C	-3.650401	2.945755	-0.058243
H	-1.678239	3.468316	0.650715
C	-4.405633	2.152932	-0.937208
H	-4.322765	0.651932	-2.502728
H	-4.151402	3.656242	0.608549
H	-5.497640	2.236812	-0.958719
C	0.755656	3.512818	-0.755414
C	1.767567	3.992243	0.094805
C	0.164616	4.402182	-1.677497
C	2.185670	5.329320	0.027047
H	2.231318	3.313465	0.814455
C	0.581233	5.737464	-1.746909
H	-0.636472	4.049058	-2.336459
C	1.593899	6.204253	-0.893832
H	2.974336	5.684376	0.698760
H	0.112468	6.417156	-2.466896
H	1.917757	7.249275	-0.946527
H	-10.222956	-0.353521	-1.376815
C	1.184078	1.368220	2.559621
C	0.085014	2.420083	2.657424
C	2.499298	1.857758	3.185525
H	0.884723	0.476736	3.130312
H	-0.881799	2.034581	2.295256
H	-0.052181	2.734197	3.714793
H	0.326423	3.333296	2.086032
H	3.273606	1.072768	3.182091
H	2.919774	2.738873	2.663036
H	2.328632	2.159003	4.238775
H	2.133408	1.156168	1.228181

[1]TS(0-1')

Lowest frequency = -1929.2069 cm^{-1}
Charge = 0, Multiplicity = 1

105

N	-0.647618	-0.439818	1.483498
C	-0.528976	-1.612725	2.205074
C	-1.793119	-1.916278	2.675326
N	-2.596637	-0.904995	2.220454
N	-1.895848	-0.010924	1.491397
C	0.839397	-2.212593	2.310698
N	1.748399	-1.249795	1.707998
C	3.087010	-1.426945	1.877563
O	3.605862	-2.339917	2.551059
C	-4.037894	-0.705901	2.359052
C	-4.791633	-1.014893	1.060343
C	-6.249733	-0.544876	1.094106
C	-6.991958	-0.823805	-0.219730
C	-8.435609	-0.302536	-0.233323
C	-9.165575	-0.577570	-1.552834
Fe	1.010748	0.244460	0.690433
C	3.088522	0.623865	0.433299
C	3.836475	-0.418879	1.073576
C	5.207271	-0.574924	0.840513
C	5.882604	0.279142	-0.039754

C	5.163714	1.294554	-0.699094
C	3.800097	1.477959	-0.451357
P	0.254349	1.701533	-0.774393
C	0.760399	1.190724	-2.511728
C	0.603124	-0.321919	-2.680708
P	1.040756	-1.216565	-1.086646
C	2.660068	-1.980859	-1.417850
C	3.118941	-3.031085	-0.591777
C	4.417492	-3.533199	-0.728008
C	5.290335	-2.982542	-1.676408
C	4.855256	-1.922151	-2.484376
C	3.555985	-1.419944	-2.352950
C	0.789887	3.473680	-0.758692
C	1.696942	3.937138	0.210052
C	2.111531	5.276789	0.216698
C	1.618773	6.168072	-0.746269
C	0.708207	5.716688	-1.714990
C	0.293063	4.379070	-1.718674
C	-1.572543	1.944662	-0.894048
C	-2.383764	1.240697	-1.803592
C	-3.778308	1.394706	-1.786448
C	-4.381943	2.254747	-0.858430
C	-3.579866	2.970179	0.044632
C	-2.189427	2.818559	0.025255
C	-0.164853	-2.607858	-1.161595
C	0.165216	-3.953021	-1.428708
C	-0.832239	-4.936382	-1.476294
C	-2.178377	-4.594779	-1.276431
C	-2.518958	-3.257273	-1.023031
C	-1.524022	-2.275221	-0.950434
C	1.008261	1.327579	3.091952
C	0.056947	2.307915	2.701121
C	2.415901	1.654636	3.477538
H	1.208922	-0.714491	-3.515050
H	-0.442799	-0.595863	-2.893847
H	0.200113	1.761312	-3.273755
H	1.823539	1.480181	-2.581358
H	3.282934	2.308907	-0.941839
H	5.712459	-1.403274	1.348803
H	5.676370	1.961115	-1.402507
H	6.953487	0.148690	-0.228545
H	0.868928	-3.193177	1.783995
H	1.124439	-2.427846	3.359611
H	-2.174128	-2.735189	3.282042
H	2.469818	-3.446713	0.182873
H	3.249365	-0.574304	-2.975771
H	4.752568	-4.334795	-0.062601
H	5.537838	-1.467751	-3.210206
H	6.312187	-3.364306	-1.772922
H	1.205119	-4.230763	-1.620745
H	-1.800916	-1.237951	-0.732128
H	-0.554948	-5.974700	-1.689390
H	-3.566801	-2.975387	-0.878211
H	-2.956642	-5.363969	-1.324062
H	-4.379650	-1.337954	3.194865
H	-4.186874	0.349956	2.642292
H	-4.742333	-2.100950	0.860925
H	-4.260944	-0.505091	0.237372
H	-6.788248	-1.029070	1.933108
H	-6.271181	0.543509	1.299988
H	-6.428338	-0.363782	-1.055292
H	-6.992063	-1.914311	-0.417097
H	-8.995970	-0.761219	0.604750
H	-8.427673	0.787136	-0.033593
H	-9.217843	-1.661960	-1.760434
H	-8.643227	-0.101532	-2.402883
H	-1.936992	0.567519	-2.539196
H	-1.569907	3.383874	0.727433
H	-4.391915	0.842746	-2.506826
H	-4.040729	3.654979	0.764969
H	-5.470602	2.375717	-0.846807
H	2.092076	3.239236	0.953054
H	-0.435606	4.038010	-2.462506
H	2.822109	5.621258	0.975437
H	0.316535	6.410754	-2.466449
H	1.941207	7.214799	-0.742243
H	-10.198514	-0.188512	-1.531867
H	0.643048	0.355621	3.435386
H	0.306196	1.703479	1.351357
H	-1.009322	2.095103	2.822596
H	0.353379	3.362422	2.655169
H	3.113781	0.831146	3.251276
H	2.778488	2.580764	2.999127
H	2.450177	1.817827	4.576355
H	2.282353	1.197144	1.102167

[1]I-1'

Lowest frequency = 13.7219 cm^{-1}
Charge = 0, Multiplicity = 1

96

P	1.182611	-1.007934	-0.967898
P	0.223389	1.836361	-0.481030
C	0.783432	-0.009264	-2.518644
H	1.451491	-0.293493	-3.349275
H	-0.231915	-0.326507	-2.808878
C	0.825737	1.496119	-2.231420
H	0.259131	2.071202	-2.984999
H	1.867355	1.859931	-2.242736
C	3.192132	0.832323	0.625666
C	3.935094	-0.210898	1.234408
C	3.840236	1.726550	-0.248447
C	5.294141	-0.368830	0.933928
C	5.192858	1.544412	-0.565219
H	3.293108	2.582382	-0.658711
C	5.920484	0.498910	0.030488
H	5.828104	-1.194113	1.415843
H	5.687852	2.232494	-1.259589
H	6.980136	0.364597	-0.213557
C	3.221073	-1.188989	2.117629
N	1.882410	-1.014295	2.029010
C	1.033946	-1.944585	2.750968
H	1.189342	-2.992751	2.408995
H	1.260762	-1.953033	3.836658
C	-0.370976	-1.497228	2.490153
C	-1.628527	-1.809588	2.976679
N	-0.554053	-0.480276	1.576707
N	-1.822185	-0.156073	1.479368
N	-2.481837	-0.957426	2.329103
H	-1.973299	-2.531526	3.714069
O	3.798365	-2.071610	2.785977
Fe	1.038149	0.311801	0.844520
C	2.799130	-1.809038	-1.264738
C	3.192815	-2.839123	-0.382156
C	3.727060	-1.334926	-2.211118
C	4.470910	-3.401138	-0.466467
H	2.504756	-3.188320	0.392433
C	5.006146	-1.900617	-2.295108
H	3.463821	-0.509300	-2.879498
C	5.380218	-2.935895	-1.426882

H	4.761120	-4.180591	0.244484
H	5.718511	-1.518147	-3.033849
H	6.385378	-3.366567	-1.487043
C	-0.034000	-2.387151	-1.172279
C	0.304457	-3.722591	-1.463169
C	-1.399486	-2.042547	-1.060512
C	-0.696633	-4.691629	-1.626588
H	1.353903	-4.007564	-1.578306
C	-2.396542	-3.008624	-1.242052
H	-1.682114	-1.009804	-0.830756
C	-2.049076	-4.339831	-1.518203
H	-0.414156	-5.725395	-1.853997
H	-3.449642	-2.718300	-1.169830
H	-2.828818	-5.096796	-1.655458
C	-3.933689	-0.800211	2.444001
H	-4.269703	-1.475491	3.247257
H	-4.127520	0.238552	2.763337
C	-4.641319	-1.087856	1.116219
C	-6.120186	-0.687676	1.130206
H	-4.533078	-2.160800	0.874170
H	-4.117148	-0.519580	0.328352
C	-6.807880	-0.939204	-0.218507
H	-6.659146	-1.234908	1.929161
H	-6.199233	0.387974	1.383375
C	-8.271310	-0.478276	-0.259327
H	-6.237459	-0.419530	-1.013475
H	-6.754714	-2.019189	-0.461643
H	-8.838617	-0.995252	0.539203
H	-8.316308	0.601247	-0.014441
C	-8.945348	-0.726260	-1.613501
H	-8.944395	-1.801977	-1.867634
H	-8.416148	-0.193032	-2.424474
C	-1.602422	2.047262	-0.663927
C	-2.343345	1.493422	-1.723715
C	-2.291690	2.713336	0.370288
C	-3.743908	1.593748	-1.745561
H	-1.836395	0.977341	-2.544408
C	-3.685538	2.822024	0.344564
H	-1.724888	3.141911	1.203955
C	-4.419044	2.257011	-0.712171
H	-4.305496	1.157178	-2.578850
H	-4.205194	3.349281	1.152339
H	-5.511155	2.339540	-0.732279
C	0.717080	3.602636	-0.242421
C	1.310451	3.997029	0.970486
C	0.515448	4.564002	-1.254230
C	1.706569	5.328231	1.166667
H	1.446334	3.245308	1.756043
C	0.916665	5.891446	-1.059519
H	0.032483	4.277080	-2.194722
C	1.515494	6.275345	0.151403
H	2.168113	5.622968	2.115164
H	0.758428	6.630717	-1.852342
H	1.828841	7.314060	0.302200
H	-9.994090	-0.381455	-1.612156
H	2.309519	1.288015	1.243559
H	0.859079	1.296773	2.034349

[1]TS(1'-1)

Lowest frequency = -1098.4277 cm^{-1}

Charge = 0, Multiplicity = 1

96

P	1.163808	-1.030525	-0.998918
P	0.287735	1.827237	-0.499437
C	0.808047	-0.017925	-2.549275
H	1.490470	-0.313516	-3.365252
H	-0.205726	-0.317460	-2.862584
C	0.874197	1.485113	-2.253551
H	0.309198	2.073377	-2.997909
H	1.920738	1.834404	-2.268978
C	3.110576	0.704912	0.600904
C	3.904638	-0.288919	1.240921
C	3.779864	1.686163	-0.161713
C	5.296606	-0.317551	1.089844
C	5.173429	1.648828	-0.325654
H	3.218016	2.507998	-0.620611
C	5.935710	0.646636	0.298797
H	5.846653	-1.115540	1.599741
H	5.667722	2.415218	-0.933252
H	7.023793	0.625330	0.171670
C	3.190604	-1.331476	2.043836
N	1.852437	-1.165163	1.919608
C	0.972132	-2.111585	2.575328
H	1.039461	-3.128622	2.124084
H	1.235484	-2.244534	3.644208
C	-0.409069	-1.556832	2.409865
C	-1.667548	-1.839286	2.913444
N	-0.559014	-0.463327	1.584030
N	-1.809554	-0.062102	1.559300
N	-2.488309	-0.890611	2.366361
H	-2.031691	-2.600167	3.600641
O	3.753836	-2.232355	2.697611
Fe	1.080592	0.266884	0.806929
C	2.787910	-1.820612	-1.266098
C	3.148140	-2.913958	-0.450978
C	3.763413	-1.239128	-2.098566
C	4.449651	-3.426752	-0.483137
H	2.419234	-3.346868	0.239386
C	5.064523	-1.755309	-2.130515
H	3.522260	-0.360980	-2.705243
C	5.410222	-2.849449	-1.324433
H	4.716337	-4.254543	0.180473
H	5.816902	-1.284697	-2.771835
H	6.433842	-3.238457	-1.337805
C	-0.059422	-2.397606	-1.230207
C	0.268772	-3.696720	-1.663149
C	-1.416451	-2.075706	-1.008426
C	-0.736112	-4.656641	-1.854715
H	1.312024	-3.957742	-1.862613
C	-2.418235	-3.031565	-1.217233
H	-1.686628	-1.068026	-0.675920
C	-2.080877	-4.328738	-1.632971
H	-0.464251	-5.663295	-2.190615
H	-3.466689	-2.760804	-1.055433
H	-2.863784	-5.078610	-1.789734
C	-3.934670	-0.699064	2.493525
H	-4.276982	-1.335263	3.325551
H	-4.097988	0.355264	2.773618
C	-4.662091	-1.020910	1.183519
C	-6.136330	-0.604074	1.201205
H	-4.569130	-2.102555	0.974554
H	-4.140664	-0.483730	0.372047
C	-6.842594	-0.889309	-0.131141
H	-6.671395	-1.120805	2.022696
H	-6.200868	0.479548	1.422311
C	-8.303474	-0.419804	-0.166536
H	-6.279063	-0.396777	-0.948070
H	-6.799646	-1.976174	-0.343688
H	-8.863406	-0.909907	0.653832

H	-8.338539	0.666554	0.047654
C	-8.996645	-0.702373	-1.504165
H	-9.005602	-1.784957	-1.727036
H	-8.475034	-0.196105	-2.337009
C	-1.542185	2.036434	-0.676260
C	-2.294859	1.423433	-1.694731
C	-2.222833	2.767983	0.318783
C	-3.694785	1.530260	-1.714280
H	-1.797656	0.855078	-2.485684
C	-3.616401	2.881793	0.296629
H	-1.650072	3.247637	1.119812
C	-4.360188	2.258345	-0.718857
H	-4.263417	1.047357	-2.516667
H	-4.127165	3.460231	1.074474
H	-5.451942	2.346081	-0.737388
C	0.755684	3.602537	-0.272198
C	1.413128	4.011065	0.901743
C	0.455297	4.562999	-1.260172
C	1.774080	5.354224	1.083421
H	1.633950	3.266276	1.673220
C	0.822056	5.902447	-1.081818
H	-0.080182	4.264026	-2.168004
C	1.484192	6.300178	0.090942
H	2.286485	5.658582	2.002187
H	0.586894	6.639986	-1.856908
H	1.769816	7.348382	0.230348
H	-10.043144	-0.350935	-1.499040
H	2.181347	1.198092	1.430068
H	1.071742	1.247854	2.037509

^{1}I-1

Lowest frequency = 11.1840 cm^{-1}

Charge = 0, Multiplicity = 1

107

P	1.079817	-1.352576	-1.106691
P	0.348077	1.527629	-1.181828
C	1.461981	-0.552993	-2.753238
H	2.538298	-0.322650	-2.729532
H	1.270854	-1.251142	-3.585810
C	0.642604	0.738154	-2.884364
H	-0.341448	0.533096	-3.336325
H	1.150463	1.474679	-3.529347
C	3.036494	0.337472	-0.045553
C	3.846015	-0.435047	0.838973
C	3.739968	1.081517	-1.019018
C	5.245705	-0.450344	0.774051
C	5.145649	1.071285	-1.101502
H	3.197379	1.695404	-1.749497
C	5.907131	0.307465	-0.202485
H	5.785244	-1.082019	1.489461
H	5.645816	1.666917	-1.875411
H	7.001154	0.300768	-0.269646
C	3.123450	-1.291367	1.837858
N	1.789910	-1.055551	1.742579
C	0.881735	-1.833750	2.556859
H	0.821540	-2.895213	2.224823
H	1.208173	-1.875331	3.615840
C	-0.451072	-1.167712	2.407381
C	-1.684161	-1.248676	3.031511
N	-0.569922	-0.207939	1.421650
N	-1.784481	0.307649	1.421888
N	-2.464672	-0.318054	2.398448
H	-2.056009	-1.859799	3.851399
O	3.665153	-2.076187	2.638365
Fe	1.091656	0.185646	0.400832
C	2.324575	-2.695000	-0.934351
C	1.977513	-3.859614	-0.220737
C	3.649882	-2.537154	-1.380745
C	2.936012	-4.843441	0.045942
H	0.948587	-3.995344	0.129700
C	4.604029	-3.531040	-1.125915
H	3.955717	-1.622396	-1.896744
C	4.252782	-4.681904	-0.408584
H	2.655714	-5.735141	0.616562
H	5.633346	-3.387645	-1.470413
H	5.005781	-5.447571	-0.193945
C	-0.417745	-2.367903	-1.547421
C	-0.313414	-3.372655	-2.534393
C	-1.667609	-2.141326	-0.945180
C	-1.432494	-4.124736	-2.910601
H	0.658004	-3.576132	-2.999019
C	-2.790509	-2.896349	-1.320776
H	-1.766141	-1.354883	-0.197365
C	-2.676697	-3.888225	-2.303116
H	-1.334155	-4.900951	-3.677361
H	-3.757442	-2.702674	-0.844132
H	-3.552065	-4.478086	-2.595978
C	-3.892957	-0.032222	2.539375
H	-4.200543	-0.360434	3.545707
H	-4.003774	1.062852	2.478703
C	-4.707432	-0.713610	1.433483
C	-6.179112	-0.287408	1.424740
H	-4.624098	-1.811229	1.543476
H	-4.241118	-0.444347	0.469829
C	-6.970269	-0.926732	0.275479
H	-6.656553	-0.544459	2.391197
H	-6.234024	0.815344	1.335139
C	-8.439720	-0.486461	0.219969
H	-6.476188	-0.676232	-0.684214
H	-6.919815	-2.030251	0.364962
H	-8.930278	-0.736170	1.181093
H	-8.483060	0.617001	0.133348
C	-9.217875	-1.123957	-0.936576
H	-9.219154	-2.226321	-0.855704
H	-8.766629	-0.862614	-1.911207
C	-1.475652	1.877533	-1.239959
C	-2.381693	0.957713	-1.802012
C	-1.998592	3.006083	-0.574672
C	-3.765717	1.150347	-1.694861
H	-2.013585	0.070044	-2.324010
C	-3.381383	3.194999	-0.455380
H	-1.314662	3.749271	-0.151836
C	-4.273257	2.265028	-1.011620
H	-4.447797	0.423835	-2.150610
H	-3.764633	4.080116	0.064743
H	-5.355085	2.416402	-0.927269
C	0.979820	3.255605	-1.419741
C	1.967203	3.765403	-0.557405
C	0.496174	4.066679	-2.467840
C	2.459830	5.067081	-0.736897
H	2.345691	3.125358	0.243420
C	0.992236	5.363713	-2.648645
H	-0.282658	3.683901	-3.137604
C	1.975374	5.866947	-1.780931
H	3.231374	5.452563	-0.061495
H	0.610693	5.985118	-3.466353
H	2.362823	6.881894	-1.922022
H	-10.268732	-0.786128	-0.950580
O	1.376800	1.785387	1.790496

C	0.278159	2.592911	2.254614
C	2.316985	1.765974	2.886311
C	0.084562	2.200575	3.733425
H	0.564040	3.658169	2.149014
H	-0.585626	2.378500	1.612436
C	1.440097	1.545783	4.123839
H	3.053508	0.980328	2.686717
H	2.824030	2.751536	2.914421
H	-0.744930	1.482961	3.840676
H	-0.152361	3.082954	4.351272
H	1.314434	0.465939	4.297497
H	1.885331	1.989443	5.029562

^{1}I-2

Lowest frequency = 11.6501 cm^{-1}

Charge = 0, Multiplicity = 1

126

P	-0.454506	1.697169	-1.628545
P	-0.945493	-1.313181	-1.781555
C	-0.588672	0.980049	-3.361782
H	-1.063455	1.707488	-4.040304
H	0.449370	0.858164	-3.709022
C	-1.332287	-0.358809	-3.347996
H	-1.112601	-0.960809	-4.247180
H	-2.416167	-0.171130	-3.322890
C	-2.859252	0.854575	-0.380889
C	-3.186072	1.891145	0.537005
C	-3.866218	0.490722	-1.297584
C	-4.430798	2.536332	0.523577
C	-5.112513	1.140679	-1.326501
H	-3.721737	-0.356396	-1.976697
C	-5.402251	2.169448	-0.416972
H	-4.597798	3.332509	1.257949
H	-5.865997	0.826769	-2.059302
H	-6.374925	2.673337	-0.441169
C	-2.113224	2.330492	1.478110
N	-0.983622	1.624737	1.234988
C	0.239889	2.016287	1.904046
H	0.544735	3.054082	1.633966
H	0.140500	2.016599	3.006376
C	1.279112	1.043579	1.451732
C	2.616039	0.833815	1.737685
N	0.927592	0.095837	0.519517
N	1.951830	-0.670017	0.219052
N	2.983148	-0.223683	0.955376
H	3.305169	1.338352	2.409830
O	-2.221120	3.254896	2.311887
Fe	-0.988943	0.135776	-0.068409
C	-1.466150	3.222857	-1.635330
C	-1.306078	4.167008	-0.600252
C	-2.539772	3.378775	-2.534085
C	-2.186255	5.248094	-0.477107
H	-0.524806	4.036501	0.150928
C	-3.417895	4.461429	-2.410346
H	-2.723144	2.634778	-3.314193
C	-3.243580	5.399371	-1.382602
H	-2.061427	5.945049	0.356704
H	-4.257121	4.556900	-3.106991
H	-3.944688	6.233850	-1.276153
C	1.303206	2.290121	-1.689575
C	1.715568	3.625581	-1.521285
C	2.284907	1.313826	-1.967564
C	3.073039	3.970266	-1.611532
H	0.976610	4.409858	-1.342864
C	3.635028	1.662707	-2.080099
H	1.994969	0.267583	-2.099100
C	4.038280	2.993652	-1.891310
H	3.371586	5.015788	-1.479036
H	4.370979	0.887710	-2.321975
H	5.095337	3.269216	-1.974414
C	-2.391205	-1.061658	0.965795
C	-1.243072	-1.154201	1.502441
C	-3.814037	-1.472685	0.975985
C	-4.076869	-2.554508	2.040479
H	-4.099521	-1.848305	-0.023401
H	-4.457128	-0.591329	1.154665
C	-5.511785	-3.086771	1.975093
H	-3.360036	-3.383383	1.899978
H	-3.873586	-2.131631	3.042799
H	-5.695955	-3.843942	2.757599
H	-6.246147	-2.272225	2.111135
H	-5.709816	-3.555367	0.993884
C	-0.594864	-1.576074	2.768030
C	4.295050	-0.858381	0.811733
H	4.445153	-1.565356	1.647053
H	4.229676	-1.443155	-0.119674
C	5.438181	0.158068	0.751381
C	6.771224	-0.500890	0.373378
H	5.542735	0.663178	1.730060
H	5.180543	0.940458	0.016576
C	7.935105	0.497009	0.307897
H	7.012331	-1.300883	1.101184
H	6.662366	-1.002708	-0.608452
C	9.271711	-0.146448	-0.087608
H	7.685378	1.299438	-0.414731
H	8.044487	0.996428	1.291091
H	9.515325	-0.948988	0.635821
H	9.156390	-0.645990	-1.069432
C	10.429460	0.855970	-0.153218
H	10.588436	1.345237	0.825057
H	10.226130	1.651050	-0.893655
C	0.620940	-2.199854	-2.254598
C	1.398375	-1.820332	-3.366563
C	1.085916	-3.260616	-1.450597
C	2.616997	-2.456913	-3.650443
H	1.062864	-1.022286	-4.033612
C	2.286757	-3.914268	-1.748377
H	0.505044	-3.589177	-0.586029
C	3.068517	-3.506309	-2.840874
H	3.203154	-2.136690	-4.518879
H	2.614100	-4.750987	-1.121634
H	4.012126	-4.014215	-3.067864
C	-2.141798	-2.718544	-1.839461
C	-2.140582	-3.640813	-0.770722
C	-3.079346	-2.886307	-2.878590
C	-3.048155	-4.707301	-0.752781
H	-1.455636	-3.514726	0.071757
C	-3.998276	-3.945046	-2.847075
H	-3.104134	-2.192604	-3.723698
C	-3.985171	-4.860092	-1.784916
H	-3.028348	-5.411709	0.085380
H	-4.723687	-4.055521	-3.660412
H	-4.702485	-5.687438	-1.762252
O	0.774026	-2.066403	2.593067
C	0.896083	-3.312072	2.093272
O	-0.048056	-4.008613	1.758725
C	2.345988	-3.718776	1.976607
H	2.416578	-4.815796	1.964256
H	2.731915	-3.326251	1.020870

H	2.945212	-3.297495	2.798463
H	-1.173695	-2.435437	3.155141
C	-0.548962	-0.475430	3.821001
C	-1.671255	0.353341	3.992294
C	0.582101	-0.276195	4.631042
C	-1.657963	1.382098	4.941097
H	-2.540831	0.223156	3.343364
C	0.591467	0.748385	5.590013
H	1.457787	-0.916215	4.496991
C	-0.524332	1.583500	5.743384
H	-2.520210	2.049559	5.018832
H	1.480868	0.899778	6.212196
H	-0.507330	2.396659	6.476830
H	11.374115	0.362207	-0.440227

[1]TS(2-3)

Lowest frequency = -280.7414 cm^{-1}

Charge = 0, Multiplicity = 1

126

P	-0.510431	1.946040	-1.415795
P	-0.744520	-1.057591	-1.948501
C	-0.501314	1.446875	-3.227230
H	-0.995022	2.214015	-3.846879
H	0.559216	1.440090	-3.524957
C	-1.140517	0.067242	-3.402972
H	-0.849369	-0.400953	-4.359946
H	-2.238809	0.168800	-3.390428
C	-2.904883	0.559892	-0.161589
C	-3.252484	1.561513	0.799380
C	-3.846515	0.315485	-1.194937
C	-4.483827	2.226395	0.753072
C	-5.058616	1.011618	-1.262602
H	-3.655200	-0.479273	-1.924326
C	-5.394699	1.957330	-0.277072
H	-4.680805	2.979036	1.524309
H	-5.756922	0.800697	-2.081241
H	-6.352606	2.486599	-0.323929
C	-2.202023	1.974751	1.777836
N	-1.013136	1.442878	1.410924
C	0.178029	1.798808	2.158061
H	0.446832	2.871522	2.022548
H	0.040226	1.656097	3.247378
C	1.267250	0.924250	1.622765
C	2.582886	0.664387	1.960778
N	0.986835	0.139768	0.526890
N	2.035239	-0.569049	0.172554
N	3.010335	-0.254134	1.042664
H	3.219889	1.040615	2.757600
O	-2.386630	2.760245	2.732246
Fe	-0.874162	0.161812	-0.084510
C	-1.725692	3.308477	-1.287494
C	-1.714745	4.139575	-0.146052
C	-2.768030	3.457456	-2.222422
C	-2.701393	5.114503	0.036052
H	-0.953336	4.006310	0.625502
C	-3.755095	4.433767	-2.037813
H	-2.829652	2.798493	-3.093353
C	-3.722506	5.267351	-0.911720
H	-2.684013	5.729378	0.940974
H	-4.563242	4.529317	-2.770532
H	-4.502693	6.021575	-0.763816
C	1.136717	2.791657	-1.328014
C	1.333365	4.163161	-1.078996
C	2.267811	1.984266	-1.580744
C	2.626300	4.708615	-1.066066
H	0.474631	4.818515	-0.913859
C	3.553957	2.535576	-1.591506
H	2.142086	0.914390	-1.772675
C	3.741410	3.899991	-1.322613
H	2.756694	5.778417	-0.869949
H	4.412926	1.895137	-1.820370
H	4.748370	4.331027	-1.324029
C	-2.519337	-1.049272	0.792264
C	-1.307354	-1.244974	1.221195
C	-3.870832	-1.694356	0.882497
C	-3.897306	-2.895451	1.840762
H	-4.168969	-2.030756	-0.128248
H	-4.623791	-0.939827	1.181525
C	-5.257929	-3.600925	1.829856
H	-3.105011	-3.609648	1.552431
H	-3.659287	-2.555761	2.866341
H	-5.273661	-4.452292	2.532755
H	-6.069390	-2.908214	2.117767
H	-5.490985	-3.987887	0.821122
C	-0.697154	-1.942378	2.387047
C	4.325923	-0.876116	0.893902
H	4.492940	-1.562687	1.743341
H	4.252358	-1.479300	-0.024799
C	5.459335	0.148163	0.788026
C	6.807227	-0.515073	0.476979
H	5.536008	0.721471	1.731151
H	5.203617	0.876532	-0.000991
C	7.957253	0.493306	0.351814
H	7.049785	-1.254018	1.266294
H	6.719760	-1.090954	-0.465540
C	9.308992	-0.156716	0.024232
H	7.706763	1.234708	-0.432995
H	8.044698	1.067397	1.295657
H	9.554937	-0.897158	0.810379
H	9.215050	-0.732055	-0.917565
C	10.451941	0.856623	-0.103945
H	10.589548	1.422620	0.835421
H	10.246703	1.588633	-0.906321
C	0.859286	-1.837778	-2.461636
C	1.770727	-1.178640	-3.309942
C	1.234327	-3.083713	-1.919984
C	3.033850	-1.726725	-3.579346
H	1.505365	-0.226171	-3.774993
C	2.486265	-3.641508	-2.205123
H	0.543887	-3.630358	-1.271949
C	3.400781	-2.958853	-3.022816
H	3.725430	-1.190462	-4.238692
H	2.748093	-4.619354	-1.786305
H	4.382124	-3.394382	-3.240220
C	-1.911294	-2.465158	-2.232909
C	-2.017587	-3.458754	-1.238105
C	-2.710873	-2.580339	-3.389460
C	-2.895939	-4.538770	-1.397700
H	-1.425243	-3.389142	-0.323097
C	-3.601844	-3.652222	-3.538600
H	-2.647781	-1.835024	-4.187073
C	-3.698477	-4.635271	-2.542926
H	-2.954682	-5.299648	-0.612408
H	-4.218607	-3.720871	-4.441376
H	-4.394631	-5.472484	-2.661826
O	0.677410	-2.373372	2.132760
C	0.838948	-3.578495	1.553680
O	-0.081736	-4.290481	1.186506
C	2.304076	-3.913554	1.404391

H	2.414841	-4.984313	1.183468
H	2.705820	-3.324128	0.563837
H	2.861172	-3.644243	2.315810
H	-1.264272	-2.862990	2.613421
C	-0.652631	-1.047768	3.624883
C	-1.773332	-0.268308	3.958231
C	0.488396	-0.987021	4.444323
C	-1.751100	0.574519	5.075587
H	-2.652964	-0.282780	3.310388
C	0.507980	-0.150591	5.570526
H	1.365092	-1.586321	4.187183
C	-0.608119	0.637347	5.886384
H	-2.616063	1.210616	5.281590
H	1.405713	-0.108573	6.197891
H	-0.583977	1.305873	6.753587
H	11.408121	0.357910	-0.339744

[1]I-3

Lowest frequency = 8.2123 cm^{-1}

Charge = 0, Multiplicity = 1

126

P	-0.617191	-1.545765	1.350869
P	1.978302	-1.783407	-0.112640
C	0.510170	-2.967216	1.870483
H	0.358300	-3.199029	2.938217
H	0.197912	-3.846321	1.281109
C	1.974226	-2.621126	1.558580
H	2.601270	-3.529494	1.557077
H	2.393330	-1.897099	2.278463
C	3.756734	1.077526	0.359273
C	3.321092	0.754777	1.674063
C	5.134897	0.888975	0.074335
C	4.229628	0.198236	2.600868
C	6.021497	0.334732	0.995413
H	5.510443	1.161147	-0.915871
C	5.561896	-0.044277	2.268217
H	3.870365	0.020205	3.620221
H	7.070139	0.186886	0.717149
H	6.247580	-0.483254	3.001431
C	2.046629	1.312461	2.249855
N	0.890062	1.079325	1.608862
C	-0.174066	1.995194	2.048977
H	-0.555982	1.750669	3.057937
H	0.234055	3.021217	2.139919
C	-1.275980	1.951677	1.047002
C	-2.494907	2.586661	0.885490
N	-1.133668	1.113029	-0.028379
N	-2.176870	1.181761	-0.830298
N	-3.003387	2.086878	-0.281209
H	-3.017573	3.336111	1.474680
O	2.141003	2.034403	3.270916
Fe	0.500647	-0.095381	-0.058225
C	-1.080092	-0.823239	2.977434
C	-2.379528	-0.330705	3.207347
C	-0.084753	-0.618953	3.954705
C	-2.677106	0.353473	4.394824
H	-3.159369	-0.481636	2.453377
C	-0.378639	0.079389	5.131052
H	0.936792	-0.967593	3.781321
C	-1.675924	0.568897	5.353440
H	-3.692431	0.727944	4.566625
H	0.414657	0.263031	5.861898
H	-1.903596	1.120415	6.271786
C	-2.201014	-2.393517	0.909650
C	-2.765042	-3.403290	1.714409
C	-2.862748	-2.002845	-0.270256
C	-3.967098	-4.017946	1.339940
H	-2.266653	-3.703686	2.642678
C	-4.068820	-2.616803	-0.641341
H	-2.422661	-1.218434	-0.894329
C	-4.621958	-3.625037	0.161362
H	-4.396739	-4.804836	1.969433
H	-4.574597	-2.309931	-1.563876
H	-5.561776	-4.106798	-0.129768
C	2.908535	1.710886	-0.699629
C	1.649666	1.271653	-1.044411
C	3.613649	2.916507	-1.345310
C	4.160676	3.937523	-0.323575
H	2.929439	3.460282	-2.015998
H	4.453417	2.579538	-1.987732
C	4.622284	5.238981	-0.986234
H	3.378709	4.153974	0.428124
H	4.993488	3.484785	0.241914
H	5.034346	5.944806	-0.243380
H	5.409282	5.046073	-1.739173
H	3.784379	5.743174	-1.502689
C	0.960489	2.062469	-2.140553
C	-4.326348	2.284028	-0.866041
H	-4.647840	3.310505	-0.624716
H	-4.199350	2.214003	-1.958393
C	-5.322221	1.233171	-0.356964
C	-6.685532	1.303533	-1.053914
H	-5.446873	1.354731	0.735697
H	-4.873718	0.235368	-0.510260
C	-7.654055	0.216948	-0.565916
H	-7.139358	2.302090	-0.895917
H	-6.542441	1.201346	-2.148153
C	-9.016688	0.244482	-1.272124
H	-7.184566	-0.776660	-0.708830
H	-7.803724	0.325745	0.526620
H	-9.482245	1.238680	-1.124886
H	-8.861182	0.139693	-2.363851
C	-9.972573	-0.849028	-0.781902
H	-10.172274	-0.747318	0.300436
H	-9.544658	-1.854697	-0.947578
C	1.418452	-3.260585	-1.131158
C	2.333197	-4.256159	-1.536375
C	0.054365	-3.458394	-1.411738
C	1.888424	-5.412730	-2.192135
H	3.402267	-4.131188	-1.341546
C	-0.393448	-4.616861	-2.060217
H	-0.662895	-2.691164	-1.130180
C	0.523330	-5.600361	-2.455037
H	2.616784	-6.171785	-2.498486
H	-1.464373	-4.742294	-2.254765
H	0.179201	-6.506382	-2.965914
C	3.716481	-1.773519	-0.689276
C	3.942163	-1.249748	-1.979645
C	4.775418	-2.382851	0.003587
C	5.209612	-1.335200	-2.563295
H	3.113898	-0.771161	-2.512280
C	6.044950	-2.472324	-0.587741
H	4.620612	-2.784946	1.008318
C	6.264596	-1.951537	-1.869184
H	5.378011	-0.922582	-3.564019
H	6.865852	-2.945885	-0.038644
H	7.256629	-2.022330	-2.328130
O	0.107362	1.321467	-3.118868
C	-0.411988	0.110709	-3.041308

O	-0.282550	-0.713997	-2.134912
C	-1.272215	-0.201905	-4.244480
H	-2.292132	0.164355	-4.028645
H	-0.900873	0.307482	-5.146161
H	-1.313136	-1.290894	-4.392321
H	1.707183	2.450145	-2.854346
C	0.135411	3.263507	-1.668864
C	0.546475	3.984885	-0.534616
C	-0.988154	3.718371	-2.385259
C	-0.137949	5.138824	-0.129482
H	1.392279	3.613547	0.048195
C	-1.683948	4.862495	-1.970720
H	-1.320060	3.164773	-3.267157
C	-1.259658	5.582217	-0.842964
H	0.203176	5.681415	0.758794
H	-2.558274	5.199148	-2.539931
H	-1.798805	6.481698	-0.525874
H	-10.941538	-0.804201	-1.308989

^{1}TS(3-4)

Lowest frequency = -160.5817 cm^{-1}

Charge = 0, Multiplicity = 1

126

P	-0.332893	-1.533387	1.436965
P	2.021815	-1.528931	-0.251591
C	1.178674	-2.182838	2.325178
H	1.435806	-1.381718	3.036468
H	0.946759	-3.090401	2.907183
C	2.367253	-2.397853	1.361401
H	2.575121	-3.465501	1.190495
H	3.277713	-1.940943	1.778221
C	3.771802	1.149120	0.616167
C	3.346206	0.705673	1.897205
C	5.142528	0.965751	0.292644
C	4.267473	0.086438	2.770254
C	6.031285	0.311775	1.143738
H	5.506862	1.309219	-0.679017
C	5.590033	-0.149556	2.395607
H	3.913539	-0.170907	3.773973
H	7.069479	0.160883	0.829470
H	6.281214	-0.651851	3.081321
C	2.041808	1.122908	2.516917
N	0.905366	1.000096	1.803609
C	-0.170306	1.797214	2.419843
H	-0.505840	1.383557	3.388419
H	0.207367	2.811029	2.663724
C	-1.291115	1.860659	1.446201
C	-2.519009	2.492244	1.373562
N	-1.138442	1.171143	0.272831
N	-2.185451	1.326046	-0.512850
N	-3.020601	2.142343	0.151414
H	-3.052846	3.146900	2.058109
O	2.082347	1.624635	3.666480
Fe	0.462539	0.085653	0.045150
C	-1.469726	-1.051607	2.806016
C	-2.812430	-0.794011	2.457870
C	-1.072881	-0.903068	4.147950
C	-3.737045	-0.397403	3.429960
H	-3.130637	-0.910706	1.415642
C	-2.002299	-0.507499	5.121750
H	-0.032253	-1.066189	4.441029
C	-3.334321	-0.252235	4.766993
H	-4.776697	-0.202186	3.143966
H	-1.677902	-0.392745	6.161376
H	-4.056838	0.056991	5.529834
C	-1.262543	-3.033759	0.879008
C	-1.394091	-4.174561	1.695493
C	-1.892295	-3.008781	-0.379081
C	-2.121003	-5.284208	1.246662
H	-0.933254	-4.197239	2.689399
C	-2.630961	-4.115780	-0.819368
H	-1.751554	-2.131388	-1.015207
C	-2.740271	-5.257183	-0.013809
H	-2.211959	-6.170473	1.884372
H	-3.105436	-4.092390	-1.806379
H	-3.307053	-6.127011	-0.363504
C	2.918835	1.876978	-0.367706
C	1.651912	1.518991	-0.750047
C	3.579561	3.127102	-0.949248
C	3.878000	4.202033	0.114979
H	2.928835	3.568267	-1.724598
H	4.529571	2.864696	-1.456651
C	4.510638	5.465577	-0.475996
H	2.937360	4.459962	0.635712
H	4.543137	3.768329	0.884208
H	4.718287	6.216565	0.306740
H	5.466318	5.233117	-0.981553
H	3.843958	5.933541	-1.223932
C	0.975459	2.235692	-1.786554
C	-4.315195	2.463473	-0.443979
H	-4.704816	3.347578	0.086168
H	-4.125746	2.749945	-1.491632
C	-5.288124	1.280989	-0.359545
C	-6.640396	1.577073	-1.018645
H	-5.434001	1.013034	0.704047
H	-4.811948	0.408806	-0.843651
C	-7.611751	0.390754	-0.949205
H	-7.104248	2.460294	-0.535845
H	-6.478181	1.856998	-2.078327
C	-8.968241	0.668242	-1.612486
H	-7.142295	-0.491567	-1.427689
H	-7.771822	0.111886	0.111416
H	-9.432697	1.551896	-1.132494
H	-8.802987	0.948310	-2.671287
C	-9.931240	-0.522171	-1.539770
H	-10.139927	-0.802010	-0.490993
H	-9.505221	-1.409814	-2.042065
C	1.380277	-2.934646	-1.303046
C	1.064492	-4.204353	-0.786596
C	1.210399	-2.720960	-2.686423
C	0.577202	-5.224317	-1.617232
H	1.170933	-4.415752	0.279406
C	0.725175	-3.735693	-3.518479
H	1.472112	-1.753627	-3.122934
C	0.399184	-4.993016	-2.985609
H	0.327137	-6.197238	-1.181986
H	0.605643	-3.544356	-4.590640
H	0.015978	-5.786549	-3.636070
C	3.650834	-1.390771	-1.099705
C	3.742974	-0.567380	-2.239950
C	4.768597	-2.148463	-0.707691
C	4.940296	-0.489498	-2.961489
H	2.878443	0.029233	-2.542761
C	5.966769	-2.066827	-1.429995
H	4.716728	-2.798863	0.170001
C	6.057920	-1.234342	-2.554250
H	5.003105	0.160617	-3.840895
H	6.833881	-2.654039	-1.109013
H	6.996552	-1.168648	-3.114943

O	-0.295099	1.150172	-2.954114
C	-0.703930	-0.000054	-2.624937
O	-0.415483	-0.627532	-1.561207
C	-1.658182	-0.693873	-3.583232
H	-1.551445	-0.293068	-4.602480
H	-1.485317	-1.781525	-3.570193
H	-2.686622	-0.496997	-3.230667
H	1.453863	2.293851	-2.772880
C	0.053846	3.355529	-1.502768
C	0.077520	3.965760	-0.231723
C	-0.785064	3.897795	-2.503933
C	-0.716614	5.086891	0.037356
H	0.743715	3.550738	0.529726
C	-1.591269	5.005691	-2.227395
H	-0.812718	3.417067	-3.484681
C	-1.560933	5.604476	-0.955124
H	-0.677249	5.553545	1.027600
H	-2.243344	5.413199	-3.008021
H	-2.185363	6.480100	-0.745337
H	-10.895561	-0.291003	-2.024951

[1]I-4

Lowest frequency = 9.4699 cm^{-1}

Charge = 0, Multiplicity = 1

126

P	0.786788	1.034890	1.403646
P	-1.833341	1.679253	-0.000672
C	-0.260567	2.384559	2.179422
H	-0.114153	2.384134	3.272497
H	0.122632	3.341668	1.789222
C	-1.731376	2.202220	1.797975
H	-2.286113	3.142260	1.955654
H	-2.211759	1.402291	2.385515
C	-3.890060	-1.022647	0.706681
C	-3.137206	-0.962523	1.910676
C	-5.276048	-0.741054	0.772855
C	-3.770768	-0.548229	3.101673
C	-5.882337	-0.313417	1.953139
H	-5.875614	-0.812073	-0.138768
C	-5.118346	-0.184808	3.125672
H	-3.186627	-0.575592	4.027487
H	-6.950056	-0.070075	1.958264
H	-5.584410	0.149634	4.058946
C	-1.814095	-1.663834	2.078965
N	-0.792597	-1.477853	1.213158
C	0.238926	-2.507937	1.445083
H	0.615932	-2.476811	2.483710
H	-0.198290	-3.518026	1.333392
C	1.338142	-2.291131	0.473753
C	2.542724	-2.906273	0.178025
N	1.242441	-1.214200	-0.361331
N	2.291918	-1.115104	-1.146958
N	3.084128	-2.144672	-0.819751
H	3.037655	-3.787983	0.577457
O	-1.748637	-2.480935	3.028836
Fe	-0.395964	-0.111585	-0.258049
C	1.265831	0.013250	2.850573
C	2.538012	-0.592059	2.901431
C	0.333118	-0.257210	3.869382
C	2.864467	-1.458135	3.953020
H	3.271898	-0.382984	2.116712
C	0.655489	-1.135856	4.909824
H	-0.665388	0.186572	3.835698
C	1.921318	-1.738977	4.953317
H	3.856320	-1.922123	3.986231
H	-0.094623	-1.366755	5.671528
H	2.170892	-2.430734	5.764623
C	2.398544	1.875513	1.065382
C	3.061996	2.555226	2.109365
C	2.980071	1.835245	-0.216358
C	4.289764	3.185559	1.874051
H	2.623290	2.578454	3.113065
C	4.212199	2.468778	-0.444524
H	2.445703	1.327184	-1.025177
C	4.869498	3.140655	0.595410
H	4.797416	3.710736	2.690227
H	4.656254	2.437969	-1.445746
H	5.831088	3.632837	0.412775
C	-3.286443	-1.424516	-0.578382
C	-2.040394	-1.087683	-0.960901
C	-4.146705	-2.199899	-1.572054
C	-4.714737	-3.521283	-1.021998
H	-3.539776	-2.402228	-2.471344
H	-4.985734	-1.556037	-1.908427
C	-5.573591	-4.261919	-2.052521
H	-3.876778	-4.166727	-0.700542
H	-5.309812	-3.318050	-0.113084
H	-5.970803	-5.206981	-1.641788
H	-6.434208	-3.644465	-2.370342
H	-4.988019	-4.506220	-2.957786
C	-1.065933	-1.389387	-1.894693
C	4.425818	-2.203660	-1.398256
H	4.743186	-3.259245	-1.399488
H	4.330147	-1.868814	-2.443299
C	5.396096	-1.306639	-0.617643
C	6.776381	-1.188492	-1.273235
H	5.496648	-1.695677	0.413526
H	4.937348	-0.305242	-0.533426
C	7.711606	-0.245611	-0.502917
H	7.244375	-2.189508	-1.356481
H	6.656432	-0.817507	-2.310403
C	9.089057	-0.073559	-1.157898
H	7.223706	0.744307	-0.404108
H	7.840479	-0.623754	0.530786
H	9.573464	-1.065117	-1.252799
H	8.954249	0.299868	-2.191941
C	10.009628	0.877104	-0.384225
H	10.188969	0.509794	0.642754
H	9.562488	1.884837	-0.303176
C	-1.288249	3.298479	-0.757620
C	0.066616	3.681192	-0.728828
C	-2.222473	4.206866	-1.295577
C	0.479095	4.934110	-1.201295
H	0.822717	2.982707	-0.375536
C	-1.809694	5.457407	-1.775758
H	-3.279916	3.939897	-1.349644
C	-0.459618	5.831130	-1.727793
H	1.542080	5.196343	-1.162390
H	-2.555311	6.142360	-2.193934
H	-0.142106	6.809643	-2.103855
C	-3.626885	1.768829	-0.381471
C	-4.058607	1.377929	-1.666635
C	-4.551179	2.334653	0.515469
C	-5.400586	1.538839	-2.031927
H	-3.321680	0.988010	-2.372521
C	-5.889981	2.507857	0.136985
H	-4.244785	2.632648	1.520710
C	-6.319824	2.105534	-1.133848
H	-5.726816	1.232687	-3.031776

H	-6.599312	2.946100	0.846957
H	-7.367369	2.235795	-1.426742
O	-1.307114	1.416149	-2.984337
C	-0.088121	1.428381	-2.748108
O	0.476120	0.949531	-1.679613
C	0.898160	2.055634	-3.726893
H	1.278083	2.997966	-3.294201
H	1.759152	1.384807	-3.886303
H	0.396506	2.275038	-4.680891
H	-0.929413	-0.713629	-2.743233
C	-0.457893	-2.723638	-2.032683
C	-0.874425	-3.826349	-1.254526
C	0.581900	-2.921652	-2.970050
C	-0.263605	-5.076602	-1.395748
H	-1.679571	-3.679311	-0.528885
C	1.197659	-4.170950	-3.106841
H	0.911149	-2.073112	-3.578876
C	0.782104	-5.254854	-2.316091
H	-0.604215	-5.918092	-0.782250
H	2.003859	-4.303206	-3.837729
H	1.260772	-6.234180	-2.425714
H	10.990135	0.982613	-0.880371

[1]TS(4-5)
Lowest frequency = -308.8846 cm^{-1}
Charge = 0, Multiplicity = 1

126

P	0.824840	1.143815	1.285360
P	-1.838535	1.654132	-0.049941
C	-0.187690	2.541541	2.013124
H	0.000683	2.633577	3.096095
H	0.172800	3.466616	1.533302
C	-1.671155	2.321402	1.700795
H	-2.231236	3.265993	1.804723
H	-2.119886	1.569126	2.370870
C	-3.962194	-1.077790	0.980670
C	-3.171588	-0.800132	2.134960
C	-5.366267	-0.887561	1.081951
C	-3.786122	-0.295434	3.302421
C	-5.949503	-0.364955	2.231581
H	-5.996958	-1.109486	0.217518
C	-5.154110	-0.037605	3.348258
H	-3.156042	-0.150350	4.185632
H	-7.031539	-0.196935	2.259684
H	-5.610635	0.378097	4.252694
C	-1.802931	-1.353652	2.292058
N	-0.975030	-1.485819	1.180290
C	0.052295	-2.530941	1.434642
H	0.367689	-2.437858	2.489097
H	-0.393310	-3.536867	1.325895
C	1.208685	-2.341602	0.527030
C	2.399495	-3.005564	0.287301
N	1.222437	-1.225584	-0.258960
N	2.331159	-1.143468	-0.959232
N	3.048429	-2.226273	-0.629285
H	2.820173	-3.930338	0.673926
O	-1.474828	-1.808255	3.405779
Fe	-0.369830	-0.084611	-0.231523
C	1.391842	0.177900	2.743551
C	2.608575	-0.532339	2.649687
C	0.630229	0.076764	3.920214
C	3.042532	-1.336273	3.708364
H	3.220115	-0.439942	1.746473
C	1.070300	-0.723930	4.983270
H	-0.323765	0.602625	4.010183
C	2.270944	-1.436448	4.878107
H	3.988255	-1.883036	3.623542
H	0.454210	-0.808340	5.883359
H	2.608427	-2.068690	5.706193
C	2.431628	1.952987	0.846998
C	3.160473	2.636633	1.843426
C	2.949736	1.872222	-0.459500
C	4.389396	3.232894	1.536631
H	2.772578	2.686998	2.867024
C	4.184769	2.469473	-0.759348
H	2.370561	1.349874	-1.227198
C	4.906451	3.146717	0.233291
H	4.948071	3.761826	2.316317
H	4.581707	2.402722	-1.778457
H	5.870383	3.609942	-0.004795
C	-3.353790	-1.562139	-0.243119
C	-2.017957	-1.456052	-0.490871
C	-4.212034	-2.220732	-1.313902
C	-4.757643	-3.602062	-0.900557
H	-3.613049	-2.327037	-2.233869
H	-5.058534	-1.555530	-1.574262
C	-5.654850	-4.226111	-1.974719
H	-3.903046	-4.271379	-0.688801
H	-5.316134	-3.508280	0.049396
H	-6.028144	-5.218098	-1.664305
H	-6.532112	-3.585259	-2.180947
H	-5.106295	-4.353288	-2.925981
C	-1.177068	-1.532100	-1.601516
C	4.416842	-2.327137	-1.136661
H	4.736001	-3.375198	-1.019525
H	4.375622	-2.096643	-2.213558
C	5.340355	-1.349165	-0.397744
C	6.749243	-1.267238	-0.995134
H	5.395339	-1.641809	0.668067
H	4.867050	-0.351366	-0.428960
C	7.629090	-0.238655	-0.270509
H	7.235933	-2.262307	-0.963188
H	6.674788	-0.994570	-2.066493
C	9.032531	-0.092284	-0.874292
H	7.117493	0.743891	-0.283009
H	7.715658	-0.520675	0.797725
H	9.542029	-1.075651	-0.856152
H	8.939930	0.183204	-1.943147
C	9.894112	0.947526	-0.148769
H	10.031188	0.679016	0.914664
H	9.421691	1.946465	-0.180611
C	-1.378703	3.214008	-0.962179
C	-0.029273	3.608504	-1.044824
C	-2.356546	4.062286	-1.519031
C	0.336653	4.818374	-1.649492
H	0.753615	2.950430	-0.670520
C	-1.990344	5.268967	-2.131230
H	-3.411517	3.780637	-1.482947
C	-0.644533	5.656035	-2.196846
H	1.395793	5.094483	-1.696691
H	-2.767898	5.909226	-2.562001
H	-0.363181	6.600097	-2.675802
C	-3.651676	1.657093	-0.334608
C	-4.124065	1.165274	-1.569240
C	-4.560182	2.231804	0.573107
C	-5.486563	1.249732	-1.881422
H	-3.403759	0.760214	-2.285211
C	-5.920853	2.324530	0.248850
H	-4.221551	2.603577	1.542934

C	-6.388296	1.832392	-0.976403
H	-5.843637	0.869142	-2.844524
H	-6.617314	2.771930	0.965814
H	-7.452408	1.902363	-1.227097
O	-1.412299	1.109407	-3.049136
C	-0.183746	1.160652	-2.869053
O	0.442173	0.796785	-1.789530
C	0.736335	1.723480	-3.947124
H	1.700522	1.189412	-3.963976
H	0.242861	1.668140	-4.929218
H	0.934443	2.785104	-3.713678
H	-1.452403	-0.851118	-2.418872
C	-0.400475	-2.726452	-1.982449
C	-0.668006	-3.989748	-1.407008
C	0.614705	-2.647040	-2.963677
C	0.071099	-5.122135	-1.768065
H	-1.479146	-4.069464	-0.675173
C	1.356352	-3.778068	-3.322962
H	0.831892	-1.676597	-3.420110
C	1.097232	-5.020932	-2.720663
H	-0.159284	-6.090701	-1.309853
H	2.143008	-3.691839	-4.081705
H	1.676450	-5.905822	-3.006586
H	10.895071	1.033429	-0.606434

^{1}I-5

Lowest frequency = 7.4049 cm^{-1}

Charge = 0, Multiplicity = 1

126

P	-0.825127	-1.404758	1.142607
P	1.868211	-1.607626	-0.240918
C	0.253533	-2.841583	1.671574
H	0.073226	-3.114080	2.724742
H	-0.052311	-3.699477	1.048774
C	1.725683	-2.497454	1.416469
H	2.344509	-3.411230	1.406577
H	2.107534	-1.822379	2.200177
C	4.076593	1.257807	1.224185
C	3.344069	0.654334	2.293195
C	5.494870	1.301845	1.348430
C	4.016696	0.086654	3.401680
C	6.138604	0.745757	2.446397
H	6.088417	1.755466	0.551092
C	5.403742	0.118099	3.480369
H	3.406252	-0.351054	4.197275
H	7.232072	0.783779	2.504309
H	5.922093	-0.320171	4.339189
C	1.892251	0.732759	2.357120
N	1.201867	1.358262	1.240875
C	0.159679	2.319792	1.750137
H	-0.045085	1.984714	2.780430
H	0.582932	3.338218	1.769555
C	-1.085582	2.249298	0.938847
C	-2.248256	2.994389	0.853670
N	-1.208848	1.225740	0.043087
N	-2.357959	1.282135	-0.588854
N	-2.994407	2.356419	-0.096068
H	-2.587022	3.896739	1.356444
O	1.235640	0.387971	3.343125
Fe	0.327775	0.057569	-0.175898
C	-1.603118	-0.756972	2.691181
C	-2.785282	0.000814	2.551105
C	-1.093241	-0.981390	3.982088
C	-3.427239	0.540252	3.671769
H	-3.216498	0.145896	1.555920
C	-1.743785	-0.452120	5.104925
H	-0.169530	-1.547219	4.119984
C	-2.907024	0.316292	4.955474
H	-4.344535	1.125905	3.542542
H	-1.331126	-0.635781	6.102647
H	-3.409975	0.731565	5.835426
C	-2.350442	-2.242970	0.492212
C	-2.978516	-3.239291	1.268443
C	-2.921761	-1.859477	-0.735392
C	-4.155184	-3.850481	0.819757
H	-2.554393	-3.526080	2.237362
C	-4.107666	-2.469795	-1.175958
H	-2.418736	-1.102240	-1.342848
C	-4.725908	-3.462762	-0.403875
H	-4.633643	-4.625064	1.429037
H	-4.544981	-2.167424	-2.134295
H	-5.649948	-3.937392	-0.752170
C	3.370921	1.736035	0.071437
C	1.981481	1.689651	0.036779
C	4.130569	2.294124	-1.113238
C	4.501782	3.783541	-0.948160
H	3.526995	2.180373	-2.028065
H	5.049947	1.705769	-1.281041
C	5.310412	4.321675	-2.133080
H	3.571873	4.369193	-0.831681
H	5.071474	3.918729	-0.008847
H	5.561280	5.389147	-2.001589
H	6.257781	3.764423	-2.254685
H	4.743001	4.220867	-3.076143
C	1.175405	1.708475	-1.177189
C	-4.351321	2.634324	-0.569512
H	-4.639414	3.618592	-0.167175
H	-4.301542	2.714557	-1.668382
C	-5.320699	1.526560	-0.138930
C	-6.723790	1.684851	-0.735085
H	-5.377579	1.507511	0.965687
H	-4.889726	0.558199	-0.450535
C	-7.662521	0.539197	-0.331139
H	-7.162849	2.652658	-0.420643
H	-6.648943	1.725065	-1.839794
C	-9.067238	0.650350	-0.939740
H	-7.205121	-0.424475	-0.630786
H	-7.741995	0.505273	0.773685
H	-9.519701	1.615138	-0.637190
H	-8.982019	0.686678	-2.043509
C	-9.993003	-0.501946	-0.533516
H	-10.122705	-0.540570	0.563515
H	-9.579101	-1.475704	-0.853315
C	1.503557	-3.072027	-1.327943
C	0.166809	-3.438316	-1.576325
C	2.531681	-3.890277	-1.836112
C	-0.138776	-4.591847	-2.310954
H	-0.643696	-2.801517	-1.221680
C	2.225163	-5.040449	-2.576837
H	3.578399	-3.628320	-1.658683
C	0.890540	-5.397774	-2.816803
H	-1.188245	-4.851360	-2.488479
H	3.038864	-5.660650	-2.968754
H	0.655609	-6.297362	-3.396050
C	3.687849	-1.464395	-0.467897
C	4.165499	-0.961822	-1.696132
C	4.609735	-1.899128	0.504025
C	5.543381	-0.906460	-1.942668

H	3.439587	-0.648754	-2.452420
C	5.986564	-1.839264	0.250705
H	4.266440	-2.277696	1.469401
C	6.457970	-1.344315	-0.972586
H	5.903712	-0.520170	-2.902447
H	6.690843	-2.173143	1.019749
H	7.534737	-1.298746	-1.169150
O	1.441481	-0.659505	-3.068592
C	0.206734	-0.712691	-2.953404
O	-0.472097	-0.517780	-1.860500
C	-0.661336	-1.085375	-4.151649
H	-0.743950	-2.187082	-4.183441
H	-1.675380	-0.663084	-4.065432
H	-0.177664	-0.751416	-5.082727
H	1.713154	1.297245	-2.042173
C	0.344905	2.880002	-1.554564
C	0.531183	4.145109	-0.948860
C	-0.637427	2.784582	-2.569357
C	-0.246041	5.254007	-1.308258
H	1.319858	4.254978	-0.195437
C	-1.414359	3.890523	-2.930820
H	-0.808167	1.815626	-3.045118
C	-1.233504	5.132113	-2.296848
H	-0.070959	6.220643	-0.821660
H	-2.171533	3.783761	-3.716693
H	-1.841180	5.996912	-2.585171
H	-10.993601	-0.395392	-0.987700

[3]I-0

Lowest frequency = 8.5255 cm^{-1}

Charge = 0, Multiplicity = 3

105

P	0.042644	-0.645967	-1.083689
P	1.824982	1.509078	-0.232118
C	1.342300	-0.335008	-2.379595
H	2.039639	-1.187175	-2.318974
H	0.919759	-0.307277	-3.398459
C	2.087292	0.969412	-2.027862
H	1.805521	1.795198	-2.699943
H	3.174195	0.828894	-2.132403
C	3.722287	-1.395214	-0.116715
C	3.236203	-2.703330	-0.287706
C	4.766068	-0.910473	-0.917735
C	3.822492	-3.525595	-1.268749
C	5.314077	-1.722205	-1.922811
H	5.156016	0.099856	-0.754946
C	4.840220	-3.033434	-2.095483
H	3.452698	-4.552714	-1.352200
H	6.121360	-1.339669	-2.557089
H	5.275714	-3.675086	-2.869711
C	2.105177	-3.274358	0.526905
N	1.239248	-2.374135	1.049962
C	0.120308	-3.024606	1.724261
H	-0.281790	-3.849023	1.101106
H	0.457080	-3.512078	2.664210
C	-0.956216	-2.027932	2.031708
C	-2.250263	-2.159299	2.510734
N	-0.749554	-0.684124	1.837584
N	-1.811915	0.009659	2.181700
N	-2.731548	-0.881818	2.592156
H	-2.848404	-3.027514	2.778942
O	1.975895	-4.515350	0.619789
Fe	1.119428	-0.333605	0.973735
C	-0.657113	-2.303994	-1.424714
C	-2.004334	-2.549805	-1.081742
C	0.151875	-3.381228	-1.833537
C	-2.524732	-3.848379	-1.140097
H	-2.643850	-1.718332	-0.766718
C	-0.368121	-4.680445	-1.878990
H	1.201571	-3.217349	-2.090574
C	-1.705111	-4.919046	-1.530023
H	-3.572999	-4.025972	-0.874305
H	0.283836	-5.509975	-2.168739
H	-2.107277	-5.937243	-1.560169
C	-1.364378	0.427707	-1.625546
C	-1.851880	0.394312	-2.948019
C	-1.985102	1.271706	-0.688103
C	-2.935892	1.198051	-3.324267
H	-1.392047	-0.275436	-3.683655
C	-3.079977	2.064852	-1.060650
H	-1.608032	1.306238	0.335388
C	-3.554455	2.033342	-2.379002
H	-3.305781	1.167438	-4.355027
H	-3.547872	2.721128	-0.319498
H	-4.403784	2.659301	-2.674331
C	-4.109440	-0.426893	2.783164
H	-4.591913	-1.095719	3.514966
H	-4.045497	0.581525	3.222146
C	-4.859443	-0.397170	1.443464
C	-6.242792	0.256769	1.529812
H	-4.949786	-1.430259	1.057428
H	-4.234774	0.156041	0.719614
C	-6.937046	0.332592	0.162458
H	-6.883639	-0.298591	2.243152
H	-6.136681	1.279651	1.942629
C	-8.307033	1.023495	0.203389
H	-6.275398	0.867139	-0.547523
H	-7.053625	-0.691381	-0.244878
H	-8.966658	0.483969	0.910997
H	-8.185860	2.043781	0.617411
C	-8.981870	1.103718	-1.170677
H	-9.146132	0.095602	-1.593059
H	-8.356382	1.666050	-1.887995
C	0.635659	2.926357	-0.350517
C	0.138029	3.461597	-1.553049
C	0.195448	3.480530	0.869664
C	-0.774886	4.525451	-1.538018
H	0.432992	3.038459	-2.517072
C	-0.715996	4.542416	0.884574
H	0.565624	3.066980	1.812448
C	-1.205787	5.069168	-0.320984
H	-1.160131	4.918716	-2.484589
H	-1.046969	4.958109	1.842382
H	-1.923597	5.896321	-0.310359
C	3.384252	2.472904	0.040213
C	4.348702	1.990233	0.939754
C	3.637327	3.669401	-0.661255
C	5.554811	2.679879	1.131451
H	4.145486	1.070576	1.492577
C	4.843507	4.356111	-0.473841
H	2.883442	4.070313	-1.347457
C	5.805276	3.862627	0.422609
H	6.295364	2.291330	1.838581
H	5.031993	5.284091	-1.024671
H	6.745265	4.404729	0.571557
H	-9.962223	1.607802	-1.110598
C	1.917389	-0.192301	2.866140
C	1.722072	1.214179	3.434279
C	3.288727	-0.787587	3.183659

H	1.179385	-0.855698	3.360198
H	0.674901	1.552241	3.319874
H	1.953312	1.253818	4.522105
H	2.376097	1.959114	2.942491
H	3.375661	-1.817086	2.796868
H	4.133137	-0.205001	2.770626
H	3.456322	-0.827540	4.282106
H	3.298632	-0.776549	0.685710

^{3}TS(0-1)
Lowest frequency = -1275.0332 cm^{-1}
Charge = 0, Multiplicity = 3

105

P	1.074420	-1.189380	-1.135160
P	0.117314	1.847337	-0.707674
C	0.673750	-0.180257	-2.661886
H	1.334257	-0.475865	-3.495059
H	-0.345907	-0.482010	-2.949281
C	0.751242	1.330564	-2.399240
H	0.224232	1.903450	-3.183674
H	1.804287	1.660951	-2.399531
C	3.128303	0.521838	0.477013
C	3.904780	-0.517309	1.078263
C	3.806807	1.419616	-0.378852
C	5.278437	-0.631126	0.836187
C	5.180564	1.290486	-0.635183
H	3.262693	2.245928	-0.848392
C	5.923958	0.271472	-0.020507
H	5.811051	-1.457852	1.318786
H	5.671820	1.997586	-1.313866
H	6.997297	0.176055	-0.218173
C	3.195603	-1.562213	1.895866
N	1.851677	-1.397252	1.765826
C	0.943584	-2.311513	2.423451
H	0.950794	-3.324647	1.962295
H	1.211400	-2.469716	3.488979
C	-0.425947	-1.697169	2.296594
C	-1.688339	-1.991238	2.788461
N	-0.559901	-0.534590	1.567985
N	-1.801411	-0.102263	1.593832
N	-2.491021	-0.980230	2.335126
H	-2.066403	-2.801152	3.408941
O	3.770036	-2.461715	2.534180
Fe	1.127707	0.214992	0.814460
C	2.671967	-2.003814	-1.464768
C	3.046822	-3.115546	-0.677838
C	3.620687	-1.444713	-2.343778
C	4.327582	-3.666396	-0.786681
H	2.345664	-3.533463	0.049064
C	4.901872	-1.999207	-2.450687
H	3.374516	-0.555831	-2.932597
C	5.259020	-3.111952	-1.675906
H	4.603360	-4.511521	-0.148688
H	5.630524	-1.545076	-3.130243
H	6.266988	-3.533187	-1.751011
C	-0.185504	-2.522206	-1.282031
C	0.093006	-3.846125	-1.679667
C	-1.527179	-2.159531	-1.020588
C	-0.941259	-4.783898	-1.804037
H	1.120264	-4.141459	-1.910790
C	-2.558909	-3.093870	-1.170738
H	-1.762378	-1.136530	-0.708056
C	-2.270759	-4.413051	-1.553058
H	-0.705819	-5.807290	-2.116154
H	-3.593913	-2.788688	-0.987611
H	-3.077839	-5.145593	-1.660679
C	-3.932948	-0.770984	2.494644
H	-4.262881	-1.390006	3.344495
H	-4.069519	0.289399	2.763317
C	-4.698534	-1.101161	1.209145
C	-6.166006	-0.663408	1.260689
H	-4.627688	-2.187215	1.015808
H	-4.193627	-0.583905	0.374398
C	-6.924445	-1.004121	-0.029289
H	-6.676476	-1.132769	2.125201
H	-6.208032	0.430304	1.430726
C	-8.371108	-0.491688	-0.045575
H	-6.374988	-0.578097	-0.891530
H	-6.920372	-2.102414	-0.177639
H	-8.916827	-0.912619	0.821480
H	-8.365901	0.606272	0.101856
C	-9.118124	-0.832574	-1.339902
H	-9.167588	-1.925841	-1.495044
H	-8.610261	-0.394949	-2.218842
C	-1.704505	1.957918	-0.927984
C	-2.425858	1.211890	-1.886450
C	-2.439515	2.734215	0.000168
C	-3.829375	1.229483	-1.905335
H	-1.899154	0.618334	-2.637290
C	-3.835280	2.763550	-0.029867
H	-1.902880	3.324029	0.749905
C	-4.542641	2.003304	-0.979878
H	-4.364242	0.643246	-2.660993
H	-4.378858	3.384350	0.691065
H	-5.637367	2.024209	-1.002277
C	0.577885	3.636917	-0.766172
C	1.658028	4.105315	0.006911
C	-0.112854	4.548696	-1.592790
C	2.048782	5.450578	-0.051389
H	2.188775	3.413354	0.665958
C	0.275168	5.893289	-1.647461
H	-0.963349	4.201800	-2.189673
C	1.358639	6.347662	-0.878524
H	2.889861	5.796638	0.558713
H	-0.269703	6.590001	-2.293935
H	1.660211	7.399702	-0.921894
H	-10.152668	-0.447716	-1.322528
C	1.323073	1.502325	2.622066
C	0.155536	2.480047	2.682624
C	2.607914	2.088997	3.219195
H	1.076277	0.592709	3.194348
H	-0.782980	2.020933	2.330400
H	-0.006051	2.821115	3.728200
H	0.341546	3.386092	2.080303
H	3.429738	1.352712	3.228864
H	2.963360	2.975314	2.658947
H	2.435177	2.417169	4.264797
H	2.273950	1.190126	1.331033

^{3}TS(0-1')
Lowest frequency = -1128.0425 cm^{-1}
Charge = 0, Multiplicity = 3

105

N	-0.890835	-0.430393	1.292337
C	-0.757399	-1.572111	2.044735
C	-2.016850	-1.871335	2.539901

N	-2.825557	-0.878405	2.057899
N	-2.138838	-0.009914	1.300451
C	0.592439	-2.201893	2.166613
N	1.594883	-1.288167	1.619578
C	2.847447	-1.532074	2.090402
O	3.106068	-2.396607	2.956740
C	-4.265375	-0.667588	2.224946
C	-5.022026	-0.880200	0.909338
C	-6.490269	-0.449376	0.994057
C	-7.232468	-0.611271	-0.339318
C	-8.695473	-0.149370	-0.288898
C	-9.425682	-0.307031	-1.627383
Fe	0.867451	0.116048	0.418440
C	3.846305	0.115256	0.383595
C	3.979321	-0.724277	1.502427
C	5.258493	-0.899094	2.063725
C	6.372787	-0.246115	1.523087
C	6.227847	0.581710	0.398515
C	4.958294	0.757937	-0.172420
P	0.202075	1.763638	-0.847574
C	0.420737	1.334045	-2.673630
C	0.169235	-0.159328	-2.894259
P	0.849871	-1.118156	-1.434405
C	2.498741	-1.733235	-1.937932
C	3.166784	-2.623071	-1.066280
C	4.512237	-2.945294	-1.272448
C	5.219407	-2.371694	-2.339233
C	4.567461	-1.484405	-3.207356
C	3.216348	-1.167071	-3.010248
C	1.034857	3.417753	-0.839468
C	2.388646	3.475773	-0.457422
C	3.091872	4.687132	-0.500825
C	2.444835	5.859197	-0.919620
C	1.095067	5.811592	-1.300428
C	0.393542	4.598102	-1.264469
C	-1.570261	2.248337	-0.699466
C	-2.565990	1.791969	-1.582928
C	-3.921398	2.046645	-1.324246
C	-4.298632	2.763434	-0.179827
C	-3.311056	3.228967	0.703686
C	-1.961183	2.966967	0.449426
C	-0.246365	-2.597253	-1.407258
C	0.190588	-3.917007	-1.628862
C	-0.713272	-4.986400	-1.544300
C	-2.064123	-4.751730	-1.251675
C	-2.510367	-3.436880	-1.044747
C	-1.609075	-2.368601	-1.111957
C	0.673825	1.712290	3.482320
C	1.647207	1.980676	2.511011
C	0.705415	0.534702	4.402416
H	0.585076	-0.529416	-3.847849
H	-0.911179	-0.382851	-2.904779
H	-0.212120	1.975411	-3.313068
H	1.473891	1.587862	-2.889317
H	4.835742	1.386243	-1.061422
H	5.339994	-1.573019	2.922209
H	7.100834	1.081033	-0.036558
H	7.361490	-0.388904	1.973605
H	0.596622	-3.169843	1.618054
H	0.832575	-2.451336	3.217134
H	-2.388777	-2.670955	3.176990
H	2.639933	-3.032703	-0.199205
H	2.729748	-0.459455	-3.689553
H	5.016231	-3.622300	-0.575478
H	5.114314	-1.029547	-4.040475
H	6.278815	-2.605888	-2.486053
H	1.238174	-4.110127	-1.877548
H	-1.958854	-1.346426	-0.926796
H	-0.358389	-6.008172	-1.717371
H	-3.565678	-3.242014	-0.825951
H	-2.767580	-5.588874	-1.189464
H	-4.607977	-1.353872	3.016033
H	-4.400862	0.367648	2.580815
H	-4.950095	-1.944129	0.616224
H	-4.507247	-0.289600	0.131523
H	-7.012725	-1.028866	1.781085
H	-6.536336	0.611629	1.309662
H	-6.694124	-0.040712	-1.122031
H	-7.192474	-1.672833	-0.655059
H	-9.229669	-0.720092	0.495766
H	-8.728882	0.910983	0.029799
H	-9.436794	-1.363287	-1.952924
H	-8.930550	0.280409	-2.422153
H	-2.292057	1.231482	-2.482286
H	-1.196149	3.311279	1.154177
H	-4.684524	1.689221	-2.024804
H	-3.596448	3.793770	1.598099
H	-5.357040	2.964911	0.018897
H	2.885885	2.563262	-0.114247
H	-0.659866	4.567739	-1.562814
H	4.143785	4.713975	-0.196592
H	0.585157	6.724365	-1.627942
H	2.989411	6.809256	-0.945685
H	-10.472987	0.035207	-1.558610
H	-0.239405	2.324821	3.488651
H	1.448409	1.422068	1.251012
H	1.676438	3.001355	2.102338
H	2.642527	1.536003	2.660391
H	0.579716	0.845720	5.460030
H	-0.125974	-0.172306	4.196156
H	1.649432	-0.029188	4.318874
H	2.856694	0.259913	-0.086784

^{3}I-1'

Lowest frequency = 9.4264 cm^{-1}

Charge = 0, Multiplicity = 3

96

P	0.492383	-0.917226	-1.126595
P	1.230947	1.914794	-0.358019
C	0.737886	0.304459	-2.525127
H	1.039694	-0.209394	-3.454692
H	-0.257408	0.749129	-2.703777
C	1.725098	1.389967	-2.089958
H	1.727578	2.236071	-2.799929
H	2.752462	0.990879	-2.006886
C	3.657474	-0.159665	0.956671
C	3.627286	-1.534555	1.258034
C	4.722245	0.386264	0.225686
C	4.698658	-2.346087	0.848025
C	5.764273	-0.442172	-0.218966
H	4.748266	1.461027	0.022790
C	5.754972	-1.808328	0.102775
H	4.665024	-3.403842	1.123990
H	6.593992	-0.016616	-0.794704
H	6.574534	-2.455400	-0.229491
C	2.483342	-2.186435	1.988637
N	1.300960	-1.513005	1.951851
C	0.226874	-2.232133	2.621171
H	0.127238	-3.265325	2.221006

H	0.445292	-2.372958	3.699493
C	-1.042191	-1.462117	2.436732
C	-2.321109	-1.591073	2.953502
N	-1.051153	-0.358554	1.621539
N	-2.244552	0.194223	1.613145
N	-3.022600	-0.545553	2.421160
H	-2.771763	-2.306768	3.638270
O	2.629791	-3.307626	2.517766
Fe	0.861812	0.110662	0.865066
C	1.686788	-2.270398	-1.431784
C	1.584975	-3.433172	-0.638972
C	2.761707	-2.151950	-2.332877
C	2.518674	-4.466437	-0.766997
H	0.779672	-3.522428	0.095574
C	3.699412	-3.186124	-2.455383
H	2.881838	-1.248710	-2.938487
C	3.576802	-4.346928	-1.679721
H	2.432532	-5.350235	-0.127874
H	4.535855	-3.076776	-3.153557
H	4.317462	-5.148426	-1.770375
C	-1.147224	-1.635325	-1.570251
C	-1.344008	-2.960686	-2.002801
C	-2.261210	-0.772894	-1.480362
C	-2.629027	-3.414879	-2.334429
H	-0.489789	-3.638178	-2.092462
C	-3.538334	-1.223107	-1.835838
H	-2.123553	0.256196	-1.132674
C	-3.728540	-2.548464	-2.256399
H	-2.766602	-4.448755	-2.669507
H	-4.390050	-0.537112	-1.782707
H	-4.729320	-2.901783	-2.527701
C	-4.435403	-0.197041	2.584283
H	-4.848263	-0.885883	3.338990
H	-4.488075	0.827167	2.992698
C	-5.194263	-0.296128	1.257662
C	-6.681490	0.050831	1.386142
H	-5.073153	-1.317701	0.853312
H	-4.707718	0.384380	0.537220
C	-7.418803	-0.019962	0.041417
H	-7.165966	-0.637077	2.107410
H	-6.790545	1.068359	1.811219
C	-8.912950	0.318730	0.137566
H	-6.931639	0.670884	-0.675551
H	-7.298899	-1.035464	-0.386036
H	-9.395746	-0.372714	0.855618
H	-9.027627	1.333211	0.566999
C	-9.636287	0.246488	-1.211991
H	-9.565411	-0.766865	-1.647796
H	-9.194393	0.952516	-1.938661
C	-0.156284	3.089993	-0.700112
C	-0.000129	4.253116	-1.479405
C	-1.421078	2.767037	-0.181323
C	-1.105059	5.070879	-1.749655
H	0.987040	4.529280	-1.866461
C	-2.525135	3.589465	-0.450587
H	-1.529831	1.871009	0.436289
C	-2.370698	4.738762	-1.237924
H	-0.979148	5.973892	-2.357117
H	-3.506297	3.328521	-0.037879
H	-3.231590	5.382311	-1.449857
C	2.594260	3.058503	0.131525
C	2.804462	3.309067	1.501457
C	3.447377	3.667862	-0.812042
C	3.843209	4.150230	1.919608
H	2.151254	2.815083	2.229860
C	4.487986	4.508171	-0.391262
H	3.316940	3.478820	-1.881903
C	4.689156	4.750366	0.975267
H	3.998697	4.328953	2.988827
H	5.147290	4.969371	-1.134597
H	5.507228	5.400530	1.303114
H	-10.707045	0.494829	-1.109623
H	2.876818	0.503528	1.367085
H	0.846979	1.308162	1.910113

^{3}TS(1'-1)
Lowest frequency = -1357.4715 cm^{-1}
Charge = 0, Multiplicity = 3

96

P	1.273017	-0.927992	-1.046662
P	0.103463	1.950899	-0.430197
C	0.882561	0.178037	-2.511587
H	1.600940	-0.014257	-3.326616
H	-0.102488	-0.158738	-2.873095
C	0.849692	1.665319	-2.127691
H	0.322037	2.265894	-2.890487
H	1.877247	2.058283	-2.045625
C	3.192594	0.724374	0.725493
C	3.987292	-0.320924	1.280524
C	3.846683	1.718865	-0.034570
C	5.368238	-0.374057	1.054034
C	5.228072	1.652022	-0.273719
H	3.277475	2.567825	-0.430875
C	5.992258	0.607126	0.271355
H	5.924198	-1.210221	1.491682
H	5.710893	2.427383	-0.880020
H	7.070612	0.560074	0.083555
C	3.294986	-1.412286	2.046443
N	1.944872	-1.240640	1.964016
C	1.067774	-2.215416	2.575746
H	1.138195	-3.211825	2.085562
H	1.319512	-2.392264	3.641739
C	-0.324482	-1.662525	2.435886
C	-1.582075	-2.014986	2.901564
N	-0.494022	-0.500424	1.717551
N	-1.750323	-0.117897	1.726623
N	-2.416060	-1.031167	2.443484
H	-1.938535	-2.846297	3.506414
O	3.881292	-2.348453	2.617201
Fe	1.175086	0.313339	0.962970
C	2.887960	-1.702829	-1.372744
C	3.250951	-2.843729	-0.622078
C	3.858775	-1.088881	-2.188965
C	4.543601	-3.370013	-0.707513
H	2.529641	-3.303430	0.058981
C	5.151212	-1.620037	-2.273048
H	3.619367	-0.176946	-2.744456
C	5.497513	-2.761900	-1.535826
H	4.810290	-4.238113	-0.097205
H	5.896995	-1.125841	-2.904369
H	6.514217	-3.164600	-1.592189
C	0.031065	-2.260991	-1.293827
C	0.332478	-3.555368	-1.764232
C	-1.319670	-1.920647	-1.050977
C	-0.690969	-4.489241	-1.976481
H	1.368516	-3.829448	-1.982283
C	-2.339446	-2.850071	-1.288169
H	-1.571312	-0.920254	-0.683197
C	-2.029895	-4.141250	-1.742533

H	-0.439343	-5.490311	-2.343408
H	-3.381508	-2.561838	-1.118786
H	-2.828137	-4.869840	-1.920232
C	-3.870120	-0.886431	2.567713
H	-4.199297	-1.556768	3.377941
H	-4.060415	0.154272	2.877596
C	-4.577327	-1.190043	1.242664
C	-6.066234	-0.829422	1.261558
H	-4.443384	-2.260285	1.000197
H	-4.072883	-0.607489	0.451883
C	-6.760259	-1.151454	-0.068976
H	-6.580780	-1.361359	2.086545
H	-6.170595	0.252436	1.475190
C	-8.228152	-0.706394	-0.121189
H	-6.200356	-0.664799	-0.891351
H	-6.698116	-2.241452	-0.259840
H	-8.785118	-1.186792	0.706972
H	-8.280675	0.383797	0.068510
C	-8.909290	-1.028738	-1.456000
H	-8.900449	-2.115936	-1.655264
H	-8.390374	-0.532769	-2.296668
C	-1.703304	2.024099	-0.739114
C	-2.345697	1.404904	-1.835916
C	-2.514306	2.611223	0.263622
C	-3.746117	1.371634	-1.923181
H	-1.757697	0.952352	-2.639060
C	-3.906237	2.594367	0.164622
H	-2.036896	3.084004	1.128951
C	-4.534926	1.967611	-0.928939
H	-4.220084	0.889003	-2.785294
H	-4.510879	3.071430	0.944303
H	-5.627266	1.952733	-1.005541
C	0.473708	3.744442	-0.187942
C	1.352001	4.154919	0.831829
C	-0.102116	4.720369	-1.029762
C	1.661913	5.512687	1.000068
H	1.778662	3.405131	1.504113
C	0.208990	6.074913	-0.861806
H	-0.805089	4.413555	-1.812240
C	1.095123	6.473931	0.152267
H	2.345274	5.817107	1.800000
H	-0.242410	6.823242	-1.522432
H	1.337525	7.534112	0.283075
H	-9.961029	-0.693301	-1.464393
H	2.258068	1.314885	1.608187
H	1.235498	1.516498	2.048260

^{3}I-1

Lowest frequency = 13.9253 cm^{-1}

Charge = 0, Multiplicity = 3

107

P	0.989260	-1.553826	-1.151364
P	0.135285	1.469622	-1.264754
C	1.130889	-0.680872	-2.791874
H	2.151583	-0.264031	-2.821530
H	1.006153	-1.381575	-3.636010
C	0.090063	0.445383	-2.842497
H	-0.917996	0.005495	-2.924761
H	0.238441	1.094052	-3.722745
C	2.985626	0.251524	-0.054807
C	3.781523	-0.529961	0.839209
C	3.682107	0.845901	-1.131391
C	5.160393	-0.713535	0.668337
C	5.066815	0.672397	-1.311905
H	3.149050	1.472533	-1.857529
C	5.812351	-0.110189	-0.415197
H	5.692701	-1.337782	1.395280
H	5.566080	1.156015	-2.161036
H	6.889324	-0.247803	-0.564866
C	3.061236	-1.173282	1.978669
N	1.731888	-0.864008	1.899899
C	0.844138	-1.442677	2.888144
H	0.856102	-2.553468	2.853283
H	1.144651	-1.184336	3.925925
C	-0.522886	-0.915514	2.580681
C	-1.778615	-1.030501	3.154833
N	-0.663762	-0.081339	1.490480
N	-1.915778	0.316681	1.371657
N	-2.592274	-0.249526	2.380504
H	-2.145575	-1.574294	4.022898
O	3.572950	-1.876624	2.868679
Fe	1.038580	0.218210	0.440327
C	2.400366	-2.702268	-1.075017
C	2.452944	-3.565008	0.041298
C	3.518598	-2.618371	-1.927106
C	3.601402	-4.316111	0.307228
H	1.598279	-3.617122	0.724268
C	4.666322	-3.375751	-1.659998
H	3.513227	-1.939631	-2.783946
C	4.714263	-4.219488	-0.541531
H	3.638561	-4.951599	1.197332
H	5.535787	-3.287657	-2.319956
H	5.622619	-4.790790	-0.322956
C	-0.470126	-2.646114	-1.415127
C	-0.381165	-3.966753	-1.902678
C	-1.741722	-2.113916	-1.109427
C	-1.540501	-4.729663	-2.096065
H	0.602101	-4.392764	-2.128963
C	-2.900909	-2.872492	-1.324868
H	-1.811660	-1.105284	-0.687369
C	-2.803395	-4.182909	-1.816732
H	-1.459195	-5.754562	-2.474969
H	-3.882882	-2.444120	-1.097389
H	-3.708002	-4.779914	-1.975591
C	-4.038820	-0.029215	2.455516
H	-4.373978	-0.384879	3.443220
H	-4.202047	1.060057	2.400273
C	-4.766820	-0.737365	1.308481
C	-6.259495	-0.398125	1.247646
H	-4.620777	-1.829336	1.403557
H	-4.279265	-0.424288	0.369251
C	-6.967232	-1.072489	0.064373
H	-6.756581	-0.694187	2.192782
H	-6.377576	0.700443	1.165961
C	-8.458809	-0.725521	-0.040499
H	-6.456235	-0.780842	-0.874988
H	-6.849546	-2.171447	0.145465
H	-8.965782	-1.016256	0.900343
H	-8.569409	0.373804	-0.119225
C	-9.153605	-1.399061	-1.229247
H	-9.087078	-2.500029	-1.157528
H	-8.686371	-1.099387	-2.185121
C	-1.608828	2.058581	-1.083360
C	-2.734605	1.404609	-1.617102
C	-1.815162	3.163039	-0.229059
C	-4.031330	1.829202	-1.289403
H	-2.617262	0.548567	-2.287461
C	-3.106807	3.581368	0.105019
H	-0.947813	3.691485	0.181298

C	-4.224503	2.909597	-0.418450
H	-4.894732	1.311870	-1.722779
H	-3.244327	4.439313	0.772522
H	-5.237693	3.238756	-0.162878
C	0.919880	3.062067	-1.783398
C	1.912469	3.627305	-0.962625
C	0.518799	3.741404	-2.951615
C	2.505392	4.849513	-1.312202
H	2.202472	3.098769	-0.051551
C	1.117535	4.957685	-3.302667
H	-0.275478	3.326477	-3.582847
C	2.114254	5.512730	-2.483319
H	3.280501	5.279443	-0.668676
H	0.802973	5.477577	-4.214225
H	2.581757	6.464291	-2.759037
H	-10.222792	-1.128693	-1.277682
O	1.380008	2.177074	1.869176
C	0.385400	2.660130	2.779039
C	2.622435	2.253401	2.591883
C	0.758840	2.042823	4.141632
H	0.432463	3.769332	2.818305
H	-0.596807	2.357689	2.388789
C	2.287390	1.782104	4.018706
H	3.350217	1.627265	2.058635
H	2.970060	3.307907	2.589965
H	0.212495	1.098951	4.297291
H	0.506404	2.718507	4.976209
H	2.521449	0.712143	4.131105
H	2.869421	2.337000	4.773119

^{3}**I-2**

Lowest frequency = 9.3196 cm^{-1}

Charge = 0, Multiplicity = 3

126

P	0.077342	1.920284	-1.492096
P	-1.080252	-1.000296	-1.948655
C	-0.206455	1.333117	-3.248487
H	-0.461858	2.183656	-3.904177
H	0.772522	0.957440	-3.584998
C	-1.272868	0.231463	-3.331488
H	-1.248161	-0.274635	-4.313289
H	-2.274472	0.669929	-3.198492
C	-2.770578	1.139528	-0.316776
C	-2.975878	2.217237	0.586001
C	-3.772128	0.949156	-1.289068
C	-4.087296	3.067529	0.508910
C	-4.883491	1.806626	-1.389467
H	-3.736753	0.091232	-1.969353
C	-5.045932	2.874116	-0.493926
H	-4.164406	3.876940	1.243695
H	-5.633375	1.625180	-2.169483
H	-5.913510	3.538535	-0.574129
C	-1.923612	2.434248	1.619725
N	-0.912145	1.541381	1.435524
C	0.289380	1.703735	2.234125
H	0.688676	2.737499	2.160458
H	0.105738	1.532664	3.313094
C	1.267147	0.714483	1.694941
C	2.602783	0.432458	1.918795
N	0.843337	-0.152191	0.717430
N	1.823336	-0.934026	0.327277
N	2.897068	-0.583086	1.054186
H	3.338798	0.869067	2.588498
O	-1.935785	3.320456	2.493777
Fe	-1.110541	0.058209	0.169683
C	-0.786780	3.520575	-1.374866
C	-0.549721	4.342305	-0.251887
C	-1.839864	3.858205	-2.249718
C	-1.325950	5.483525	-0.024583
H	0.215490	4.064860	0.478374
C	-2.617848	4.998154	-2.017418
H	-2.082460	3.215771	-3.101036
C	-2.362306	5.816087	-0.907561
H	-1.142750	6.087319	0.869083
H	-3.443234	5.234394	-2.696924
H	-2.985316	6.696475	-0.718331
C	1.878087	2.287061	-1.579259
C	2.472413	3.539191	-1.315409
C	2.723959	1.189252	-1.875132
C	3.866078	3.689251	-1.354737
H	1.842003	4.407655	-1.103778
C	4.111482	1.351656	-1.942589
H	2.290611	0.199513	-2.059247
C	4.693738	2.601990	-1.673512
H	4.306318	4.672620	-1.155425
H	4.741767	0.494274	-2.205837
H	5.780952	2.728073	-1.719184
C	-2.597942	-1.099727	0.930439
C	-1.500410	-1.410670	1.512090
C	-4.057240	-1.315087	0.810832
C	-4.541755	-2.501189	1.664364
H	-4.315814	-1.483165	-0.250453
H	-4.585881	-0.384096	1.090503
C	-6.027628	-2.803826	1.447726
H	-3.934056	-3.390418	1.415803
H	-4.348840	-2.280978	2.731786
H	-6.363548	-3.646371	2.077671
H	-6.653764	-1.926654	1.692616
H	-6.222429	-3.068862	0.392452
C	-0.959812	-2.003250	2.756077
C	4.180784	-1.246976	0.809256
H	4.278035	-2.105433	1.498205
H	4.111016	-1.643511	-0.216131
C	5.367546	-0.291569	0.955476
C	6.667438	-0.901586	0.415137
H	5.503300	-0.020496	2.019435
H	5.140534	0.641209	0.410375
C	7.866315	0.048251	0.538109
H	6.889083	-1.848581	0.946002
H	6.523505	-1.172736	-0.649483
C	9.170083	-0.534412	-0.025156
H	7.630968	0.996652	0.015240
H	8.013424	0.316402	1.603239
H	9.399577	-1.482914	0.498767
H	9.016404	-0.803172	-1.088644
C	10.361606	0.422165	0.096474
H	10.558702	0.680402	1.152956
H	10.171190	1.365870	-0.446569
C	0.393163	-1.984597	-2.492533
C	1.100270	-1.729829	-3.684434
C	0.820381	-3.049888	-1.674697
C	2.218415	-2.501469	-4.036241
H	0.783918	-0.932625	-4.361774
C	1.915347	-3.839628	-2.041644
H	0.286651	-3.270800	-0.748221
C	2.630187	-3.560252	-3.216714
H	2.756700	-2.279272	-4.964008
H	2.213333	-4.678365	-1.403576
H	3.494419	-4.171852	-3.497568

C	-2.417912	-2.235389	-2.230949
C	-2.522923	-3.313784	-1.325770
C	-3.352613	-2.129111	-3.279969
C	-3.539167	-4.264729	-1.476802
H	-1.834495	-3.401594	-0.480133
C	-4.379301	-3.075213	-3.414834
H	-3.292325	-1.305677	-3.997584
C	-4.475305	-4.145896	-2.515213
H	-3.605426	-5.094656	-0.765623
H	-5.103746	-2.973832	-4.230080
H	-5.276916	-4.884306	-2.622628
O	0.392997	-2.545932	2.599466
C	0.492202	-3.660048	1.848325
O	-0.455699	-4.199872	1.302249
C	1.925957	-4.126472	1.743436
H	1.945265	-5.210466	1.559696
H	2.382610	-3.612251	0.880911
H	2.498235	-3.871228	2.648391
H	-1.594888	-2.860430	3.052105
C	-0.896386	-0.976442	3.883570
C	-1.922842	-0.024177	4.017329
C	0.174585	-0.961215	4.794027
C	-1.875002	0.936212	5.034903
H	-2.739938	-0.005089	3.291934
C	0.218649	-0.003128	5.817941
H	0.979675	-1.692434	4.687153
C	-0.802109	0.950589	5.938794
H	-2.657436	1.698199	5.091259
H	1.061833	0.002795	6.517766
H	-0.756350	1.710813	6.725722
H	11.281661	-0.025225	-0.318158

^{3}TS(2-3)

Lowest frequency = -347.0267 cm^{-1}

Charge = 0, Multiplicity = 3

126

P	-0.476950	1.703889	-1.734884
P	-0.917635	-1.412358	-1.858578
C	-0.602445	0.909516	-3.426665
H	-1.052772	1.611311	-4.148784
H	0.435184	0.741743	-3.755323
C	-1.383708	-0.409998	-3.362123
H	-1.260301	-0.996407	-4.290240
H	-2.457781	-0.192673	-3.234032
C	-2.909346	0.687869	-0.086368
C	-3.141708	1.877746	0.676259
C	-3.901347	0.335835	-1.039738
C	-4.326942	2.613100	0.534288
C	-5.059243	1.102472	-1.208571
H	-3.791654	-0.588584	-1.617629
C	-5.293140	2.234602	-0.405962
H	-4.437294	3.511600	1.151606
H	-5.796952	0.802424	-1.962571
H	-6.209259	2.821721	-0.530699
C	-2.022699	2.419760	1.508844
N	-0.866143	1.786651	1.177809
C	0.365439	2.165231	1.833691
H	0.701273	3.185291	1.539415
H	0.262893	2.188805	2.937167
C	1.386866	1.150348	1.408979
C	2.731007	0.956753	1.677574
N	1.014103	0.143676	0.551482
N	2.030730	-0.641399	0.276197
N	3.079625	-0.152390	0.959433
H	3.437350	1.504049	2.296525
O	-2.135678	3.357962	2.322790
Fe	-0.952036	0.119293	0.041475
C	-1.561582	3.169950	-1.789854
C	-1.431245	4.178258	-0.808592
C	-2.645331	3.239686	-2.688751
C	-2.338570	5.241206	-0.753078
H	-0.646683	4.113435	-0.052494
C	-3.552374	4.304446	-2.629036
H	-2.803976	2.450012	-3.428944
C	-3.399877	5.311423	-1.665878
H	-2.228446	5.995781	0.031673
H	-4.393298	4.335154	-3.329726
H	-4.118580	6.135967	-1.613587
C	1.259516	2.314080	-1.794942
C	1.665838	3.651605	-1.620043
C	2.251808	1.331009	-2.018348
C	3.025846	3.993307	-1.658369
H	0.919930	4.437048	-1.476423
C	3.604625	1.679204	-2.078278
H	1.967611	0.280671	-2.139984
C	4.001534	3.012663	-1.887537
H	3.320505	5.039769	-1.523781
H	4.349762	0.900814	-2.277225
H	5.061525	3.286264	-1.927214
C	-2.553962	-0.861655	1.056264
C	-1.344196	-1.008282	1.537544
C	-3.910426	-1.463742	1.252908
C	-3.945126	-2.552282	2.336777
H	-4.243587	-1.888247	0.286771
H	-4.634434	-0.658709	1.482221
C	-5.310230	-3.244523	2.411276
H	-3.156710	-3.297817	2.127706
H	-3.699809	-2.099936	3.316680
H	-5.328284	-4.013928	3.203087
H	-6.115256	-2.518337	2.625723
H	-5.551881	-3.737312	1.451955
C	-0.699999	-1.508178	2.785280
C	4.379091	-0.819342	0.849897
H	4.505097	-1.500372	1.711258
H	4.310010	-1.435937	-0.060545
C	5.545640	0.167467	0.763280
C	6.870674	-0.538171	0.445363
H	5.643655	0.717557	1.718095
H	5.320089	0.919037	-0.013573
C	8.058758	0.429007	0.357463
H	7.077895	-1.306553	1.216346
H	6.770547	-1.084885	-0.513021
C	9.388016	-0.263140	0.024209
H	7.843861	1.200222	-0.408966
H	8.158842	0.973602	1.317433
H	9.596896	-1.034464	0.791161
H	9.282158	-0.807554	-0.934522
C	10.570338	0.708420	-0.063685
H	10.719545	1.241628	0.892942
H	10.402127	1.470380	-0.846535
C	0.594522	-2.320042	-2.389514
C	1.376808	-1.925906	-3.497286
C	1.062431	-3.386820	-1.588813
C	2.598857	-2.555804	-3.776911
H	1.036021	-1.127999	-4.162493
C	2.268146	-4.029279	-1.884582
H	0.474131	-3.723207	-0.730110
C	3.054221	-3.608058	-2.971443
H	3.187464	-2.228373	-4.641187

H	2.599392	-4.866311	-1.259974
H	4.003094	-4.106876	-3.195710
C	-2.183941	-2.746955	-1.843111
C	-2.237587	-3.600034	-0.720731
C	-3.119653	-2.933553	-2.883134
C	-3.192572	-4.622414	-0.649729
H	-1.550617	-3.457284	0.117727
C	-4.088606	-3.942219	-2.795976
H	-3.101220	-2.294552	-3.770529
C	-4.127356	-4.792831	-1.680897
H	-3.211250	-5.275712	0.228842
H	-4.811595	-4.068164	-3.609299
H	-4.883402	-5.582654	-1.617269
O	0.645602	-2.027949	2.561300
C	0.737127	-3.270110	2.048035
O	-0.227052	-3.958655	1.755483
C	2.178851	-3.676252	1.858664
H	2.243024	-4.769697	1.767251
H	2.538596	-3.209926	0.925760
H	2.802423	-3.316019	2.691927
H	-1.290620	-2.347713	3.194966
C	-0.573345	-0.395655	3.822786
C	-1.643188	0.494755	4.018241
C	0.596236	-0.237945	4.586991
C	-1.544606	1.535381	4.949311
H	-2.544220	0.402160	3.406239
C	0.690934	0.797869	5.528118
H	1.434520	-0.921272	4.431594
C	-0.375771	1.690064	5.708989
H	-2.368789	2.247327	5.044069
H	1.609000	0.912774	6.115493
H	-0.292325	2.510795	6.429309
H	11.509053	0.179847	-0.304520

³I-3

Lowest frequency = 9.1483 cm^{-1}

Charge = 0, Multiplicity = 3

126

P	0.419198	0.395220	2.017783
P	-1.361283	2.013413	0.191455
C	-0.936450	1.312186	2.893484
H	-1.737577	0.563454	3.012007
H	-0.641494	1.649767	3.902913
C	-1.465473	2.463384	2.025420
H	-0.891274	3.392338	2.177693
H	-2.517578	2.659297	2.285725
C	-3.740815	-0.526363	0.600602
C	-3.201020	-1.275568	1.680926
C	-4.727394	0.444228	0.893497
C	-3.695649	-1.064701	2.984270
C	-5.143277	0.706167	2.203208
H	-5.170270	1.011428	0.069289
C	-4.633270	-0.063499	3.259956
H	-3.337436	-1.728765	3.777805
H	-5.885740	1.489440	2.391808
H	-4.981172	0.099177	4.286051
C	-2.176175	-2.377982	1.534565
N	-1.024463	-2.015307	0.931331
C	-0.011571	-3.064256	0.932799
H	0.275514	-3.345708	1.965456
H	-0.412498	-3.992356	0.481604
C	1.178059	-2.584996	0.168196
C	2.409548	-3.144114	-0.136149
N	1.156812	-1.341317	-0.400474
N	2.283865	-1.095858	-1.033300
N	3.052987	-2.189788	-0.872087
H	2.858915	-4.107288	0.094307
O	-2.383656	-3.490086	2.064178
Fe	-0.673644	-0.241760	0.011837
C	0.849182	-0.961719	3.181168
C	2.153042	-1.495343	3.223422
C	-0.169368	-1.599773	3.917420
C	2.425314	-2.648775	3.971009
H	2.961302	-1.009736	2.668516
C	0.101244	-2.759059	4.653518
H	-1.189932	-1.213294	3.893866
C	1.398712	-3.291891	4.678724
H	3.444607	-3.050127	3.994407
H	-0.712732	-3.256781	5.189580
H	1.608643	-4.203605	5.247990
C	1.893853	1.498784	2.161274
C	1.919377	2.659292	2.957236
C	3.029229	1.190669	1.379278
C	3.048324	3.491291	2.971636
H	1.055099	2.924417	3.573596
C	4.165262	2.008169	1.413445
H	3.000708	0.328352	0.705919
C	4.176172	3.166496	2.205527
H	3.046100	4.396505	3.588462
H	5.038669	1.750127	0.804387
H	5.057221	3.816865	2.220193
C	-3.405673	-0.823997	-0.826891
C	-2.110106	-0.759318	-1.246595
C	-4.620724	-1.198340	-1.665318
C	-5.179721	-2.592021	-1.314461
H	-4.394010	-1.153631	-2.745844
H	-5.416274	-0.446465	-1.497373
C	-6.475429	-2.923827	-2.061477
H	-4.408510	-3.352487	-1.540145
H	-5.346414	-2.646382	-0.222707
H	-6.846939	-3.929746	-1.797553
H	-7.270860	-2.195718	-1.817229
H	-6.325399	-2.896560	-3.156931
C	-1.759936	-1.161360	-2.661674
C	4.396068	-2.213136	-1.450678
H	4.834259	-3.194070	-1.205335
H	4.297575	-2.149569	-2.548626
C	5.254832	-1.063427	-0.912893
C	6.670694	-1.041285	-1.499508
H	5.302140	-1.140654	0.189635
H	4.735457	-0.116413	-1.142927
C	7.502607	0.141966	-0.985169
H	7.191285	-1.989431	-1.258490
H	6.612193	-0.994918	-2.604990
C	8.928902	0.186720	-1.551073
H	6.979722	1.087017	-1.234303
H	7.547836	0.101722	0.121518
H	9.448090	-0.758925	-1.300195
H	8.878920	0.223126	-2.656923
C	9.746500	1.375580	-1.033511
H	9.839476	1.345474	0.067509
H	9.266575	2.334857	-1.300599
C	-0.190867	3.311386	-0.431631
C	-0.589806	4.640446	-0.677605
C	1.156570	2.953487	-0.618608
C	0.351886	5.596165	-1.085607
H	-1.639039	4.929719	-0.557046
C	2.097892	3.911989	-1.013123
H	1.446299	1.914496	-0.464824

C	1.698503	5.235717	-1.248237
H	0.031582	6.626488	-1.276177
H	3.145088	3.617690	-1.139310
H	2.432658	5.985533	-1.563086
C	-2.949867	2.649448	-0.503828
C	-3.320499	2.224993	-1.795685
C	-3.801036	3.539622	0.181293
C	-4.523211	2.653659	-2.373134
H	-2.684486	1.528839	-2.342958
C	-5.002489	3.972135	-0.397407
H	-3.539519	3.898465	1.180754
C	-5.373422	3.521634	-1.672720
H	-4.799260	2.296687	-3.370975
H	-5.652540	4.659699	0.154408
H	-6.318302	3.848870	-2.119414
O	-1.320587	-0.025050	-3.505229
C	-0.348113	0.782642	-3.060220
O	0.220748	0.643258	-1.987059
C	-0.070518	1.928645	-4.001818
H	-0.616419	2.813031	-3.625506
H	1.002832	2.168190	-3.974299
H	-0.405480	1.706370	-5.025354
H	-2.662128	-1.468579	-3.215839
C	-0.781553	-2.323472	-2.737741
C	-1.153303	-3.520192	-2.097039
C	0.422053	-2.284718	-3.457714
C	-0.337718	-4.654832	-2.174228
H	-2.080687	-3.547837	-1.515931
C	1.249864	-3.415347	-3.518650
H	0.724048	-1.370485	-3.974377
C	0.874511	-4.604822	-2.879191
H	-0.645972	-5.577589	-1.671167
H	2.190141	-3.365842	-4.079684
H	1.518320	-5.489442	-2.935150
H	10.765730	1.379541	-1.457587

^{3}TS(3-4)

Lowest frequency = -147.9620 cm^{-1}

Charge = 0, Multiplicity = 3

126

P	0.871759	0.949512	1.340724
P	-1.657361	1.870126	0.011201
C	-0.159812	2.130576	2.391918
H	-0.318932	1.639175	3.363976
H	0.444640	3.033177	2.582097
C	-1.525416	2.482627	1.761090
H	-1.721367	3.566989	1.816085
H	-2.334207	1.965434	2.302622
C	-3.403174	-0.638874	1.497040
C	-2.453766	-0.851992	2.534569
C	-4.528699	0.173381	1.782186
C	-2.663434	-0.255373	3.796256
C	-4.687432	0.809163	3.015229
H	-5.275751	0.333165	0.999063
C	-3.745207	0.595371	4.035671
H	-1.967134	-0.512067	4.601258
H	-5.549989	1.463116	3.181494
H	-3.871594	1.066335	5.016556
C	-1.325767	-1.856275	2.476150
N	-0.436391	-1.719119	1.477398
C	0.642038	-2.703624	1.540510
H	1.207172	-2.623342	2.489265
H	0.220538	-3.730786	1.551841
C	1.545281	-2.527095	0.365188
C	2.677460	-3.191126	-0.078646
N	1.249096	-1.576307	-0.568083
N	2.109300	-1.607814	-1.558832
N	2.979659	-2.594789	-1.271969
H	3.263689	-4.009966	0.332818
O	-1.242149	-2.709566	3.388061
Fe	-0.548654	-0.299267	-0.011404
C	1.863782	0.057778	2.604584
C	3.184658	-0.327134	2.296711
C	1.286317	-0.398175	3.806151
C	3.910744	-1.145756	3.171156
H	3.641732	0.014757	1.363472
C	2.010283	-1.220872	4.677681
H	0.253179	-0.143440	4.054515
C	3.324323	-1.599290	4.362645
H	4.937065	-1.433204	2.917467
H	1.533693	-1.584069	5.593482
H	3.887776	-2.247321	5.042327
C	2.145833	2.058641	0.593025
C	2.863882	2.996251	1.364920
C	2.359739	1.993458	-0.796077
C	3.753386	3.884308	0.747505
H	2.727877	3.032602	2.452140
C	3.261090	2.878153	-1.407049
H	1.782093	1.279480	-1.392476
C	3.946487	3.831374	-0.643377
H	4.302069	4.615489	1.351345
H	3.410571	2.831418	-2.491015
H	4.638965	4.530148	-1.125265
C	-3.337678	-1.305553	0.166658
C	-2.220574	-1.249692	-0.627569
C	-4.577740	-2.102915	-0.202279
C	-4.839709	-3.293557	0.748000
H	-4.485715	-2.477451	-1.236578
H	-5.460025	-1.434047	-0.175837
C	-3.685551	-4.299210	0.805092
H	-5.035968	-2.900495	1.763309
H	-5.768552	-3.799095	0.421340
H	-3.952374	-5.170076	1.428746
H	-3.425276	-4.667673	-0.204225
H	-2.782771	-3.841846	1.244978
C	-2.193175	-1.764341	-1.956909
C	4.161655	-2.789053	-2.111823
H	4.260344	-3.866569	-2.325775
H	3.941777	-2.263099	-3.053704
C	5.422146	-2.232925	-1.431258
C	5.338364	-0.728800	-1.138353
H	6.292413	-2.458092	-2.075688
H	5.583772	-2.787375	-0.486555
C	6.422031	-0.233506	-0.172739
H	5.388315	-0.162548	-2.088047
H	4.346116	-0.495888	-0.710448
C	6.290237	1.259509	0.154009
H	6.356939	-0.818889	0.766968
H	7.427544	-0.441128	-0.590541
H	6.404844	1.846957	-0.776791
H	5.262109	1.462197	0.505746
C	7.290734	1.749478	1.204627
H	8.334359	1.593139	0.874506
H	7.157883	1.209391	2.160205
C	-0.879115	3.225766	-0.968437
C	-0.034318	4.188091	-0.381795
C	-1.093975	3.276064	-2.361526
C	0.599746	5.159662	-1.168408
H	0.151106	4.183469	0.694860

C	-0.462260	4.247724	-3.145630
H	-1.769486	2.556432	-2.834036
C	0.394716	5.189237	-2.553540
H	1.262508	5.888704	-0.691145
H	-0.643347	4.271084	-4.225816
H	0.893098	5.945535	-3.169245
C	-3.424584	2.122366	-0.432064
C	-3.944189	1.394345	-1.520668
C	-4.266415	3.001846	0.276081
C	-5.294314	1.528162	-1.876006
H	-3.286168	0.728933	-2.088631
C	-5.614773	3.130351	-0.080971
H	-3.879122	3.580130	1.119878
C	-6.134864	2.385819	-1.151352
H	-5.690123	0.954067	-2.720716
H	-6.262891	3.811752	0.480945
H	-7.191534	2.481066	-1.423739
O	-1.647065	-0.228766	-3.281000
C	-0.481203	0.247798	-3.072895
O	0.053771	0.388086	-1.941731
C	0.342798	0.646981	-4.288671
H	0.865734	1.596447	-4.092043
H	1.103360	-0.139517	-4.442652
H	-0.284362	0.728775	-5.189110
H	-3.089967	-1.668170	-2.581037
C	-1.251931	-2.804996	-2.395281
C	-0.682702	-3.666744	-1.434118
C	-0.980115	-3.034674	-3.763987
C	0.147198	-4.721649	-1.825727
H	-0.925911	-3.496803	-0.382639
C	-0.129532	-4.074248	-4.151058
H	-1.419205	-2.363268	-4.507033
C	0.438925	-4.920821	-3.183906
H	0.569930	-5.388440	-1.066815
H	0.089570	-4.231629	-5.212834
H	1.097160	-5.741172	-3.491090
H	7.158187	2.826583	1.408421

³I-4

Lowest frequency = 11.1672 cm^{-1}

Charge = 0, Multiplicity = 3

126

P	1.103232	1.104051	1.424717
P	-1.746738	1.758528	0.072843
C	0.060392	2.490485	2.138840
H	0.242623	2.582686	3.223987
H	0.412317	3.420944	1.663257
C	-1.436783	2.284938	1.850222
H	-1.981957	3.220779	2.060960
H	-1.865055	1.487771	2.480952
C	-4.030748	-0.927778	0.696412
C	-3.159214	-0.827885	1.815332
C	-5.381353	-0.539638	0.874386
C	-3.652628	-0.292276	3.025016
C	-5.846173	0.002147	2.071487
H	-6.075271	-0.631196	0.035946
C	-4.967603	0.154264	3.155185
H	-2.983207	-0.289733	3.891358
H	-6.892064	0.314490	2.155043
H	-5.317179	0.577453	4.102981
C	-1.857670	-1.580603	1.950411
N	-0.821939	-1.405729	1.088110
C	0.209129	-2.435482	1.364964
H	0.600521	-2.339947	2.393786
H	-0.243279	-3.442358	1.320057
C	1.299241	-2.291111	0.368619
C	2.508312	-2.915411	0.120170
N	1.191866	-1.278557	-0.544509
N	2.241342	-1.226625	-1.333540
N	3.043961	-2.226917	-0.933935
H	3.012984	-3.757616	0.587115
O	-1.812256	-2.397576	2.894809
Fe	-0.384602	-0.076945	-0.348814
C	1.505348	0.078577	2.878533
C	2.724452	-0.632160	2.933921
C	0.526179	-0.168899	3.863424
C	2.954479	-1.562893	3.954672
H	3.491062	-0.455110	2.172268
C	0.753371	-1.111775	4.872436
H	-0.436964	0.347733	3.823423
C	1.967923	-1.812235	4.922010
H	3.906839	-2.103647	3.990112
H	-0.031769	-1.314804	5.606813
H	2.142596	-2.554245	5.707924
C	2.723285	1.869007	1.005611
C	3.556640	2.480743	1.964926
C	3.139996	1.806760	-0.340829
C	4.787805	3.028054	1.581785
H	3.244513	2.516267	3.014544
C	4.372303	2.362286	-0.719144
H	2.489100	1.327459	-1.082754
C	5.196592	2.970409	0.238763
H	5.431250	3.502039	2.330978
H	4.688623	2.314440	-1.766957
H	6.159401	3.400872	-0.057933
C	-3.625936	-1.473754	-0.621317
C	-2.424407	-1.379962	-1.190774
C	-4.695220	-2.182430	-1.461800
C	-5.260984	-3.446045	-0.788605
H	-4.248939	-2.448360	-2.434300
H	-5.518393	-1.474728	-1.683807
C	-6.349322	-4.120642	-1.630729
H	-4.430105	-4.153580	-0.610112
H	-5.663253	-3.186252	0.207519
H	-6.733232	-5.030236	-1.136250
H	-7.204786	-3.439398	-1.794294
H	-5.962763	-4.413199	-2.624141
C	-1.458375	-1.711029	-2.065236
C	4.401869	-2.291651	-1.471010
H	4.710759	-3.350048	-1.490571
H	4.339953	-1.927643	-2.508670
C	5.356338	-1.424746	-0.637455
C	6.752595	-1.283620	-1.253025
H	5.431885	-1.849186	0.381850
H	4.897699	-0.425570	-0.530115
C	7.668627	-0.379452	-0.416123
H	7.220377	-2.281267	-1.371215
H	6.658690	-0.864163	-2.274310
C	9.056880	-0.164049	-1.034049
H	7.172412	0.600539	-0.273631
H	7.780449	-0.812193	0.598020
H	9.552324	-1.145227	-1.170809
H	8.938845	0.261817	-2.049777
C	9.953072	0.752974	-0.193878
H	10.115817	0.334334	0.816088
H	9.493926	1.750902	-0.069233
C	-1.175628	3.321839	-0.772008
C	0.202190	3.598476	-0.873714
C	-2.083688	4.282414	-1.261141

C	0.663938	4.802206	-1.421108
H	0.928731	2.851692	-0.557206
C	-1.622419	5.481894	-1.821995
H	-3.159006	4.096651	-1.210965
C	-0.249174	5.751959	-1.899633
H	1.742574	4.984853	-1.479588
H	-2.347880	6.209883	-2.201143
H	0.105983	6.691974	-2.335534
C	-3.556594	1.958649	-0.152464
C	-4.086687	1.580169	-1.405376
C	-4.389220	2.585703	0.791351
C	-5.434799	1.820843	-1.694658
H	-3.416738	1.135857	-2.147090
C	-5.736352	2.833318	0.489336
H	-4.001089	2.883256	1.768510
C	-6.263204	2.449410	-0.750470
H	-5.837396	1.527410	-2.670308
H	-6.375856	3.321081	1.232814
H	-7.316244	2.642239	-0.983019
O	-1.500340	1.224103	-3.079706
C	-0.276264	1.312343	-2.917761
O	0.379718	0.865407	-1.878543
C	0.618510	1.971490	-3.958890
H	0.959917	2.944974	-3.565122
H	1.510751	1.350552	-4.144332
H	0.058232	2.135766	-4.891109
H	-1.214498	-1.001028	-2.865196
C	-0.739873	-2.993285	-2.041284
C	-1.106922	-4.040050	-1.165380
C	0.360963	-3.195423	-2.904305
C	-0.387690	-5.238311	-1.141721
H	-1.956088	-3.889691	-0.490999
C	1.081507	-4.394695	-2.877926
H	0.659732	-2.386855	-3.579340
C	0.714983	-5.421562	-1.993201
H	-0.686573	-6.035625	-0.452300
H	1.935377	-4.529904	-3.551733
H	1.277957	-6.361091	-1.972965
H	10.942078	0.893376	-0.663903

^{3}TS(4-5)

Lowest frequency = -290.3725 cm^{-1}

Charge = 0, Multiplicity = 3

126

P	-0.933509	-1.394238	1.331025
P	1.852536	-1.707060	-0.081728
C	0.150806	-2.810407	1.890293
H	-0.020145	-3.048852	2.954341
H	-0.163154	-3.687909	1.299970
C	1.629943	-2.488761	1.618139
H	2.234328	-3.409475	1.685774
H	2.019077	-1.763367	2.353065
C	4.014791	1.092771	0.921886
C	3.261622	0.628661	2.040609
C	5.427178	0.948013	0.987060
C	3.921969	0.020049	3.132020
C	6.058787	0.335960	2.065314
H	6.037611	1.297254	0.152249
C	5.302672	-0.152342	3.147039
H	3.304780	-0.275553	3.985496
H	7.148490	0.226446	2.059417
H	5.793444	-0.638502	3.996642
C	1.839360	0.978970	2.272380
N	1.056283	1.374865	1.183633
C	-0.014242	2.314865	1.622035
H	-0.318026	2.017030	2.641963
H	0.387734	3.341748	1.682271
C	-1.175408	2.238154	0.699526
C	-2.361413	2.932260	0.534920
N	-1.195485	1.218075	-0.207896
N	-2.301724	1.222911	-0.915869
N	-3.013724	2.265868	-0.464404
H	-2.771954	3.817199	1.013884
O	1.403585	1.005956	3.437541
Fe	0.368048	0.021928	-0.233307
C	-1.555976	-0.576145	2.844520
C	-2.731673	0.199975	2.727323
C	-0.875753	-0.604406	4.075691
C	-3.213438	0.926902	3.820357
H	-3.272313	0.221008	1.775252
C	-1.366598	0.118993	5.170674
H	0.054422	-1.167712	4.182666
C	-2.531109	0.888843	5.047644
H	-4.127520	1.522053	3.715892
H	-0.821128	0.093419	6.119371
H	-2.906743	1.458669	5.904076
C	-2.495370	-2.167719	0.727567
C	-3.293974	-2.952528	1.585538
C	-2.921959	-1.923870	-0.592857
C	-4.501338	-3.492329	1.126057
H	-2.976619	-3.125463	2.619905
C	-4.135739	-2.465429	-1.045457
H	-2.298235	-1.316632	-1.258570
C	-4.925895	-3.246401	-0.190492
H	-5.116397	-4.101853	1.796903
H	-4.462327	-2.271333	-2.073041
H	-5.873729	-3.665122	-0.546355
C	3.399008	1.723014	-0.238909
C	2.065769	1.761067	-0.499780
C	4.290055	2.436901	-1.252490
C	4.829948	3.787299	-0.741238
H	3.708152	2.605400	-2.171921
H	5.133713	1.779564	-1.536769
C	5.769001	4.463846	-1.745580
H	3.968785	4.447038	-0.526882
H	5.352602	3.639156	0.222302
H	6.129224	5.437644	-1.369296
H	6.654068	3.833133	-1.950043
H	5.258240	4.641279	-2.709536
C	1.239798	2.051128	-1.565962
C	-4.371440	2.450399	-0.975037
H	-4.681047	3.477125	-0.722798
H	-4.315180	2.367807	-2.072601
C	-5.317441	1.393660	-0.388766
C	-6.719421	1.415919	-1.007188
H	-5.382962	1.537674	0.706514
H	-4.856428	0.402680	-0.550344
C	-7.622056	0.309090	-0.443787
H	-7.193509	2.403605	-0.840634
H	-6.635309	1.295797	-2.105453
C	-9.021325	0.273075	-1.073447
H	-7.125420	-0.670211	-0.591818
H	-7.715368	0.437416	0.653029
H	-9.515724	1.252253	-0.919561
H	-8.922150	0.150856	-2.169868
C	-9.906286	-0.844662	-0.510041
H	-10.049902	-0.727775	0.579687
H	-9.449211	-1.836784	-0.679991
C	1.430301	-3.177838	-1.140702

C	0.080126	-3.535566	-1.321896
C	2.422869	-3.988777	-1.726540
C	-0.273813	-4.678647	-2.050128
H	-0.707753	-2.899119	-0.920934
C	2.068388	-5.126773	-2.464618
H	3.478778	-3.730997	-1.613260
C	0.721325	-5.479912	-2.626920
H	-1.333012	-4.930712	-2.171213
H	2.855371	-5.739974	-2.916951
H	0.449566	-6.370361	-3.203999
C	3.671478	-1.669738	-0.325241
C	4.142976	-1.081988	-1.518575
C	4.580167	-2.296637	0.546498
C	5.506749	-1.134736	-1.830620
H	3.419936	-0.626157	-2.202274
C	5.943700	-2.348473	0.223774
H	4.238185	-2.746388	1.481791
C	6.410123	-1.770179	-0.964101
H	5.863803	-0.683819	-2.762894
H	6.642853	-2.836585	0.911028
H	7.476026	-1.812553	-1.213359
O	1.557578	-0.709106	-3.156674
C	0.341663	-0.894931	-3.013901
O	-0.328525	-0.669489	-1.917145
C	-0.517406	-1.431878	-4.152039
H	0.029298	-1.362194	-5.104098
H	-0.748727	-2.492373	-3.946959
H	-1.471400	-0.882024	-4.209942
H	1.340090	1.392076	-2.439765
C	0.375978	3.215610	-1.670987
C	0.555226	4.357014	-0.843628
C	-0.669211	3.265296	-2.630837
C	-0.287965	5.466688	-0.940968
H	1.387483	4.358977	-0.130575
C	-1.510004	4.378447	-2.727857
H	-0.823574	2.399989	-3.284000
C	-1.337835	5.484180	-1.876442
H	-0.119593	6.333492	-0.291134
H	-2.311232	4.386128	-3.476714
H	-1.995829	6.356238	-1.957387
H	-10.903800	-0.847791	-0.983062

^{3}I-5
Lowest frequency = 6.9035 cm^{-1}
Charge = 0, Multiplicity = 3

126

P	-1.404160	0.858550	-0.975169
P	1.347614	1.844119	-0.331588
C	-0.603394	1.997749	-2.247472
H	-0.982795	1.757844	-3.254795
H	-0.893460	3.032471	-1.998361
C	0.923369	1.850596	-2.160279
H	1.429476	2.676166	-2.691888
H	1.260966	0.892315	-2.594549
C	4.170792	-1.280288	-1.492922
C	3.364749	-2.003615	-2.421191
C	5.420375	-0.786995	-1.969518
C	3.767946	-2.174781	-3.763317
C	5.806496	-0.961596	-3.294397
H	6.078560	-0.244364	-1.287388
C	4.975694	-1.647129	-4.209591
H	3.107065	-2.747011	-4.421150
H	6.769436	-0.560060	-3.630105
H	5.288787	-1.775933	-5.250738
C	2.153463	-2.687724	-1.972427
N	1.770985	-2.450400	-0.635905
C	0.797638	-3.424662	-0.118528
H	0.710825	-4.180514	-0.914200
H	1.221692	-3.891881	0.785586
C	-0.576818	-2.931890	0.210179
C	-1.752480	-3.664379	0.323987
N	-0.890533	-1.648761	0.543925
N	-2.167353	-1.541836	0.850964
N	-2.689705	-2.761194	0.730294
H	-1.980840	-4.713793	0.151314
O	1.504893	-3.472238	-2.685209
Fe	0.198255	0.040985	0.547438
C	-2.007941	-0.479476	-2.099914
C	-3.332984	-0.583507	-2.561866
C	-1.052476	-1.411927	-2.549623
C	-3.688531	-1.604501	-3.456391
H	-4.090274	0.128194	-2.220005
C	-1.404712	-2.430155	-3.444226
H	-0.026397	-1.348825	-2.173634
C	-2.728749	-2.527954	-3.898435
H	-4.724017	-1.676505	-3.808344
H	-0.633517	-3.140359	-3.756598
H	-3.013128	-3.323546	-4.595834
C	-2.962129	1.714337	-0.497685
C	-3.669833	2.559392	-1.376014
C	-3.451845	1.504354	0.808297
C	-4.861179	3.171793	-0.962376
H	-3.296193	2.738223	-2.389835
C	-4.644638	2.119146	1.215671
H	-2.871079	0.885588	1.499851
C	-5.353645	2.948136	0.332718
H	-5.406036	3.824952	-1.652604
H	-5.017341	1.955956	2.232942
H	-6.284568	3.427491	0.654860
C	3.690386	-1.046820	-0.152165
C	2.442649	-1.540181	0.230472
C	4.595601	-0.351632	0.841829
C	5.623257	-1.308711	1.482703
H	4.006781	0.117745	1.642909
H	5.138428	0.472493	0.351576
C	6.579955	-0.576153	2.429327
H	5.082510	-2.101022	2.031950
H	6.196521	-1.820868	0.686519
H	7.309269	-1.268420	2.886014
H	7.145143	0.207761	1.892199
H	6.024111	-0.080557	3.245966
C	1.721704	-1.080853	1.439498
C	-4.123604	-2.932502	1.013954
H	-4.363300	-3.992607	0.835364
H	-4.271045	-2.720343	2.086973
C	-4.954105	-1.982825	0.145352
C	-6.415341	-1.845578	0.582156
H	-4.891256	-2.311266	-0.906685
H	-4.473073	-0.991129	0.192241
C	-7.148586	-0.787520	-0.255639
H	-6.940834	-2.818364	0.508209
H	-6.451820	-1.552267	1.650290
C	-8.592487	-0.525576	0.192112
H	-6.576017	0.159233	-0.209038
H	-7.142359	-1.096258	-1.320108
H	-9.174130	-1.466402	0.133034
H	-8.591149	-0.233704	1.260721
C	-9.284555	0.564012	-0.635064
H	-9.330485	0.284015	-1.703427

H	-8.734247	1.520341	-0.566550
C	0.621021	3.501027	0.079749
C	-0.588463	3.586671	0.788793
C	1.204483	4.677947	-0.431079
C	-1.219972	4.824714	0.970090
H	-1.031994	2.675114	1.194689
C	0.576444	5.916369	-0.241823
H	2.153940	4.626708	-0.975309
C	-0.640158	5.992275	0.455040
H	-2.168565	4.869603	1.515787
H	1.038773	6.825884	-0.641043
H	-1.131007	6.961309	0.597215
C	3.130809	2.273327	-0.283366
C	3.654445	2.802187	0.917017
C	3.961371	2.162878	-1.413543
C	4.986954	3.226158	0.970579
H	3.005379	2.864168	1.796494
C	5.296997	2.588963	-1.351541
H	3.581095	1.736870	-2.346144
C	5.811259	3.124742	-0.162553
H	5.386954	3.635565	1.904396
H	5.934298	2.494773	-2.237107
H	6.853117	3.459807	-0.116884
O	1.256174	1.787215	2.572956
C	0.122485	1.444744	2.977170
O	-0.731843	0.790208	2.258141
C	-0.312576	1.765936	4.400307
H	-1.389508	1.997843	4.436489
H	-0.137493	0.873977	5.029769
H	0.278594	2.601592	4.804400
H	2.254508	-0.240890	1.907756
C	1.396469	-2.090399	2.512994
C	2.266391	-3.180554	2.751529
C	0.262497	-1.968018	3.344507
C	2.012463	-4.114195	3.765644
H	3.160438	-3.289070	2.125322
C	0.011080	-2.895983	4.364529
H	-0.428848	-1.137578	3.173500
C	0.878314	-3.977566	4.579930
H	2.707267	-4.947042	3.923424
H	-0.878031	-2.774406	4.994548
H	0.676385	-4.703372	5.375316
H	-10.317611	0.741749	-0.288483

^{5}I-0

Lowest frequency = 8.4423 cm^{-1}

Charge = 0, Multiplicity = 5

105

P	1.025301	-1.056007	-1.198360
P	0.317755	2.003590	-0.765919
C	0.946813	0.065687	-2.699510
H	1.617071	-0.298507	-3.496937
H	-0.084378	-0.008130	-3.083853
C	1.269569	1.511511	-2.299746
H	1.084294	2.223175	-3.124133
H	2.333402	1.607020	-2.016973
C	3.848983	0.317488	0.467580
C	4.081720	-0.944153	1.046766
C	4.817442	0.936927	-0.332296
C	5.332295	-1.552173	0.831484
C	6.049070	0.305499	-0.560094
H	4.620412	1.922871	-0.768546
C	6.305879	-0.938331	0.035319
H	5.498675	-2.523702	1.305720
H	6.807104	0.788142	-1.186734
H	7.268488	-1.435150	-0.128942
C	3.073088	-1.723614	1.863716
N	1.793823	-1.269145	1.850725
C	0.891318	-2.141084	2.591357
H	0.974528	-3.193279	2.243678
H	1.165001	-2.185932	3.666587
C	-0.519083	-1.647069	2.448284
C	-1.733723	-2.072932	2.967299
N	-0.786424	-0.518434	1.717258
N	-2.066618	-0.217954	1.764349
N	-2.647199	-1.157367	2.521722
H	-2.012109	-2.911631	3.602130
O	3.438246	-2.762553	2.453854
Fe	0.959520	0.433890	1.034571
C	2.431407	-2.184447	-1.472217
C	2.523364	-3.337404	-0.660777
C	3.505633	-1.871506	-2.329485
C	3.641604	-4.173458	-0.733594
H	1.725413	-3.568244	0.050720
C	4.624580	-2.710642	-2.398542
H	3.485022	-0.960734	-2.935840
C	4.694166	-3.866558	-1.608032
H	3.699499	-5.049572	-0.080720
H	5.453880	-2.448651	-3.063803
H	5.576675	-4.512952	-1.655914
C	-0.476568	-2.085526	-1.471495
C	-0.461911	-3.428493	-1.897245
C	-1.719362	-1.437798	-1.294869
C	-1.664918	-4.107655	-2.137886
H	0.491255	-3.941306	-2.056186
C	-2.916264	-2.110918	-1.566649
H	-1.748240	-0.399221	-0.951525
C	-2.894928	-3.451687	-1.980764
H	-1.637396	-5.151657	-2.468877
H	-3.867433	-1.581297	-1.455044
H	-3.831804	-3.980770	-2.186629
C	-4.096672	-1.095282	2.740044
H	-4.358182	-1.928817	3.411330
H	-4.315005	-0.149978	3.266638
C	-4.855346	-1.167200	1.411452
C	-6.365058	-0.951353	1.559225
H	-4.651014	-2.143843	0.936527
H	-4.432003	-0.396587	0.743815
C	-7.086293	-0.956785	0.203736
H	-6.799933	-1.732636	2.213681
H	-6.551289	0.015402	2.068085
C	-8.599027	-0.718249	0.306587
H	-6.635030	-0.180934	-0.446855
H	-6.900176	-1.924012	-0.304334
H	-9.046270	-1.496739	0.955138
H	-8.779391	0.246634	0.819710
C	-9.302726	-0.715818	-1.055212
H	-9.166480	-1.681444	-1.575547
H	-8.896139	0.075449	-1.711406
C	-1.418068	2.159881	-1.347071
C	-1.810052	2.110568	-2.702462
C	-2.420571	2.209210	-0.352451
C	-3.167786	2.088113	-3.050830
H	-1.056455	2.091608	-3.495722
C	-3.775044	2.212999	-0.704782
H	-2.130954	2.219413	0.701917
C	-4.154981	2.138182	-2.055105
H	-3.455172	2.037238	-4.106623
H	-4.538170	2.260004	0.079790

H	-5.215173	2.119631	-2.329887
C	0.871491	3.741491	-0.571397
C	2.208995	3.953736	-0.178828
C	0.027394	4.851377	-0.763622
C	2.698646	5.251561	0.005215
H	2.858846	3.092558	0.005502
C	0.517598	6.150223	-0.567026
H	-1.014086	4.697164	-1.063416
C	1.851881	6.354637	-0.185139
H	3.738912	5.400417	0.313362
H	-0.148025	7.007646	-0.714527
H	2.230238	7.370708	-0.031290
H	-10.387426	-0.540557	-0.948798
C	1.130559	1.772825	2.641083
C	0.035852	2.834717	2.725439
C	2.511295	2.309844	3.015207
H	0.883469	0.962818	3.364781
H	-0.969029	2.398885	2.563758
H	0.011304	3.334908	3.720355
H	0.177241	3.635523	1.975532
H	3.271417	1.508611	3.029907
H	2.859146	3.089587	2.312855
H	2.510512	2.778714	4.025644
H	2.917006	0.869937	0.672045

^{5}TS(0-1)
Lowest frequency = -1262.1748 cm^{-1}
Charge = 0, Multiplicity = 5

105

P	0.634998	-1.145821	-1.228736
P	0.941288	1.986443	-0.593721
C	0.774084	0.090675	-2.622527
H	1.172544	-0.391761	-3.532526
H	-0.257633	0.415277	-2.845769
C	1.622420	1.289861	-2.187702
H	1.650161	2.069448	-2.969611
H	2.657788	0.974479	-1.969426
C	3.319901	-0.281542	0.701199
C	3.596448	-1.614235	1.105909
C	4.334146	0.404264	0.004786
C	4.824039	-2.226705	0.805936
C	5.554480	-0.209737	-0.322635
H	4.189174	1.458808	-0.258506
C	5.801025	-1.531229	0.082069
H	4.977647	-3.256693	1.144711
H	6.321211	0.348600	-0.874165
H	6.754194	-2.013683	-0.162758
C	2.548470	-2.420991	1.822876
N	1.330876	-1.806411	1.852146
C	0.274529	-2.582830	2.459781
H	0.148639	-3.571533	1.964831
H	0.494531	-2.828148	3.520468
C	-0.989175	-1.782562	2.365062
C	-2.277127	-1.978385	2.842250
N	-0.982776	-0.565451	1.732309
N	-2.173893	-0.007331	1.801694
N	-2.962069	-0.854471	2.476206
H	-2.741486	-2.787833	3.401929
O	2.776035	-3.555171	2.287516
Fe	1.027009	0.004988	0.972973
C	1.907858	-2.408359	-1.559783
C	1.845830	-3.624243	-0.846246
C	3.026525	-2.152571	-2.376358
C	2.866986	-4.572110	-0.965616
H	1.008305	-3.818743	-0.170400
C	4.047955	-3.103478	-2.492385
H	3.120154	-1.202355	-2.910327
C	3.969876	-4.315347	-1.792073
H	2.812938	-5.495336	-0.381349
H	4.918616	-2.885542	-3.119423
H	4.779036	-5.048703	-1.872487
C	-0.966330	-1.955430	-1.635838
C	-1.094760	-3.254329	-2.166605
C	-2.131896	-1.188394	-1.418095
C	-2.361822	-3.773432	-2.469240
H	-0.201652	-3.857358	-2.355041
C	-3.392240	-1.700200	-1.748482
H	-2.044689	-0.181820	-0.996653
C	-3.513475	-2.999014	-2.266923
H	-2.445779	-4.784949	-2.881500
H	-4.283848	-1.082236	-1.600639
H	-4.500452	-3.403043	-2.516949
C	-4.379689	-0.527953	2.657054
H	-4.784241	-1.242080	3.392175
H	-4.431065	0.482761	3.095409
C	-5.140554	-0.589461	1.328759
C	-6.619074	-0.208777	1.462629
H	-5.043751	-1.606953	0.908100
H	-4.638795	0.092298	0.619453
C	-7.354076	-0.231555	0.114953
H	-7.122048	-0.898984	2.168591
H	-6.702266	0.802116	1.908792
C	-8.838230	0.147515	0.215432
H	-6.845307	0.459730	-0.586508
H	-7.261958	-1.241070	-0.332961
H	-9.342303	-0.544976	0.917607
H	-8.924864	1.155493	0.666221
C	-9.559578	0.124696	-1.136864
H	-9.516684	-0.880732	-1.594000
H	-9.095793	0.833100	-1.847365
C	-0.693785	2.649252	-1.147261
C	-0.821668	3.474200	-2.284154
C	-1.839830	2.322379	-0.401746
C	-2.080177	3.944424	-2.678045
H	0.068315	3.759865	-2.855983
C	-3.099438	2.804099	-0.792502
H	-1.753483	1.683512	0.481752
C	-3.223221	3.608446	-1.932818
H	-2.171583	4.580555	-3.565160
H	-3.984296	2.547196	-0.199329
H	-4.206049	3.981032	-2.241384
C	1.872606	3.567417	-0.428549
C	3.159665	3.758238	-0.970557
C	1.299301	4.610397	0.331881
C	3.865492	4.946382	-0.732540
H	3.622499	2.980699	-1.584300
C	2.003951	5.796218	0.565303
H	0.289042	4.492941	0.738313
C	3.295222	5.965754	0.041604
H	4.866457	5.073338	-1.158663
H	1.542834	6.591812	1.160218
H	3.849950	6.890746	0.230241
H	-10.622935	0.401458	-1.031070
C	2.042304	1.334619	2.746885
C	0.699366	2.030305	2.979994
C	3.206502	2.321729	2.774951
H	2.187249	0.541557	3.504960
H	-0.142625	1.324298	3.077518
H	0.722252	2.635781	3.914141

H	0.452369	2.733302	2.162188
H	4.175977	1.811876	2.633593
H	3.108536	3.079743	1.976779
H	3.252721	2.870653	3.739740
H	2.585692	0.542013	1.586841

^{5}TS(0-1')

Lowest frequency = -806.3698 cm^{-1}
Charge = 0, Multiplicity = 5

105

N	1.153214	-0.085631	1.455305
C	1.522189	0.992621	2.221074
C	2.843058	0.786003	2.585501
N	3.180731	-0.413724	2.022870
N	2.151971	-0.931857	1.333345
C	0.521596	2.065634	2.529407
N	-0.747256	1.669738	1.954965
C	-1.689529	2.640984	1.872510
O	-1.556227	3.830809	2.214039
C	4.482396	-1.082548	1.999633
C	5.511545	-0.298554	1.176923
C	6.823576	-1.068687	0.985105
C	7.849630	-0.293573	0.147007
C	9.161746	-1.057694	-0.078660
C	10.177611	-0.277764	-0.920929
Fe	-0.930874	-0.047166	0.851912
C	-3.452448	0.839980	1.542652
C	-2.954679	2.125102	1.229175
C	-3.646139	2.924366	0.303266
C	-4.787961	2.432798	-0.341669
C	-5.276668	1.152243	-0.033165
C	-4.619275	0.365425	0.923403
P	-2.054702	-1.458948	-0.810114
C	-2.202408	-0.621194	-2.473068
C	-0.830226	-0.006589	-2.778846
P	-0.195314	0.978093	-1.317881
C	-0.797479	2.682412	-1.619671
C	-0.394941	3.683700	-0.710680
C	-0.868928	4.994330	-0.831607
C	-1.764276	5.324203	-1.859896
C	-2.181947	4.334108	-2.761682
C	-1.704650	3.021660	-2.643222
C	-3.652528	-2.294496	-0.559076
C	-3.760530	-3.162114	0.552247
C	-4.989952	-3.746566	0.876769
C	-6.127658	-3.481482	0.096892
C	-6.023734	-2.633416	-1.017024
C	-4.796221	-2.042301	-1.344204
C	-0.941489	-2.878034	-1.197324
C	-1.282190	-3.873261	-2.134469
C	-0.378897	-4.905741	-2.420111
C	0.864284	-4.957480	-1.767097
C	1.201026	-3.976232	-0.823804
C	0.298690	-2.941321	-0.539782
C	1.600424	1.062213	-1.721591
C	2.306436	2.259467	-1.953055
C	3.678946	2.231510	-2.240356
C	4.364787	1.010612	-2.316227
C	3.668847	-0.187163	-2.084607
C	2.304959	-0.161560	-1.773642
C	-0.687032	-0.936672	4.081065
C	-1.093145	-1.941427	3.218339
C	-1.625262	-0.032375	4.821535
H	-0.834017	0.611949	-3.693198
H	-0.090495	-0.811468	-2.935181
H	-2.515564	-1.316713	-3.272531
H	-2.975531	0.161742	-2.363754
H	-5.018545	-0.616219	1.194042
H	-3.264023	3.927058	0.092789
H	-6.178429	0.771089	-0.524653
H	-5.304347	3.053730	-1.082422
H	0.856186	3.051636	2.138030
H	0.451749	2.204695	3.629517
H	3.539905	1.365335	3.187567
H	0.276476	3.430798	0.113807
H	-2.051985	2.264051	-3.351869
H	-0.563999	5.743198	-0.095172
H	-2.889377	4.581558	-3.560599
H	-2.148256	6.345878	-1.948713
H	1.779404	3.217768	-1.924817
H	1.781757	-1.101674	-1.572432
H	4.210141	3.172022	-2.423182
H	4.190348	-1.149241	-2.140433
H	5.433558	0.991529	-2.555645
H	4.820070	-1.228559	3.040647
H	4.290595	-2.072586	1.557480
H	5.714605	0.671150	1.669617
H	5.063012	-0.064877	0.195697
H	7.263954	-1.315330	1.971676
H	6.608288	-2.038522	0.494205
H	7.398733	-0.039633	-0.832884
H	8.067949	0.674352	0.640383
H	9.608155	-1.309713	0.903162
H	8.937564	-2.025331	-0.568835
H	10.445222	0.679139	-0.436740
H	9.768170	-0.041666	-1.920176
H	-2.259008	-3.844907	-2.630898
H	0.551069	-2.166849	0.190542
H	-0.645861	-5.677271	-3.150627
H	2.164046	-4.008713	-0.303049
H	1.565165	-5.768967	-1.991636
H	-2.873905	-3.360999	1.162820
H	-4.738809	-1.368515	-2.203958
H	-5.062046	-4.412136	1.743802
H	-6.905048	-2.428190	-1.634543
H	-7.089590	-3.937698	0.353102
H	11.108173	-0.853665	-1.065904
H	0.380585	-0.680650	4.118825
H	-1.486079	-1.490549	1.661931
H	-0.350541	-2.645727	2.824336
H	-2.110284	-2.344962	3.314035
H	-1.673480	0.974329	4.354824
H	-2.653234	-0.438468	4.831770
H	-1.310424	0.120015	5.872756
H	-2.959138	0.243012	2.316783

^{5}I-1'

Lowest frequency = 5.8423 cm^{-1}
Charge = 0, Multiplicity = 5

96

P	0.290835	-0.668879	-1.084911
P	2.200555	1.678128	-0.324784
C	1.014812	0.408392	-2.439040
H	1.052998	-0.150175	-3.390727
H	0.284536	1.226038	-2.572707
C	2.378764	0.998591	-2.049467

H	2.696315	1.757285	-2.787684
H	3.157995	0.216421	-1.994084
C	3.450007	-1.133862	1.109992
C	2.950736	-2.448519	1.191174
C	4.620769	-0.864046	0.384859
C	3.658628	-3.490821	0.568093
C	5.289680	-1.903192	-0.278866
H	5.022405	0.152861	0.357904
C	4.809843	-3.219521	-0.179200
H	3.269833	-4.506554	0.680667
H	6.199795	-1.689661	-0.850748
H	5.339466	-4.035753	-0.683000
C	1.681186	-2.784073	1.925635
N	0.800750	-1.749998	2.041473
C	-0.419408	-2.110664	2.749160
H	-0.768967	-3.115624	2.433492
H	-0.240700	-2.201594	3.841633
C	-1.465983	-1.069233	2.493522
C	-2.803059	-0.951153	2.840294
N	-1.142581	0.064950	1.794141
N	-2.180775	0.864396	1.690162
N	-3.193012	0.256251	2.330757
H	-3.482035	-1.599278	3.390686
O	1.468493	-3.943035	2.331774
Fe	0.933821	0.119078	1.226055
C	0.865506	-2.360016	-1.476255
C	0.324134	-3.424725	-0.723295
C	1.867930	-2.638587	-2.424877
C	0.757028	-4.737842	-0.931603
H	-0.429471	-3.218927	0.042112
C	2.302972	-3.955163	-2.629081
H	2.323748	-1.831346	-3.005624
C	1.746589	-5.007340	-1.889139
H	0.339948	-5.544322	-0.321328
H	3.088767	-4.155667	-3.365088
H	2.096468	-6.033366	-2.044152
C	-1.478775	-0.668644	-1.605151
C	-2.169516	-1.795889	-2.091881
C	-2.170167	0.558334	-1.497012
C	-3.516523	-1.694263	-2.470079
H	-1.650315	-2.753930	-2.189873
C	-3.506812	0.661750	-1.897564
H	-1.656241	1.439500	-1.100074
C	-4.188302	-0.466327	-2.383055
H	-4.037676	-2.579295	-2.851264
H	-4.018939	1.627433	-1.822290
H	-5.235881	-0.387655	-2.693355
C	-4.532798	0.843456	2.287332
H	-4.952759	0.826300	3.307942
H	-4.382051	1.893895	1.993432
C	-5.442089	0.115939	1.289417
C	-6.793328	0.817222	1.105445
H	-5.601180	-0.924701	1.630063
H	-4.913960	0.054515	0.321805
C	-7.704390	0.098613	0.100757
H	-7.312363	0.898096	2.081321
H	-6.620137	1.856848	0.763175
C	-9.050515	0.802527	-0.120248
H	-7.173642	0.007063	-0.867382
H	-7.884050	-0.938504	0.447233
H	-9.577352	0.891332	0.849992
H	-8.864914	1.839626	-0.462124
C	-9.950284	0.081593	-1.130385
H	-10.179714	-0.946777	-0.796418
H	-9.460077	0.008568	-2.118460
C	1.055179	3.096967	-0.591501
C	1.319759	4.123962	-1.518459
C	-0.147635	3.114705	0.137009
C	0.380330	5.142602	-1.725504
H	2.265520	4.131083	-2.072659
C	-1.087543	4.133954	-0.071255
H	-0.339796	2.329091	0.875588
C	-0.826167	5.146277	-1.005283
H	0.589452	5.939016	-2.448045
H	-2.021355	4.128808	0.500737
H	-1.557369	5.945265	-1.169845
C	3.794418	2.463249	0.078643
C	3.978147	2.890976	1.413242
C	4.857992	2.587897	-0.840728
C	5.202275	3.440721	1.811072
H	3.156896	2.751175	2.126669
C	6.080795	3.138181	-0.432829
H	4.739248	2.249877	-1.874940
C	6.255691	3.568166	0.891402
H	5.338258	3.763972	2.848756
H	6.900377	3.232070	-1.153710
H	7.212642	3.997536	1.206576
H	-10.908269	0.612538	-1.268310
H	2.978690	-0.328374	1.704323
H	1.487801	1.364462	2.266117

^{5}TS(1'-1)
Lowest frequency = -1017.1789 cm^{-1}
Charge = 0, Multiplicity = 5

96

P	1.105141	-0.876933	-1.139394
P	0.440000	2.138239	-0.310222
C	0.987457	0.424361	-2.480170
H	1.642924	0.161033	-3.328690
H	-0.051523	0.369052	-2.844641
C	1.298289	1.826691	-1.939121
H	1.040704	2.611702	-2.673675
H	2.372836	1.927261	-1.707808
C	3.416833	0.513864	0.895170
C	3.982762	-0.736104	1.275969
C	4.252708	1.430601	0.226716
C	5.324683	-1.038601	0.991778
C	5.588140	1.121539	-0.080839
H	3.868826	2.424651	-0.043451
C	6.126784	-0.115416	0.309214
H	5.707241	-2.014096	1.310365
H	6.213997	1.850653	-0.610253
H	7.170689	-0.357377	0.078970
C	3.139608	-1.793347	1.946766
N	1.811310	-1.478811	1.951910
C	0.908159	-2.455856	2.514972
H	0.950708	-3.425924	1.971883
H	1.171390	-2.704272	3.564398
C	-0.480552	-1.881003	2.446392
C	-1.725405	-2.327918	2.866861
N	-0.679861	-0.642009	1.888695
N	-1.947815	-0.302101	1.950056
N	-2.588320	-1.317643	2.544220
H	-2.054570	-3.240915	3.358994
O	3.617060	-2.852276	2.394078
Fe	1.160415	0.217055	1.080369
C	2.610116	-1.842173	-1.478376
C	2.769918	-3.082147	-0.822777
C	3.690790	-1.310193	-2.208451

C	3.975157	-3.784755	-0.917374
H	1.957957	-3.484278	-0.209614
C	4.894766	-2.018596	-2.303114
H	3.610266	-0.327795	-2.683445
C	5.039739	-3.256838	-1.661857
H	4.089779	-4.730172	-0.378915
H	5.731201	-1.587565	-2.862771
H	5.989302	-3.798674	-1.722841
C	-0.308823	-1.959661	-1.590521
C	-0.192965	-3.206617	-2.235393
C	-1.594639	-1.453294	-1.295244
C	-1.342487	-3.931420	-2.581213
H	0.797191	-3.604609	-2.477893
C	-2.738728	-2.167319	-1.671277
H	-1.692388	-0.494073	-0.774344
C	-2.616937	-3.412369	-2.308408
H	-1.240102	-4.900072	-3.082652
H	-3.728923	-1.749088	-1.464905
H	-3.512518	-3.974831	-2.593796
C	-4.044101	-1.233442	2.705054
H	-4.356470	-2.101551	3.307153
H	-4.259377	-0.318885	3.283563
C	-4.745209	-1.193590	1.343117
C	-6.249214	-0.917168	1.439427
H	-4.563151	-2.149927	0.820184
H	-4.261540	-0.403347	0.742274
C	-6.909920	-0.818499	0.056999
H	-6.746598	-1.709530	2.033195
H	-6.413491	0.030173	1.990679
C	-8.411417	-0.504678	0.109668
H	-6.391207	-0.037183	-0.533555
H	-6.751886	-1.768507	-0.491530
H	-8.926575	-1.289133	0.697791
H	-8.563989	0.441835	0.664426
C	-9.053304	-0.396280	-1.278129
H	-8.944382	-1.340580	-1.842187
H	-8.577691	0.403660	-1.874742
C	-1.338034	2.298663	-0.767392
C	-1.825043	2.311079	-2.090818
C	-2.264346	2.347174	0.295985
C	-3.204247	2.352350	-2.343920
H	-1.131025	2.289732	-2.936517
C	-3.639322	2.406940	0.041824
H	-1.902673	2.316083	1.327799
C	-4.115220	2.399085	-1.279154
H	-3.565734	2.351605	-3.377828
H	-4.343833	2.447328	0.879752
H	-5.192084	2.431873	-1.477446
C	0.921224	3.885268	0.024294
C	1.802368	4.156952	1.087908
C	0.439311	4.948190	-0.767131
C	2.215544	5.473655	1.339990
H	2.141226	3.333561	1.725256
C	0.851316	6.261396	-0.509516
H	-0.266848	4.746787	-1.580690
C	1.744140	6.525249	0.542069
H	2.902357	5.675854	2.168755
H	0.473245	7.082568	-1.128269
H	2.064793	7.553280	0.742580
H	-10.131105	-0.168297	-1.207783
H	2.466521	1.127431	1.874405
H	1.675539	1.469059	2.425830

^{5}I-1

Lowest frequency = 12.7182 cm^{-1}

Charge = 0, Multiplicity = 5

107

P	1.090616	-1.365734	-1.178878
P	-0.058391	1.663081	-1.179435
C	1.235269	-0.317574	-2.714320
H	2.189339	0.230622	-2.619231
H	1.272368	-0.940199	-3.626052
C	0.049946	0.655562	-2.758356
H	-0.891043	0.088008	-2.860793
H	0.119021	1.342637	-3.619915
C	3.271861	0.308152	0.178488
C	3.913933	-0.649867	1.025069
C	4.115308	0.960871	-0.747808
C	5.288022	-0.926651	0.947447
C	5.495578	0.696956	-0.831913
H	3.699337	1.706252	-1.441626
C	6.088270	-0.249958	0.017380
H	5.698571	-1.687780	1.620600
H	6.109644	1.231901	-1.568228
H	7.161388	-0.462701	-0.052369
C	3.078716	-1.435897	2.002073
N	1.761067	-1.064429	1.963520
C	0.833052	-1.816542	2.774237
H	0.806719	-2.893099	2.495530
H	1.112688	-1.807312	3.849714
C	-0.520524	-1.192688	2.571600
C	-1.790031	-1.423212	3.083107
N	-0.634875	-0.136712	1.702932
N	-1.875754	0.296107	1.653452
N	-2.584245	-0.476454	2.494918
H	-2.179184	-2.148708	3.794878
O	3.528868	-2.331170	2.739712
Fe	1.222259	0.322180	0.586030
C	2.525040	-2.489714	-1.225178
C	2.547173	-3.543623	-0.288220
C	3.669768	-2.241480	-2.006767
C	3.695632	-4.324308	-0.124901
H	1.670137	-3.728728	0.341083
C	4.816554	-3.028512	-1.843342
H	3.687094	-1.412010	-2.718075
C	4.834855	-4.065971	-0.900718
H	3.709563	-5.114429	0.632032
H	5.708168	-2.811793	-2.440660
H	5.742042	-4.662838	-0.759131
C	-0.330990	-2.464333	-1.613101
C	-0.211208	-3.599204	-2.441098
C	-1.596559	-2.125644	-1.091863
C	-1.339629	-4.368071	-2.755124
H	0.772964	-3.879001	-2.832925
C	-2.727868	-2.888506	-1.419052
H	-1.680594	-1.259474	-0.425873
C	-2.600645	-4.010746	-2.250417
H	-1.237250	-5.248501	-3.399190
H	-3.708523	-2.613423	-1.016755
H	-3.481529	-4.612332	-2.499693
C	-4.037580	-0.303579	2.546668
H	-4.387099	-0.682499	3.520985
H	-4.222324	0.781913	2.504039
C	-4.723683	-1.011517	1.372853
C	-6.221944	-0.704754	1.279730
H	-4.557836	-2.101871	1.457009
H	-4.222097	-0.675939	0.448614
C	-6.892813	-1.402529	0.088526
H	-6.730713	-1.005111	2.217183

H	-6.360408	0.390472	1.188168
C	-8.384604	-1.072183	-0.058486
H	-6.362090	-1.119331	-0.842433
H	-6.766212	-2.499005	0.187765
H	-8.912101	-1.353738	0.873790
H	-8.503456	0.024708	-0.156661
C	-9.042792	-1.770201	-1.253909
H	-8.967821	-2.869274	-1.163854
H	-8.554440	-1.480195	-2.202192
C	-1.833216	2.130021	-1.079537
C	-2.873572	1.271751	-1.494927
C	-2.179455	3.324991	-0.407266
C	-4.213370	1.597560	-1.245590
H	-2.646511	0.336584	-2.014840
C	-3.517242	3.641107	-0.147651
H	-1.388907	4.013873	-0.090384
C	-4.544174	2.776697	-0.561582
H	-5.005012	0.925385	-1.594909
H	-3.761126	4.573763	0.373004
H	-5.592238	3.028055	-0.366680
C	0.736227	3.255559	-1.647493
C	1.888072	3.661177	-0.948323
C	0.237314	4.060695	-2.693701
C	2.539775	4.853804	-1.298314
H	2.257290	3.039079	-0.127405
C	0.889324	5.250447	-3.038050
H	-0.669580	3.758391	-3.230146
C	2.044018	5.646871	-2.341892
H	3.438902	5.158958	-0.752489
H	0.497977	5.871887	-3.850954
H	2.554125	6.576991	-2.615182
H	-10.112881	-1.511020	-1.333233
O	1.361414	2.182743	1.841443
C	0.207416	2.806086	2.437265
C	2.361955	2.114515	2.880119
C	0.168764	2.299215	3.897266
H	0.339045	3.905166	2.390706
H	-0.667828	2.516969	1.837832
C	1.572684	1.668974	4.114212
H	3.134153	1.411634	2.537602
H	2.804023	3.123104	3.012792
H	-0.627903	1.550409	4.034092
H	-0.027314	3.129760	4.596225
H	1.508374	0.570439	4.115208
H	2.047611	1.991809	5.055103

^{5}I-2

Lowest frequency = 10.2351 cm^{-1}

Charge = 0, Multiplicity = 5

126

P	0.183631	1.440987	-1.818682
P	-1.071298	-1.493662	-1.836404
C	-0.154148	0.583174	-3.457165
H	-0.357706	1.325214	-4.248561
H	0.788998	0.080004	-3.725880
C	-1.295429	-0.438126	-3.346564
H	-1.374552	-1.053114	-4.261579
H	-2.255812	0.083779	-3.202741
C	-2.786373	1.334707	-0.581700
C	-2.844103	2.566543	0.122774
C	-3.814645	1.094321	-1.513786
C	-3.852944	3.513346	-0.113465
C	-4.816513	2.046300	-1.778894
H	-3.871613	0.133087	-2.042743
C	-4.836983	3.262166	-1.078520
H	-3.831492	4.441460	0.468425
H	-5.590179	1.830001	-2.526451
H	-5.620278	4.002058	-1.279162
C	-1.774111	2.867739	1.135007
N	-0.838922	1.880929	1.181447
C	0.320511	2.102332	2.012458
H	0.765610	3.103646	1.830269
H	0.073460	2.085519	3.094388
C	1.304175	1.016950	1.693515
C	2.629135	0.802484	2.040820
N	0.933881	-0.028891	0.891011
N	1.932206	-0.862277	0.723679
N	2.970888	-0.364126	1.418880
H	3.328323	1.372779	2.647505
O	-1.736354	3.915630	1.809173
Fe	-1.219986	0.144753	0.172698
C	-0.481503	3.128998	-2.009046
C	-0.126202	4.102927	-1.051945
C	-1.497663	3.428760	-2.938032
C	-0.756532	5.351916	-1.036963
H	0.616612	3.869145	-0.284091
C	-2.127544	4.678752	-2.920048
H	-1.831326	2.676535	-3.658695
C	-1.758578	5.643618	-1.972031
H	-0.489260	6.079063	-0.264775
H	-2.929177	4.888414	-3.635629
H	-2.268924	6.612073	-1.946787
C	2.019872	1.595722	-1.954579
C	2.732065	2.809949	-2.016202
C	2.746933	0.382340	-1.989427
C	4.131364	2.810236	-2.119640
H	2.190747	3.760443	-2.008909
C	4.140210	0.386638	-2.118677
H	2.218339	-0.574258	-1.922360
C	4.841455	1.602246	-2.178901
H	4.666746	3.764519	-2.175110
H	4.679043	-0.566404	-2.169582
H	5.932620	1.606931	-2.276898
C	-2.759819	-0.903293	1.021028
C	-1.722960	-1.047764	1.756937
C	-4.202182	-1.133887	0.785893
C	-4.801911	-2.127483	1.798604
H	-4.356411	-1.501853	-0.244771
H	-4.727126	-0.161098	0.827961
C	-6.271681	-2.441447	1.503570
H	-4.204120	-3.056743	1.780865
H	-4.698084	-1.707706	2.817381
H	-6.689699	-3.144498	2.245570
H	-6.887552	-1.523932	1.521672
H	-6.380257	-2.897667	0.502860
C	-1.251821	-1.404869	3.114871
C	4.265262	-1.048868	1.375602
H	4.381199	-1.659635	2.289772
H	4.196626	-1.733522	0.515477
C	5.434385	-0.073991	1.209163
C	6.744424	-0.795288	0.867230
H	5.568057	0.515062	2.136003
H	5.183503	0.639764	0.405373
C	7.923267	0.168868	0.678844
H	6.989791	-1.529747	1.659859
H	6.600728	-1.381760	-0.061835
C	9.235129	-0.533174	0.301065
H	7.663074	0.908330	-0.104381
H	8.072589	0.751450	1.609596

H	9.490549	-1.271839	1.085895
H	9.078581	-1.116830	-0.627277
C	10.405698	0.437212	0.106827
H	10.605835	1.009793	1.030822
H	10.189227	1.166008	-0.695529
C	0.375456	-2.560021	-2.244366
C	0.943173	-2.647673	-3.531817
C	0.964442	-3.285279	-1.188760
C	2.086886	-3.427864	-3.752454
H	0.496096	-2.110422	-4.373690
C	2.091752	-4.083204	-1.417143
H	0.535337	-3.220100	-0.186605
C	2.665137	-4.147086	-2.696213
H	2.521883	-3.479223	-4.756370
H	2.529828	-4.650125	-0.588523
H	3.556322	-4.759220	-2.871555
C	-2.479430	-2.669771	-1.862415
C	-2.560429	-3.610170	-0.812206
C	-3.511253	-2.614631	-2.820006
C	-3.648126	-4.488165	-0.739356
H	-1.797435	-3.637665	-0.027534
C	-4.608487	-3.483912	-2.727980
H	-3.472209	-1.891132	-3.639517
C	-4.678647	-4.423942	-1.690484
H	-3.698527	-5.213053	0.079749
H	-5.409056	-3.425234	-3.473154
H	-5.536006	-5.101637	-1.620233
O	0.103505	-1.965706	3.127394
C	0.260624	-3.157361	2.517948
O	-0.638956	-3.758446	1.955232
C	1.694041	-3.633026	2.595439
H	1.714376	-4.732423	2.571358
H	2.221390	-3.245120	1.707358
H	2.197388	-3.249337	3.495909
H	-1.906698	-2.194916	3.531626
C	-1.226912	-0.186647	4.034466
C	-2.175471	0.838794	3.870967
C	-0.257064	-0.068108	5.045643
C	-2.146519	1.972430	4.692653
H	-2.911394	0.772047	3.065962
C	-0.235081	1.062739	5.875091
H	0.489985	-0.856569	5.168336
C	-1.174959	2.088286	5.697488
H	-2.860055	2.782125	4.515739
H	0.529272	1.146725	6.655720
H	-1.142884	2.981281	6.330623
H	11.332181	-0.097819	-0.165197

^{5}TS(2-3)

Lowest frequency = -280.7385 cm^{-1}

Charge = 0, Multiplicity = 5

126

P	-0.215353	1.847183	-1.711680
P	-0.897180	-1.314515	-1.985420
C	-0.305759	1.019721	-3.390038
H	-0.545946	1.753414	-4.180261
H	0.716014	0.649141	-3.589126
C	-1.297151	-0.154822	-3.389962
H	-1.270194	-0.688730	-4.357070
H	-2.328268	0.200673	-3.215040
C	-3.004415	0.551146	-0.043911
C	-3.285487	1.746280	0.679305
C	-3.916939	0.182597	-1.059668
C	-4.458956	2.477473	0.442345
C	-5.052316	0.954813	-1.343036
H	-3.768002	-0.747679	-1.617985
C	-5.345376	2.091226	-0.571039
H	-4.624275	3.374859	1.048363
H	-5.724830	0.650484	-2.154167
H	-6.249259	2.677158	-0.770645
C	-2.250770	2.310165	1.604775
N	-1.026397	1.780603	1.340774
C	0.060840	2.271767	2.158326
H	0.314778	3.328467	1.912747
H	-0.207329	2.279391	3.233208
C	1.252629	1.396779	1.922484
C	2.481026	1.293258	2.557850
N	1.237983	0.453491	0.930485
N	2.370391	-0.213736	0.917173
N	3.130119	0.286053	1.903989
H	2.918338	1.819237	3.403559
O	-2.487920	3.201561	2.444980
Fe	-0.873263	0.128140	0.134186
C	-1.447949	3.190450	-1.757022
C	-1.429937	4.139950	-0.711277
C	-2.490365	3.239513	-2.702235
C	-2.418557	5.124747	-0.626754
H	-0.650389	4.093646	0.053404
C	-3.482242	4.225618	-2.611860
H	-2.544778	2.502601	-3.509093
C	-3.448423	5.170634	-1.578074
H	-2.397667	5.834941	0.205337
H	-4.293076	4.243640	-3.347629
H	-4.233382	5.930475	-1.502781
C	1.421235	2.676566	-1.813876
C	1.604768	4.070628	-1.912004
C	2.560480	1.842254	-1.756222
C	2.896622	4.614637	-1.948529
H	0.735023	4.732028	-1.968329
C	3.847601	2.388296	-1.813738
H	2.435084	0.759087	-1.661660
C	4.021847	3.778749	-1.901896
H	3.022265	5.700200	-2.026080
H	4.718751	1.725148	-1.783579
H	5.029122	4.207565	-1.936744
C	-2.506955	-1.046699	1.060270
C	-1.318807	-1.171348	1.607579
C	-3.832755	-1.749776	1.133005
C	-3.818992	-2.956844	2.083481
H	-4.107600	-2.091637	0.117589
H	-4.623004	-1.030167	1.421442
C	-5.130108	-3.748315	2.025440
H	-2.968938	-3.611090	1.818283
H	-3.638415	-2.607235	3.117724
H	-5.112934	-4.606598	2.720213
H	-5.993886	-3.112951	2.293719
H	-5.307681	-4.139131	1.006765
C	-0.747270	-1.801436	2.824161
C	4.483513	-0.232095	2.108654
H	4.857543	0.208656	3.046585
H	4.406847	-1.323052	2.252850
C	5.400709	0.101554	0.927053
C	6.817143	-0.459325	1.096116
H	5.435461	1.199343	0.801473
H	4.939113	-0.304862	0.009148
C	7.725073	-0.147920	-0.101740
H	7.273104	-0.049338	2.019107
H	6.764924	-1.556746	1.241246
C	9.149080	-0.702225	0.045491

H	7.264550	-0.558223	-1.022729
H	7.771326	0.949662	-0.248015
H	9.603963	-0.291206	0.967921
H	9.097404	-1.798683	0.193548
C	10.047488	-0.386875	-1.155756
H	10.143307	0.704030	-1.305632
H	9.632701	-0.815220	-2.086474
C	0.664703	-2.092428	-2.570248
C	0.729479	-2.836846	-3.765490
C	1.822954	-1.928819	-1.790577
C	1.945078	-3.393950	-4.180678
H	-0.176102	-2.991597	-4.363292
C	3.040202	-2.485773	-2.211539
H	1.773823	-1.372771	-0.849915
C	3.103976	-3.215470	-3.406109
H	1.990355	-3.974038	-5.108734
H	3.937499	-2.350981	-1.597222
H	4.052818	-3.653589	-3.734067
C	-2.110429	-2.676544	-2.139479
C	-2.049156	-3.688618	-1.158195
C	-3.111897	-2.732182	-3.130581
C	-2.973829	-4.739404	-1.175987
H	-1.293861	-3.648638	-0.366262
C	-4.042559	-3.781506	-3.133661
H	-3.180037	-1.954700	-3.897650
C	-3.976204	-4.786297	-2.157595
H	-2.916460	-5.516115	-0.406377
H	-4.821617	-3.812113	-3.903020
H	-4.706468	-5.602519	-2.160762
O	0.641874	-2.227911	2.590858
C	0.802417	-3.322630	1.823969
O	-0.119211	-3.984141	1.375071
C	2.269058	-3.610567	1.591334
H	2.694131	-2.786604	0.994362
H	2.807986	-3.646725	2.553148
H	2.377240	-4.562409	1.053756
H	-1.285156	-2.712994	3.147305
C	-0.698861	-0.777433	3.956036
C	-1.764901	0.123750	4.127380
C	0.404026	-0.700690	4.824667
C	-1.729115	1.089881	5.140472
H	-2.611166	0.096369	3.436281
C	0.439232	0.264236	5.842222
H	1.240727	-1.390631	4.687356
C	-0.623890	1.165460	6.001038
H	-2.549708	1.808565	5.221769
H	1.306657	0.315261	6.510553
H	-0.587683	1.928862	6.785794
H	11.062617	-0.798399	-1.018818

[5]I-3
Lowest frequency = 11.1375 cm^{-1}
Charge = 0, Multiplicity = 5

126

P	-0.894998	-0.567746	1.606085
P	1.358671	-2.151295	0.290543
C	0.021047	-1.722102	2.771530
H	0.290768	-1.127179	3.659198
H	-0.674759	-2.508954	3.109244
C	1.301606	-2.324563	2.141186
H	1.429939	-3.375677	2.452691
H	2.186345	-1.762137	2.485256
C	3.816180	0.427736	1.024604
C	2.998144	1.068343	1.992943
C	4.710377	-0.575154	1.458919
C	3.074591	0.667302	3.342174
C	4.770589	-0.973453	2.799228
H	5.345440	-1.075989	0.720922
C	3.944054	-0.352462	3.749228
H	2.464584	1.193963	4.084981
H	5.456251	-1.773375	3.098810
H	3.988056	-0.650824	4.802573
C	2.185664	2.317591	1.688104
N	0.891765	2.077177	1.381037
C	0.038150	3.251482	1.255134
H	-0.367497	3.556941	2.240897
H	0.614313	4.116105	0.873283
C	-1.091286	2.904627	0.337114
C	-2.329365	3.461827	0.054975
N	-0.987713	1.775047	-0.429332
N	-2.063468	1.601179	-1.162196
N	-2.881943	2.626482	-0.876818
H	-2.845916	4.342203	0.430305
O	2.737939	3.429474	1.790039
Fe	0.707261	0.492155	0.019654
C	-1.719217	0.609216	2.741828
C	-3.065492	0.980235	2.548833
C	-0.961421	1.285096	3.721838
C	-3.643549	1.994419	3.324243
H	-3.661254	0.471720	1.785752
C	-1.544600	2.290833	4.501824
H	0.097951	1.046217	3.848475
C	-2.887240	2.652497	4.304970
H	-4.692595	2.267765	3.162739
H	-0.942649	2.804118	5.259336
H	-3.339069	3.443625	4.912824
C	-2.308533	-1.577985	0.993371
C	-3.198005	-2.258079	1.850584
C	-2.487722	-1.662059	-0.400539
C	-4.249230	-3.016020	1.319975
H	-3.074331	-2.184924	2.937399
C	-3.541899	-2.422185	-0.927804
H	-1.791588	-1.141419	-1.064207
C	-4.421155	-3.099964	-0.072083
H	-4.937478	-3.541859	1.990935
H	-3.667726	-2.492602	-2.013421
H	-5.240360	-3.698297	-0.485998
C	3.731603	0.800977	-0.419933
C	2.501945	0.771960	-1.009407
C	5.050668	1.211870	-1.052044
C	5.472734	2.641623	-0.650633
H	5.005208	1.135482	-2.154418
H	5.839069	0.505039	-0.727786
C	6.895521	2.994059	-1.095369
H	4.753667	3.357650	-1.089160
H	5.370118	2.754382	0.444066
H	7.160748	4.028185	-0.812522
H	7.635985	2.317031	-0.630447
H	7.007730	2.905450	-2.192292
C	2.329175	1.224638	-2.432444
C	-4.242628	2.588472	-1.415319
H	-4.673354	3.597660	-1.312595
H	-4.155829	2.355845	-2.489449
C	-5.076682	1.522189	-0.691583
C	-6.442474	1.256166	-1.332571
H	-5.205400	1.825613	0.363942
H	-4.486144	0.588550	-0.684875
C	-7.201716	0.130410	-0.615478
H	-7.054451	2.180126	-1.331813

H	-6.299627	0.980762	-2.396383
C	-8.552297	-0.215829	-1.256562
H	-6.562414	-0.773953	-0.592423
H	-7.359941	0.415219	0.443919
H	-9.193295	0.687444	-1.271560
H	-8.389658	-0.491860	-2.316959
C	-9.283260	-1.354726	-0.536243
H	-9.489300	-1.091744	0.517432
H	-8.674548	-2.277589	-0.534468
C	0.310612	-3.539245	-0.317458
C	-0.600455	-4.235746	0.502779
C	0.337636	-3.842050	-1.695778
C	-1.475053	-5.185174	-0.042853
H	-0.643272	-4.034958	1.575996
C	-0.529380	-4.796139	-2.238984
H	1.060354	-3.336029	-2.343959
C	-1.449532	-5.465175	-1.415521
H	-2.183083	-5.703217	0.612279
H	-0.485569	-5.020996	-3.310581
H	-2.136465	-6.203967	-1.841457
C	3.030266	-2.769464	-0.168780
C	3.589438	-2.321863	-1.381883
C	3.766797	-3.658553	0.638424
C	4.866618	-2.747120	-1.774711
H	3.037820	-1.611067	-2.001828
C	5.046022	-4.075065	0.248881
H	3.349897	-4.021158	1.582548
C	5.601487	-3.616509	-0.956235
H	5.292851	-2.383262	-2.715651
H	5.613050	-4.759105	0.889672
H	6.604640	-3.938354	-1.255820
O	1.775954	0.149015	-3.312889
C	0.626693	-0.464794	-3.030688
O	-0.008735	-0.357725	-1.984511
C	0.156935	-1.316121	-4.188999
H	-0.611059	-2.021771	-3.844019
H	-0.277401	-0.653992	-4.959334
H	1.003395	-1.850591	-4.648594
H	3.277969	1.422335	-2.966301
C	1.449131	2.467084	-2.523968
C	1.703825	3.532205	-1.638782
C	0.415174	2.601202	-3.464710
C	0.943369	4.706256	-1.704632
H	2.483345	3.435594	-0.876419
C	-0.371204	3.761398	-3.503482
H	0.214506	1.796276	-4.176665
C	-0.109042	4.820895	-2.625006
H	1.165702	5.527681	-1.015270
H	-1.186660	3.837640	-4.231487
H	-0.716511	5.731857	-2.660487
H	-10.247813	-1.587413	-1.020425

^{5}TS(3-4)
Lowest frequency = -328.2056 cm^{-1}
Charge = 0, Multiplicity = 5

126

P	0.918928	0.651033	1.557390
P	-1.378534	2.149136	0.192225
C	0.051288	1.914805	2.647253
H	-0.203222	1.402384	3.588906
H	0.775464	2.707922	2.898937
C	-1.233522	2.499839	2.011146
H	-1.308982	3.581973	2.215171
H	-2.122355	2.013727	2.447929
C	-3.662894	-0.306485	1.282755
C	-2.856959	-0.816912	2.337919
C	-4.590176	0.718626	1.590505
C	-3.015954	-0.291119	3.639723
C	-4.695929	1.265750	2.871709
H	-5.225086	1.105892	0.786718
C	-3.899563	0.756400	3.910509
H	-2.446785	-0.760261	4.449093
H	-5.402557	2.081452	3.058282
H	-3.985612	1.157408	4.926339
C	-1.944984	-2.023974	2.249669
N	-0.962792	-1.968586	1.323162
C	-0.073755	-3.124458	1.348563
H	0.296492	-3.322696	2.372950
H	-0.611714	-4.050667	1.050909
C	1.061705	-2.850395	0.413552
C	2.291123	-3.444872	0.174484
N	0.977922	-1.769256	-0.420569
N	2.059755	-1.658429	-1.156383
N	2.862336	-2.675800	-0.802416
H	2.794630	-4.303477	0.613143
O	-2.098956	-2.948538	3.073259
Fe	-0.717016	-0.409027	-0.040331
C	1.639408	-0.510582	2.775060
C	2.950387	-1.004634	2.616566
C	0.824364	-1.058696	3.787741
C	3.434534	-2.017183	3.455586
H	3.591011	-0.593204	1.831462
C	1.308929	-2.071660	4.622888
H	-0.205939	-0.714098	3.909092
C	2.616460	-2.556716	4.459588
H	4.458064	-2.386151	3.322696
H	0.650765	-2.495827	5.387604
H	2.993414	-3.352811	5.110357
C	2.401252	1.540355	0.921553
C	3.313307	2.209534	1.763774
C	2.606317	1.547875	-0.471471
C	4.412728	2.884071	1.217987
H	3.168757	2.193808	2.850366
C	3.711298	2.221628	-1.012592
H	1.889333	1.039277	-1.123821
C	4.612107	2.891385	-0.173204
H	5.117989	3.402872	1.876614
H	3.858804	2.231878	-2.097779
H	5.470411	3.423240	-0.598642
C	-3.680629	-0.827218	-0.123337
C	-2.595225	-0.719571	-0.936332
C	-4.970007	-1.528798	-0.516722
C	-4.976439	-2.993532	-0.025121
H	-5.094376	-1.513634	-1.616236
H	-5.840576	-1.002954	-0.079685
C	-6.280243	-3.729757	-0.348193
H	-4.119929	-3.519555	-0.485555
H	-4.792247	-3.006732	1.065566
H	-6.249353	-4.774545	0.008341
H	-7.146048	-3.235711	0.130028
H	-6.468108	-3.750397	-1.437781
C	-2.604113	-1.059444	-2.336282
C	4.235703	-2.677885	-1.311077
H	4.634532	-3.699826	-1.204457
H	4.175488	-2.437725	-2.384936
C	5.086589	-1.639310	-0.566786
C	6.470358	-1.412610	-1.184331
H	5.189035	-1.949801	0.489778
H	4.525063	-0.687927	-0.567494

C	7.255185	-0.318399	-0.446573
H	7.051487	-2.356253	-1.183103
H	6.351841	-1.123570	-2.247435
C	8.622340	-0.006919	-1.070105
H	6.643256	0.604384	-0.421071
H	7.393159	-0.619022	0.611260
H	9.236369	-0.928696	-1.087313
H	8.479167	0.283834	-2.129370
C	9.379037	1.102977	-0.331361
H	9.565856	0.824424	0.721884
H	8.798018	2.043522	-0.327333
C	-0.278705	3.405845	-0.587152
C	0.674912	4.145718	0.140895
C	-0.328047	3.569073	-1.988394
C	1.570173	5.002935	-0.513527
H	0.736912	4.052759	1.227756
C	0.561403	4.430055	-2.640189
H	-1.081408	3.029723	-2.571112
C	1.523052	5.143401	-1.906323
H	2.311509	5.555727	0.072707
H	0.502569	4.545440	-3.728086
H	2.225833	5.809156	-2.418304
C	-3.035645	2.803049	-0.262259
C	-3.617902	2.341917	-1.459924
C	-3.745274	3.718580	0.539299
C	-4.892988	2.782796	-1.841121
H	-3.076259	1.627801	-2.086686
C	-5.023521	4.148334	0.160443
H	-3.311730	4.089763	1.472218
C	-5.603466	3.677304	-1.027788
H	-5.336376	2.413921	-2.772288
H	-5.569895	4.852851	0.797051
H	-6.605417	4.010261	-1.319275
O	-1.724641	0.173620	-3.298929
C	-0.479241	0.436357	-3.032501
O	0.062975	0.342818	-1.913466
C	0.347727	0.867924	-4.229695
H	1.131226	1.573052	-3.913420
H	0.831240	-0.035839	-4.643733
H	-0.288021	1.310997	-5.011423
H	-3.509070	-0.825907	-2.919912
C	-1.938661	-2.318281	-2.789443
C	-1.442754	-3.235148	-1.844721
C	-1.823861	-2.619825	-4.162946
C	-0.837274	-4.426886	-2.260446
H	-1.550761	-2.992748	-0.783469
C	-1.213346	-3.809036	-4.578561
H	-2.201634	-1.903282	-4.899401
C	-0.714722	-4.714983	-3.628193
H	-0.459326	-5.132451	-1.512650
H	-1.126258	-4.032990	-5.647505
H	-0.238220	-5.645931	-3.954742
H	10.355187	1.311237	-0.803234

^{5}I-4

Lowest frequency = 8.7254 cm^{-1}
Charge = 0, Multiplicity = 5

126

P	0.939870	0.985604	1.421850
P	-1.892292	1.922435	0.099870
C	-0.084563	2.339784	2.238503
H	0.085720	2.326266	3.329396
H	0.313999	3.294013	1.854098
C	-1.589481	2.235515	1.922739
H	-2.094328	3.160817	2.251492
H	-2.054803	1.383546	2.450665
C	-3.822311	-0.991121	0.775017
C	-2.940486	-1.186763	1.874357
C	-5.104387	-0.452445	1.040662
C	-3.357297	-0.788945	3.164915
C	-5.486899	-0.039684	2.316005
H	-5.803093	-0.319630	0.210651
C	-4.596779	-0.189379	3.390766
H	-2.694015	-1.019289	4.004241
H	-6.477270	0.400945	2.469056
H	-4.884141	0.121016	4.401177
C	-1.695654	-2.045320	1.865177
N	-0.696186	-1.787701	0.985743
C	0.397063	-2.750319	1.155077
H	0.778816	-2.729388	2.194148
H	0.026226	-3.781742	1.003905
C	1.489169	-2.440107	0.190049
C	2.749060	-2.971440	-0.038688
N	1.338214	-1.400922	-0.681260
N	2.406950	-1.246691	-1.426416
N	3.268154	-2.203964	-1.044078
H	3.297598	-3.794415	0.413715
O	-1.641330	-2.952203	2.722018
Fe	-0.520316	-0.243799	-0.413889
C	1.393167	-0.115319	2.809120
C	2.657738	-0.739387	2.848502
C	0.417791	-0.485628	3.756674
C	2.937630	-1.706297	3.823323
H	3.421543	-0.467589	2.112984
C	0.695169	-1.463025	4.718953
H	-0.577576	-0.033065	3.722363
C	1.957044	-2.075625	4.756435
H	3.925318	-2.180036	3.847836
H	-0.084250	-1.761787	5.426172
H	2.171875	-2.844209	5.506015
C	2.544235	1.800801	1.030826
C	3.332332	2.422529	2.021965
C	2.989229	1.785733	-0.306903
C	4.549572	3.024532	1.679146
H	2.997279	2.423503	3.065183
C	4.208724	2.394713	-0.643541
H	2.364211	1.313084	-1.075024
C	4.989267	3.011569	0.344734
H	5.157505	3.505736	2.452951
H	4.547385	2.384306	-1.685436
H	5.940699	3.485307	0.078675
C	-3.517148	-1.428038	-0.611475
C	-2.336514	-1.287211	-1.222856
C	-4.645922	-2.078932	-1.411714
C	-5.203363	-3.353922	-0.753266
H	-4.265775	-2.311079	-2.420834
H	-5.466035	-1.346119	-1.549477
C	-6.344630	-3.980550	-1.561508
H	-4.380432	-4.083314	-0.635585
H	-5.552247	-3.116451	0.268374
H	-6.725215	-4.895784	-1.074976
H	-7.191152	-3.276781	-1.665147
H	-6.011590	-4.252446	-2.579863
C	-1.450293	-1.618712	-2.202549
C	4.643245	-2.151590	-1.539978
H	5.025650	-3.184010	-1.602514
H	4.585178	-1.738051	-2.559106
C	5.511535	-1.266876	-0.634215
C	6.913574	-1.006675	-1.195643

H	5.583869	-1.735080	0.365988
H	4.984652	-0.305804	-0.496227
C	7.744135	-0.089625	-0.286752
H	7.448352	-1.965562	-1.346181
H	6.823185	-0.543373	-2.198103
C	9.133033	0.243577	-0.848431
H	7.181684	0.848733	-0.113499
H	7.854029	-0.564703	0.708474
H	9.694544	-0.696397	-1.015882
H	9.017384	0.711116	-1.845937
C	9.943421	1.172331	0.062968
H	10.103069	0.715323	1.056667
H	9.417791	2.132176	0.219872
C	-1.266058	3.509007	-0.620458
C	0.122569	3.719811	-0.729327
C	-2.137085	4.536624	-1.035477
C	0.632424	4.932668	-1.209995
H	0.813720	2.916464	-0.476181
C	-1.626203	5.744262	-1.530878
H	-3.219323	4.392719	-0.976593
C	-0.241629	5.951599	-1.613710
H	1.717365	5.068375	-1.277617
H	-2.319142	6.528831	-1.853935
H	0.152383	6.898526	-1.998155
C	-3.695249	2.128085	-0.135298
C	-4.213054	1.718211	-1.382946
C	-4.550850	2.714087	0.815452
C	-5.575218	1.888749	-1.661952
H	-3.526700	1.294004	-2.122767
C	-5.912189	2.884571	0.527250
H	-4.166027	3.032930	1.788154
C	-6.428061	2.468256	-0.708503
H	-5.971619	1.570735	-2.632323
H	-6.572009	3.339491	1.273987
H	-7.492713	2.600098	-0.930380
O	-1.546084	1.365791	-2.963405
C	-0.309320	1.441984	-2.846961
O	0.370287	0.918446	-1.870218
C	0.528446	2.190041	-3.877069
H	0.921679	3.113044	-3.415989
H	1.391400	1.574388	-4.182755
H	-0.085317	2.456778	-4.750269
H	-1.249625	-0.883724	-2.992366
C	-0.749633	-2.907623	-2.273022
C	-1.107652	-4.000860	-1.452150
C	0.337299	-3.061370	-3.164530
C	-0.391665	-5.200602	-1.510391
H	-1.941272	-3.885926	-0.752406
C	1.056022	-4.259648	-3.215927
H	0.630640	-2.213716	-3.792434
C	0.698027	-5.335369	-2.386475
H	-0.682193	-6.035884	-0.863762
H	1.902091	-4.356579	-3.905701
H	1.259970	-6.274831	-2.427136
H	10.934721	1.397523	-0.367599

^{5}TS(4-5)

Lowest frequency = -268.3105 cm^{-1}

Charge = 0, Multiplicity = 5

126

P	-0.944802	-1.327437	1.193844
P	2.035272	-1.785043	0.064014
C	0.108401	-2.710380	1.898562
H	-0.173674	-2.914805	2.946431
H	-0.138728	-3.609411	1.308539
C	1.613922	-2.403855	1.782446
H	2.195971	-3.310898	2.021985
H	1.917425	-1.605673	2.482190
C	3.908338	1.217726	0.824430
C	3.130267	0.862473	1.966314
C	5.312424	0.999866	0.898356
C	3.763137	0.289481	3.094202
C	5.912723	0.421995	2.010836
H	5.938037	1.270109	0.045146
C	5.131629	0.043575	3.120312
H	3.135308	0.089872	3.967622
H	6.994550	0.253512	2.013522
H	5.597531	-0.415310	3.998786
C	1.733445	1.325512	2.191237
N	0.924421	1.590923	1.099783
C	-0.225473	2.437162	1.489309
H	-0.519569	2.145082	2.514515
H	0.078354	3.498533	1.542227
C	-1.379921	2.263989	0.565581
C	-2.615807	2.883434	0.455357
N	-1.361513	1.261063	-0.355995
N	-2.491354	1.206348	-1.022856
N	-3.256838	2.192280	-0.534010
H	-3.071396	3.723453	0.973280
O	1.354455	1.523883	3.364345
Fe	0.490127	0.169186	-0.541323
C	-1.590425	-0.454329	2.668985
C	-2.831164	0.213666	2.569056
C	-0.844871	-0.335222	3.856588
C	-3.311313	0.980292	3.635500
H	-3.420738	0.122186	1.651489
C	-1.330286	0.432315	4.923892
H	0.133390	-0.813031	3.949084
C	-2.560105	1.094324	4.817067
H	-4.276955	1.490490	3.545125
H	-0.726729	0.530534	5.831274
H	-2.933041	1.699627	5.650034
C	-2.476655	-2.163116	0.599610
C	-3.278986	-2.926352	1.473777
C	-2.876327	-1.988906	-0.740325
C	-4.464323	-3.511955	1.013143
H	-2.982410	-3.045937	2.521782
C	-4.067705	-2.577543	-1.194558
H	-2.255152	-1.397783	-1.422668
C	-4.862071	-3.335469	-0.322840
H	-5.082728	-4.103121	1.697278
H	-4.374318	-2.436360	-2.236768
H	-5.792438	-3.790335	-0.680649
C	3.328068	1.845424	-0.353230
C	1.997651	1.895819	-0.627390
C	4.242057	2.550834	-1.351651
C	4.833022	3.869938	-0.816809
H	3.668340	2.756131	-2.269401
H	5.060893	1.867135	-1.649163
C	5.789318	4.535383	-1.812323
H	3.996891	4.554491	-0.580901
H	5.355789	3.681168	0.139228
H	6.186765	5.486711	-1.416533
H	6.649978	3.878306	-2.036983
H	5.280083	4.753453	-2.768916
C	1.150057	2.199136	-1.678563
C	-4.646456	2.280559	-0.983984
H	-5.014752	3.287849	-0.731607
H	-4.633279	2.180977	-2.081353

C	-5.491678	1.171965	-0.341847
C	-6.917564	1.086465	-0.896795
H	-5.520069	1.329705	0.752969
H	-4.970938	0.212406	-0.511607
C	-7.713888	-0.069175	-0.274211
H	-7.451729	2.042147	-0.725272
H	-6.873502	0.950399	-1.995521
C	-9.132227	-0.218716	-0.841163
H	-7.154390	-1.013274	-0.425680
H	-7.769763	0.075935	0.823079
H	-9.689807	0.725350	-0.683706
H	-9.070949	-0.356973	-1.938469
C	-9.908609	-1.385085	-0.219215
H	-10.014235	-1.255463	0.873417
H	-9.387587	-2.344934	-0.390571
C	1.602508	-3.295733	-0.924727
C	0.259525	-3.519804	-1.287697
C	2.568179	-4.254791	-1.293451
C	-0.117057	-4.677154	-1.981593
H	-0.496280	-2.767211	-1.060691
C	2.192925	-5.406568	-1.998946
H	3.618974	-4.100091	-1.032295
C	0.850243	-5.625908	-2.341583
H	-1.169618	-4.824172	-2.246624
H	2.957866	-6.138358	-2.280950
H	0.561856	-6.528829	-2.890538
C	3.861281	-1.839624	-0.053987
C	4.432285	-1.351376	-1.251213
C	4.690451	-2.420305	0.922184
C	5.810896	-1.462701	-1.463302
H	3.777265	-0.910918	-2.009708
C	6.071516	-2.528639	0.700008
H	4.269694	-2.792633	1.859925
C	6.634229	-2.054563	-0.491817
H	6.245288	-1.086203	-2.395916
H	6.708061	-2.982020	1.467477
H	7.712738	-2.142096	-0.662429
O	1.773536	-0.561276	-2.611289
C	0.622924	-0.910151	-2.986919
O	-0.406565	-0.688495	-2.245898
C	0.421361	-1.630149	-4.304621
H	0.463880	-2.717133	-4.109841
H	-0.566142	-1.395577	-4.731945
H	1.225318	-1.372601	-5.010807
H	1.297412	1.613458	-2.595601
C	0.244664	3.344561	-1.732925
C	0.425833	4.471084	-0.892487
C	-0.837389	3.369791	-2.647720
C	-0.458289	5.553704	-0.933669
H	1.282823	4.481370	-0.209961
C	-1.719284	4.454098	-2.688548
H	-0.993043	2.505401	-3.301932
C	-1.544501	5.549464	-1.824916
H	-0.294590	6.413324	-0.273664
H	-2.554141	4.446360	-3.399283
H	-2.235671	6.398681	-1.859967
H	-10.922329	-1.470641	-0.648037

^{5}I-5
Lowest frequency = 9.9173 cm^{-1}
Charge = 0, Multiplicity = 5

126

P	0.860800	1.071262	1.237084
P	-1.943435	1.786930	0.124366
C	-0.275355	2.126432	2.308608
H	-0.063177	1.942881	3.375534
H	-0.035529	3.180132	2.085821
C	-1.756643	1.856755	1.980529
H	-2.391639	2.646840	2.419139
H	-2.096760	0.883313	2.379277
C	-3.866842	-1.335863	1.299981
C	-3.197810	-1.918375	2.422688
C	-5.157752	-0.772970	1.549103
C	-3.751778	-1.857469	3.719745
C	-5.690617	-0.722448	2.830856
H	-5.735783	-0.365845	0.717931
C	-4.981515	-1.243759	3.938813
H	-3.187057	-2.326869	4.530824
H	-6.679063	-0.272622	2.980468
H	-5.408608	-1.191925	4.945434
C	-1.965257	-2.679362	2.242898
N	-1.385200	-2.649839	0.952053
C	-0.161579	-3.455425	0.852181
H	0.016113	-3.792609	1.886818
H	-0.353233	-4.351000	0.236890
C	1.065936	-2.776173	0.328777
C	2.338174	-3.319476	0.197515
N	1.130234	-1.504703	-0.158682
N	2.346586	-1.230768	-0.576810
N	3.082683	-2.328886	-0.369745
H	2.748393	-4.296430	0.442909
O	-1.435544	-3.326997	3.160603
Fe	-0.409254	-0.019818	-0.741445
C	1.529408	-0.130216	2.451959
C	2.907287	-0.414119	2.523716
C	0.634281	-0.880365	3.239676
C	3.375422	-1.432265	3.364848
H	3.610293	0.167385	1.921592
C	1.104832	-1.894431	4.083830
H	-0.442017	-0.686891	3.190683
C	2.477125	-2.177255	4.144049
H	4.450572	-1.639494	3.416759
H	0.383989	-2.479689	4.660500
H	2.845051	-2.975759	4.797237
C	2.322952	2.136347	0.903531
C	2.838868	3.046815	1.846348
C	2.952795	1.999846	-0.350463
C	3.970030	3.814865	1.539135
H	2.362767	3.151961	2.827208
C	4.089355	2.765898	-0.649079
H	2.539561	1.300512	-1.086032
C	4.598831	3.673328	0.291845
H	4.365074	4.522474	2.276126
H	4.573569	2.657172	-1.625921
H	5.484249	4.273309	0.054452
C	-3.248487	-1.368742	0.009089
C	-1.990473	-2.008468	-0.165505
C	-4.001954	-0.903156	-1.219302
C	-4.600718	-2.056877	-2.049272
H	-3.346793	-0.289294	-1.860774
H	-4.816669	-0.226181	-0.922332
C	-5.298864	-1.549910	-3.315535
H	-3.795918	-2.764416	-2.322086
H	-5.312909	-2.621602	-1.418007
H	-5.735585	-2.379116	-3.899964
H	-6.112692	-0.844186	-3.065754
H	-4.585035	-1.013018	-3.966662
C	-1.302101	-1.925579	-1.428748
C	4.486387	-2.331836	-0.797300

H	4.896020	-3.324065	-0.549810
H	4.494324	-2.222856	-1.895708
C	5.276630	-1.200592	-0.132825
C	6.681377	-1.016575	-0.716894
H	5.337887	-1.395163	0.953011
H	4.700928	-0.267227	-0.257716
C	7.424652	0.160699	-0.069714
H	7.273590	-1.945092	-0.592634
H	6.603545	-0.844092	-1.808753
C	8.815406	0.418345	-0.665204
H	6.806010	1.074212	-0.169947
H	7.518935	-0.022019	1.019323
H	9.433835	-0.494385	-0.558286
H	8.716096	0.593236	-1.754510
C	9.535113	1.607127	-0.017973
H	9.678725	1.443492	1.065719
H	8.950583	2.537761	-0.138067
C	-1.195481	3.423854	-0.315642
C	0.030181	3.469831	-1.000758
C	-1.809316	4.630377	0.079780
C	0.652099	4.698857	-1.265583
H	0.508091	2.537650	-1.312999
C	-1.191601	5.857330	-0.191323
H	-2.776557	4.606574	0.594472
C	0.043602	5.893569	-0.860678
H	1.617622	4.713325	-1.781763
H	-1.674627	6.790196	0.119545
H	0.526951	6.854873	-1.066003
C	-3.700663	2.159981	-0.220794
C	-4.048854	2.362870	-1.575591
C	-4.693603	2.249227	0.770960
C	-5.372535	2.647897	-1.923276
H	-3.275005	2.265391	-2.345440
C	-6.021278	2.534263	0.413813
H	-4.446900	2.080593	1.822417
C	-6.363886	2.731921	-0.930036
H	-5.636518	2.796469	-2.975802
H	-6.788752	2.594908	1.193150
H	-7.401064	2.950563	-1.205836
O	-1.364243	0.910063	-2.735617
C	-0.166777	0.973690	-3.132780
O	0.803067	0.592041	-2.376032
C	0.159047	1.552215	-4.494392
H	0.207212	2.652485	-4.397060
H	1.134262	1.190561	-4.854207
H	-0.638747	1.310566	-5.213811
H	-1.836941	-1.310590	-2.163664
C	-0.547386	-3.010414	-2.102036
C	-0.944417	-4.365080	-2.009311
C	0.563145	-2.706119	-2.925547
C	-0.236116	-5.378640	-2.670110
H	-1.835108	-4.615708	-1.420702
C	1.263691	-3.716644	-3.593451
H	0.888160	-1.663318	-3.006092
C	0.878062	-5.061108	-3.461033
H	-0.565743	-6.419767	-2.577413
H	2.124947	-3.453639	-4.219034
H	1.431384	-5.851433	-3.980047
H	10.529296	1.772609	-0.468628

Propane
Lowest frequency = 226.8806 cm^{-1}
Charge = 0, Multiplicity = 1

11

C	-3.490524	-2.025809	0.665987
C	-3.700831	-0.974995	1.763387
H	-3.174576	-2.993845	1.096996
H	-4.424075	-2.203512	0.100704
C	-4.770191	-1.377504	2.786545
H	-2.742111	-0.793167	2.286472
H	-3.981768	-0.009986	1.298991
H	-4.899818	-0.603342	3.563525
H	-5.750214	-1.531963	2.298274
H	-4.500081	-2.322051	3.294074
H	-2.715806	-1.709827	-0.054969

Propene
Lowest frequency = 205.0763 cm^{-1}
Charge = 0, Multiplicity = 1

9

C	-3.540656	-2.014579	0.700286
C	-3.827083	-1.009531	1.780714
H	-3.068335	-2.923805	1.119268
H	-4.460214	-2.315504	0.169913
H	-2.829255	-1.606416	-0.043026
H	-2.956761	-0.659593	2.356382
C	-5.038700	-0.520389	2.085988
H	-5.176666	0.212967	2.888707
H	-5.939096	-0.836186	1.542748

H_2
Lowest frequency = 4406.9734 cm^{-1}
Charge = 0, Multiplicity = 1

2

H	-3.689758	-0.397561	0.000000
H	-4.447299	-0.397561	-0.000000

THF
Lowest frequency = 61.2347 cm^{-1}
Charge = 0, Multiplicity = 1

13

C	-5.863462	1.178350	1.372845
H	-6.477669	1.147993	2.288452
H	-6.231416	2.006070	0.739416
C	-4.359878	1.345082	1.643249
H	-4.050060	0.701287	2.486633
H	-4.063828	2.381698	1.876262
C	-5.882557	-0.142401	0.591986
H	-5.937118	-1.011411	1.282108
H	-6.726978	-0.218299	-0.117142
C	-3.760317	0.829660	0.328225
H	-3.689370	1.645274	-0.422881
H	-2.752997	0.391254	0.449725
O	-4.653680	-0.186775	-0.144868

Alkyne
Lowest frequency = 9.5664 cm^{-1}
Charge = 0, Multiplicity = 1

32

C	-1.969634	-1.843261	0.387325
C	-1.235297	-1.608686	-0.558525
C	-2.879615	-2.099955	1.503572
C	-4.303289	-1.538441	1.282990
H	-2.449983	-1.663117	2.426720
H	-2.937360	-3.191908	1.682266
C	-5.230058	-1.822379	2.468901
H	-4.231865	-0.449936	1.105232
H	-4.719380	-1.979085	0.358723
H	-6.239753	-1.414125	2.290521
H	-5.331962	-2.908633	2.646025
H	-4.841530	-1.367293	3.398239
C	-0.375485	-1.356001	-1.710921
O	0.577276	-0.291513	-1.328695
C	0.878106	0.610347	-2.298390
O	0.397279	0.596680	-3.416420
C	1.899887	1.604802	-1.794743
H	1.993354	2.430097	-2.514161
H	1.612432	1.982543	-0.800300
H	2.873967	1.095962	-1.687877
H	-0.963909	-0.952268	-2.552798
C	0.415221	-2.564327	-2.190888
C	0.869199	-3.542339	-1.288957
C	0.738746	-2.676258	-3.555728
C	1.639014	-4.619804	-1.745564
H	0.605942	-3.456050	-0.229485
C	1.506795	-3.756485	-4.011072
H	0.395539	-1.904339	-4.251965
C	1.958757	-4.729574	-3.107587
H	1.987731	-5.379205	-1.037356
H	1.751617	-3.839368	-5.075415
H	2.557049	-5.574841	-3.464490

References

[1] G. Cera, T. Haven, L. Ackermann, *Chem. Commun.* **2017**, *53*, 6460–6463.

[2] G. Cera, T. Haven, L. Ackermann, *Chem. Eur. J.* **2017**, *23*, 3577–3582.

[3] T. Schwier, M. Rubin, V. Gevorgyan, *Org. Lett.* **2004**, *6*, 1999–2001.

[4] S. Shi, M. Szostak, *Synthesis* **2017**; *49(16)*, 3602-3608.

[5] G. Winter, D. W. Thompson, J. R. Loehe, *Inorg. Synth.* **2007**, *14*, 99−104.

[6] E. Bill, E. Mfit Program; Max-Planck Institute for Chemical Energy Conversion: Mülheim/Ruhr, Germany, **2008**.

[7] a) P. Gütlich, E. Bill, A. X. Trautwein, *Mössbauer Spectroscopy and Transition Metal Chemistry*, Springer, Heidelberg, **2011**; b) P. Gütlich, Z. *Anorg. Allg. Chem.* **2012**, *638*, 15–43.

[8] S. B. Muñoz III, S. L. Daifuku, W. W. Brennessel, M. L. Neidig, *J. Am. Chem. Soc.* **2016**, *138*, 7492−7495.

[9] J. M. Tao, J. P. Perdew, V. N. Staroverov, G. E. Scuseria, *Phys. Rev. Lett.* **2003**, *91*, 146401.

[10] a) S. Grimme, S. Ehrlich, L. Goerigk, *J. Comput. Chem.* **2011**, *32*, 1456–1465; b) S. Grimme, J. Antony, S. Ehrlich, H. Krieg, *J. Chem. Phys.* **2010**, *132*, 154104.

[11] a) F. Weigend, *Phys. Chem. Chem. Phys.* **2006**, *8*, 1057–1065; b) F. Weigend, R. Ahlrichs, *Phys. Chem. Chem. Phys.* **2005**, *7*, 3297–3305; c) A. Schaefer, C. Huber, R. Ahlrichs, *J. Chem. Phys.* **1994**, *100*, 5829–583; d) A. Schaefer, H. Horn, R. Ahlrichs, *J. Chem. Phys.* **1992**, *97*, 2571–2577.

[12] Y. Zhao, D. G. Truhlar, *J. Phys. Chem. A* **2005**, *109*, 5656–5667.

[13] A. V. Marenich, C. J. Cramer, D. G. Truhlar, *J. Phys. Chem. B* **2009**, *113*, 6378–6396.

[14] M. J. Frisch, G. W. Trucks, H. B. Schlegel, G. E. Scuseria, M. A. Robb, J. R. Cheeseman, G. Scalmani, V. Barone, G. A. Petersson, H. Nakatsuji, X. Li, M. Caricato, A. V. Marenich, J. Bloino, B. G. Janesko, R. Gomperts, B. Mennucci, H. P. Hratchian, J. V. Ortiz, A. F. Izmaylov, J. L. Sonnenberg, D. Williams-Young, F. Ding, F. Lipparini, F. Egidi, J. Goings, B. Peng, A. Petrone, T. Henderson, D. Ranasinghe, V. G. Zakrzewski, J. Gao, N. Rega, G. Zheng, W. Liang, M. Hada, M. Ehara, K. Toyota, R. Fukuda, J. Hasegawa, M. Ishida, T. Nakajima, Y. Honda, O. Kitao, H. Nakai, T. Vreven, K. Throssell, J. A. Montgomery, Jr., J. E. Peralta, F. Ogliaro, M. J. Bearpark, J. J. Heyd, E. N. Brothers, K. N. Kudin, V. N. Staroverov, T. A. Keith, R. Kobayashi, J. Normand, K. Raghavachari, A. P. Rendell, J. C. Burant, S. S. Iyengar, J. Tomasi, M. Cossi, J. M. Millam, M. Klene, C. Adamo, R. Cammi, J. W. Ochterski, R. L. Martin, K. Morokuma, O. Farkas, J. B. Foresman, D. J. Fox, *Gaussian 16, Revision A.03*, Gaussian, Inc., Wallingford CT, **2016**.

[15] The *PyMOL* Molecular Graphics System, version 1.8.x, Schrödinger, LLC.

NMR spectra

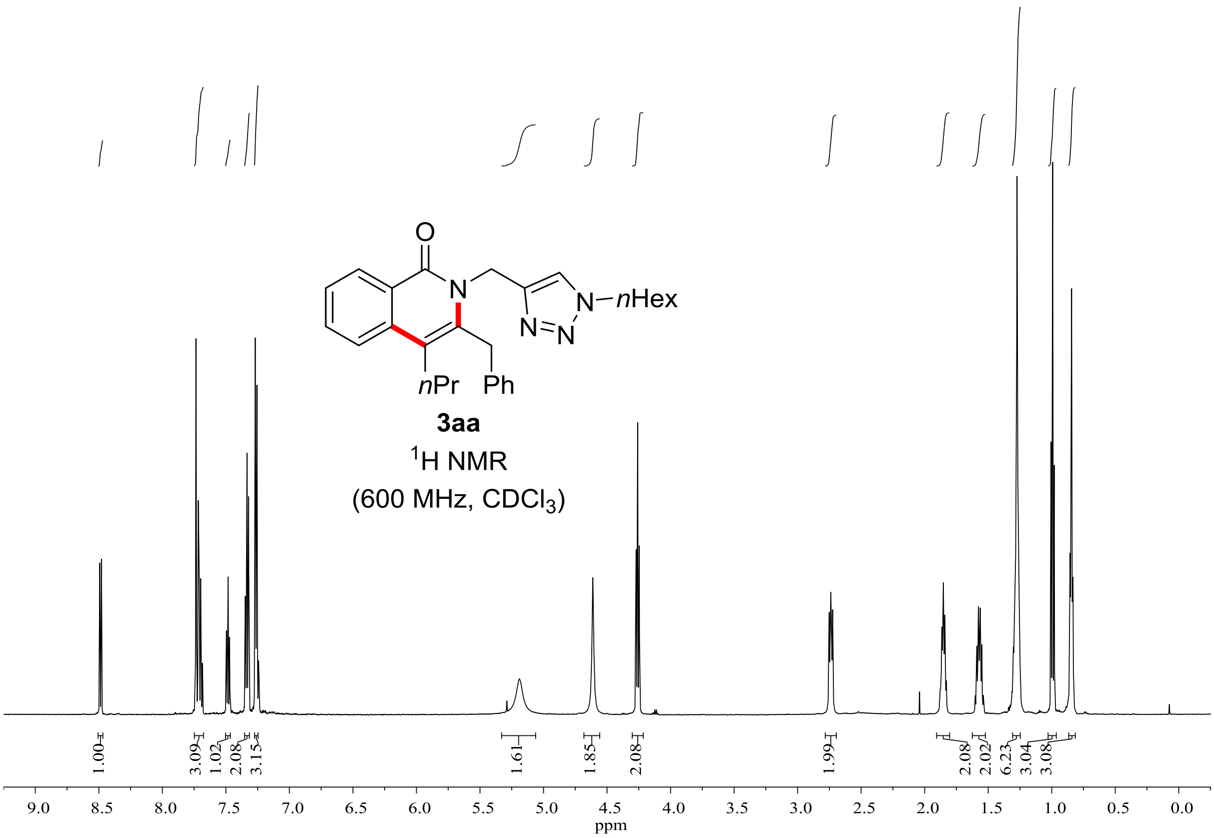

3aa
^{1}H NMR
(600 MHz, CDCl$_3$)
N
O
N−nHex
N=N
nPr
Ph
1.00
3.09
1.02
2.08
3.15
1.61
1.85
2.08
1.99
2.08
2.02
6.23
3.04
3.08
9.0 8.5 8.0 7.5 7.0 6.5 6.0 5.5 5.0 4.5 4.0 3.5 3.0 2.5 2.0 1.5 1.0 0.5 0.0
ppm

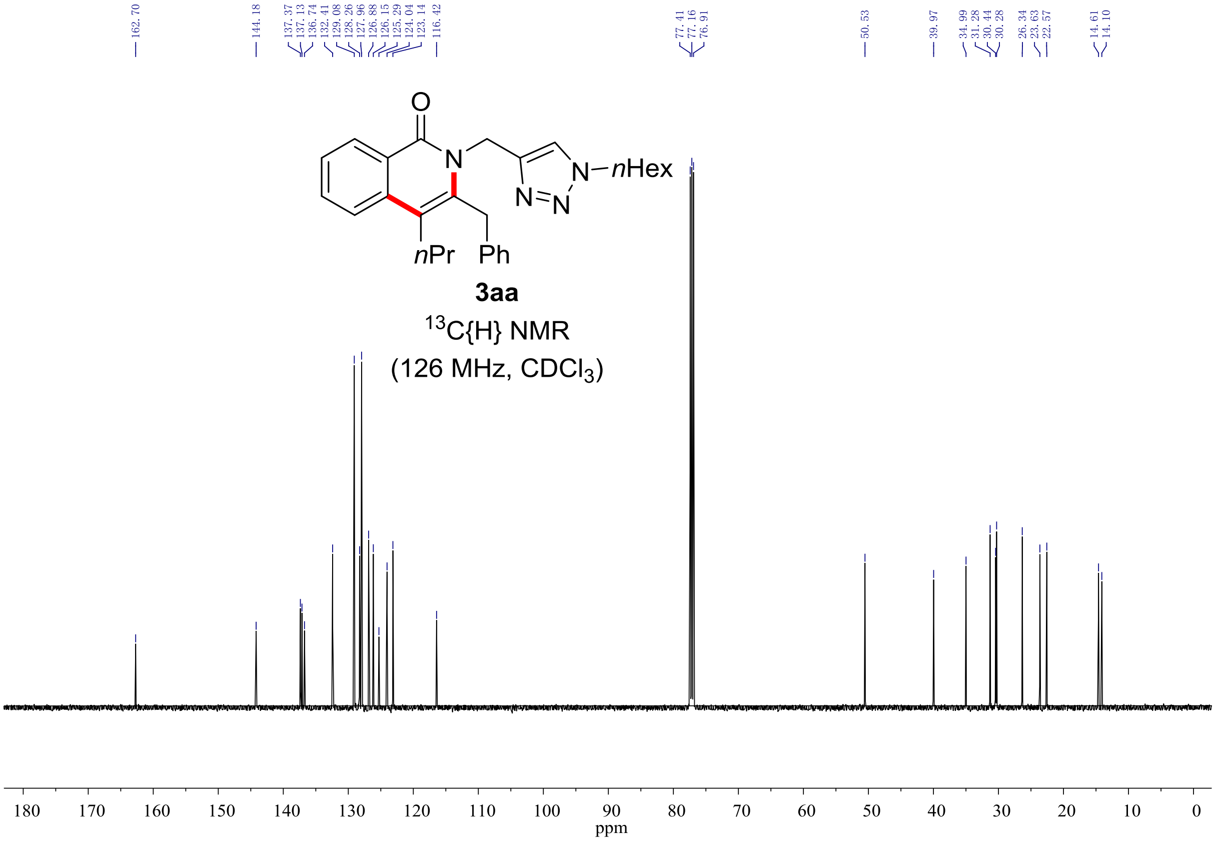

162.70
144.18
137.37
137.13
136.74
132.41
129.08
128.26
127.96
126.88
126.15
125.29
124.04
123.14
116.42
77.41
77.16
76.91
50.53
39.97
34.99
31.28
30.44
30.28
26.34
23.63
22.57
14.61
14.10
3aa
^{13}C{H} NMR
(126 MHz, CDCl$_3$)
N
O
N−nHex
N=N
nPr
Ph
180 170 160 150 140 130 120 110 100 90 80 70 60 50 40 30 20 10 0
ppm

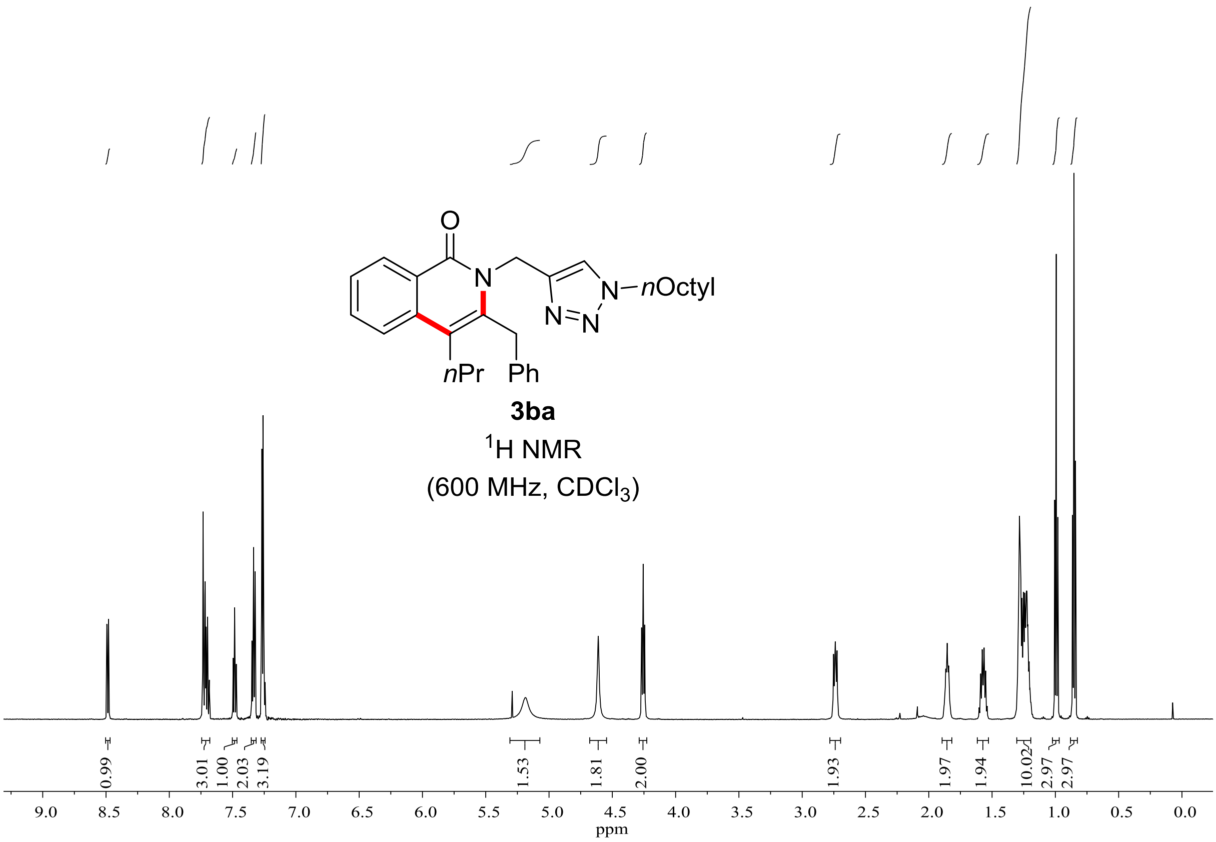

O
N
N
N
N
nOctyl
nPr
Ph
3ba
1H NMR
(600 MHz, CDCl3)
0.99
3.01
1.00
2.03
3.19
1.53
1.81
2.00
1.93
1.97
1.94
10.02
2.97
2.97
9.0
8.5
8.0
7.5
7.0
6.5
6.0
5.5
5.0
4.5
4.0
3.5
3.0
2.5
2.0
1.5
1.0
0.5
0.0
ppm

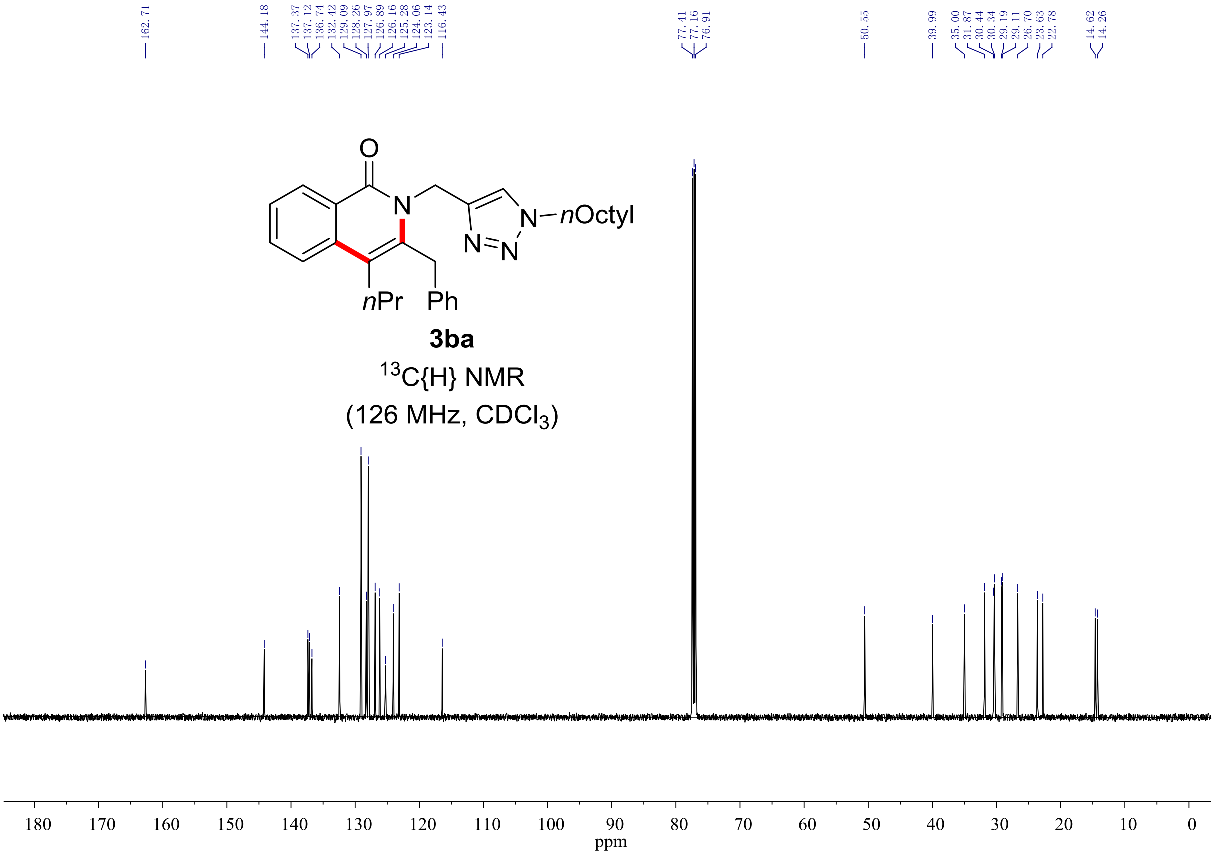

162.71
144.18
137.37
137.12
136.74
132.42
129.09
128.26
127.97
126.89
126.16
125.28
124.06
123.14
116.43
77.41
77.16
76.91
50.55
39.99
35.00
31.87
30.44
30.34
29.19
29.11
26.70
23.63
22.78
14.62
14.26
O
N
N
N
N
nOctyl
nPr
Ph
3ba
13C{H} NMR
(126 MHz, CDCl3)
180
170
160
150
140
130
120
110
100
90
80
70
60
50
40
30
20
10
0
ppm

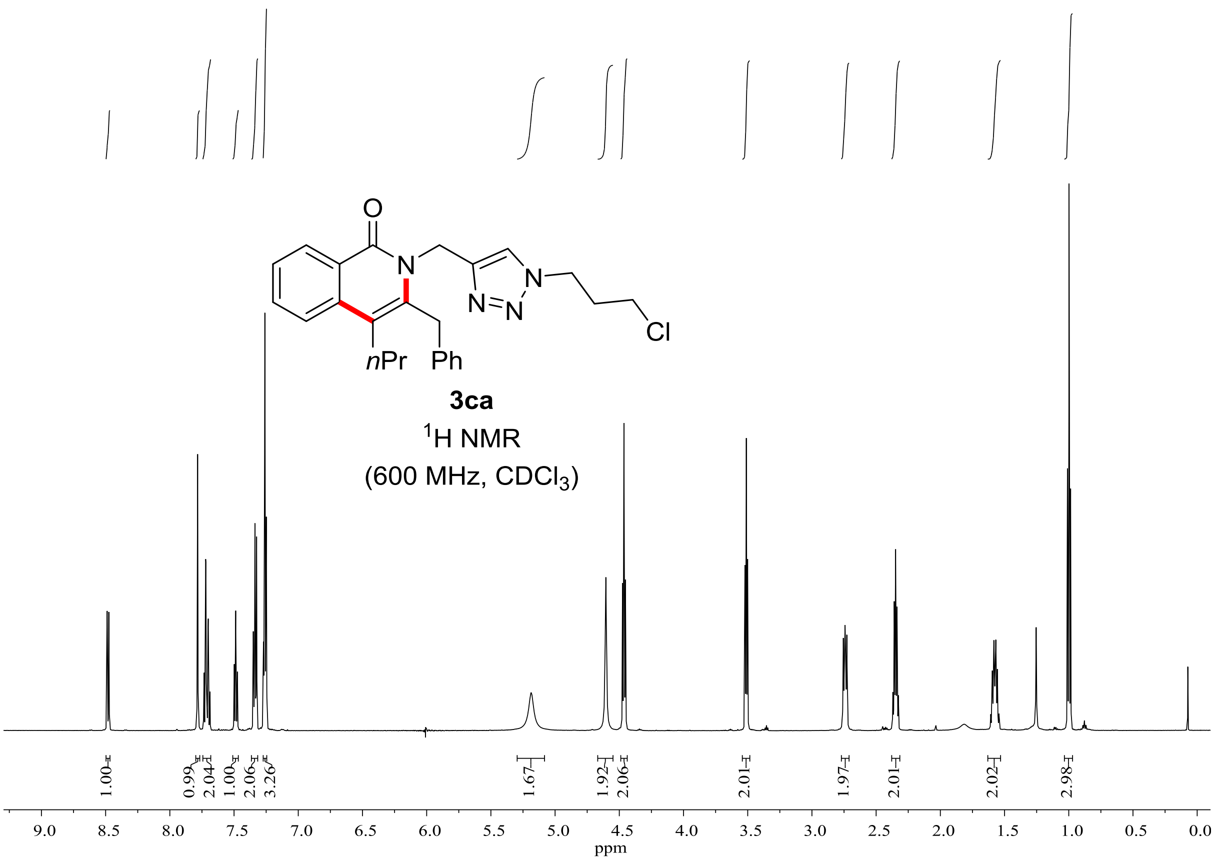

3ca

^{1}H NMR

(600 MHz, $CDCl_3$)

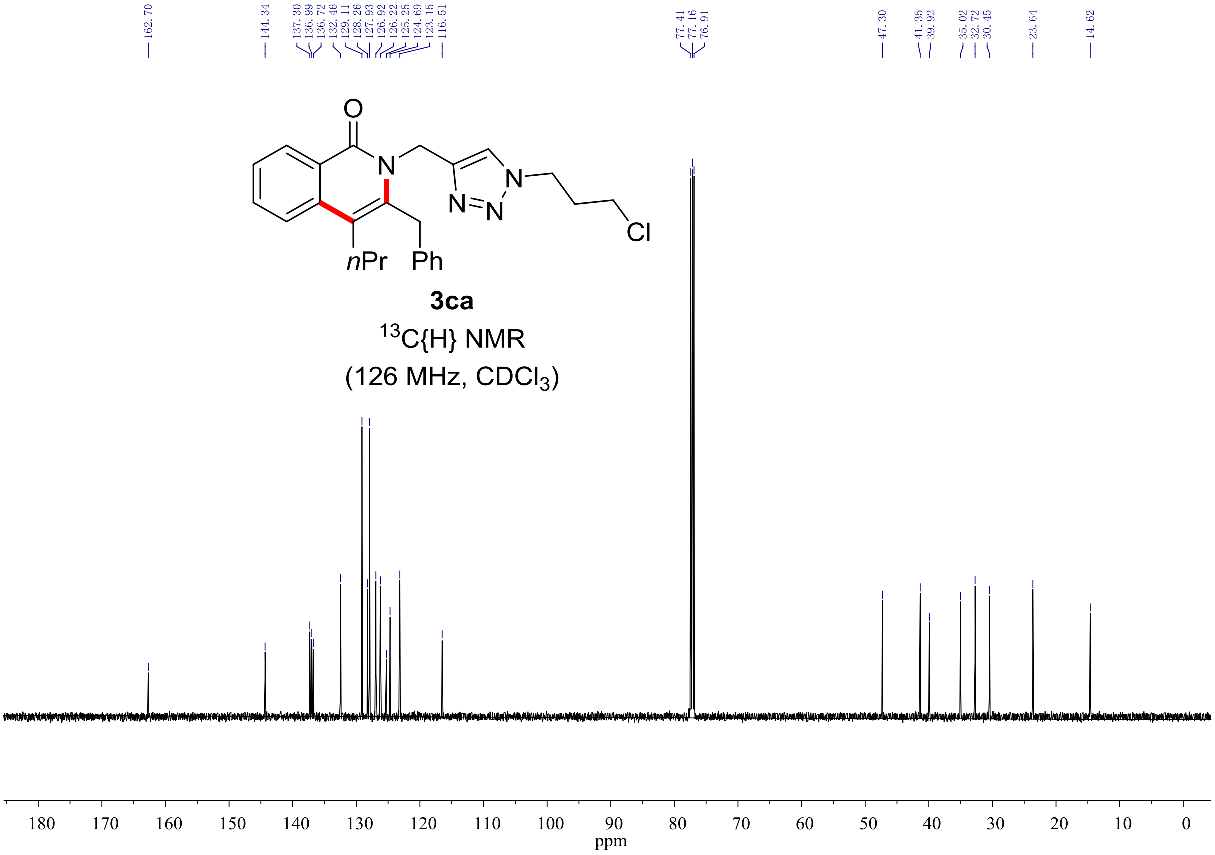

3ca

$^{13}C\{H\}$ NMR

(126 MHz, $CDCl_3$)

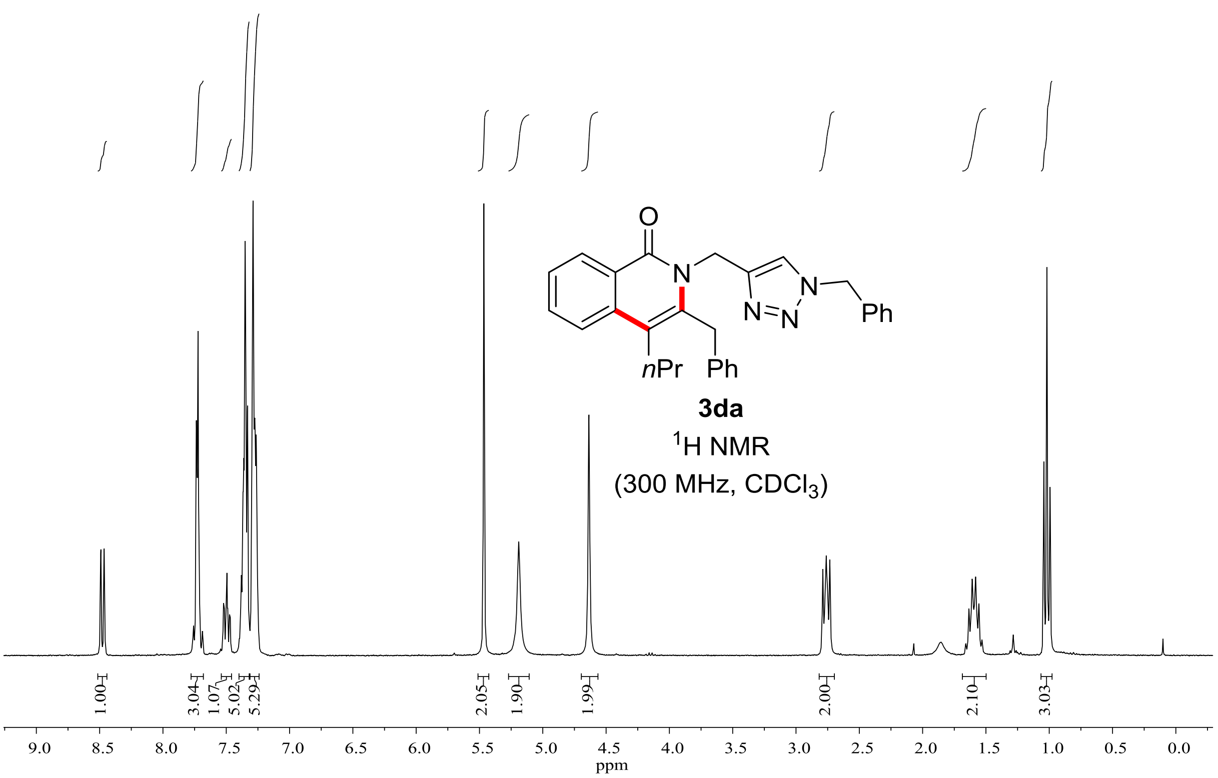

3da
^{1}H NMR
(300 MHz, CDCl$_3$)
ppm

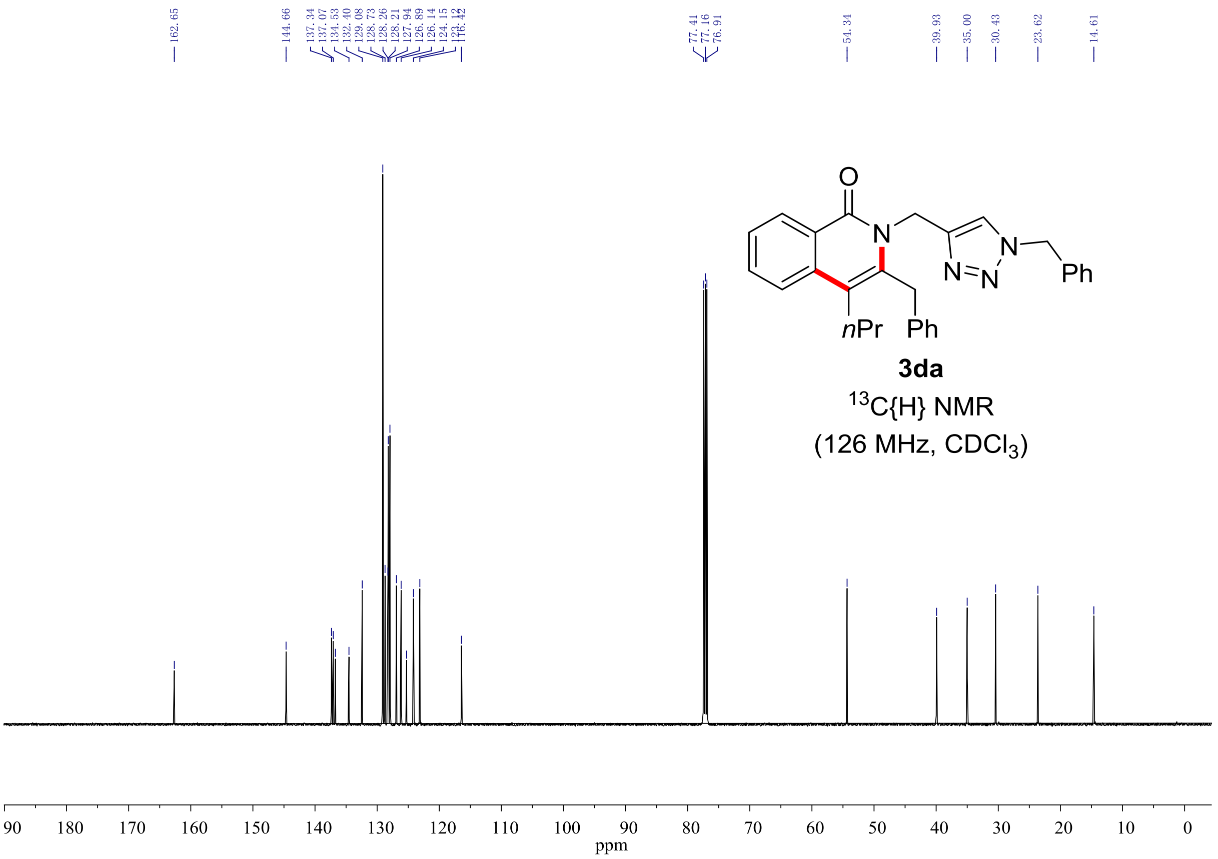

3da
^{13}C{H} NMR
(126 MHz, CDCl$_3$)
ppm

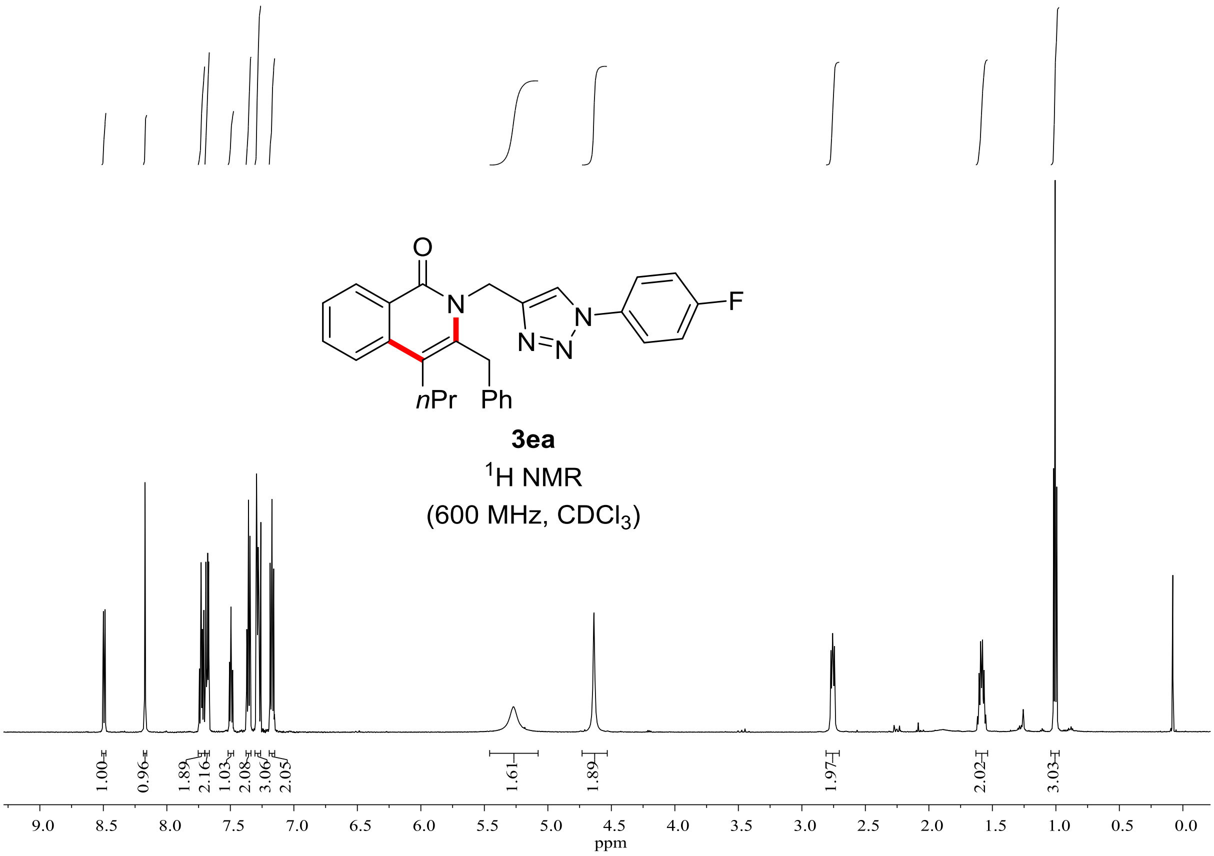
O
N
N
N=N
F
nPr
Ph
3ea
1H NMR
(600 MHz, CDCl3)
1.00
0.96
1.89
2.16
1.03
2.08
3.06
2.05
1.61
1.89
1.97
2.02
3.03
9.0 8.5 8.0 7.5 7.0 6.5 6.0 5.5 5.0 4.5 4.0 3.5 3.0 2.5 2.0 1.5 1.0 0.5 0.0
ppm

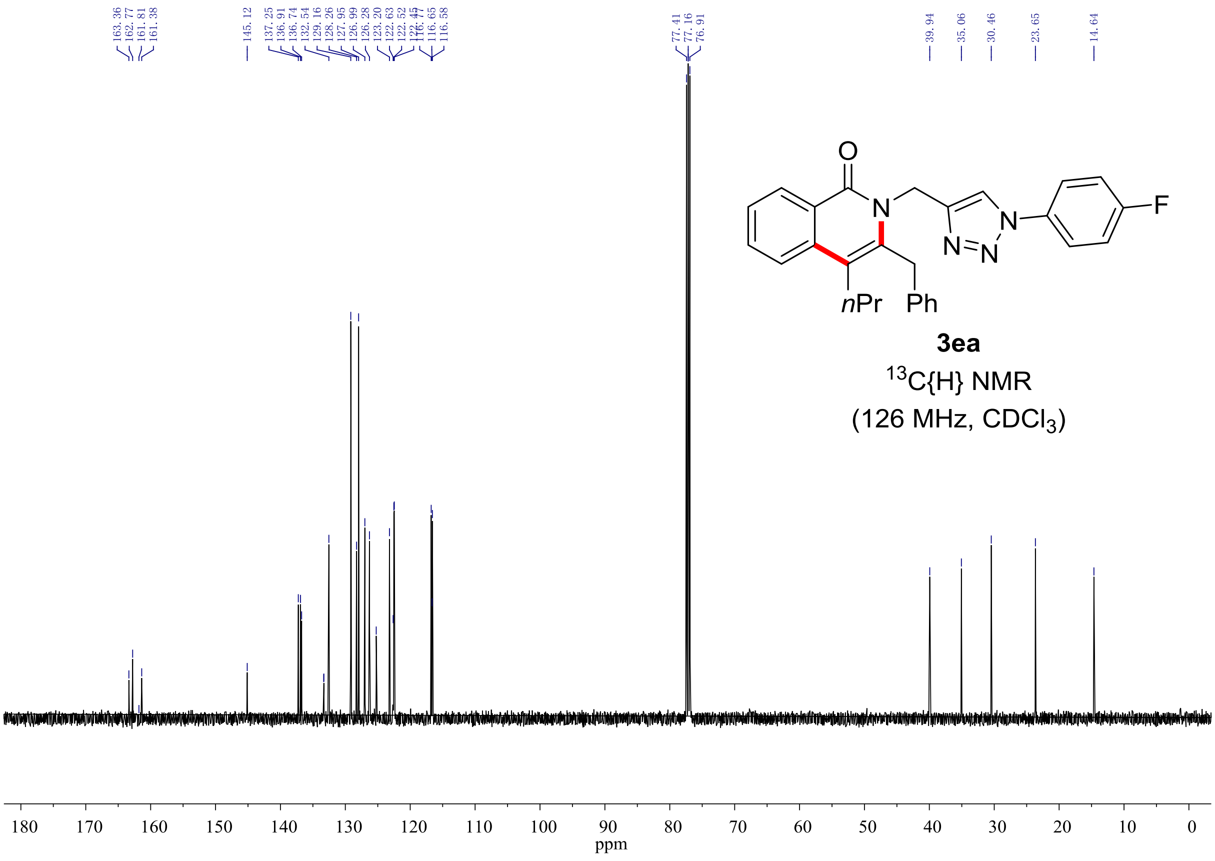
163.36
162.77
161.81
161.38
145.12
137.25
136.91
136.74
132.54
129.16
128.26
127.95
126.99
126.28
123.20
122.63
122.52
122.45
116.77
116.65
116.58
77.41
77.16
76.91
39.94
35.06
30.46
23.65
14.64
O
N
N
N=N
F
nPr
Ph
3ea
13C{H} NMR
(126 MHz, CDCl3)
180 170 160 150 140 130 120 110 100 90 80 70 60 50 40 30 20 10 0
ppm

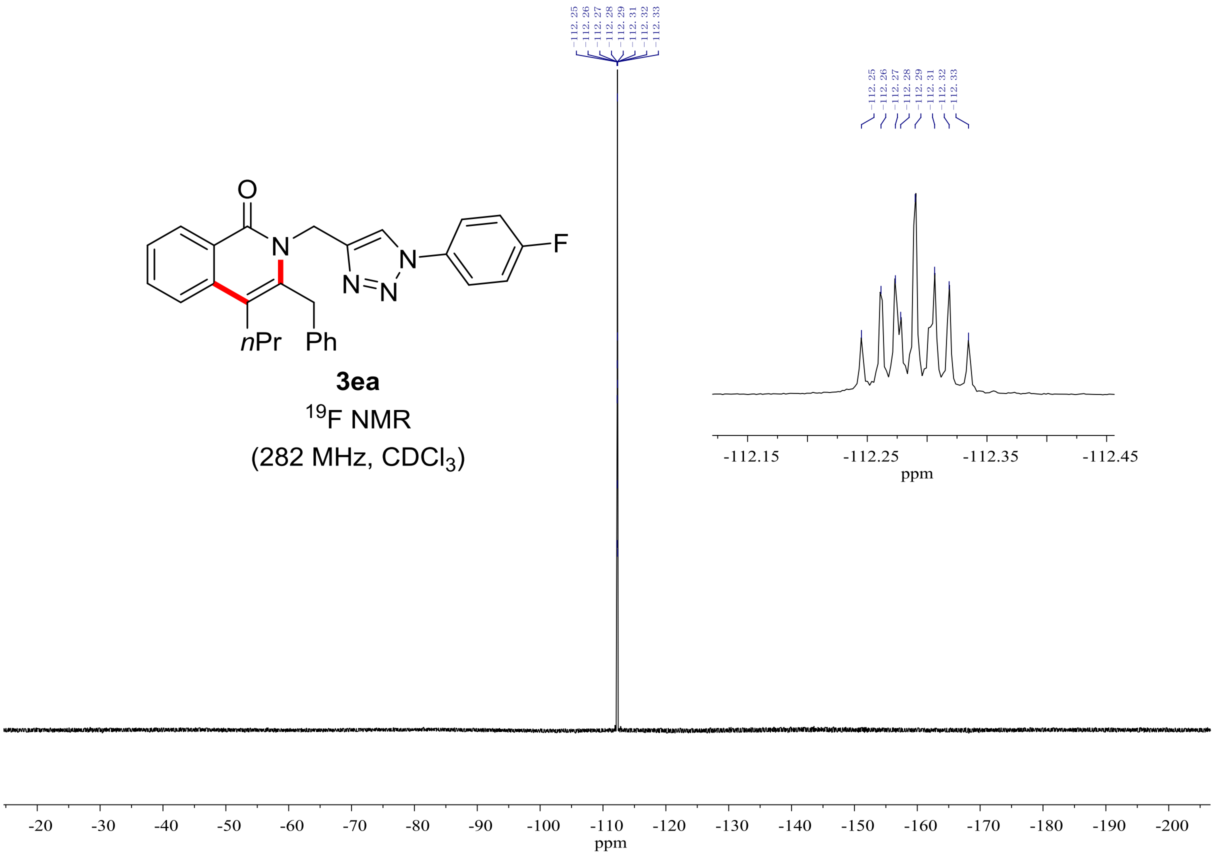

O
N
N
N
N
F
nPr
Ph
3ea
^{19}F NMR
(282 MHz, $CDCl_3$)
-112.25
-112.26
-112.27
-112.28
-112.29
-112.31
-112.32
-112.33
-112.15 -112.25 -112.35 -112.45
ppm
-20 -30 -40 -50 -60 -70 -80 -90 -100 -110 -120 -130 -140 -150 -160 -170 -180 -190 -200
ppm

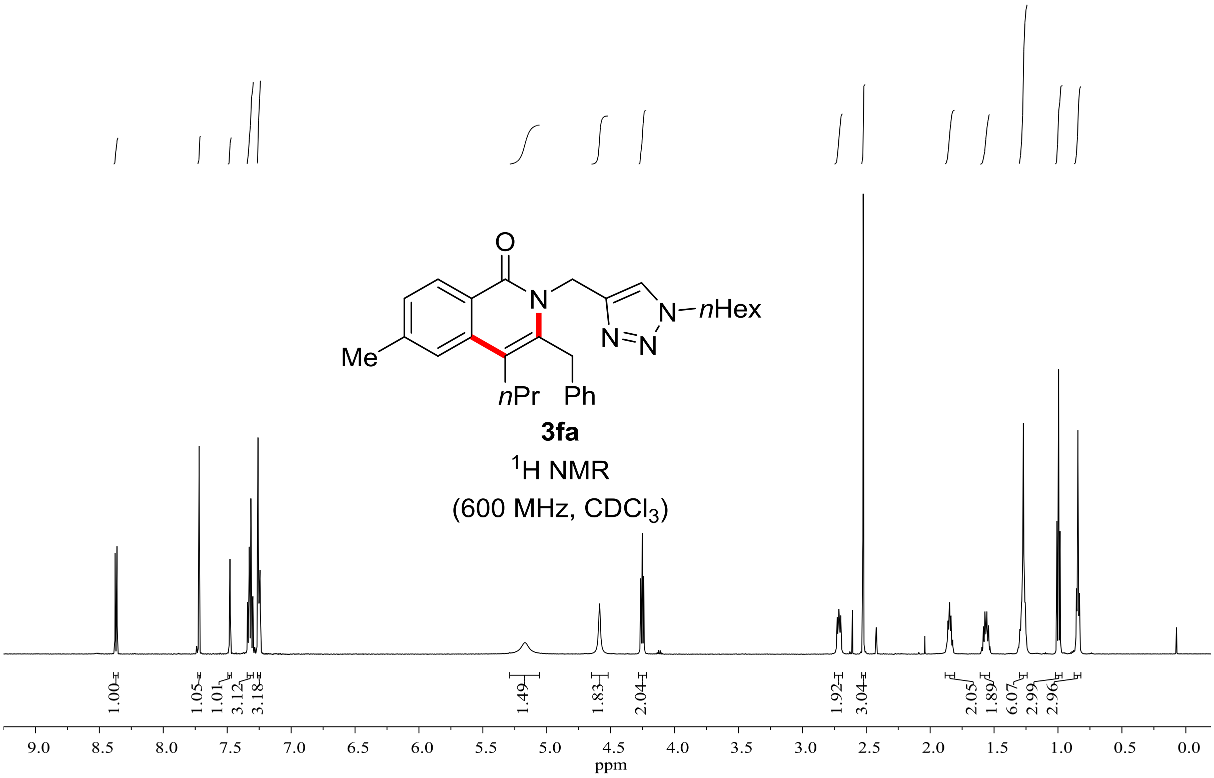

O
N
N
N=N
N−nHex
Me
nPr
Ph
3fa
1H NMR
(600 MHz, CDCl3)
1.00
1.05
1.01
3.12
3.18
1.49
1.83
2.04
1.92
3.04
2.05
1.89
6.07
2.99
2.96
9.0
8.5
8.0
7.5
7.0
6.5
6.0
5.5
5.0
4.5
4.0
3.5
3.0
2.5
2.0
1.5
1.0
0.5
0.0
ppm

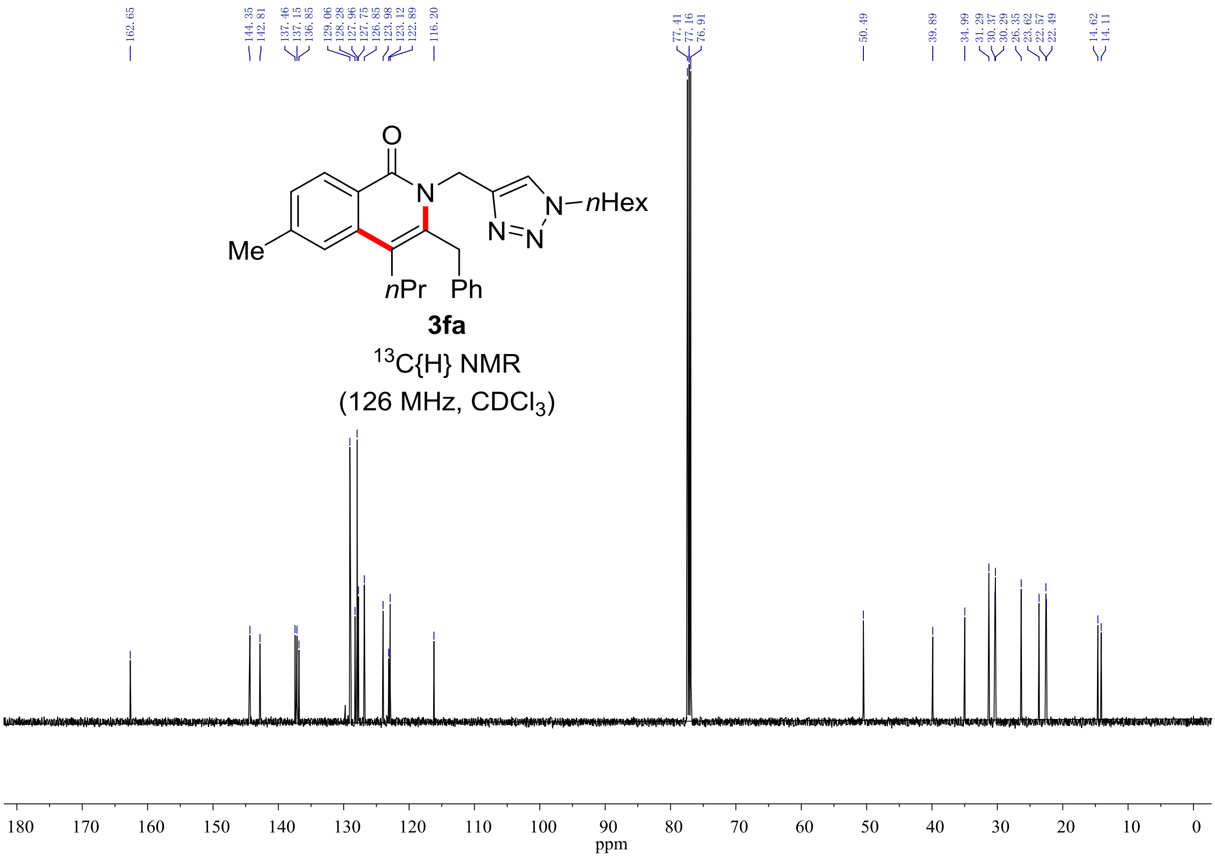

162.65
144.35
142.81
137.46
137.15
136.85
129.06
128.28
127.96
127.75
126.85
123.98
123.12
122.89
116.20
77.41
77.16
76.91
50.49
39.89
34.99
31.29
30.37
30.29
26.35
23.62
22.57
22.49
14.62
14.11
O
N
N
N=N
N−nHex
Me
nPr
Ph
3fa
13C{H} NMR
(126 MHz, CDCl3)
180
170
160
150
140
130
120
110
100
90
80
70
60
50
40
30
20
10
0
ppm

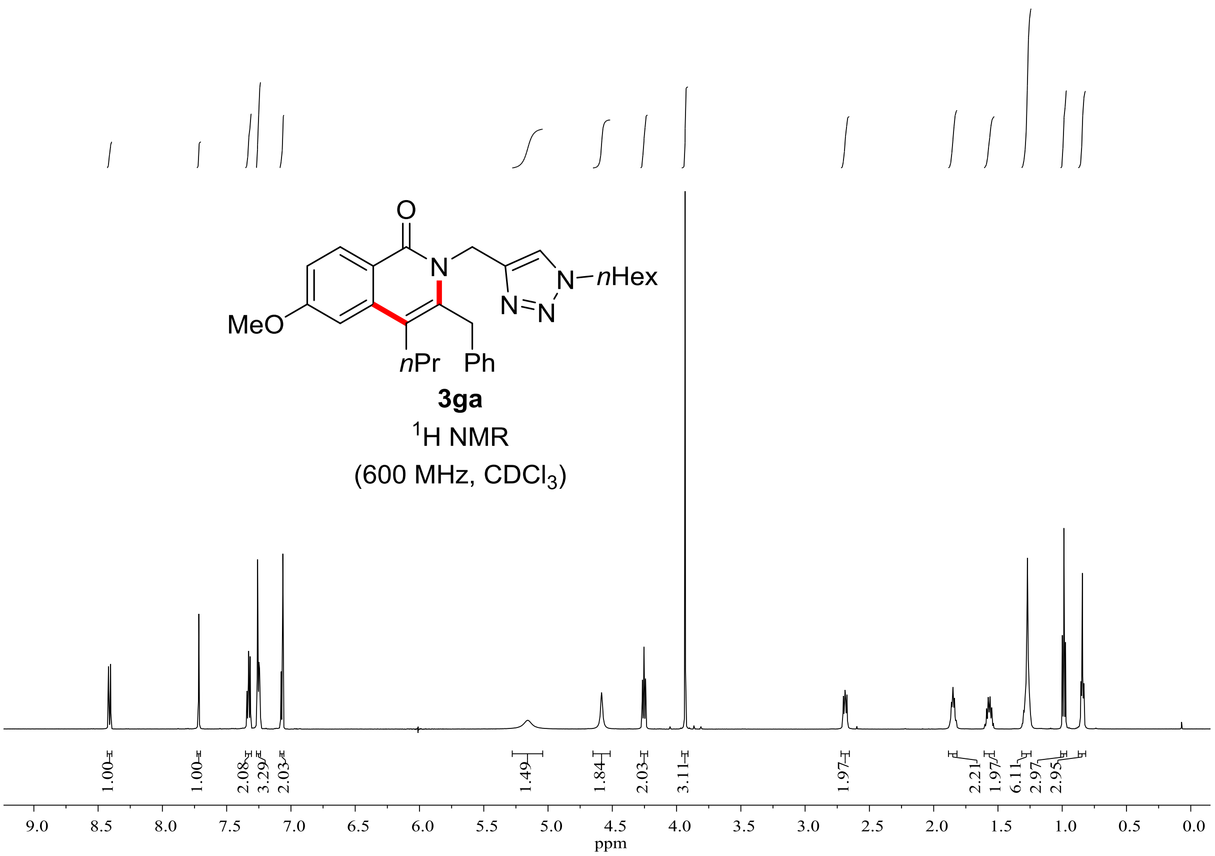

MeO
N
O
N
N=N
nHex
nPr
Ph
3ga
^{1}H NMR
(600 MHz, CDCl$_3$)
ppm

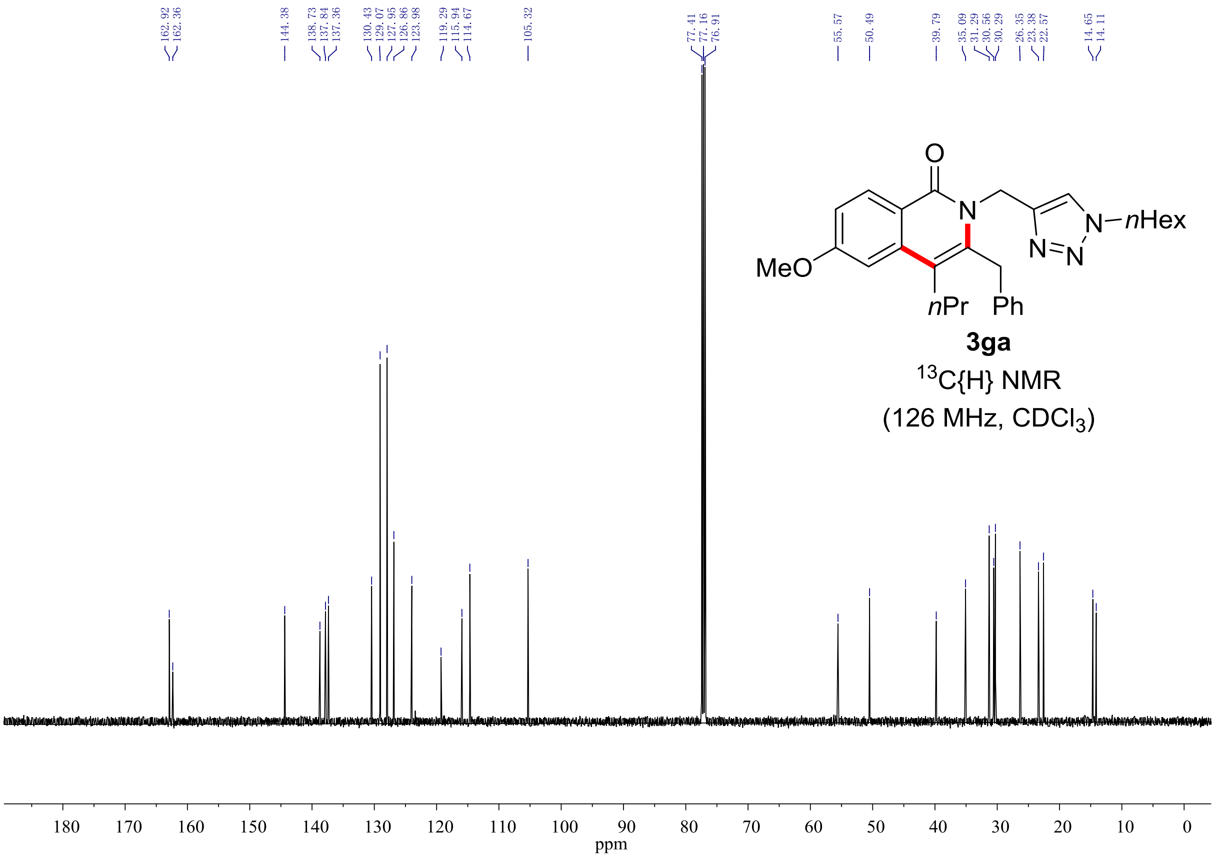

MeO
N
O
N
N=N
nHex
nPr
Ph
3ga
^{13}C{H} NMR
(126 MHz, CDCl$_3$)
ppm

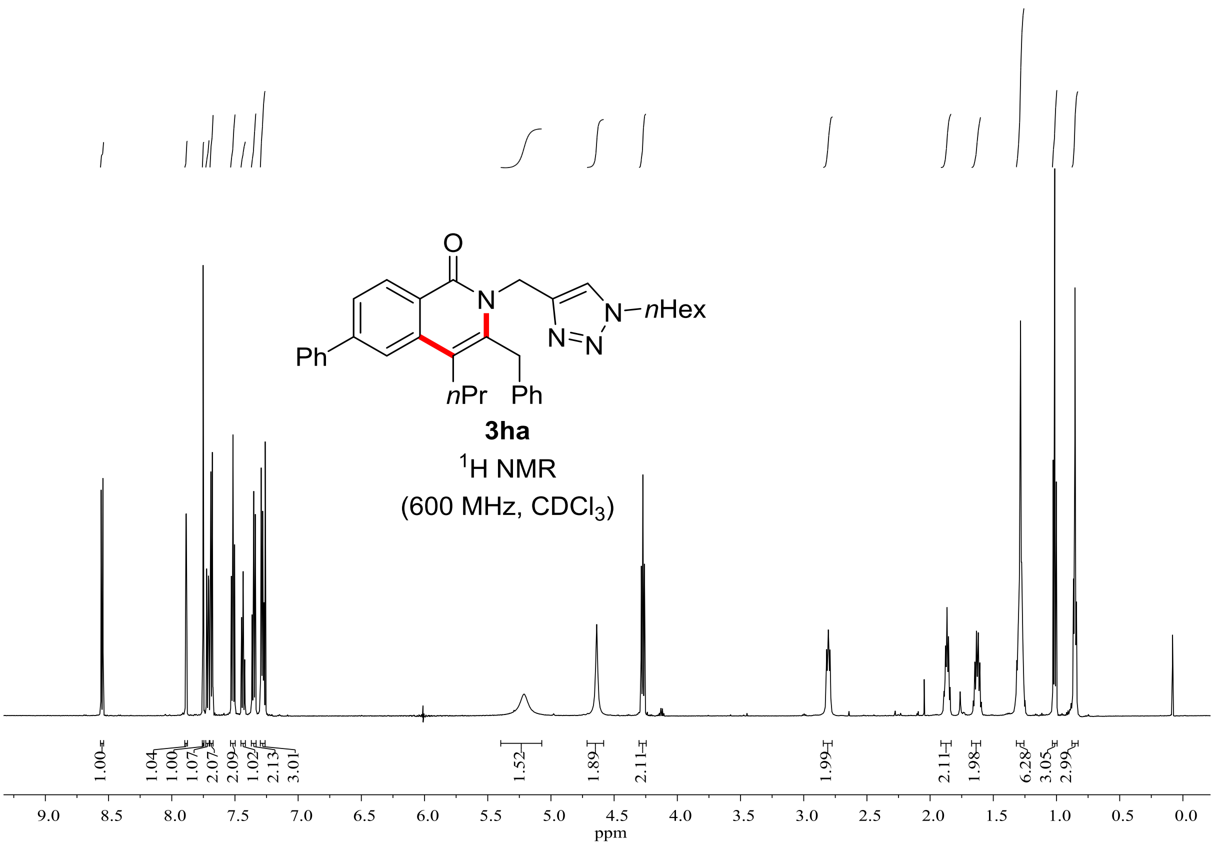

O
N
N−nHex
N=N
Ph
nPr
Ph
3ha
1H NMR
(600 MHz, CDCl3)
ppm

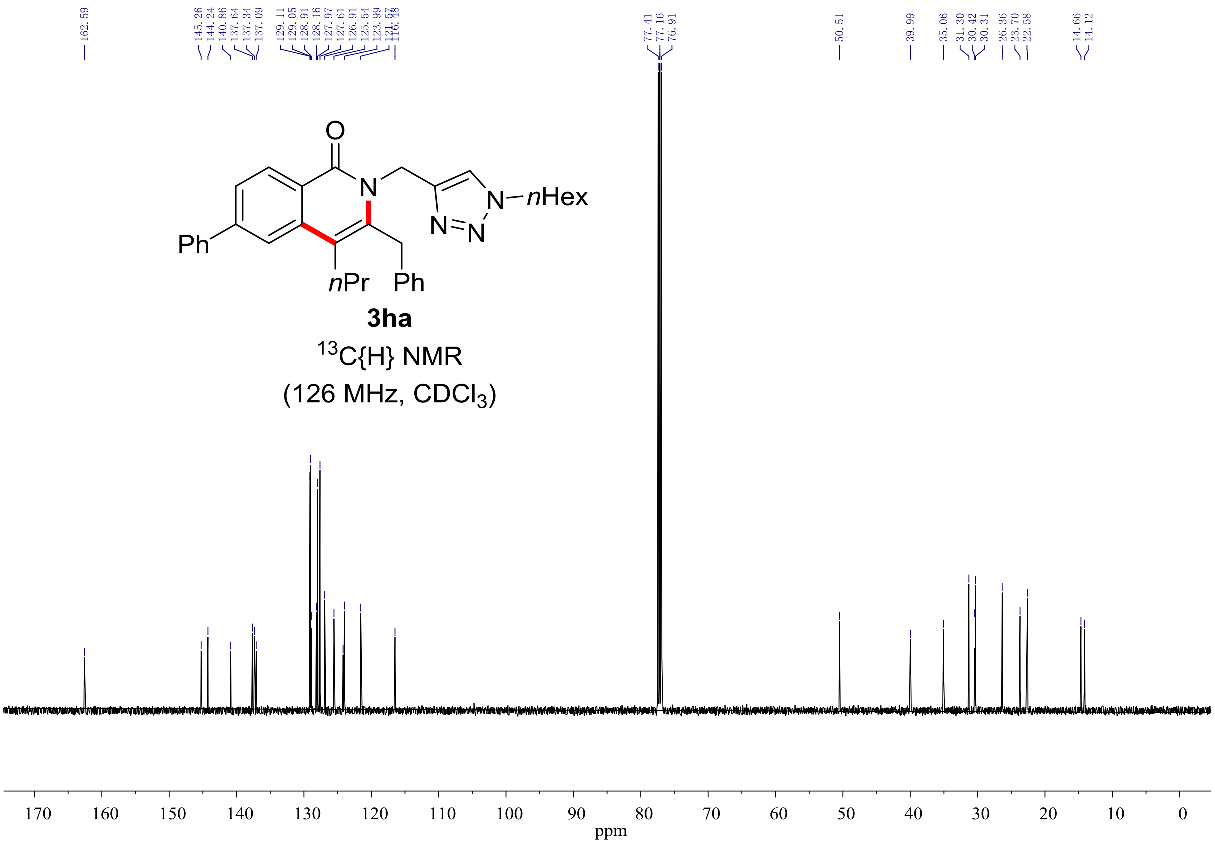

O
N
N−nHex
N=N
Ph
nPr
Ph
3ha
13C{H} NMR
(126 MHz, CDCl3)
ppm

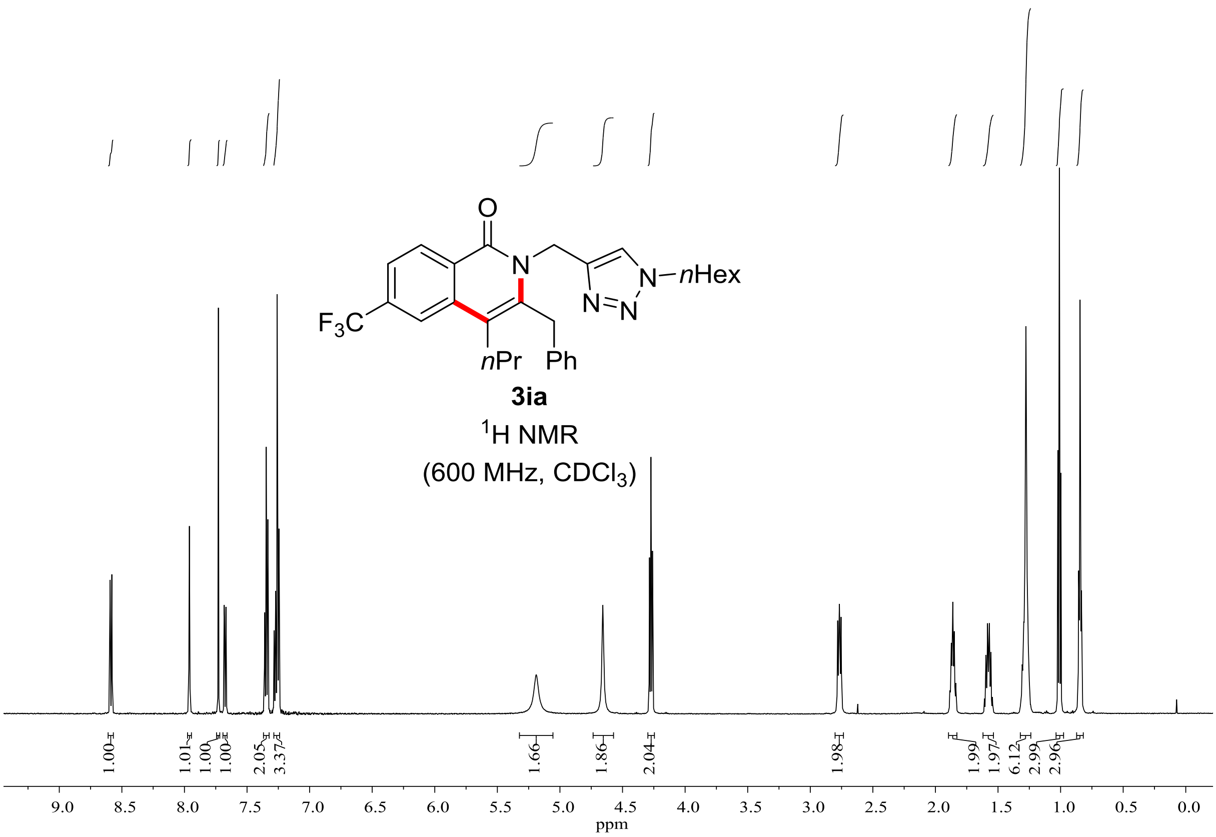
3ia
¹H NMR
(600 MHz, CDCl3)
ppm

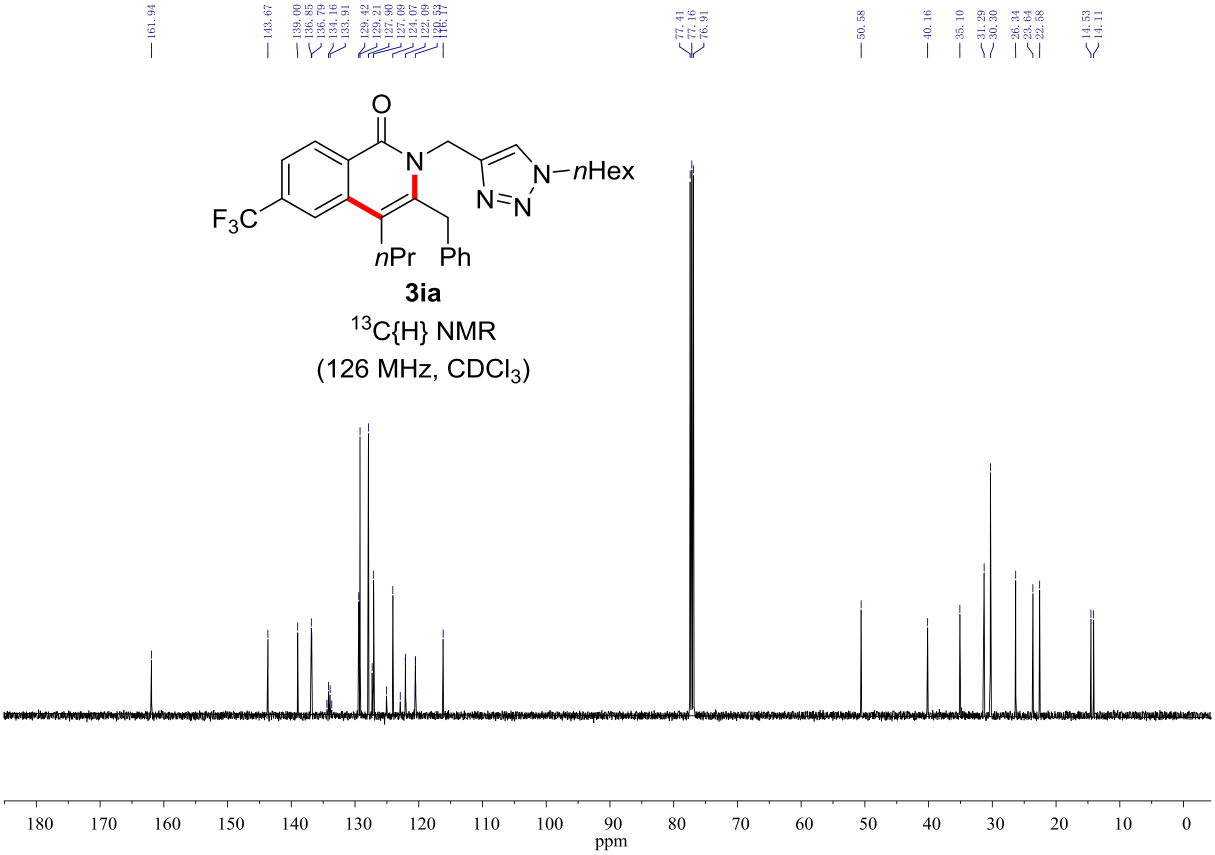
3ia
¹³C{H} NMR
(126 MHz, CDCl3)
ppm

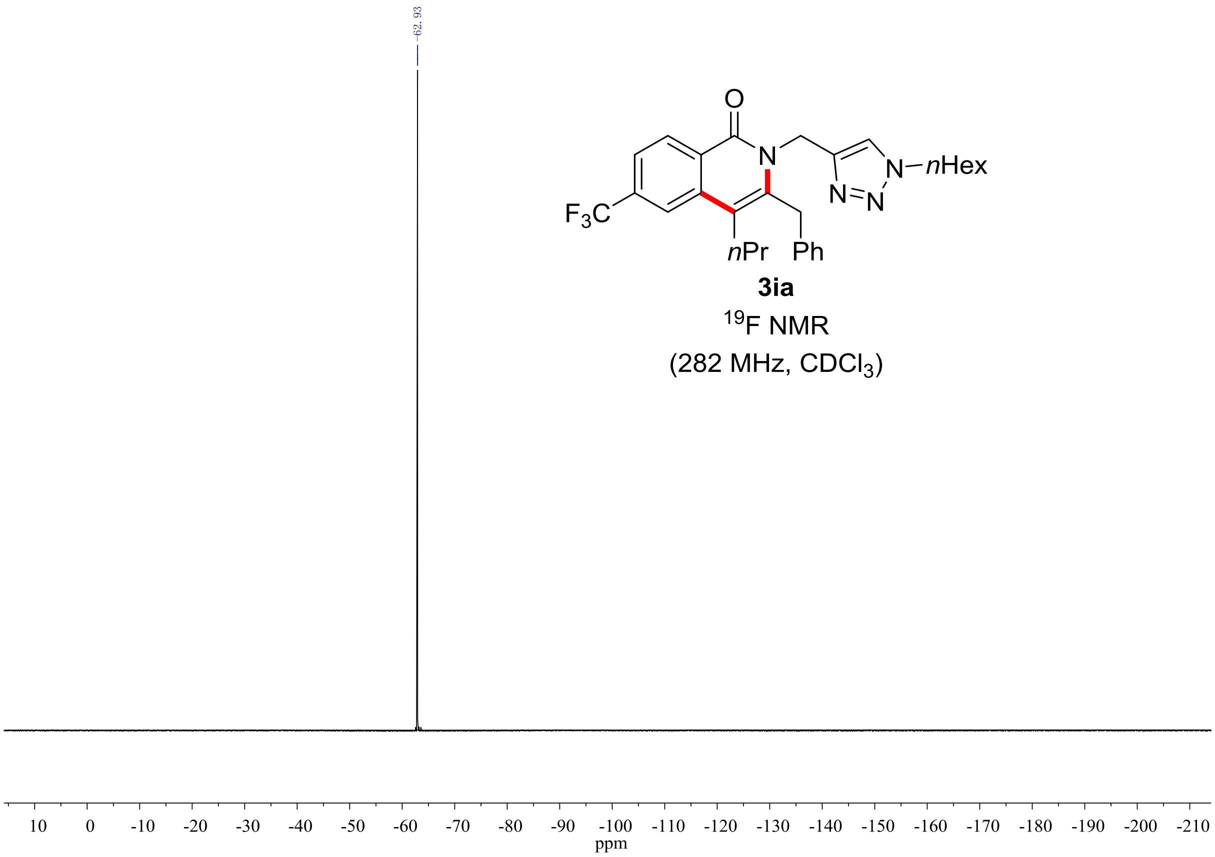
-62.93
O
N
N-nHex
N=N
F3C
nPr
Ph
3ia
19F NMR
(282 MHz, CDCl3)
10 0 -10 -20 -30 -40 -50 -60 -70 -80 -90 -100 -110 -120 -130 -140 -150 -160 -170 -180 -190 -200 -210
ppm

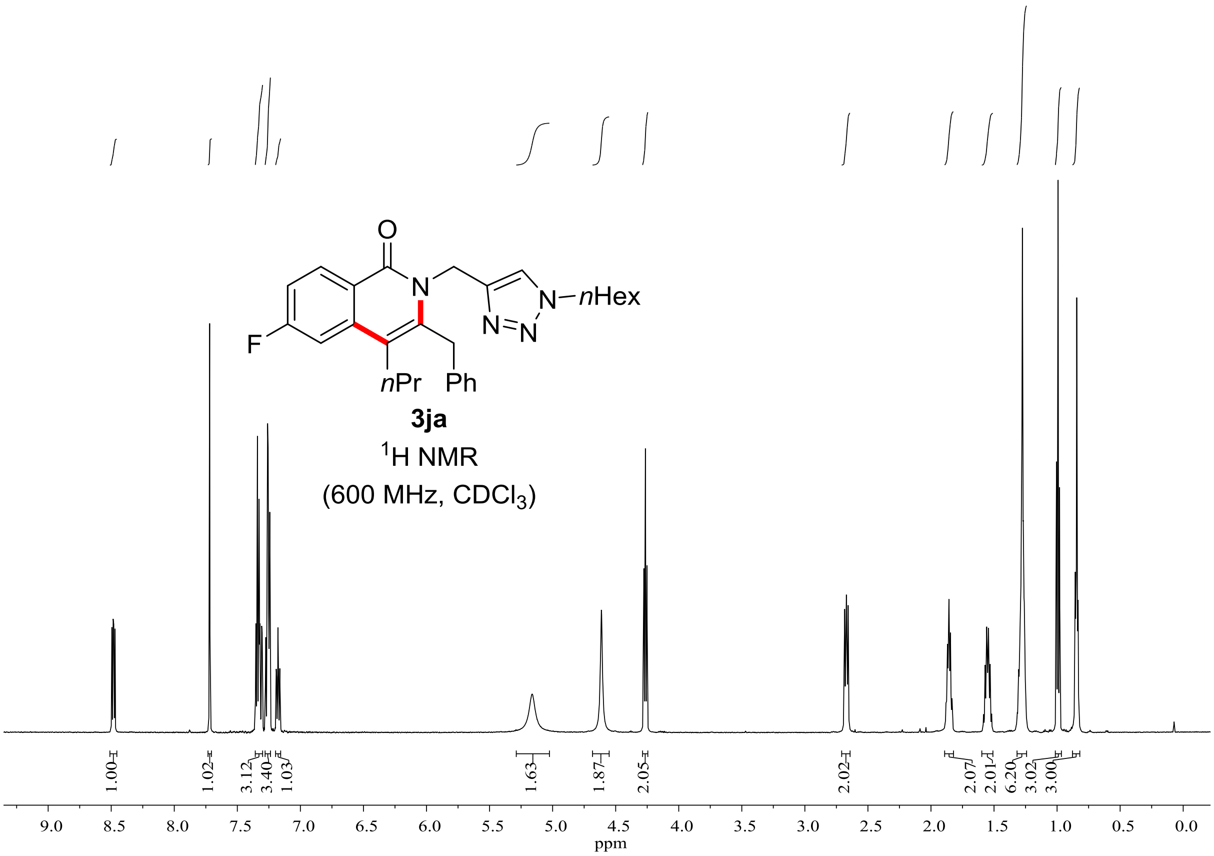

O
N
N
N
N
F
nPr
Ph
nHex
3ja
^{1}H NMR
(600 MHz, CDCl$_3$)
1.00
1.02
3.12
3.40
1.03
1.63
1.87
2.05
2.02
2.07
2.01
6.20
3.02
3.00
9.0
8.5
8.0
7.5
7.0
6.5
6.0
5.5
5.0
4.5
4.0
3.5
3.0
2.5
2.0
1.5
1.0
0.5
0.0
ppm

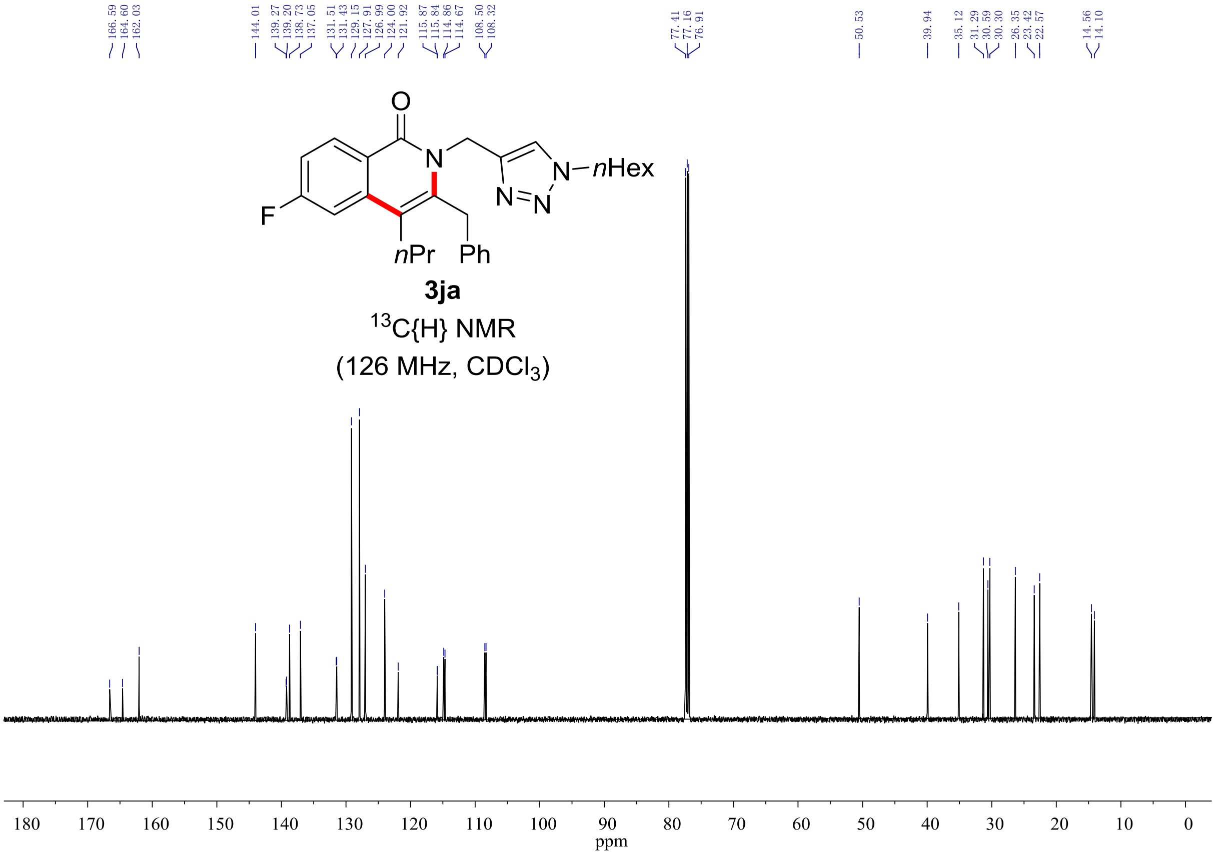

166.59
164.60
162.03
144.01
139.27
139.20
138.73
137.05
131.51
131.43
129.15
127.91
126.99
124.00
121.92
115.87
115.84
114.86
114.67
108.50
108.32
77.41
77.16
76.91
50.53
39.94
35.12
31.29
30.59
30.30
26.35
23.42
22.57
14.56
14.10
O
N
N
N
N
F
nPr
Ph
nHex
3ja
$^{13}C\{H\}$ NMR
(126 MHz, CDCl$_3$)
180
170
160
150
140
130
120
110
100
90
80
70
60
50
40
30
20
10
0
ppm

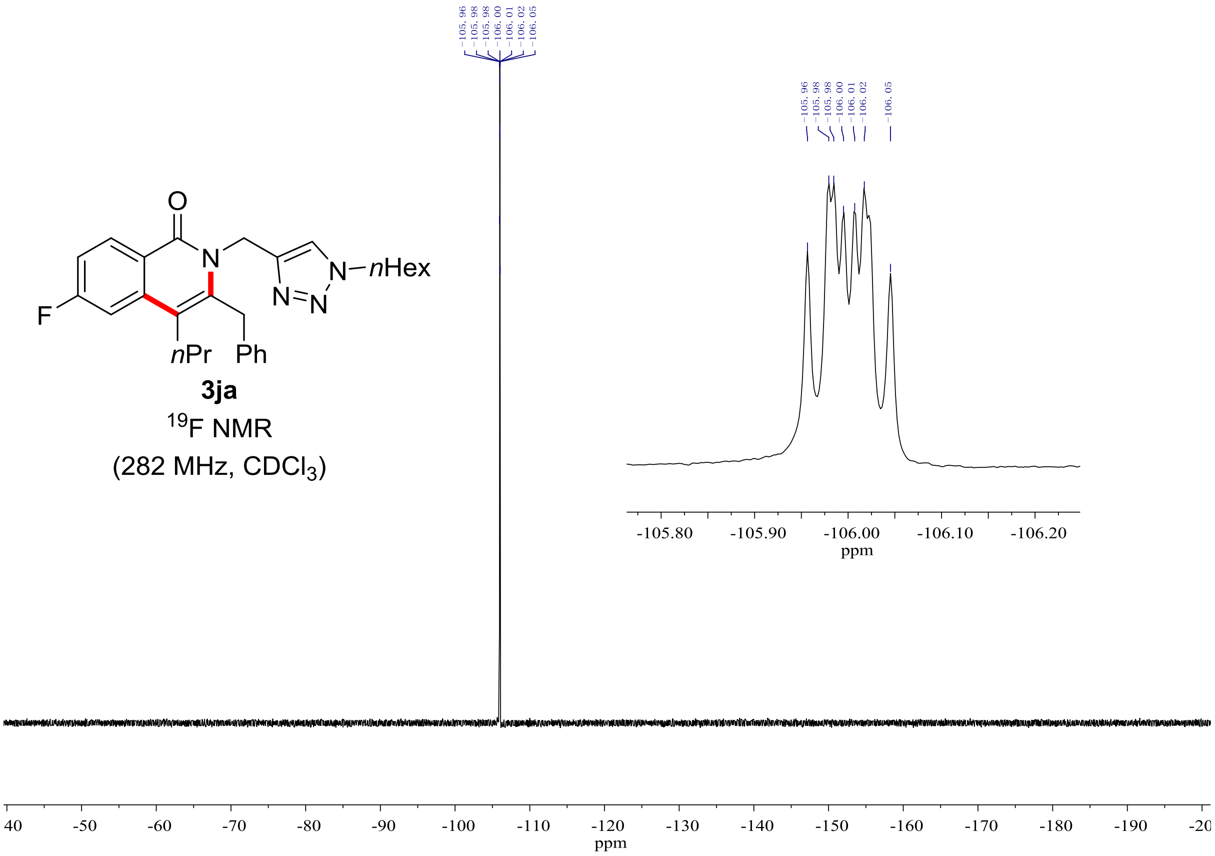
3ja
^{19}F NMR
(282 MHz, $CDCl_3$)
ppm

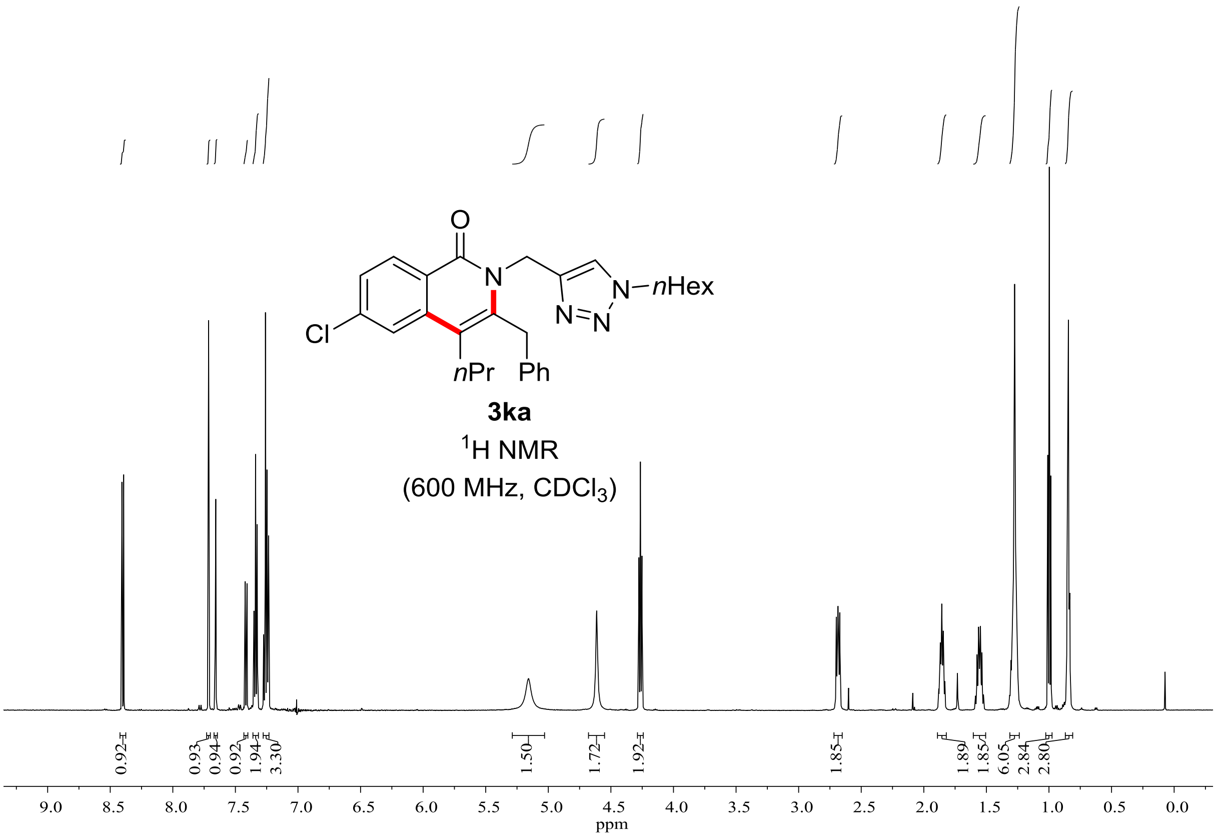
O
N
N
N=N
nHex
Cl
nPr
Ph
3ka
^{1}H NMR
(600 MHz, $CDCl_3$)
0.92
0.93
0.94
0.92
1.94
3.30
1.50
1.72
1.92
1.85
1.89
1.85
6.05
2.84
2.80
9.0 8.5 8.0 7.5 7.0 6.5 6.0 5.5 5.0 4.5 4.0 3.5 3.0 2.5 2.0 1.5 1.0 0.5 0.0
ppm

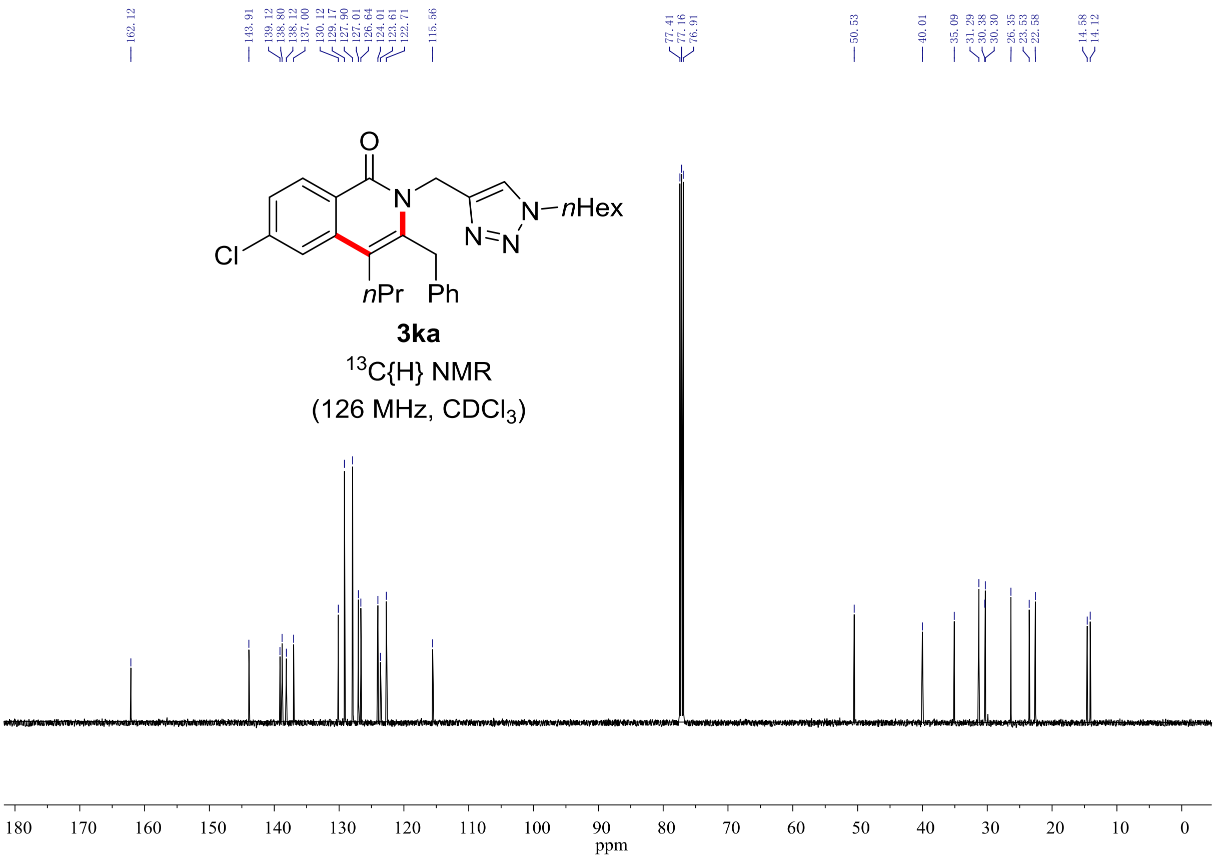
162.12
143.91
139.12
138.80
138.12
137.00
130.12
129.17
127.90
127.01
126.64
124.01
123.61
122.71
115.56
77.41
77.16
76.91
50.53
40.01
35.09
31.29
30.38
30.30
26.35
23.53
22.58
14.58
14.12
O
N
N
N=N
nHex
Cl
nPr
Ph
3ka
^{13}C{H} NMR
(126 MHz, $CDCl_3$)
180 170 160 150 140 130 120 110 100 90 80 70 60 50 40 30 20 10 0
ppm

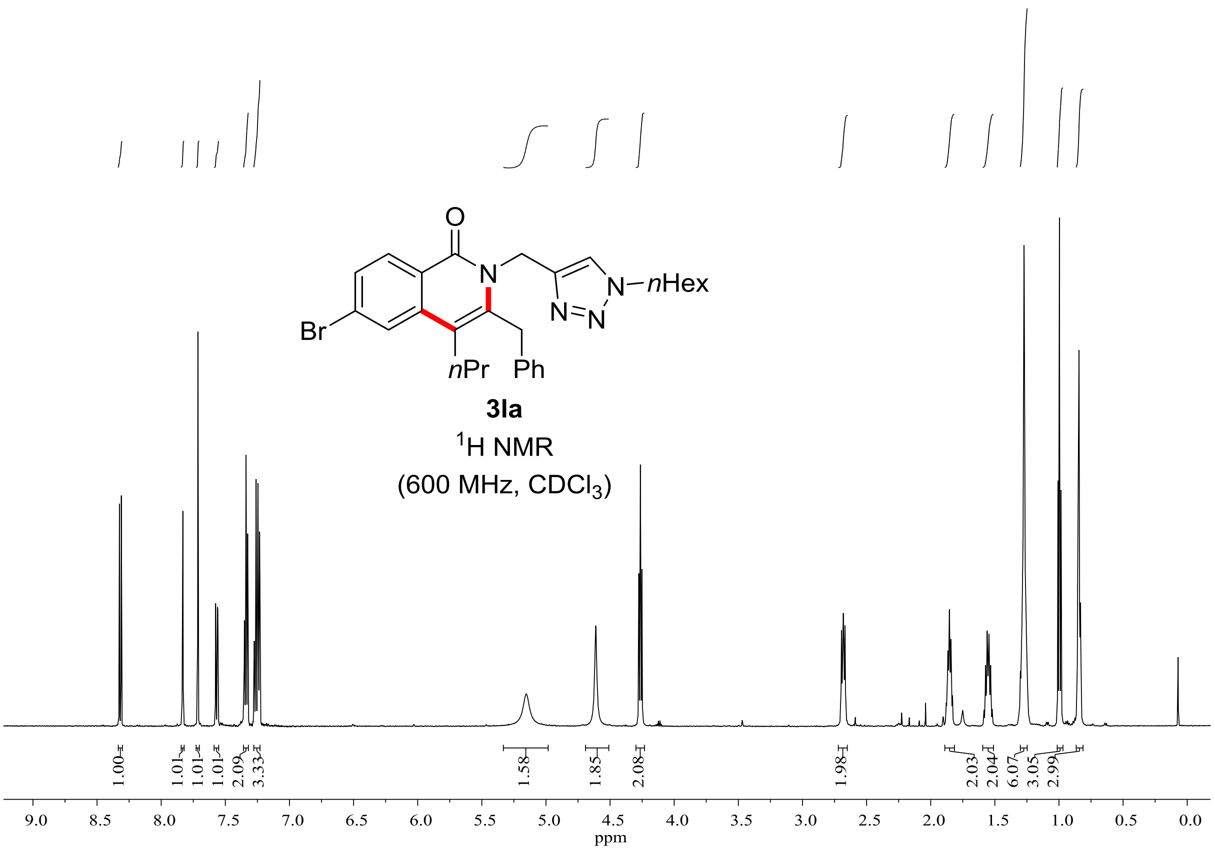

3la
^{1}H NMR
(600 MHz, CDCl$_3$)

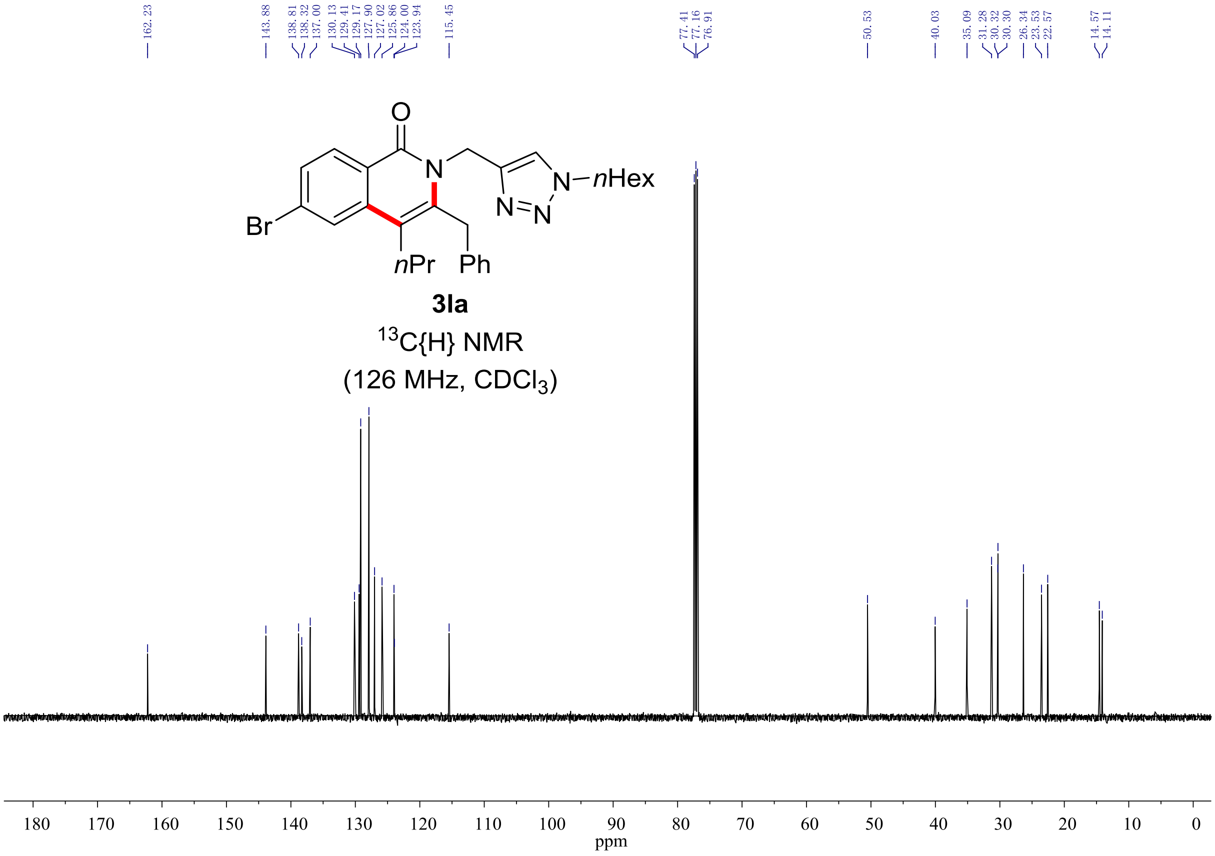

3la
^{13}C{H} NMR
(126 MHz, CDCl$_3$)

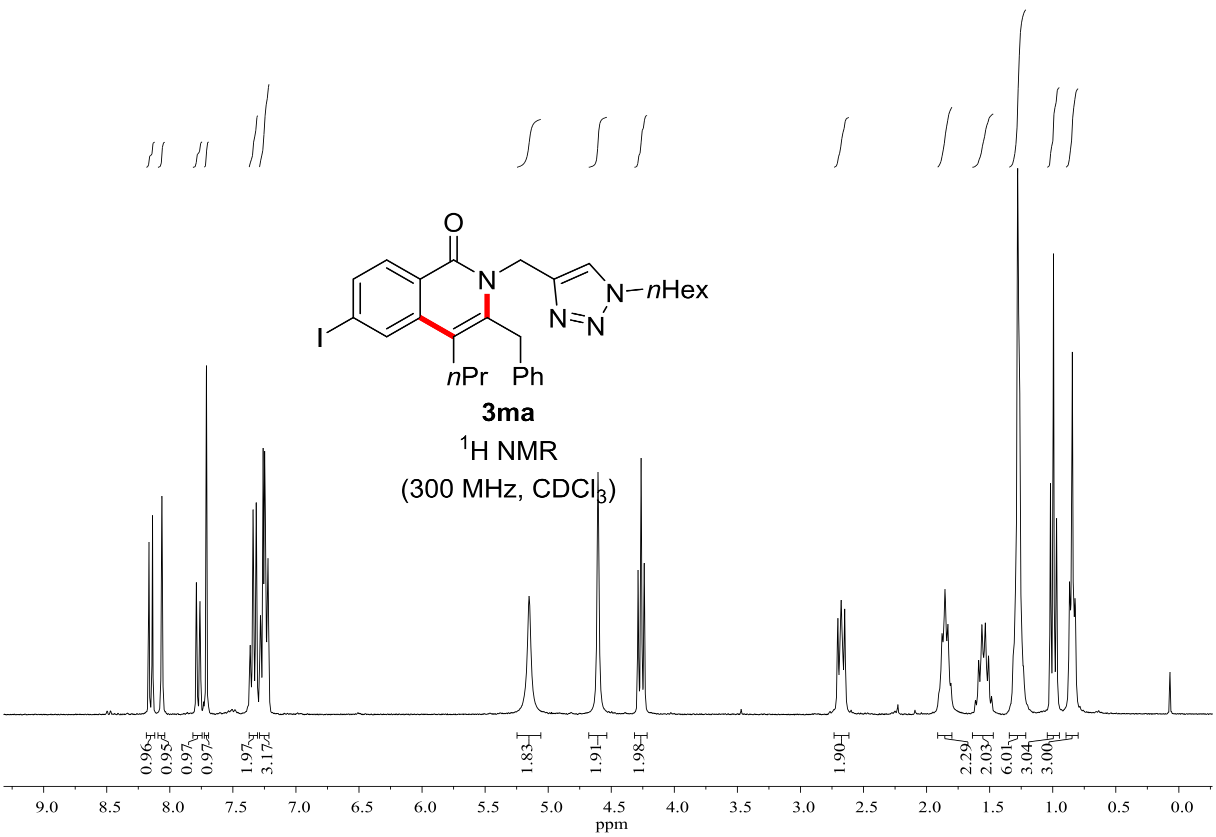

O
N
N−nHex
N=N
I
nPr
Ph
3ma
1H NMR
(300 MHz, CDCl3)
0.96
0.95
0.97
0.97
1.97
3.17
1.83
1.91
1.98
1.90
2.29
2.03
6.01
3.04
3.00
9.0
8.5
8.0
7.5
7.0
6.5
6.0
5.5
5.0
4.5
4.0
3.5
3.0
2.5
2.0
1.5
1.0
0.5
0.0
ppm

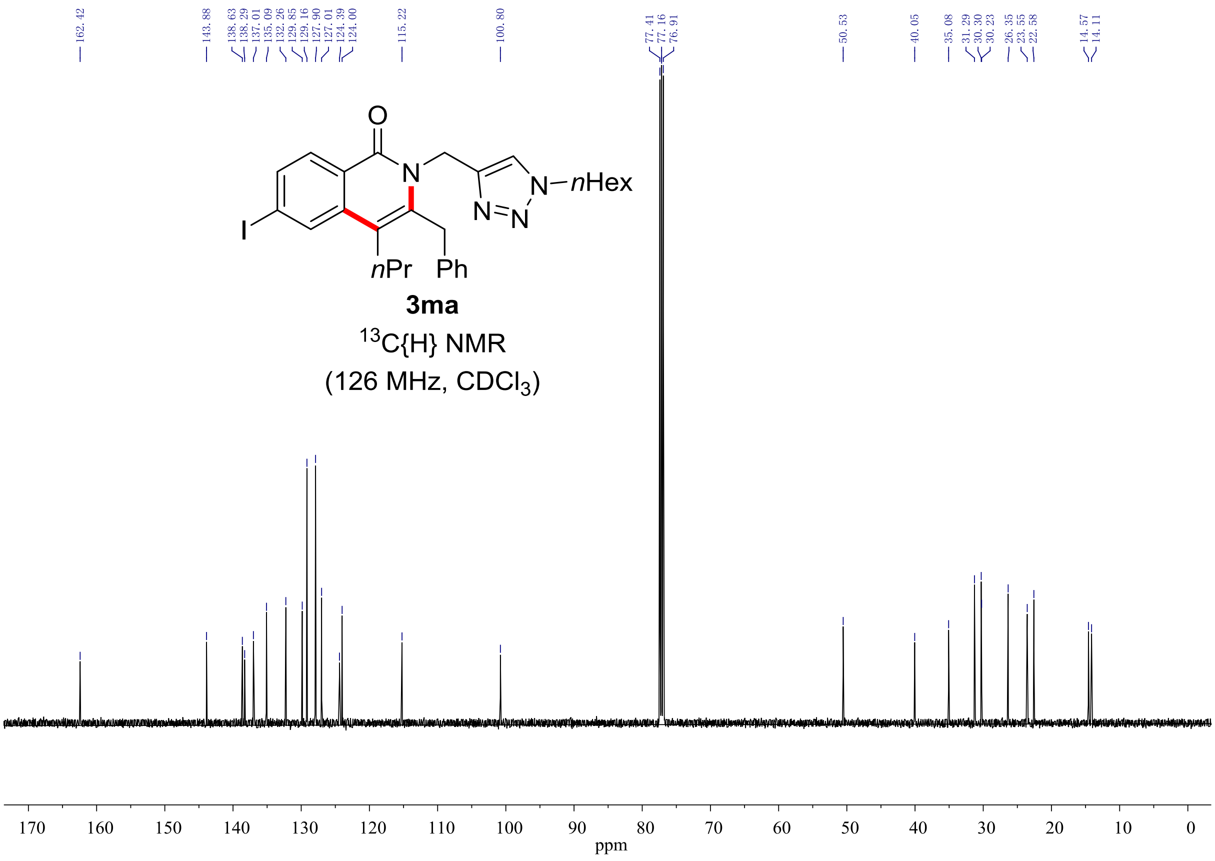

162.42
143.88
138.63
138.29
137.01
135.09
132.26
129.85
129.16
127.90
127.01
124.39
124.00
115.22
100.80
77.41
77.16
76.91
50.53
40.05
35.08
31.29
30.30
30.23
26.35
23.55
22.58
14.57
14.11
O
N
N−nHex
N=N
I
nPr
Ph
3ma
13C{H} NMR
(126 MHz, CDCl3)
170
160
150
140
130
120
110
100
90
80
70
60
50
40
30
20
10
0
ppm

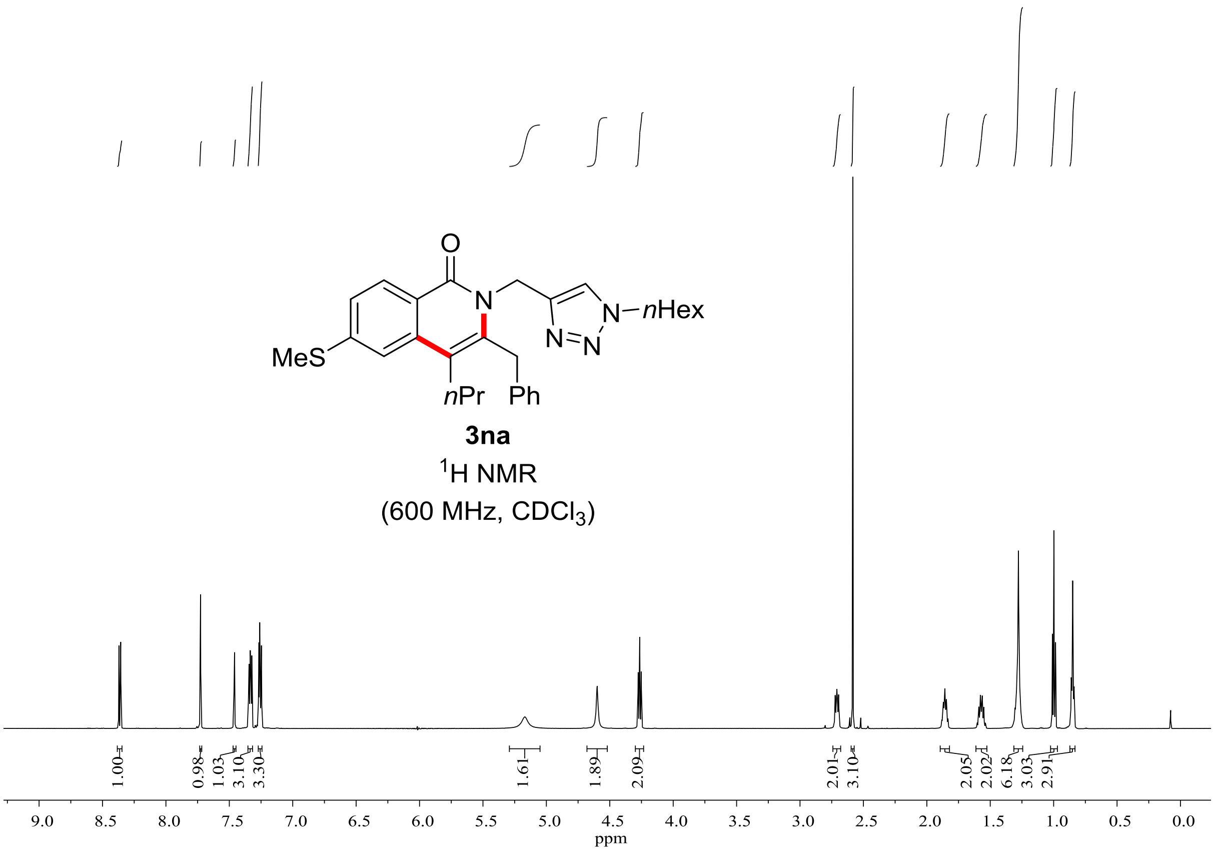
O
N
N
nHex
N
N
MeS
nPr
Ph
3na
1H NMR
(600 MHz, CDCl3)
1.00
0.98
1.03
3.10
3.30
1.61
1.89
2.09
2.01
3.10
2.05
2.02
6.18
3.03
2.91
9.0 8.5 8.0 7.5 7.0 6.5 6.0 5.5 5.0 4.5 4.0 3.5 3.0 2.5 2.0 1.5 1.0 0.5 0.0
ppm

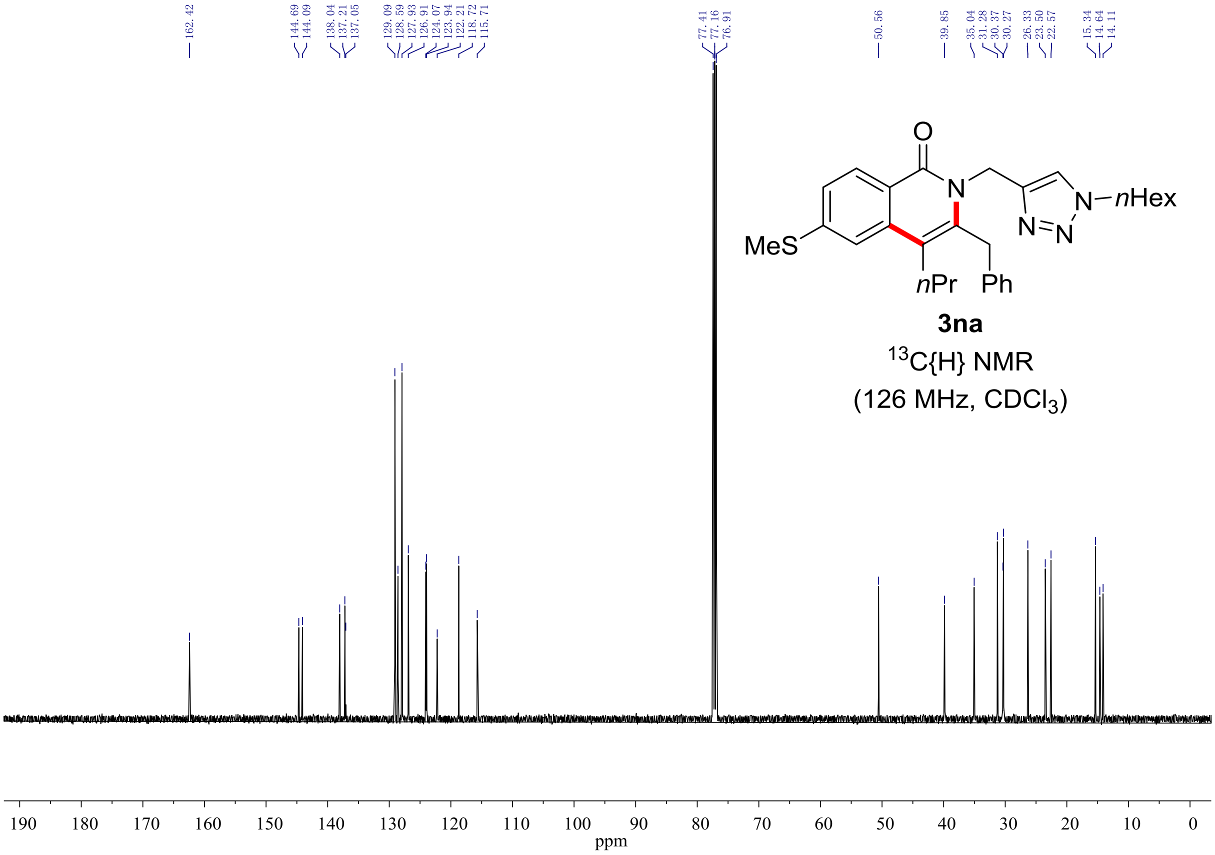
162.42
144.69
144.09
138.04
137.21
137.05
129.09
128.59
127.93
126.91
124.07
123.94
122.21
118.72
115.71
77.41
77.16
76.91
50.56
39.85
35.04
31.28
30.37
30.27
26.33
23.50
22.57
15.34
14.64
14.11
O
N
N
nHex
N
N
MeS
nPr
Ph
3na
13C{H} NMR
(126 MHz, CDCl3)
190 180 170 160 150 140 130 120 110 100 90 80 70 60 50 40 30 20 10 0
ppm

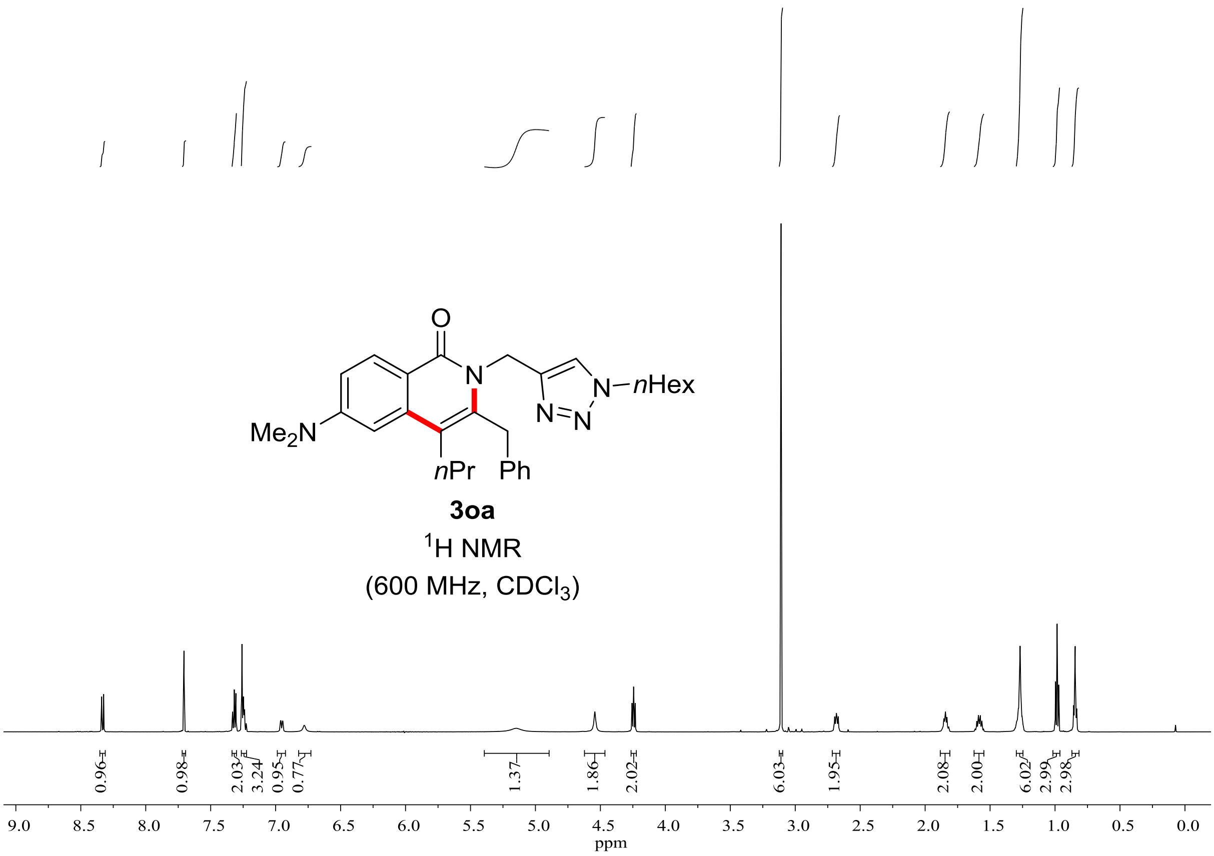
O
N
N
N=N
N−nHex
Me2N
nPr
Ph
3oa
1H NMR
(600 MHz, CDCl3)
0.96 0.98 2.03 3.24 0.95 0.77 1.37 1.86 2.02 6.03 1.95 2.08 2.00 6.02 2.99 2.98
9.0 8.5 8.0 7.5 7.0 6.5 6.0 5.5 5.0 4.5 4.0 3.5 3.0 2.5 2.0 1.5 1.0 0.5 0.0
ppm

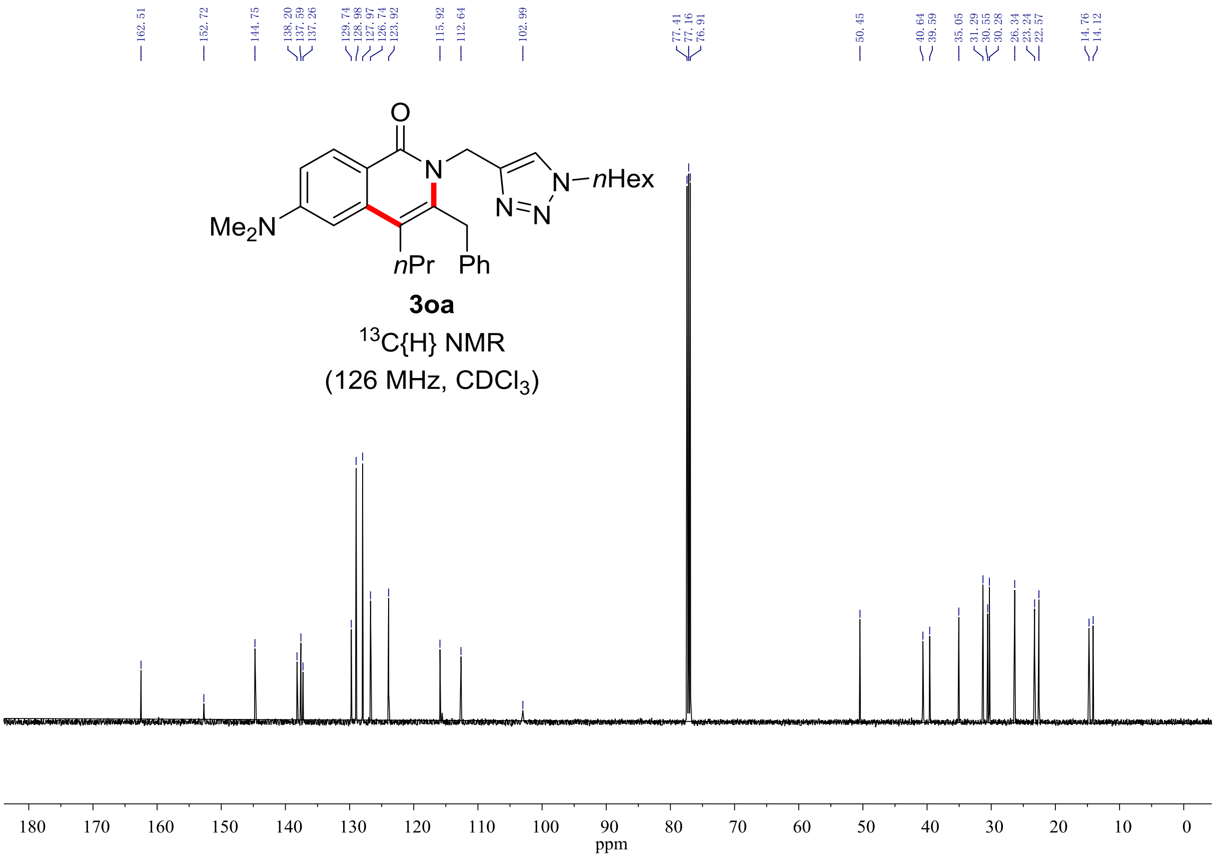
162.51 152.72 144.75 138.20 137.59 137.26 129.74 128.98 127.97 126.74 123.92 115.92 112.64 102.99 77.41 77.16 76.91 50.45 40.64 39.59 35.05 31.29 30.55 30.28 26.34 23.24 22.57 14.76 14.12
O
N
N
N=N
N−nHex
Me2N
nPr
Ph
3oa
13C{H} NMR
(126 MHz, CDCl3)
180 170 160 150 140 130 120 110 100 90 80 70 60 50 40 30 20 10 0
ppm

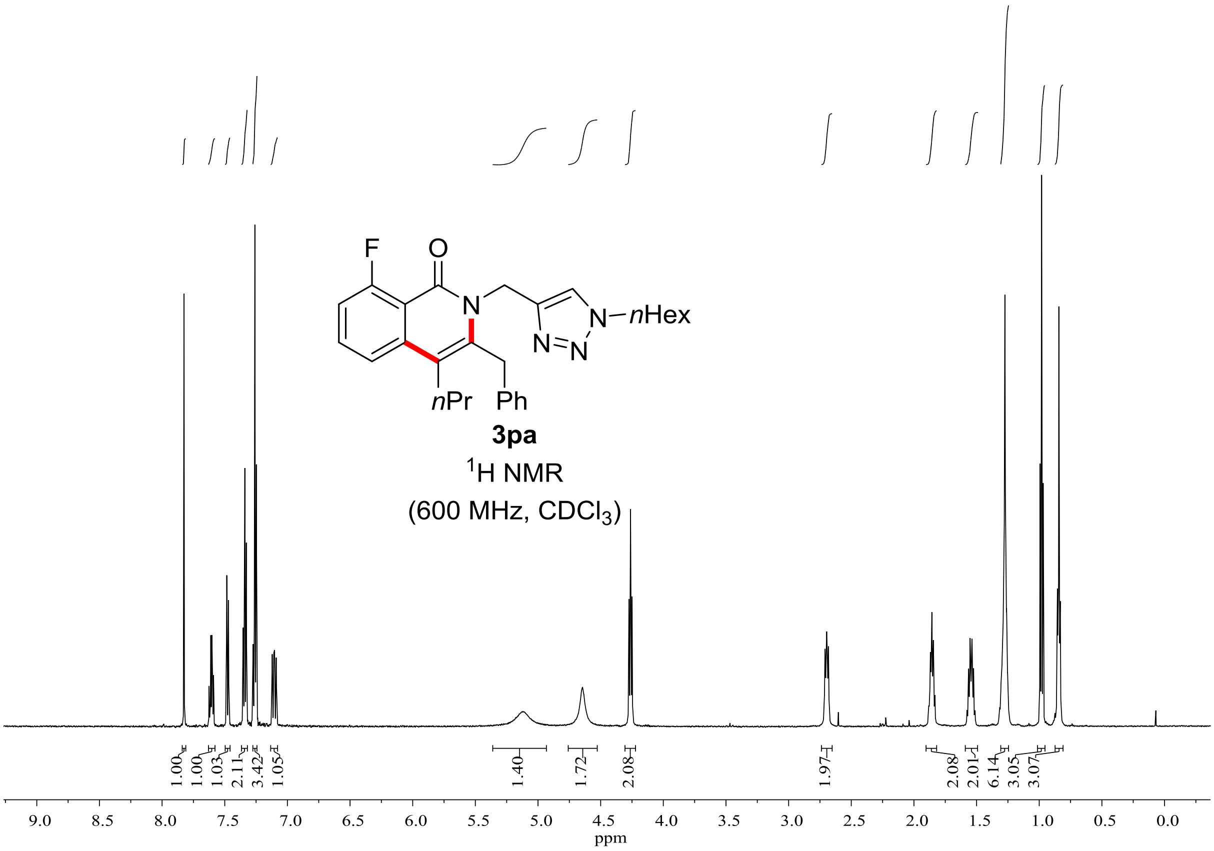
F
O
N
N−nHex
N=N
nPr
Ph
3pa
1H NMR
(600 MHz, CDCl3)
1.00
1.00
1.03
2.11
3.42
1.05
1.40
1.72
2.08
1.97
2.08
2.01
6.14
3.05
3.07
9.0
8.5
8.0
7.5
7.0
6.5
6.0
5.5
5.0
4.5
4.0
3.5
3.0
2.5
2.0
1.5
1.0
0.5
0.0
ppm

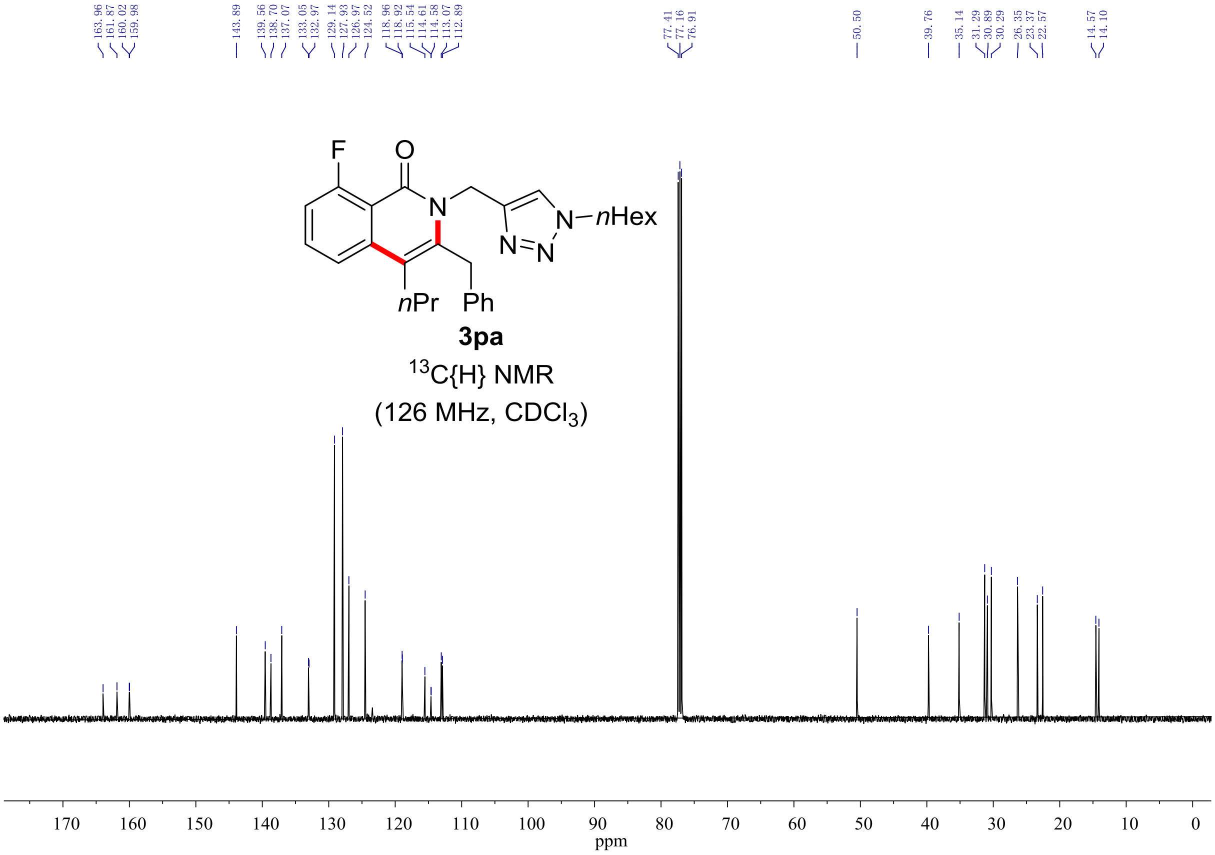
163.96
161.87
160.02
159.98
143.89
139.56
138.70
137.07
133.05
132.97
129.14
127.93
126.97
124.52
118.96
118.92
115.54
114.61
114.58
113.07
112.89
77.41
77.16
76.91
50.50
39.76
35.14
31.29
30.89
30.29
26.35
23.37
22.57
14.57
14.10
F
O
N
N−nHex
N=N
nPr
Ph
3pa
13C{H} NMR
(126 MHz, CDCl3)
170
160
150
140
130
120
110
100
90
80
70
60
50
40
30
20
10
0
ppm

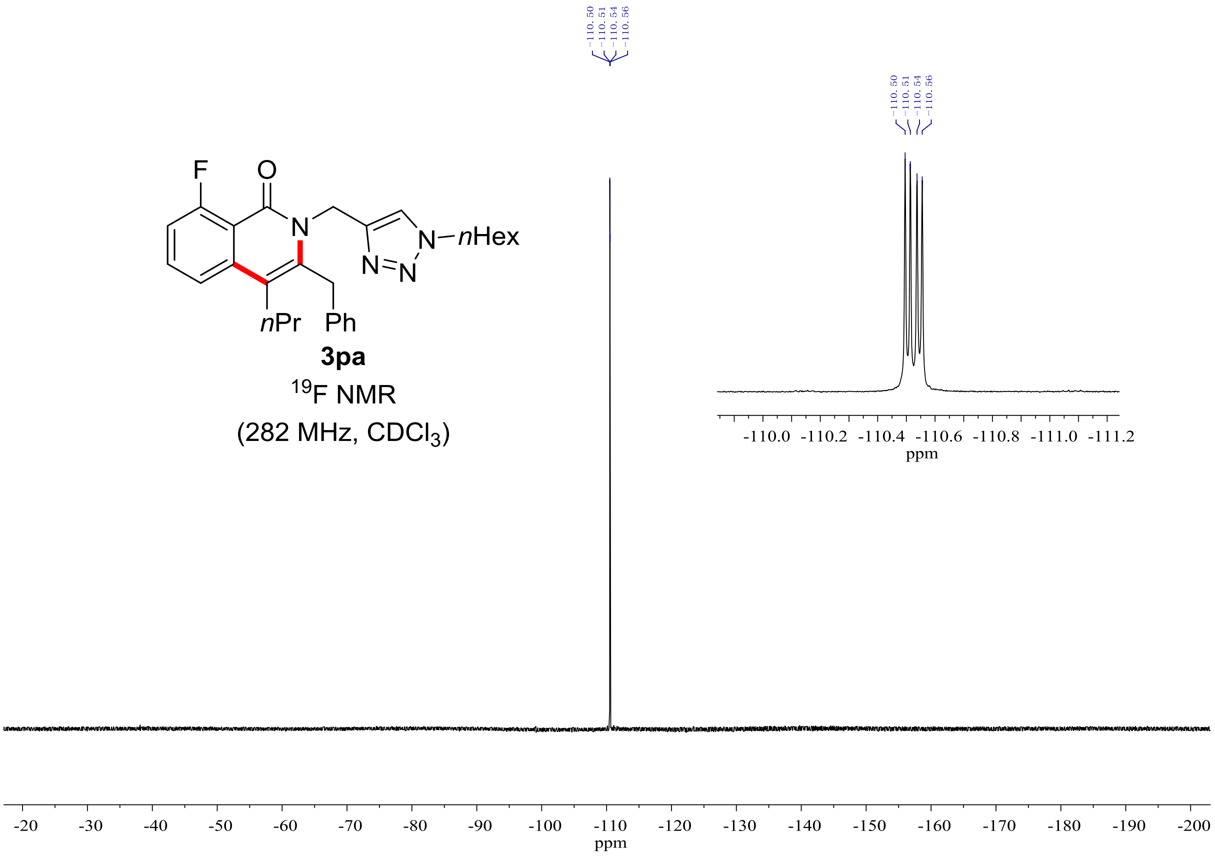

3pa

^{19}F NMR

(282 MHz, $CDCl_3$)

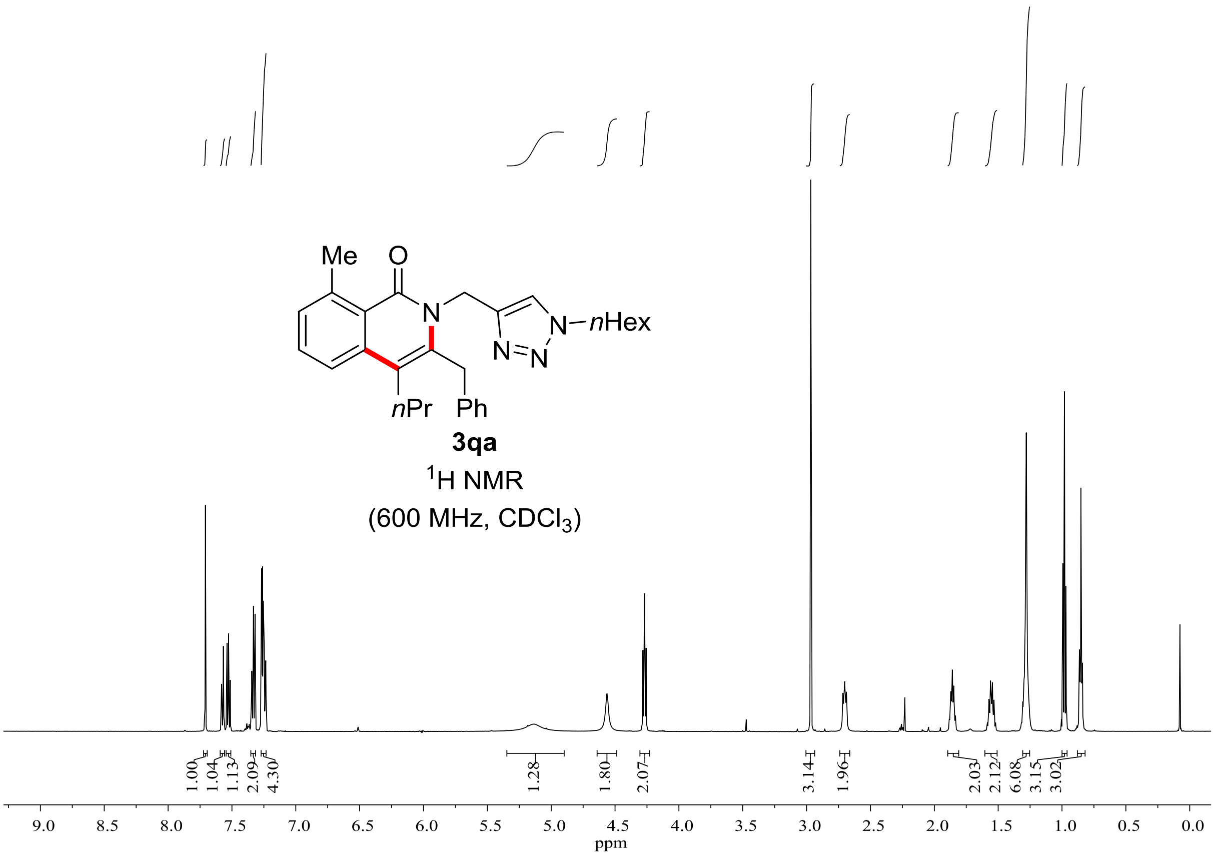

Me
O
N
N
nHex
N
N
nPr
Ph
3qa
^{1}H NMR
(600 MHz, CDCl$_3$)
ppm

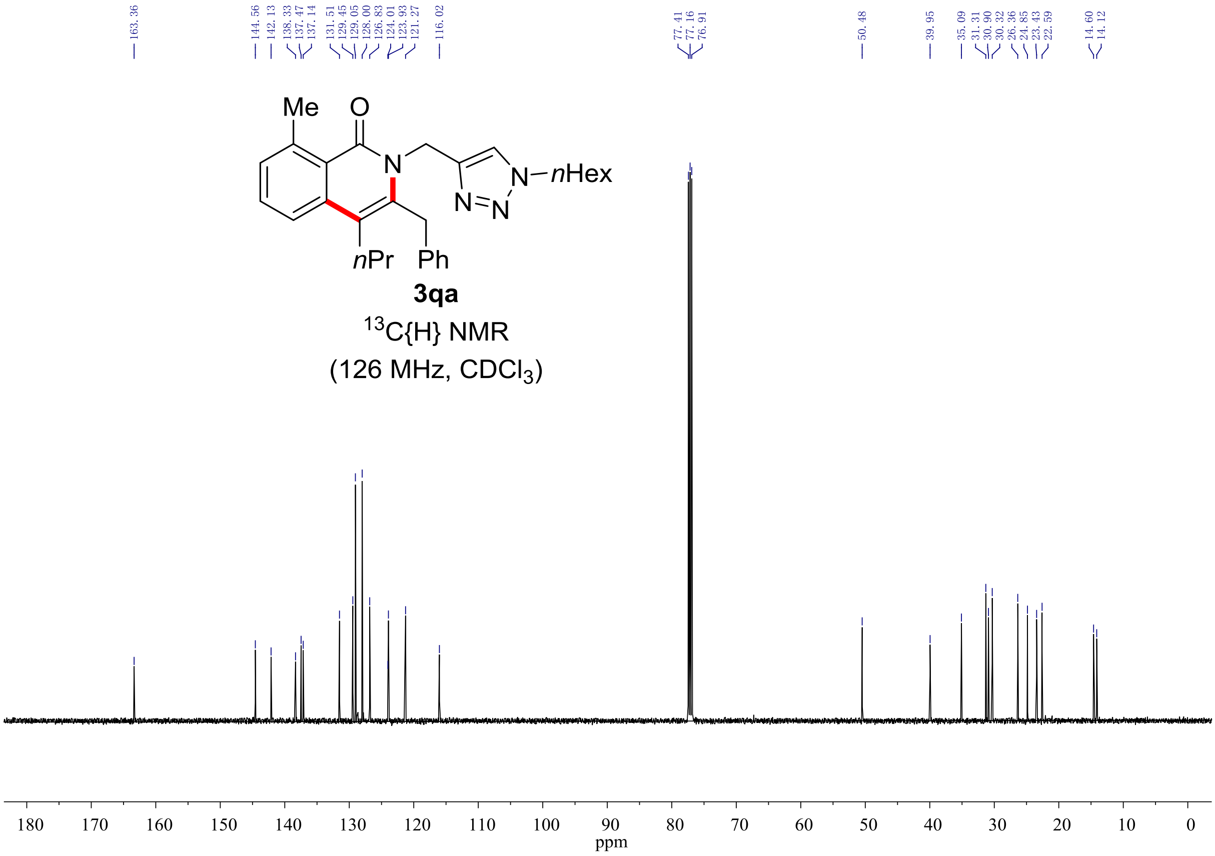

Me
O
N
N
nHex
N
N
nPr
Ph
3qa
^{13}C{H} NMR
(126 MHz, CDCl$_3$)
ppm

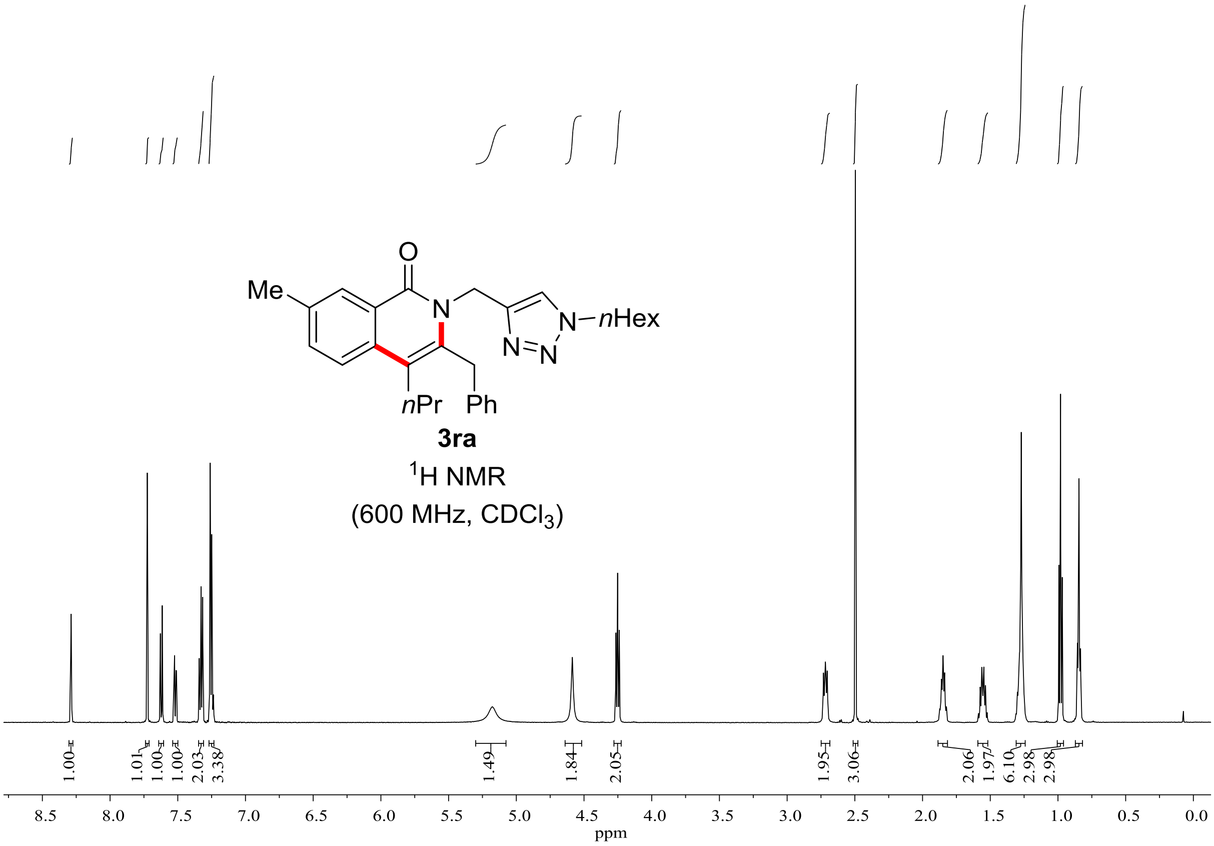

Me
O
N
N
N=N
nHex
nPr
Ph
3ra
^{1}H NMR
(600 MHz, CDCl$_3$)
1.00
1.01
1.00
1.00
2.03
3.38
1.49
1.84
2.05
1.95
3.06
2.06
1.97
6.10
2.98
2.98
ppm

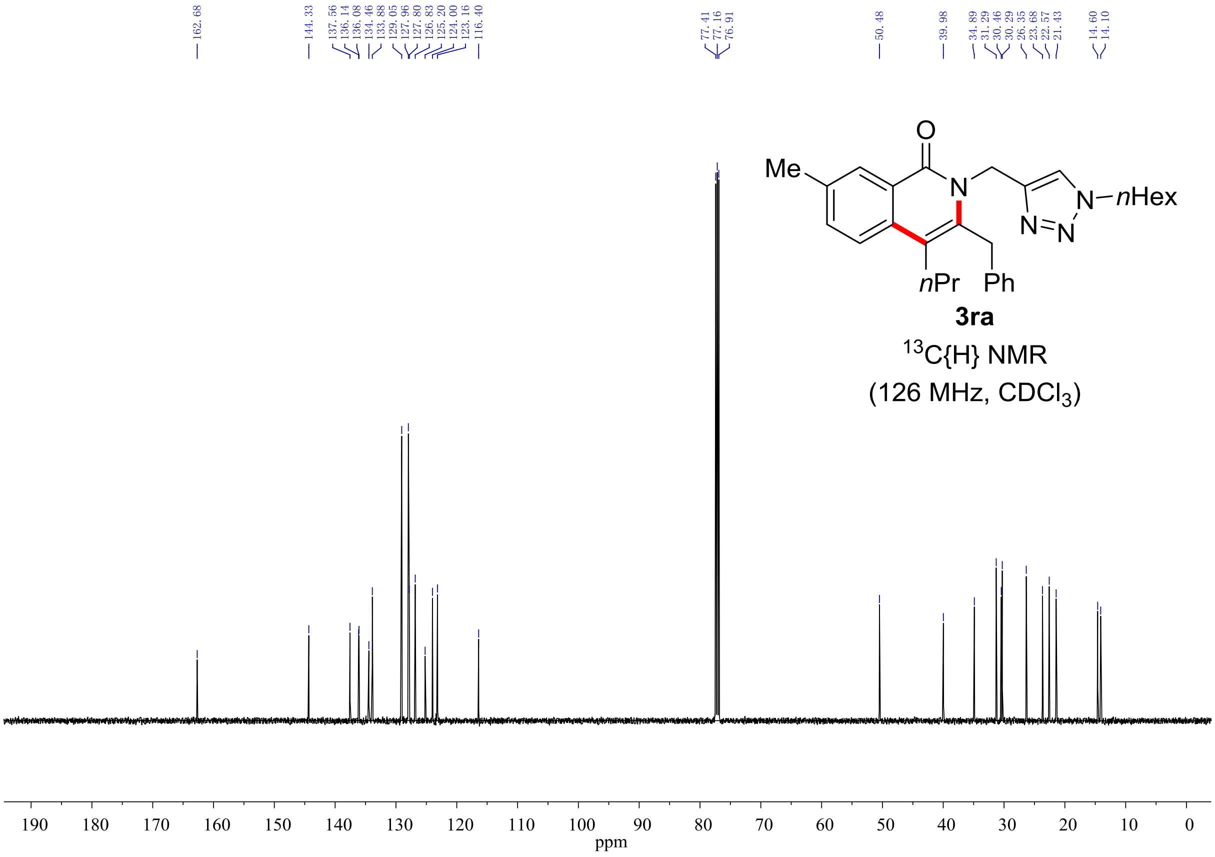

162.68
144.33
137.56
136.14
136.08
134.46
133.88
129.05
127.96
127.80
126.83
125.20
124.00
123.16
116.40
77.41
77.16
76.91
50.48
39.98
34.89
31.29
30.46
30.29
26.35
23.68
22.57
21.43
14.60
14.10
Me
O
N
N
N=N
nHex
nPr
Ph
3ra
^{13}C{H} NMR
(126 MHz, CDCl$_3$)
ppm

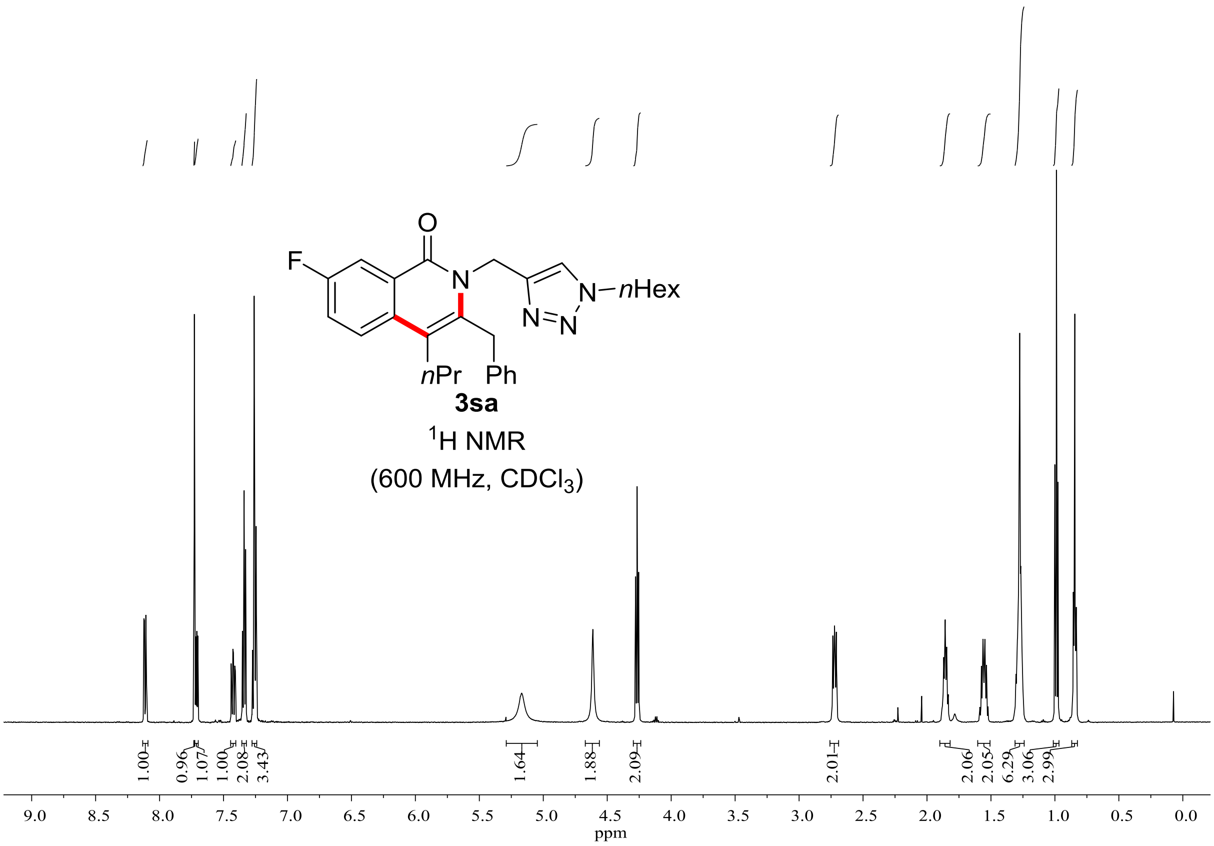
3sa
^{1}H NMR
(600 MHz, CDCl3)
ppm

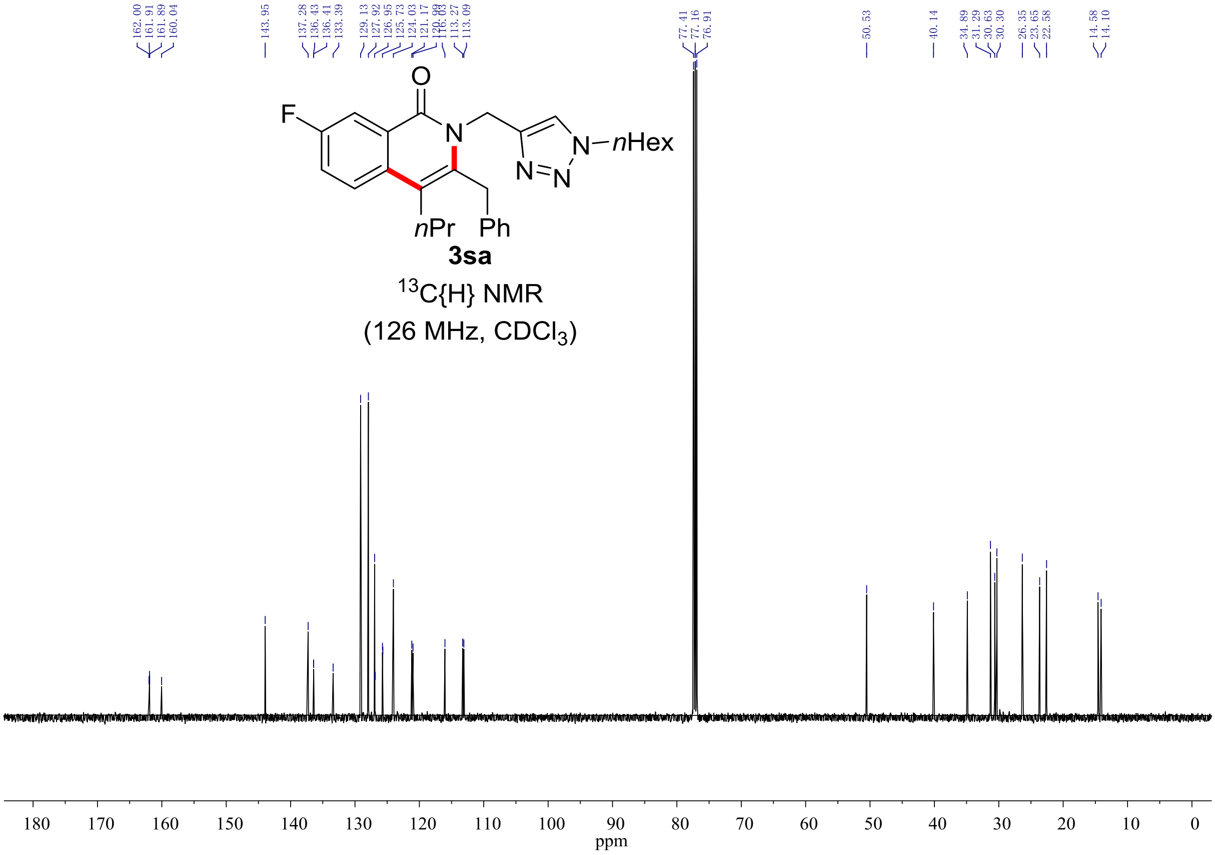
3sa
^{13}C{H} NMR
(126 MHz, CDCl3)
ppm

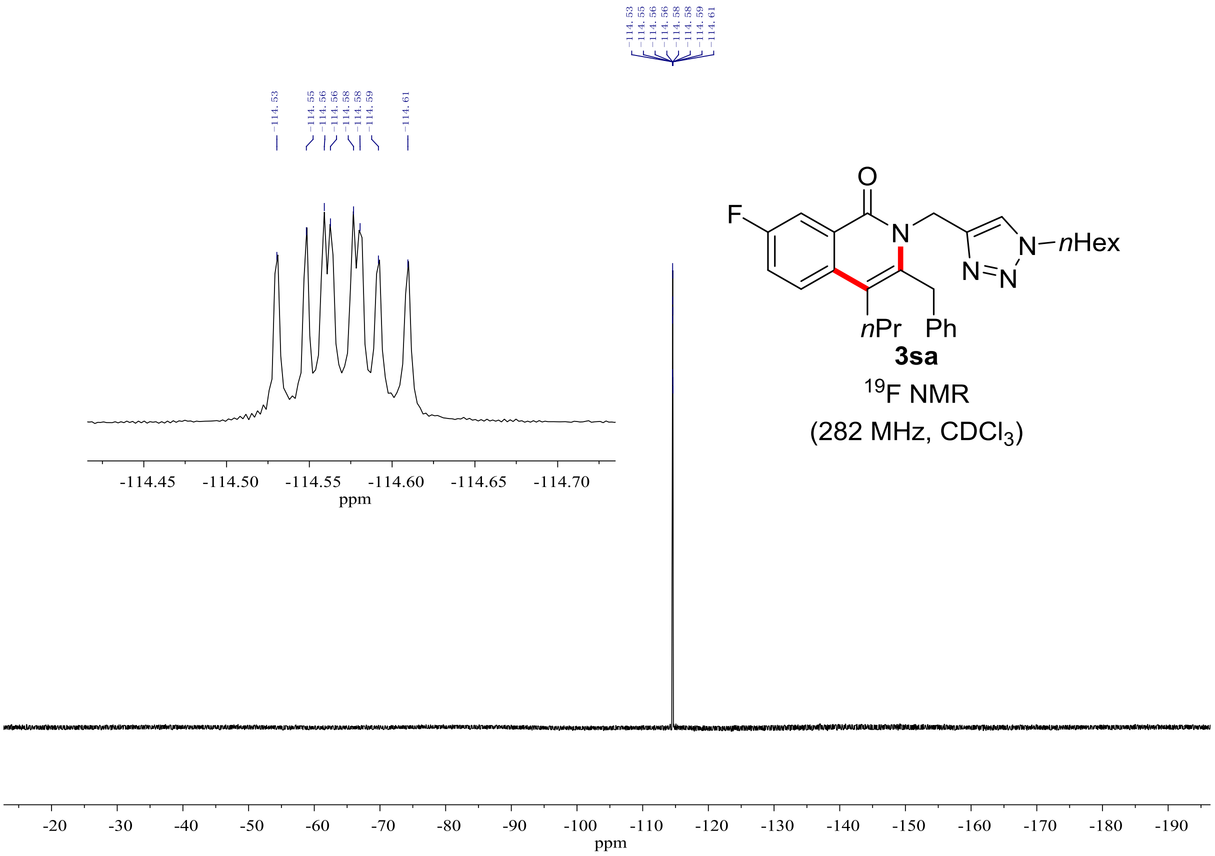

-114.53
-114.55
-114.56
-114.56
-114.58
-114.58
-114.59
-114.61
-114.45 -114.50 -114.55 -114.60 -114.65 -114.70
ppm
F
O
N
N–nHex
N=N
nPr
Ph
3sa
^{19}F NMR
(282 MHz, $CDCl_3$)
-20 -30 -40 -50 -60 -70 -80 -90 -100 -110 -120 -130 -140 -150 -160 -170 -180 -190
ppm

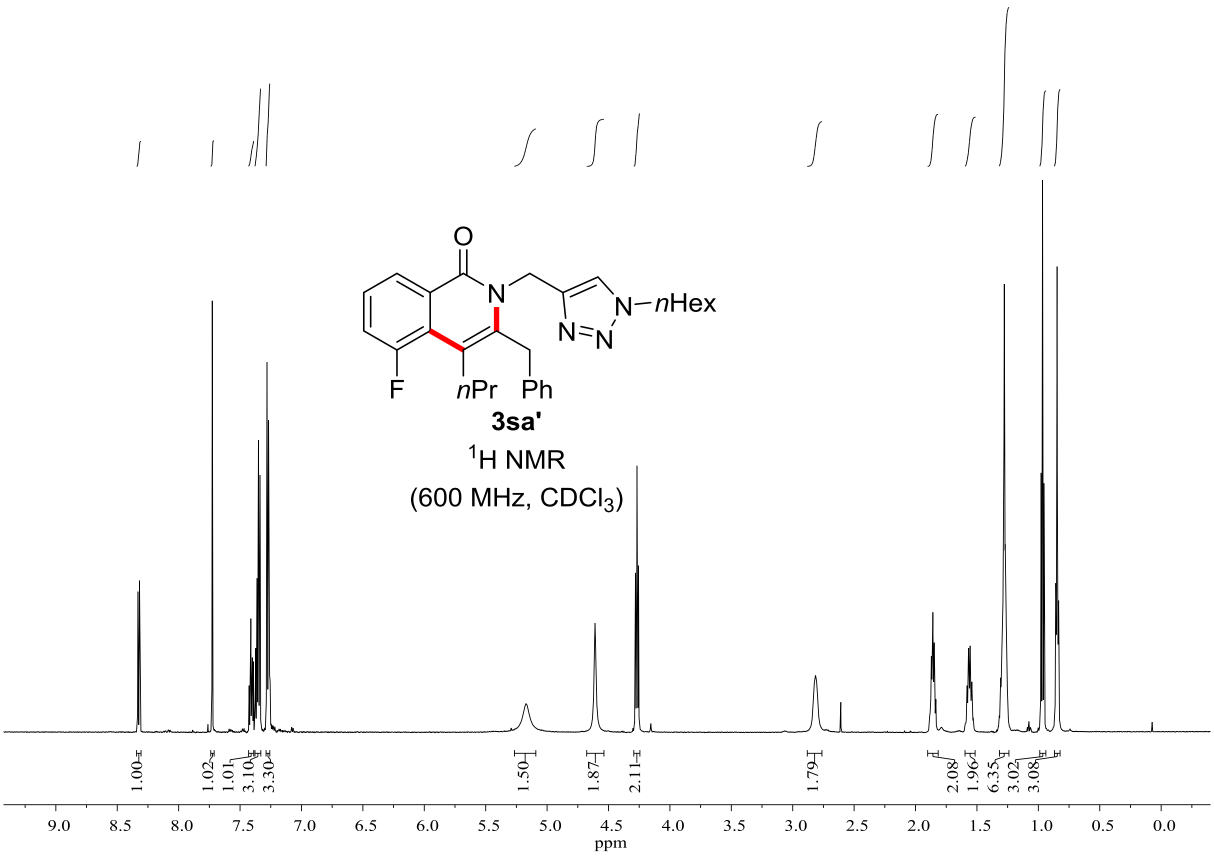

O
N
N−nHex
N=N
F
nPr
Ph
3sa'
1H NMR
(600 MHz, CDCl3)
1.00
1.02
1.01
3.10
3.30
1.50
1.87
2.11
1.79
2.08
1.96
6.35
3.02
3.08
9.0
8.5
8.0
7.5
7.0
6.5
6.0
5.5
5.0
4.5
4.0
3.5
3.0
2.5
2.0
1.5
1.0
0.5
0.0
ppm

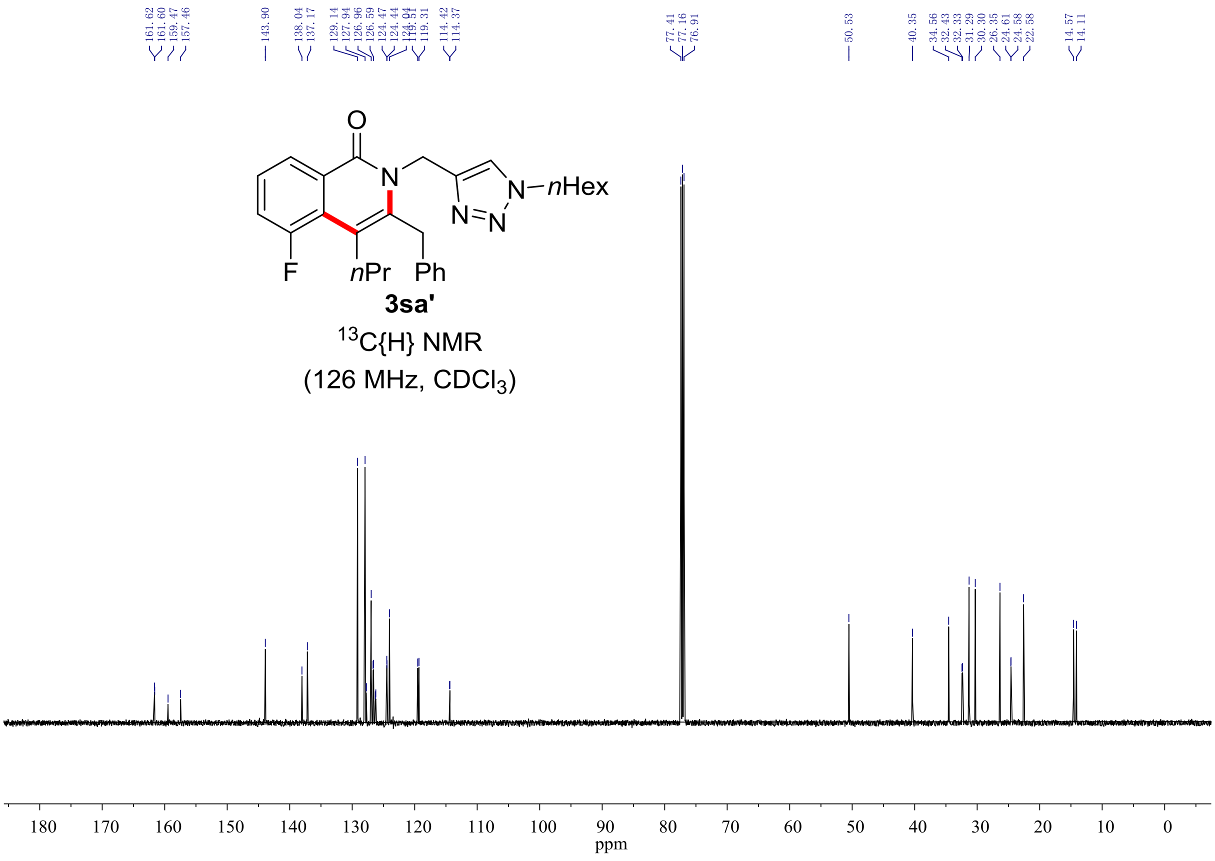

161.62
161.60
159.47
157.46
143.90
138.04
137.17
129.14
127.94
126.96
126.59
124.47
124.44
119.31
114.42
114.37
77.41
77.16
76.91
50.53
40.35
34.56
32.43
32.33
31.29
30.30
26.35
24.61
24.58
22.58
14.57
14.11
O
N
N−nHex
N=N
F
nPr
Ph
3sa'
13C{H} NMR
(126 MHz, CDCl3)
180
170
160
150
140
130
120
110
100
90
80
70
60
50
40
30
20
10
0
ppm

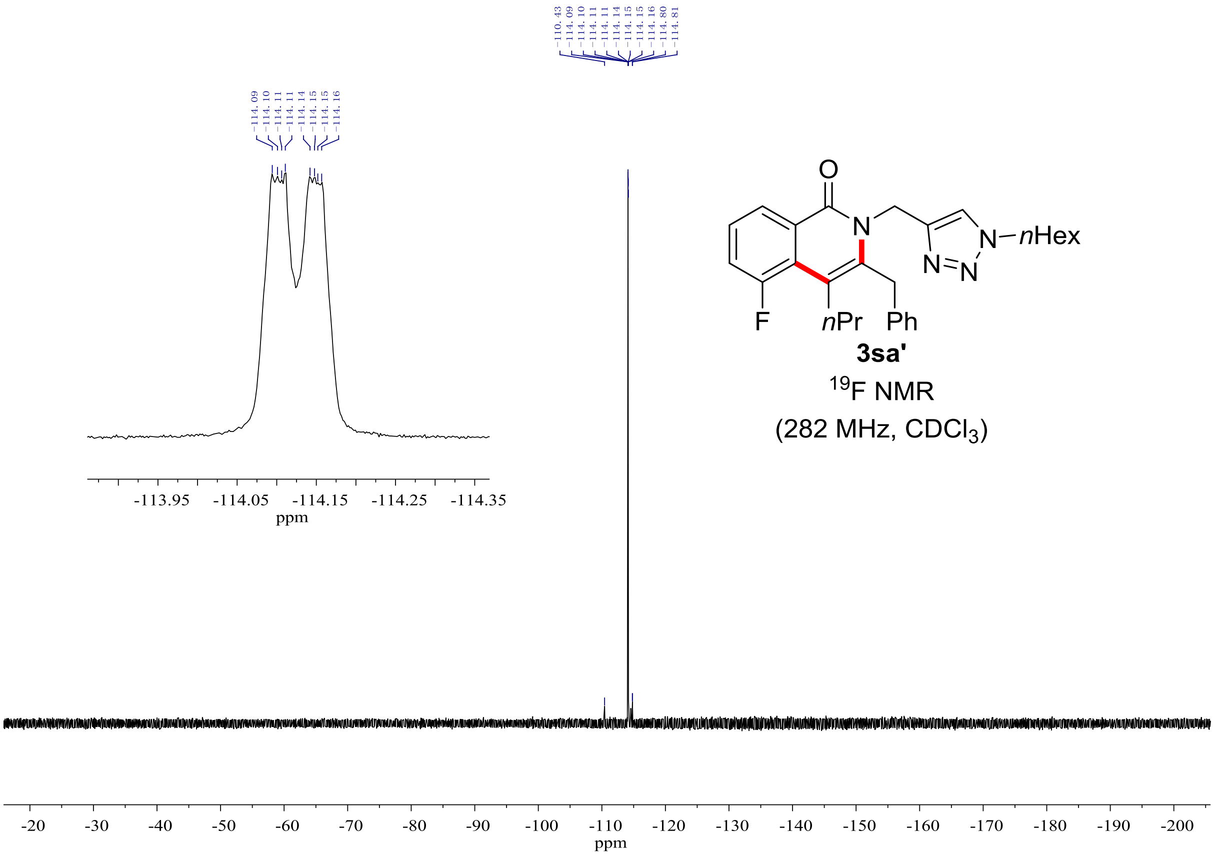

3sa'

^{19}F NMR

(282 MHz, $CDCl_3$)

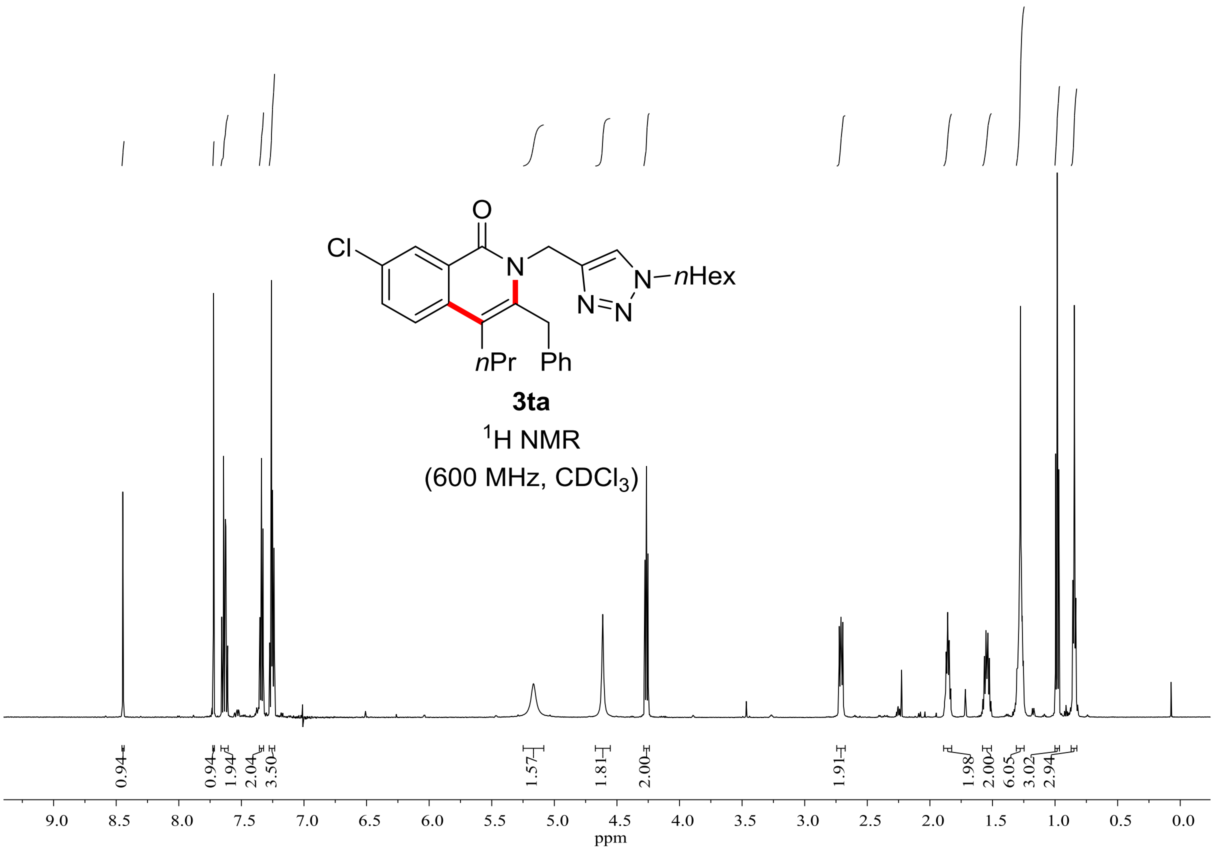
3ta
1H NMR
(600 MHz, CDCl3)
ppm

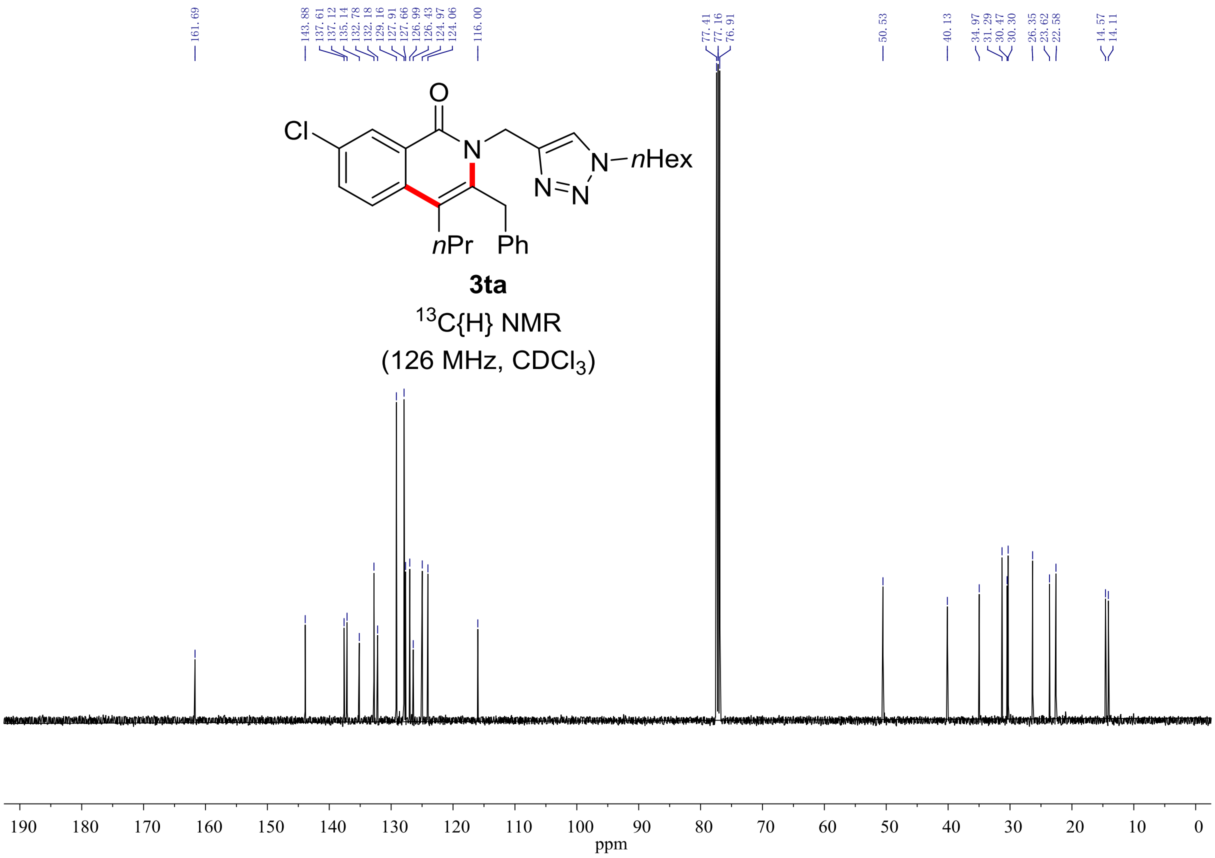
3ta
13C{H} NMR
(126 MHz, CDCl3)
ppm

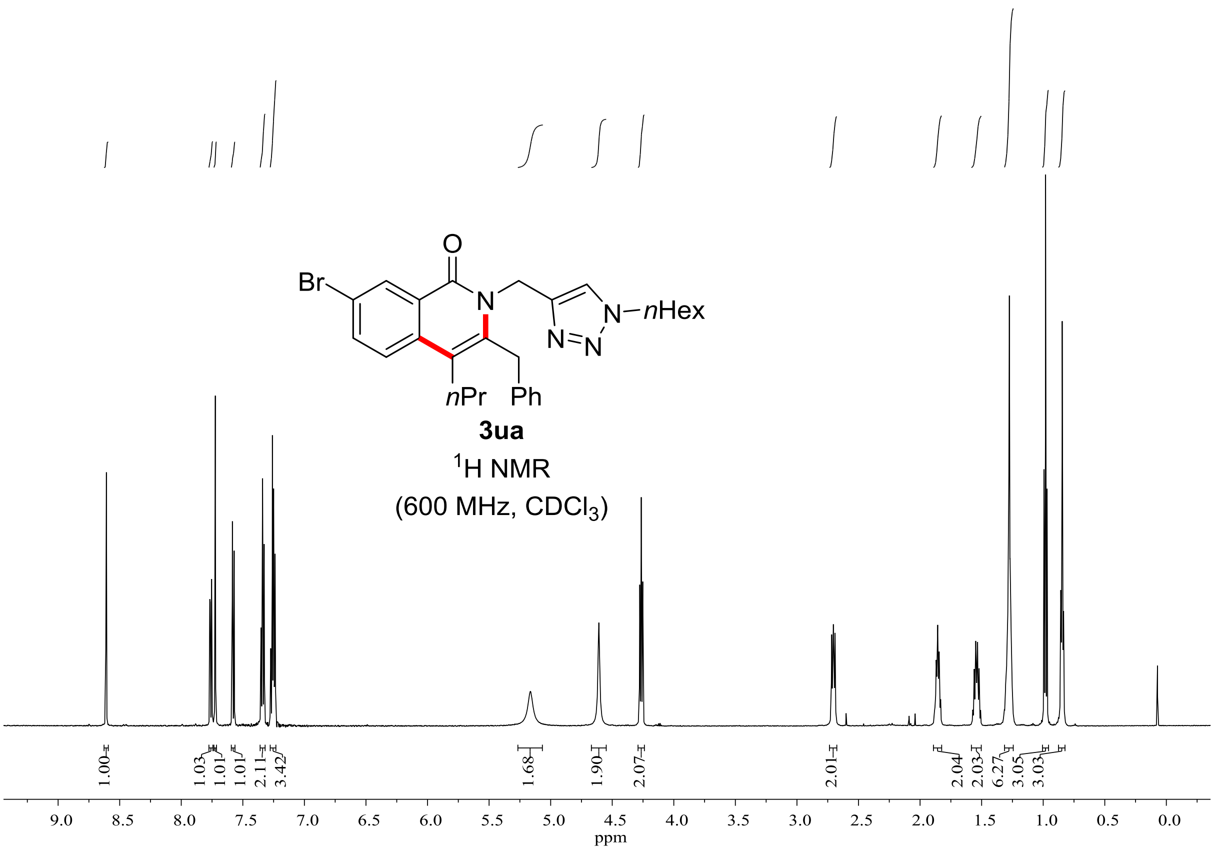
Br
O
N
N
N=N
nHex
nPr
Ph
3ua
^{1}H NMR
(600 MHz, CDCl$_3$)
ppm

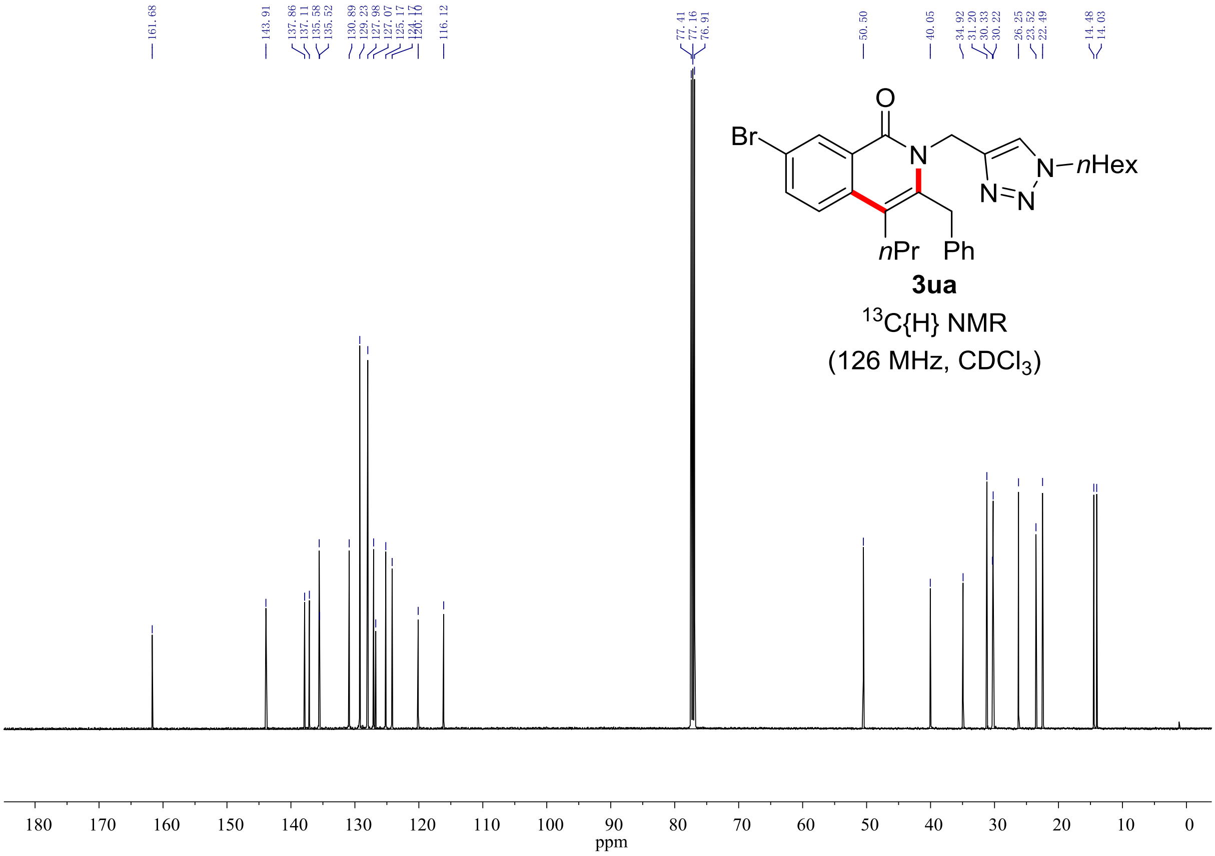
Br
O
N
N
N=N
nHex
nPr
Ph
3ua
^{13}C{H} NMR
(126 MHz, CDCl$_3$)
ppm

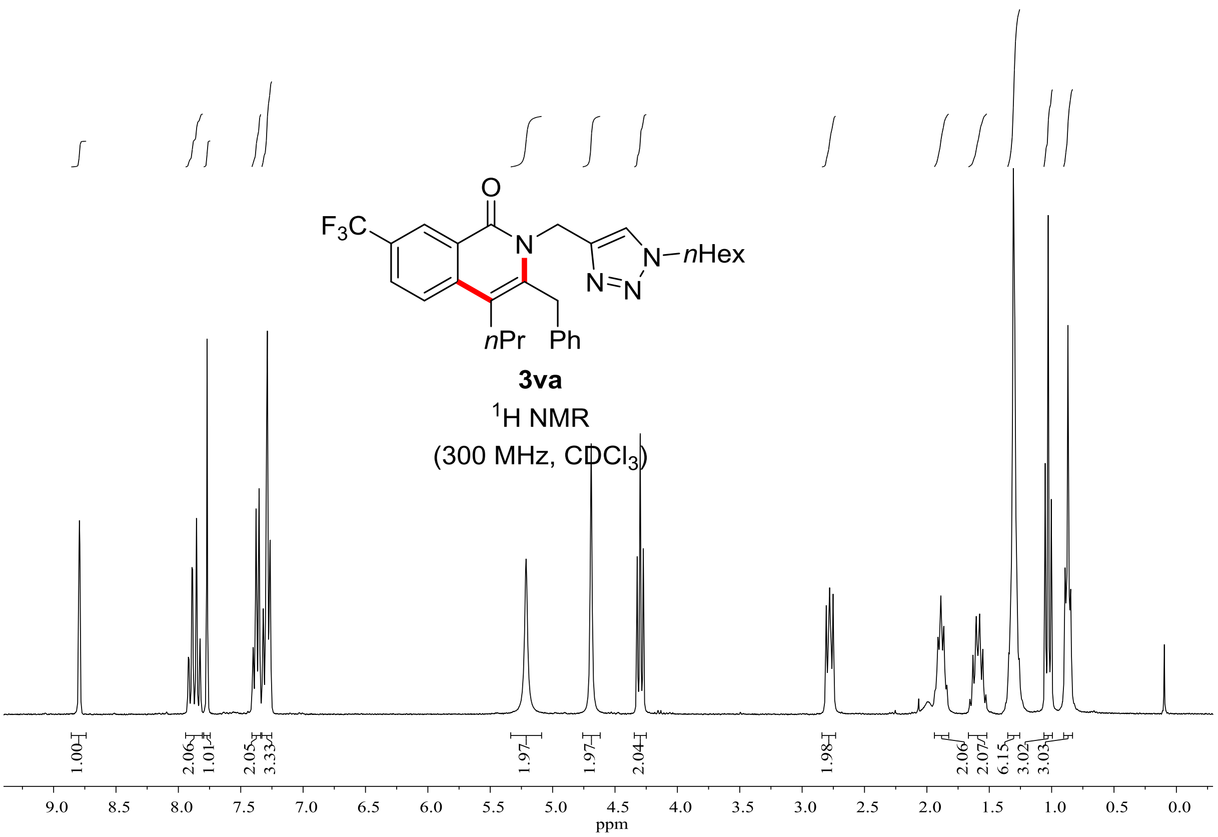
F3C
O
N
N−nHex
N=N
nPr
Ph
3va
1H NMR
(300 MHz, CDCl3)
1.00
2.06
1.01
2.05
3.33
1.97
1.97
2.04
1.98
2.06
2.07
6.15
3.02
3.03
9.0
8.5
8.0
7.5
7.0
6.5
6.0
5.5
5.0
4.5
4.0
3.5
3.0
2.5
2.0
1.5
1.0
0.5
0.0
ppm

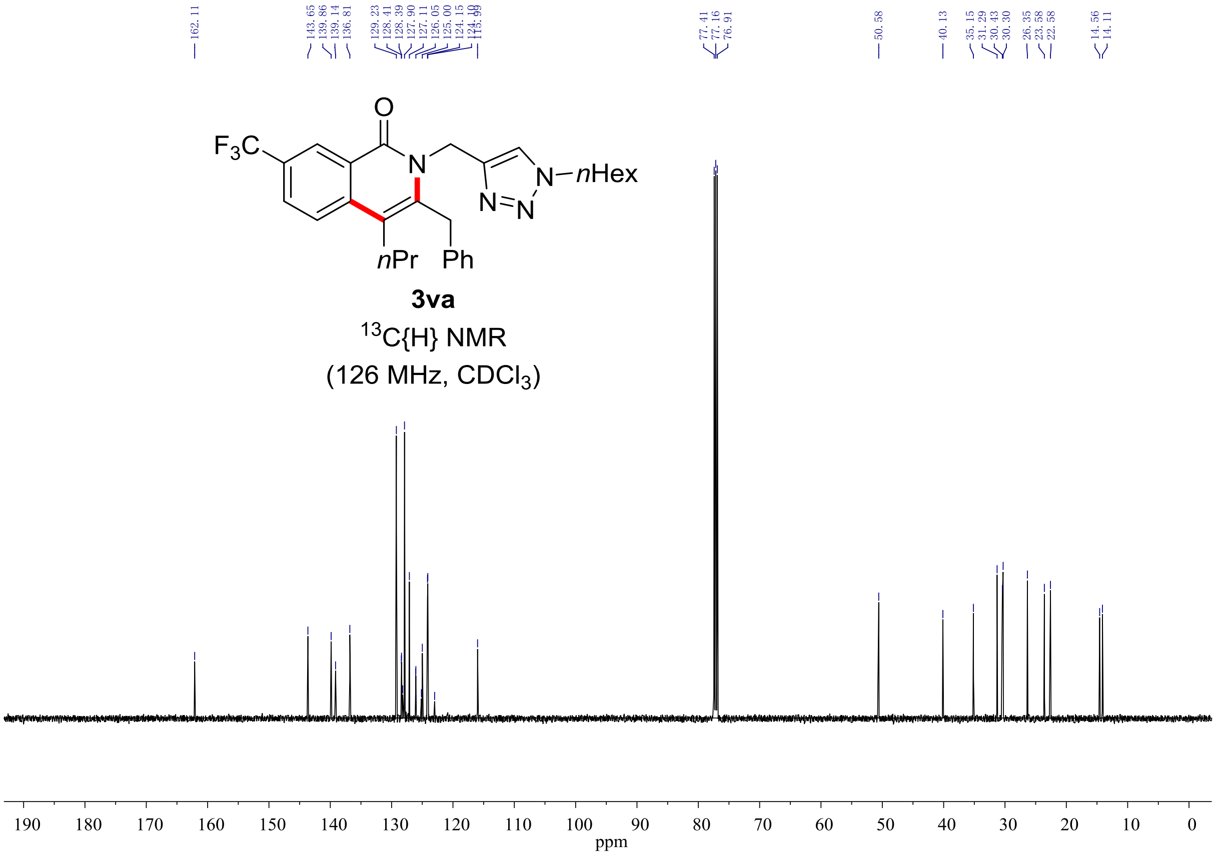
162.11
143.65
139.86
139.14
136.81
129.23
128.41
128.39
127.90
127.11
126.05
125.00
124.15
77.41
77.16
76.91
50.58
40.13
35.15
31.29
30.43
30.30
26.35
23.58
22.58
14.56
14.11
F3C
O
N
N−nHex
N=N
nPr
Ph
3va
13C{H} NMR
(126 MHz, CDCl3)
190
180
170
160
150
140
130
120
110
100
90
80
70
60
50
40
30
20
10
0
ppm

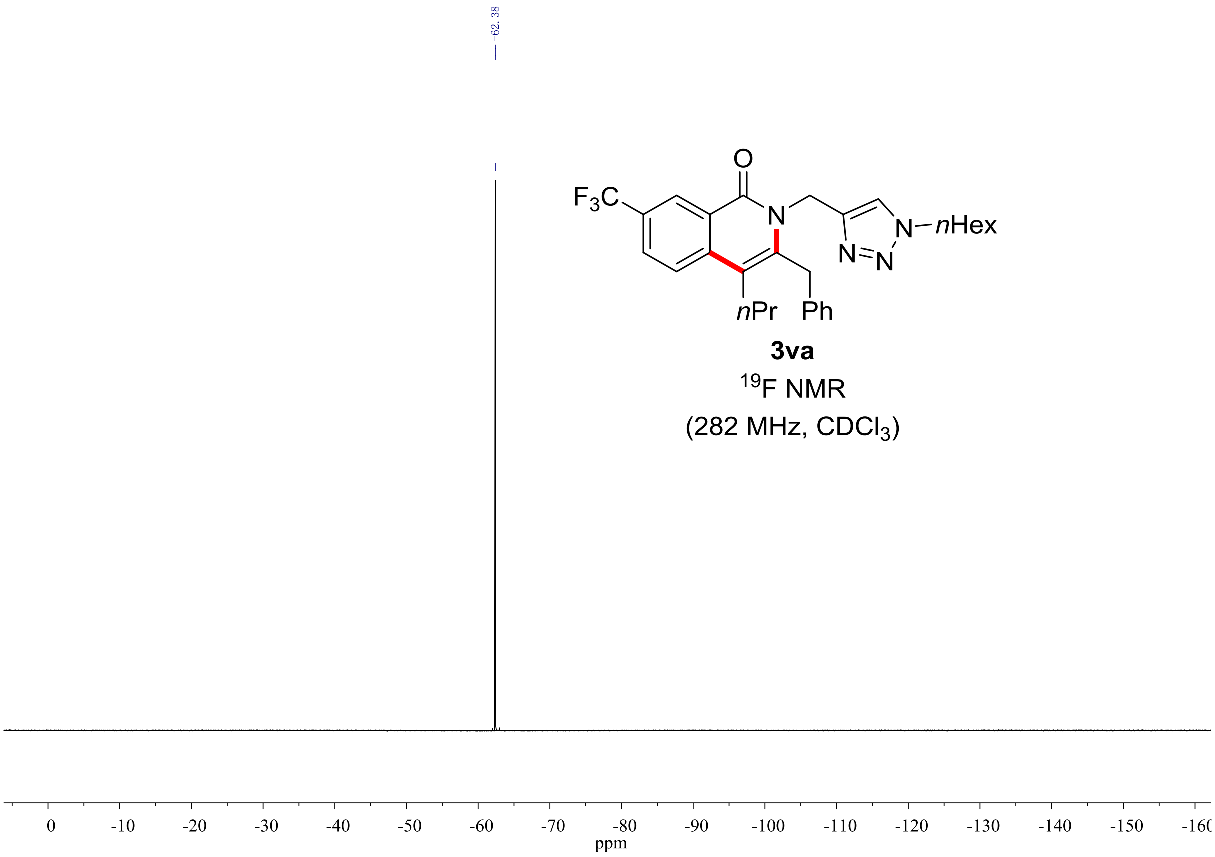

-62.38
F3C
O
N
N
N=N
N–nHex
nPr
Ph
3va
19F NMR
(282 MHz, CDCl3)
0
-10
-20
-30
-40
-50
-60
-70
-80
-90
-100
-110
-120
-130
-140
-150
-160
ppm

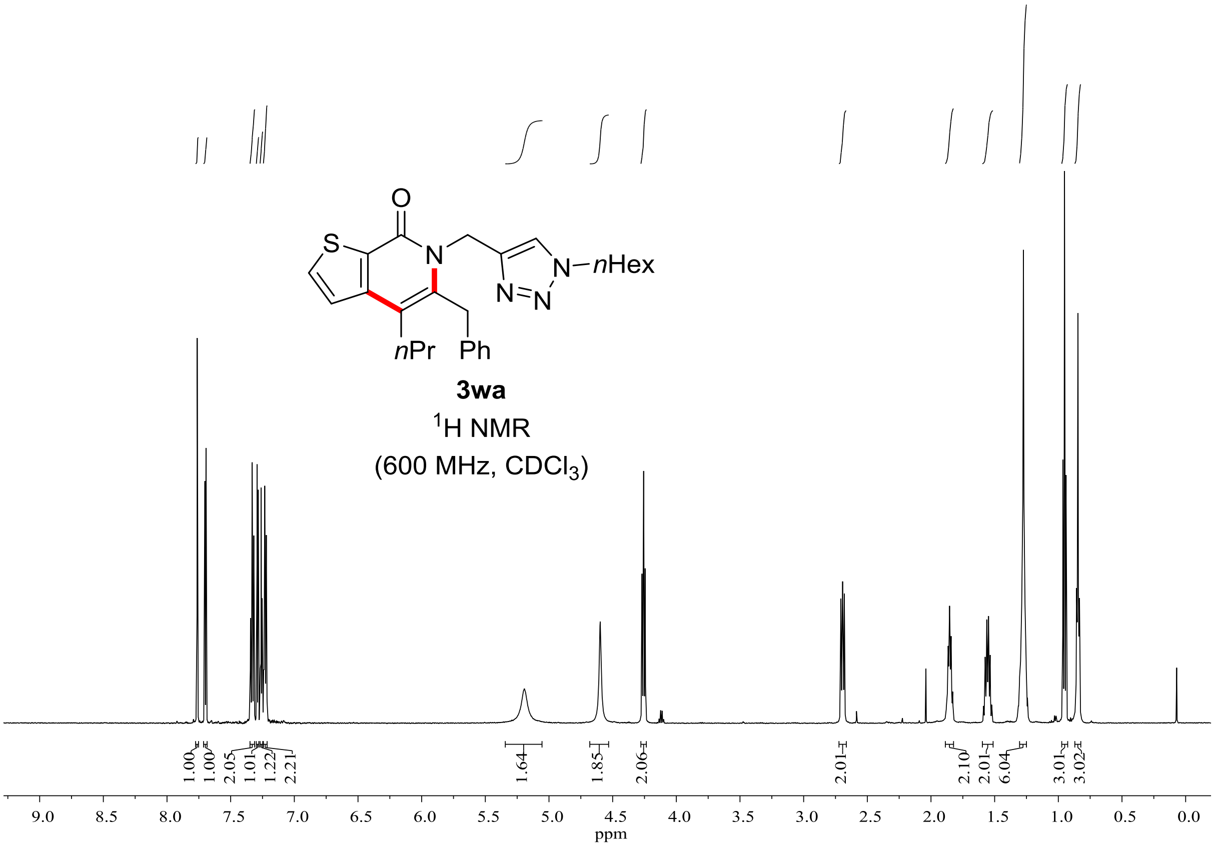
O
S
N
N−nHex
N=N
nPr
Ph
3wa
1H NMR
(600 MHz, CDCl3)
1.00 1.00 2.05 1.01 1.22 2.21 1.64 1.85 2.06 2.01 2.10 2.01 6.04 3.01 3.02
9.0 8.5 8.0 7.5 7.0 6.5 6.0 5.5 5.0 4.5 4.0 3.5 3.0 2.5 2.0 1.5 1.0 0.5 0.0
ppm

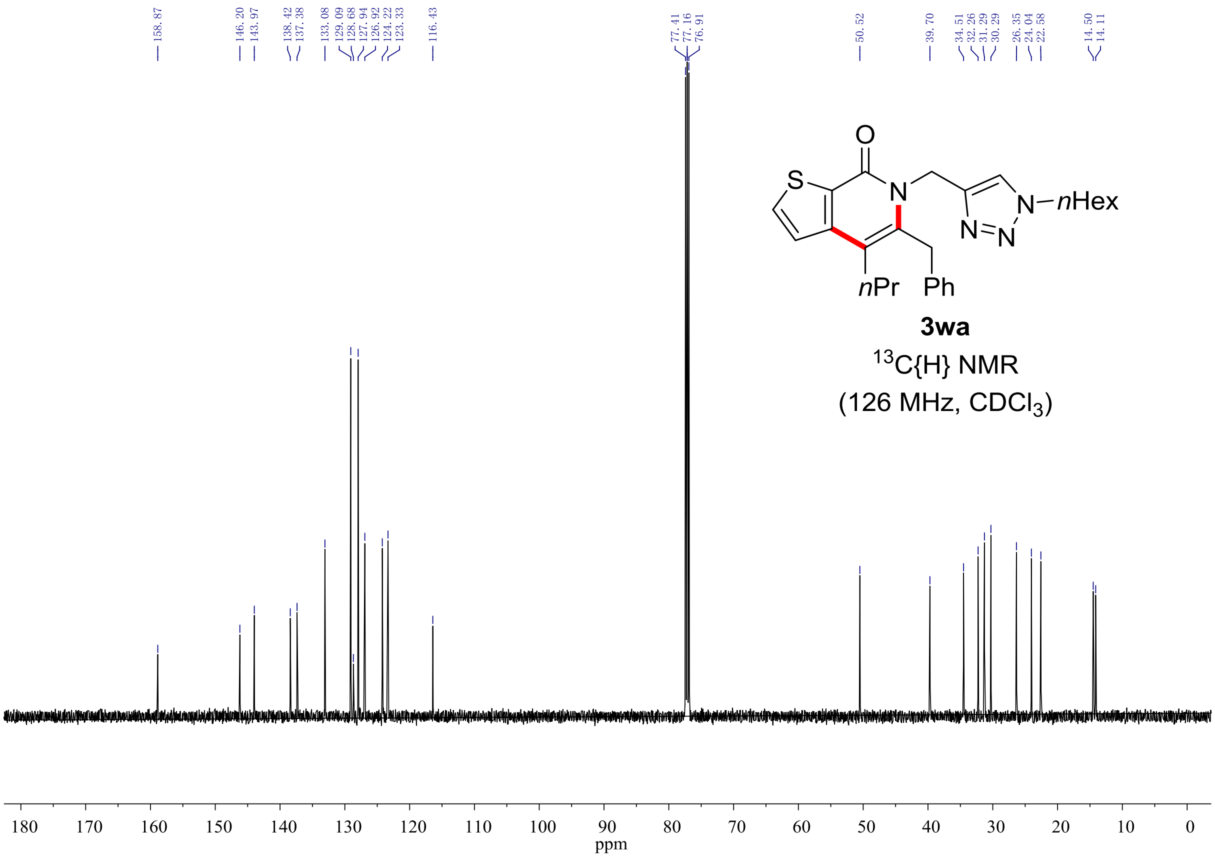
158.87
146.20 143.97
138.42 137.38
133.08 129.09 128.68 127.94 126.92 124.22 123.33
116.43
77.41 77.16 76.91
50.52
39.70
34.51 32.26 31.29 30.29
26.35 24.04 22.58
14.50 14.11
O
S
N
N−nHex
N=N
nPr
Ph
3wa
13C{H} NMR
(126 MHz, CDCl3)
180 170 160 150 140 130 120 110 100 90 80 70 60 50 40 30 20 10 0
ppm

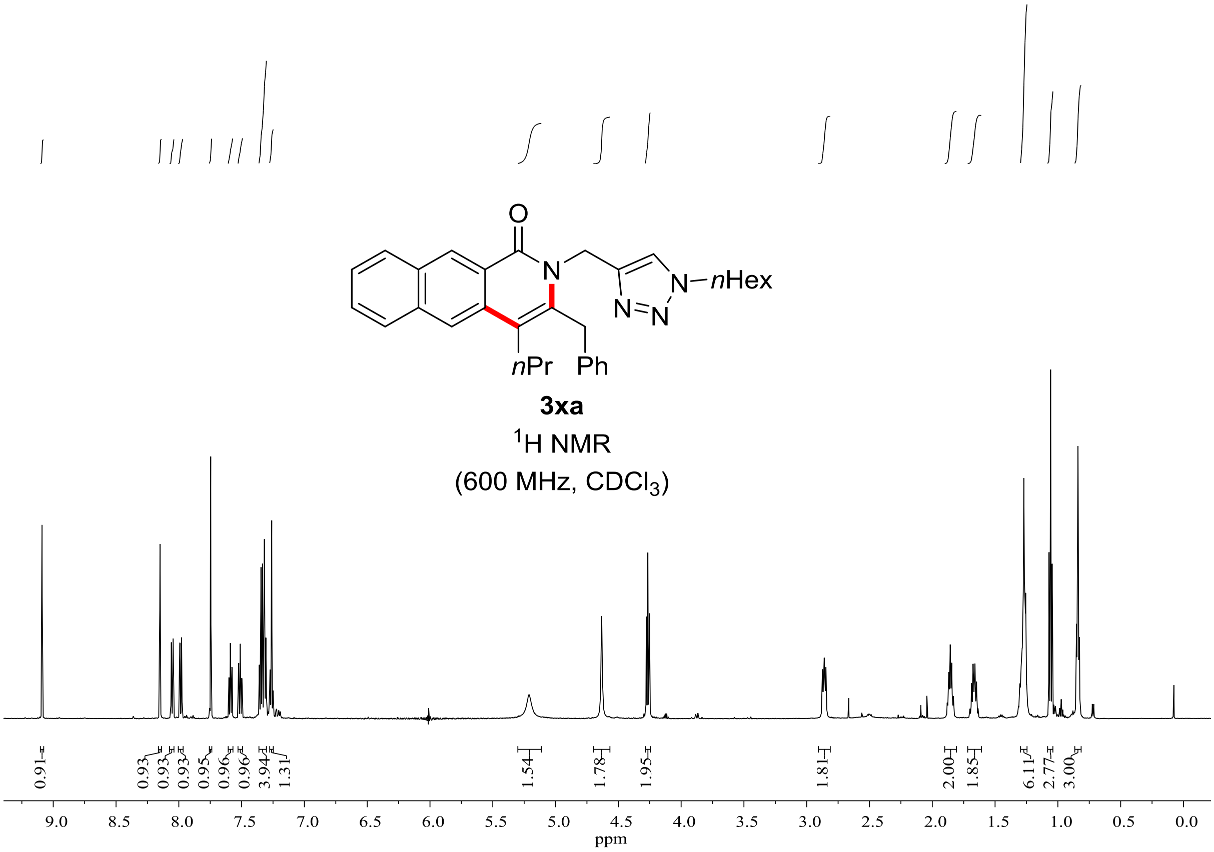
3xa
1H NMR
(600 MHz, CDCl3)
0.91 0.93 0.93 0.93 0.95 0.96 0.96 3.94 1.31 1.54 1.78 1.95 1.81 2.00 1.85 6.11 2.77 3.00
ppm

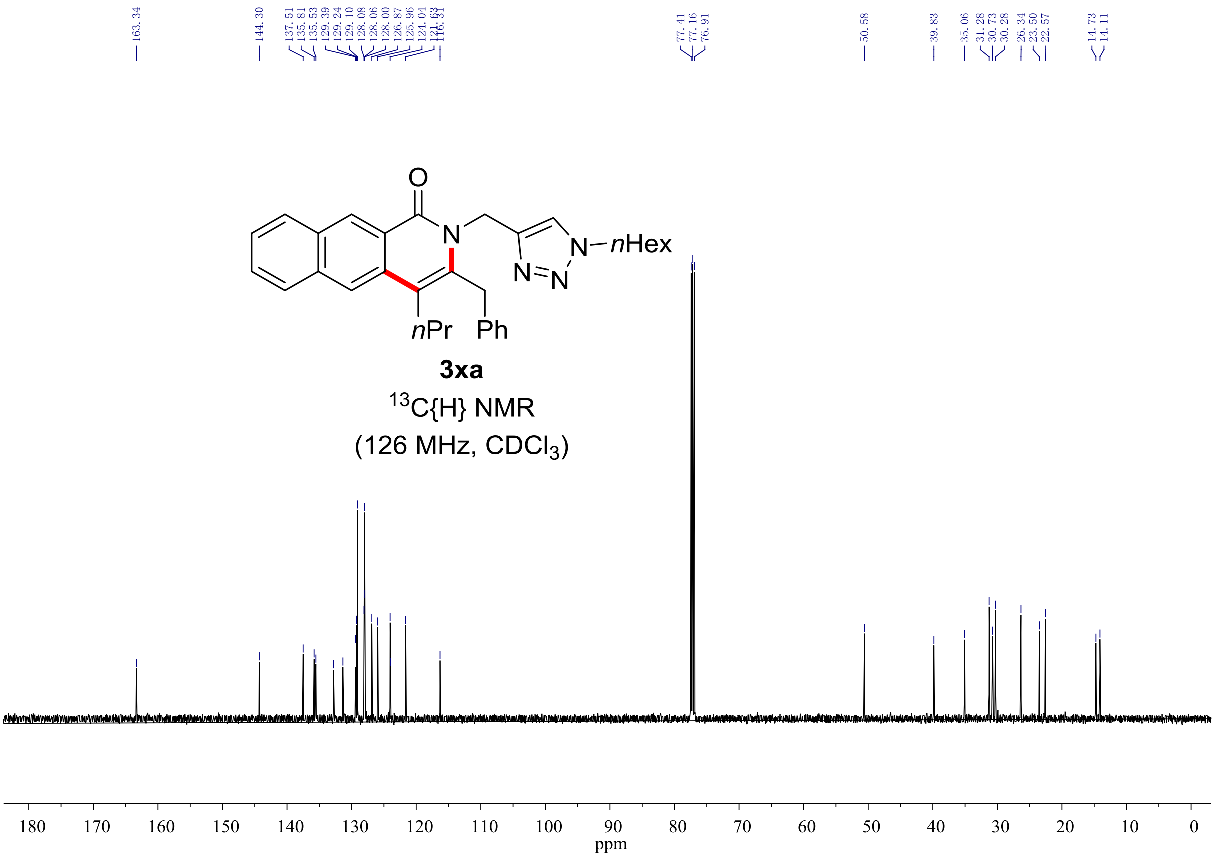
163.34 144.30 137.51 135.81 135.53 129.39 129.24 129.10 128.08 128.06 128.00 126.87 125.96 124.04
77.41 77.16 76.91
50.58
39.83 35.06 31.28 30.73 30.28 26.34 23.50 22.57
14.73 14.11
3xa
13C{H} NMR
(126 MHz, CDCl3)
ppm

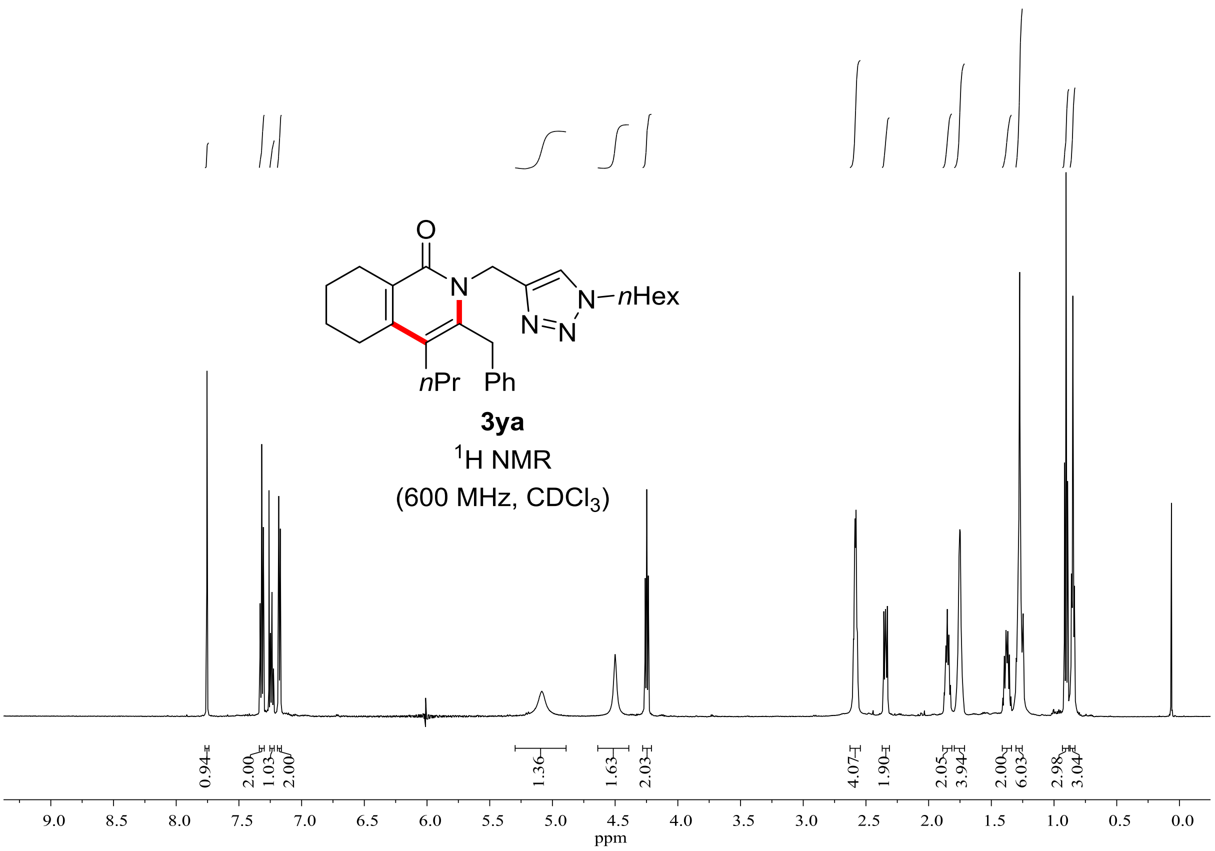

O
N
N−nHex
N=N
nPr
Ph
3ya
¹H NMR
(600 MHz, CDCl3)
ppm

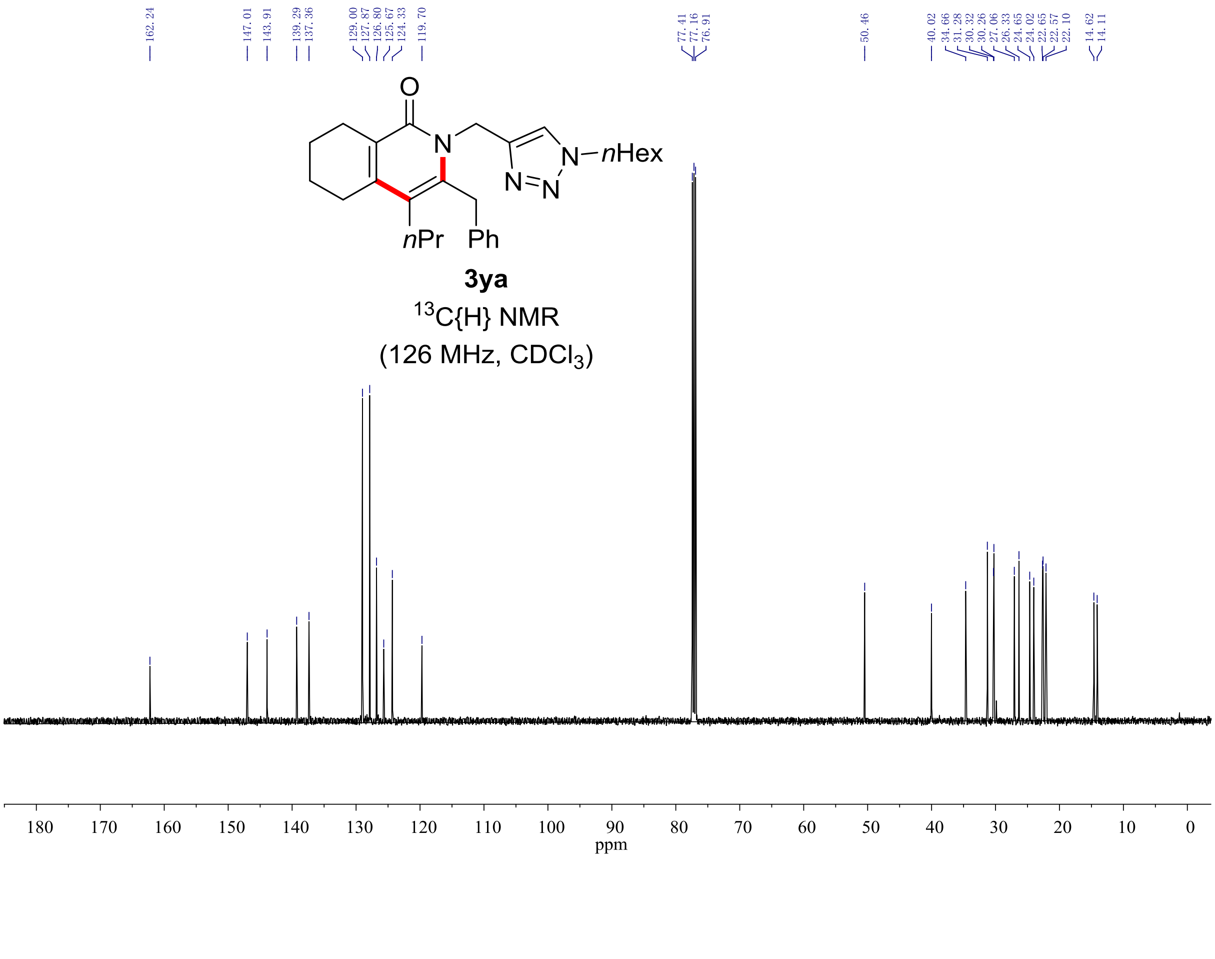

O
N
N−nHex
N=N
nPr
Ph
3ya
¹³C{H} NMR
(126 MHz, CDCl3)
ppm

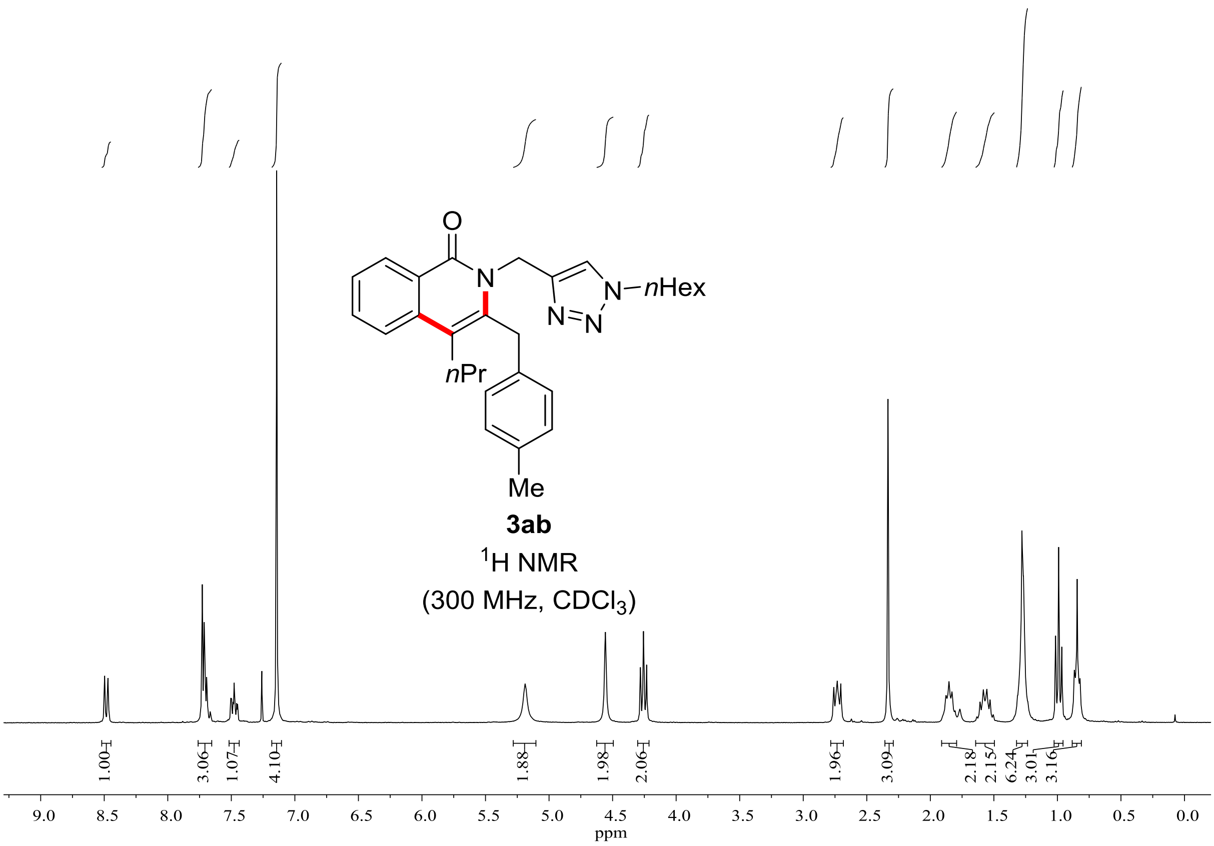
O
N
N
N=N
nHex
nPr
Me
3ab
1H NMR
(300 MHz, CDCl3)
1.00
3.06
1.07
4.10
1.88
1.98
2.06
1.96
3.09
2.18
2.15
6.24
3.01
3.16
9.0
8.5
8.0
7.5
7.0
6.5
6.0
5.5
5.0
4.5
4.0
3.5
3.0
2.5
2.0
1.5
1.0
0.5
0.0
ppm

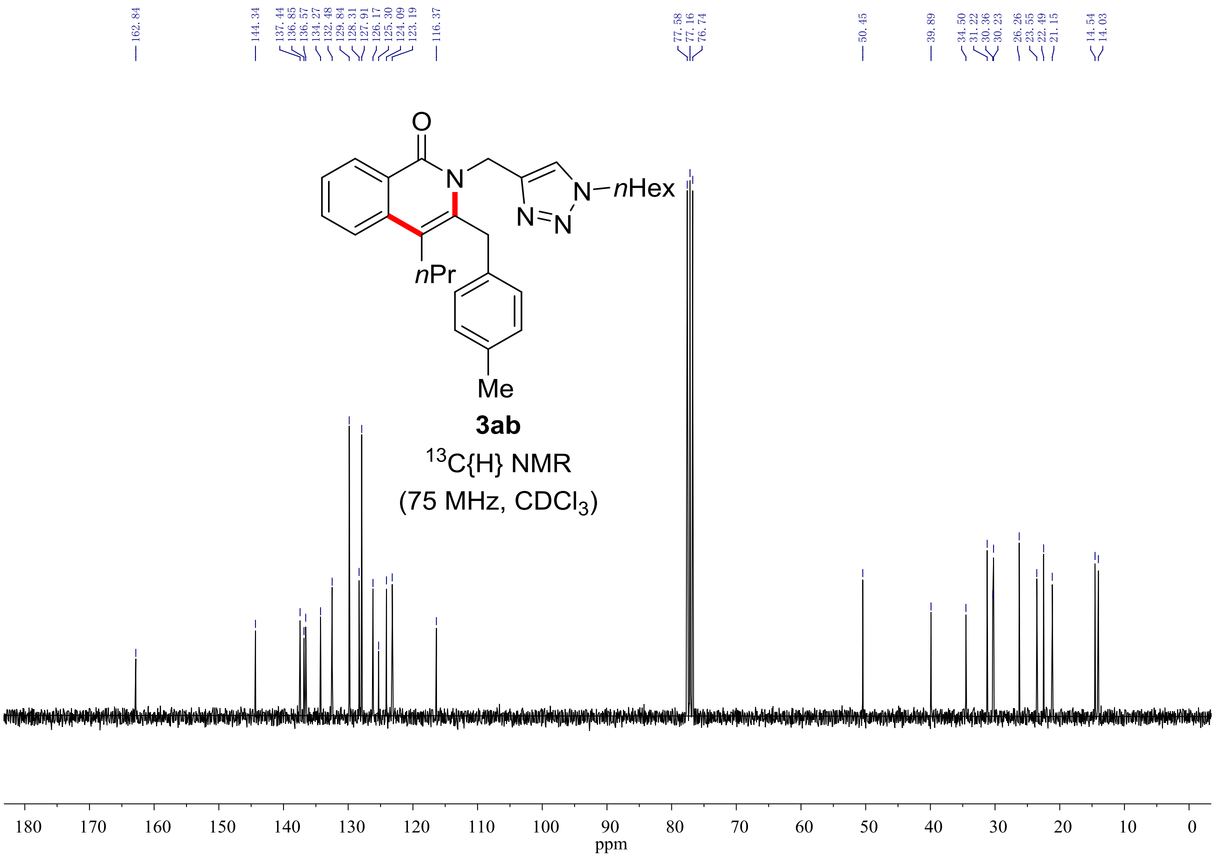
162.84
144.34
137.44
136.85
136.57
134.27
132.48
129.84
128.31
127.91
126.17
125.30
124.09
123.19
116.37
77.58
77.16
76.74
50.45
39.89
34.50
31.22
30.36
30.23
26.26
23.55
22.49
21.15
14.54
14.03
O
N
N
N=N
nHex
nPr
Me
3ab
13C{H} NMR
(75 MHz, CDCl3)
180
170
160
150
140
130
120
110
100
90
80
70
60
50
40
30
20
10
0
ppm

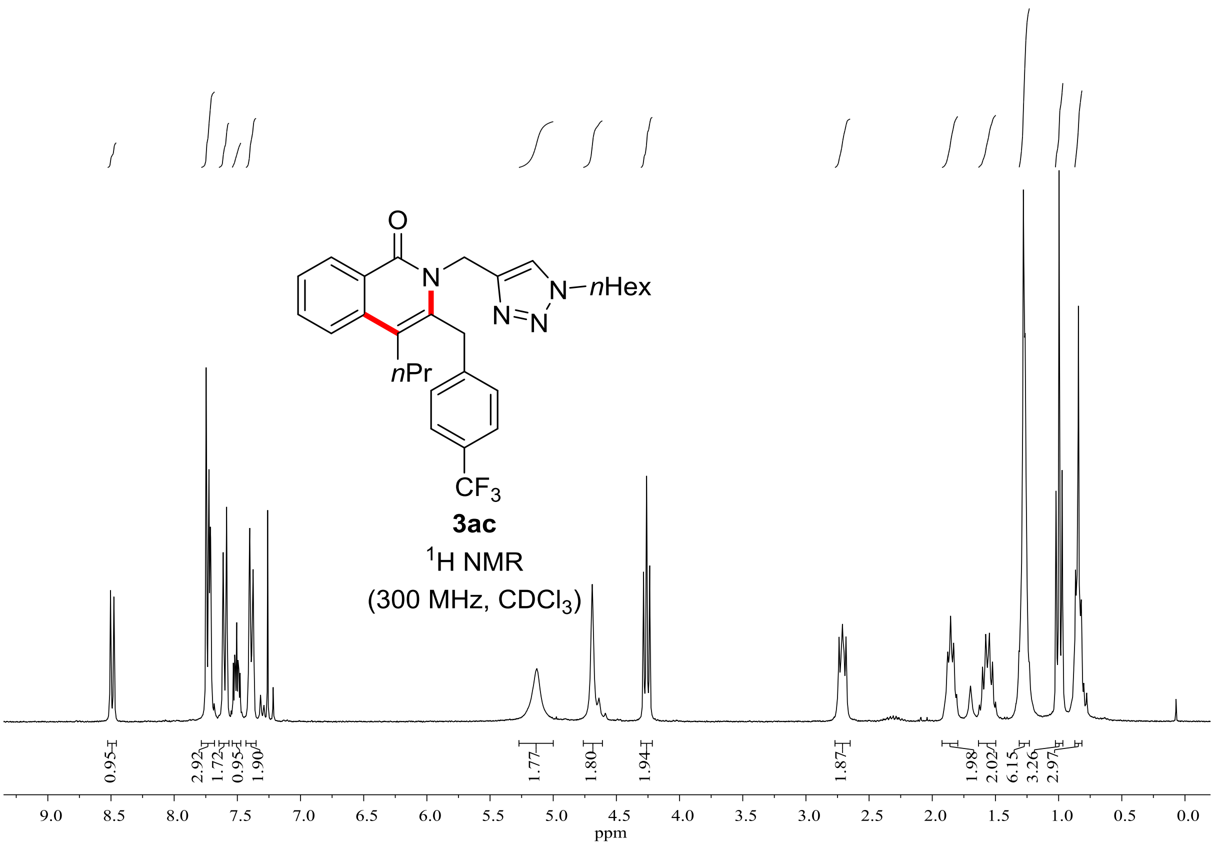
O
N
N−nHex
N=N
nPr
CF3
3ac
1H NMR
(300 MHz, CDCl3)
ppm

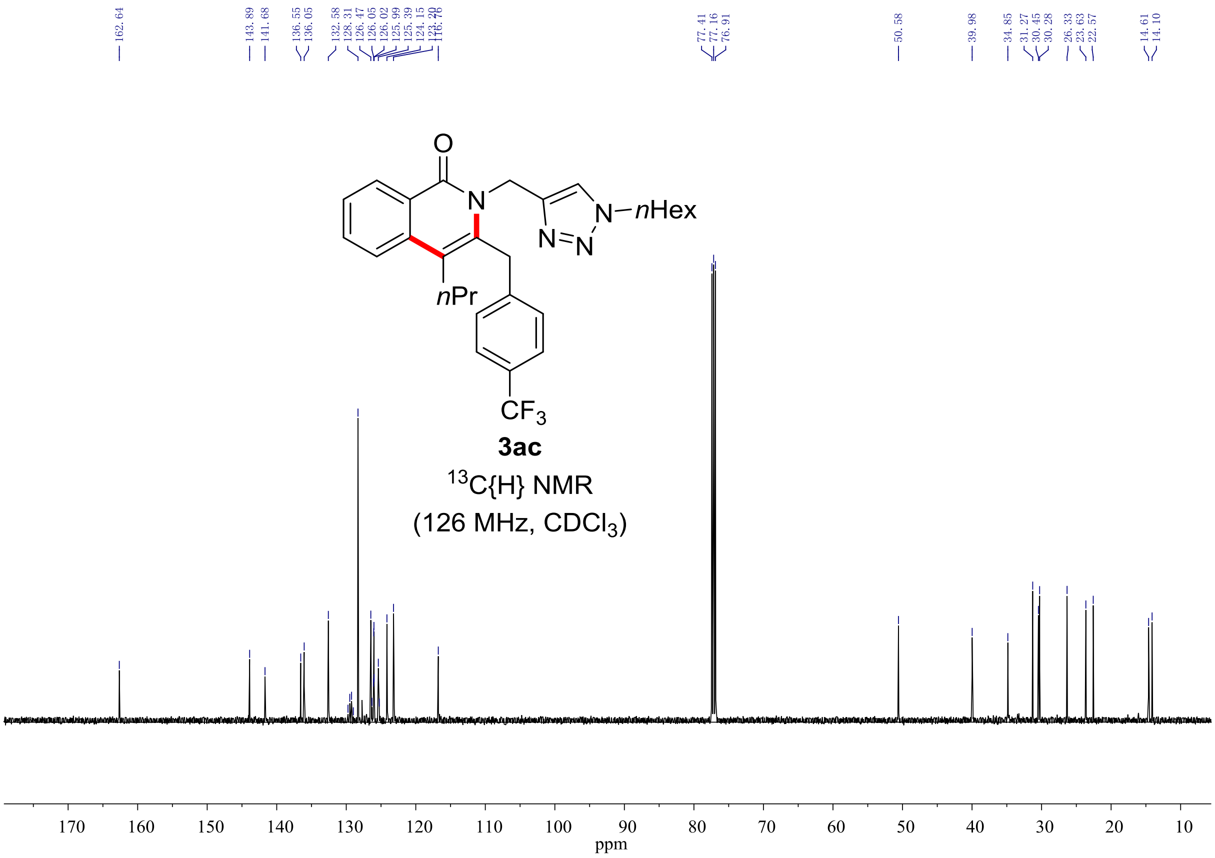
O
N
N−nHex
N=N
nPr
CF3
3ac
13C{H} NMR
(126 MHz, CDCl3)
ppm

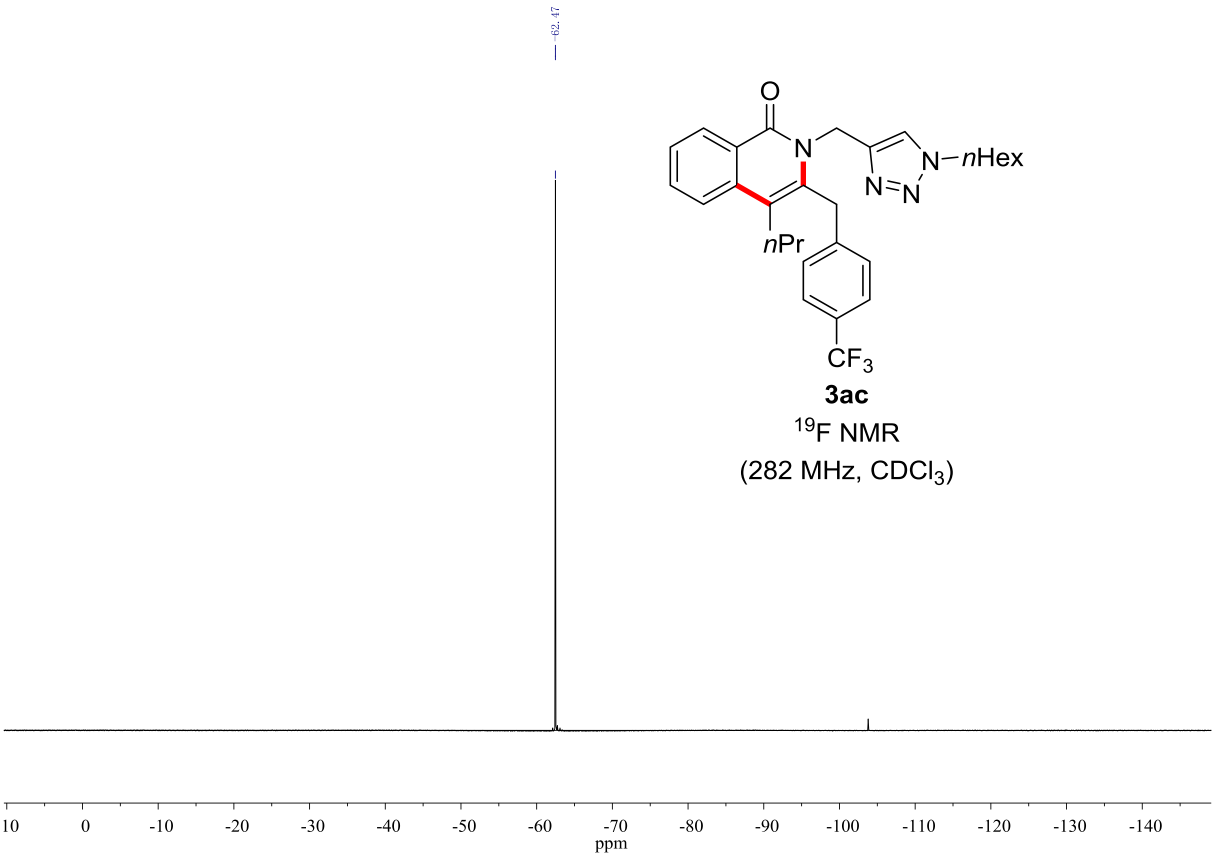
-62.47
O
N
N
N
N
nHex
nPr
CF3
3ac
19F NMR
(282 MHz, CDCl3)
10
0
-10
-20
-30
-40
-50
-60
-70
-80
-90
-100
-110
-120
-130
-140
ppm

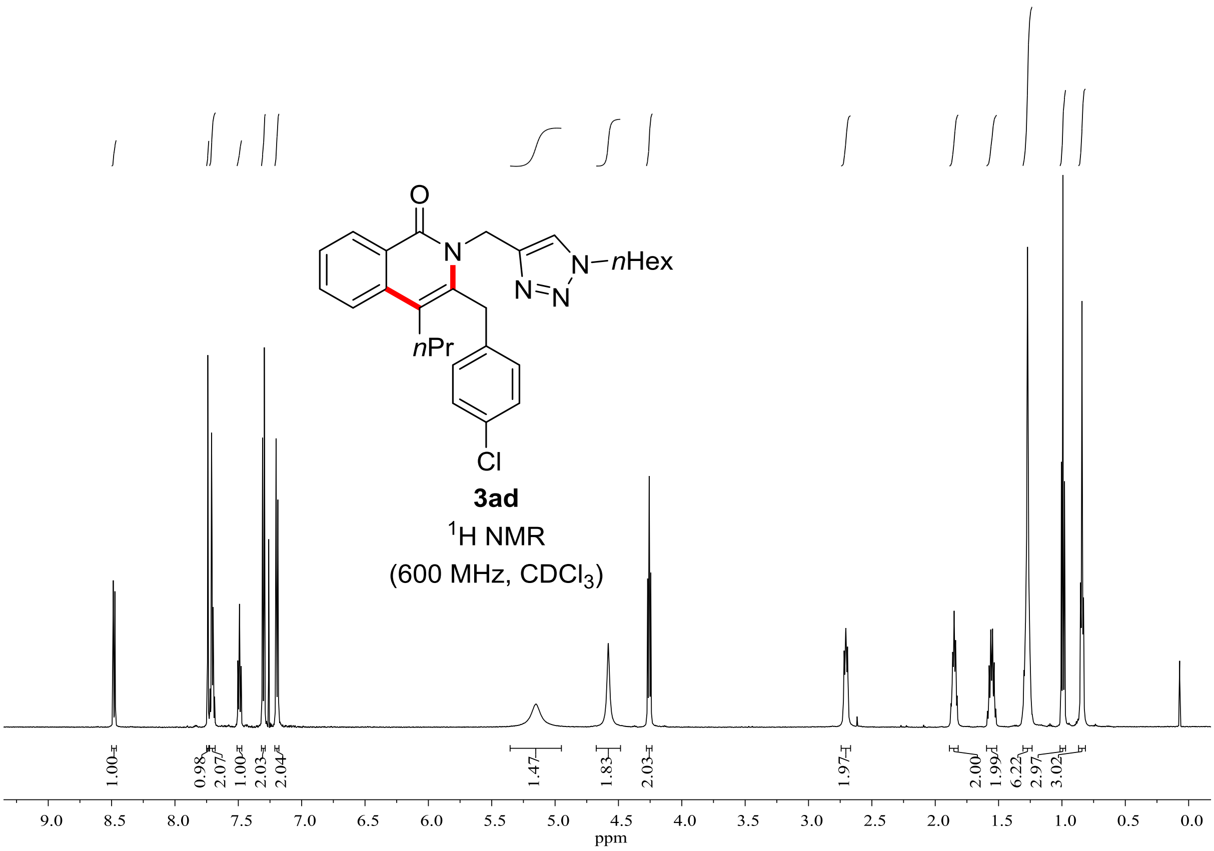

O
N
N
N=N
nHex
nPr
Cl
3ad
^{1}H NMR
(600 MHz, $CDCl_3$)
1.00
0.98
2.07
1.00
2.03
2.04
1.47
1.83
2.03
1.97
2.00
1.99
6.22
2.97
3.02
ppm

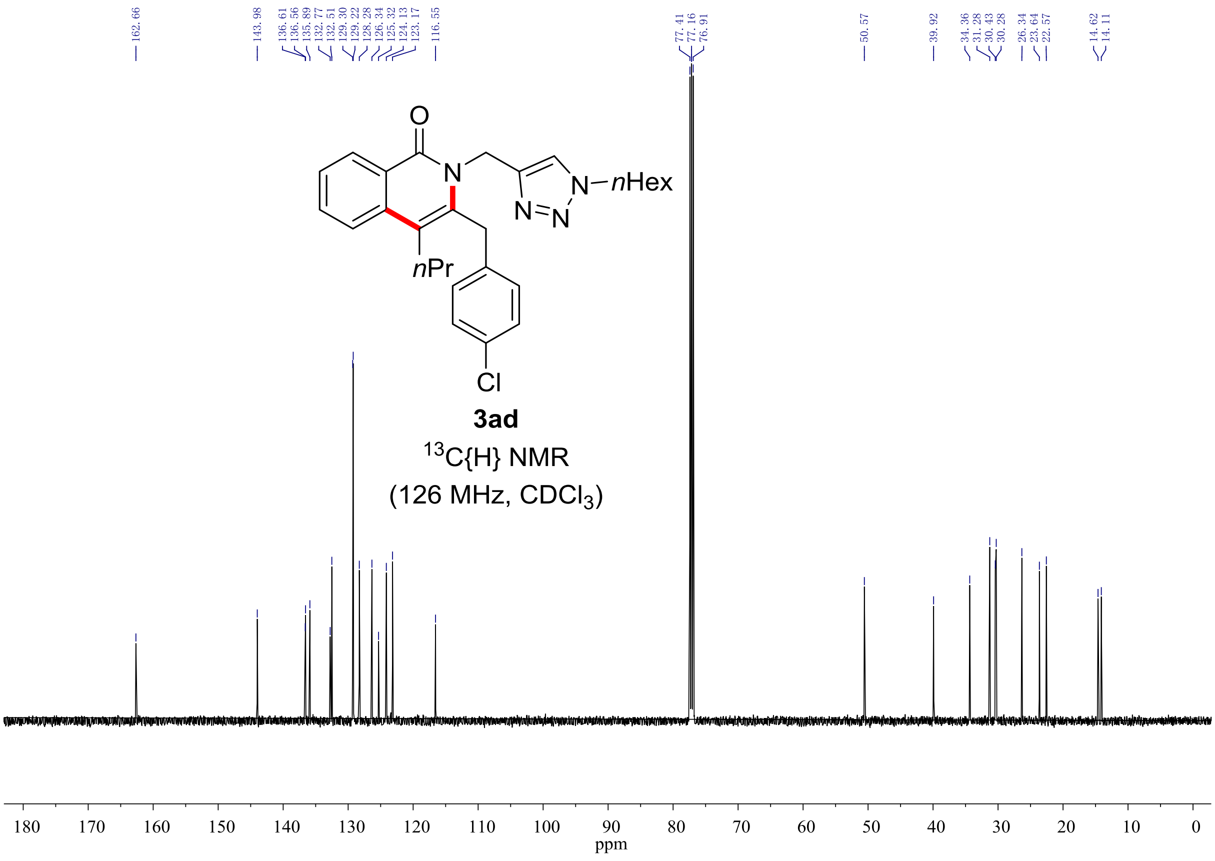

162.66
143.98
136.61
136.56
135.89
132.77
132.51
129.30
129.22
128.28
126.34
125.32
124.13
123.17
116.55
77.41
77.16
76.91
50.57
39.92
34.36
31.28
30.43
30.28
26.34
23.64
22.57
14.62
14.11
3ad
^{13}C{H} NMR
(126 MHz, $CDCl_3$)
ppm

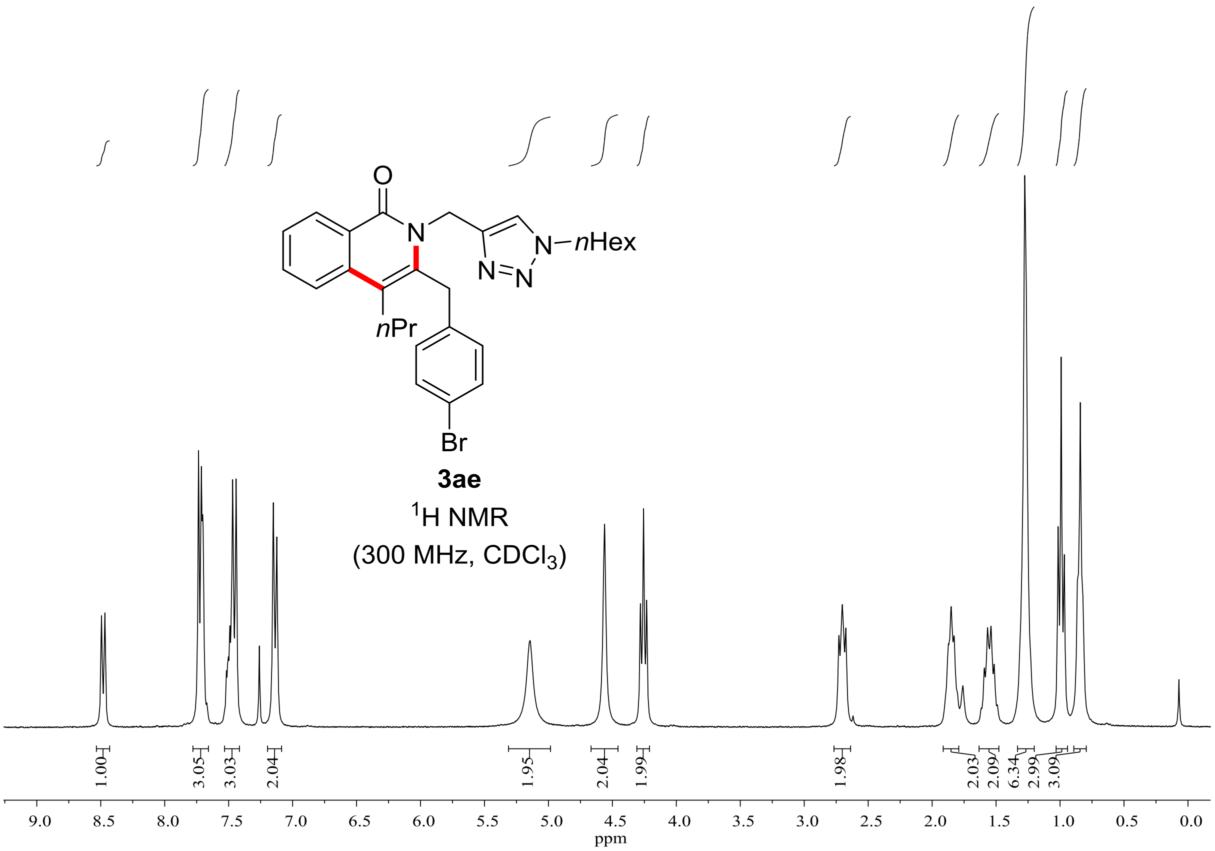
O
N
N
N
N
N−nHex
nPr
Br
3ae
1H NMR
(300 MHz, CDCl3)
1.00
3.05
3.03
2.04
1.95
2.04
1.99
1.98
2.03
2.09
6.34
2.99
3.09
9.0 8.5 8.0 7.5 7.0 6.5 6.0 5.5 5.0 4.5 4.0 3.5 3.0 2.5 2.0 1.5 1.0 0.5 0.0
ppm

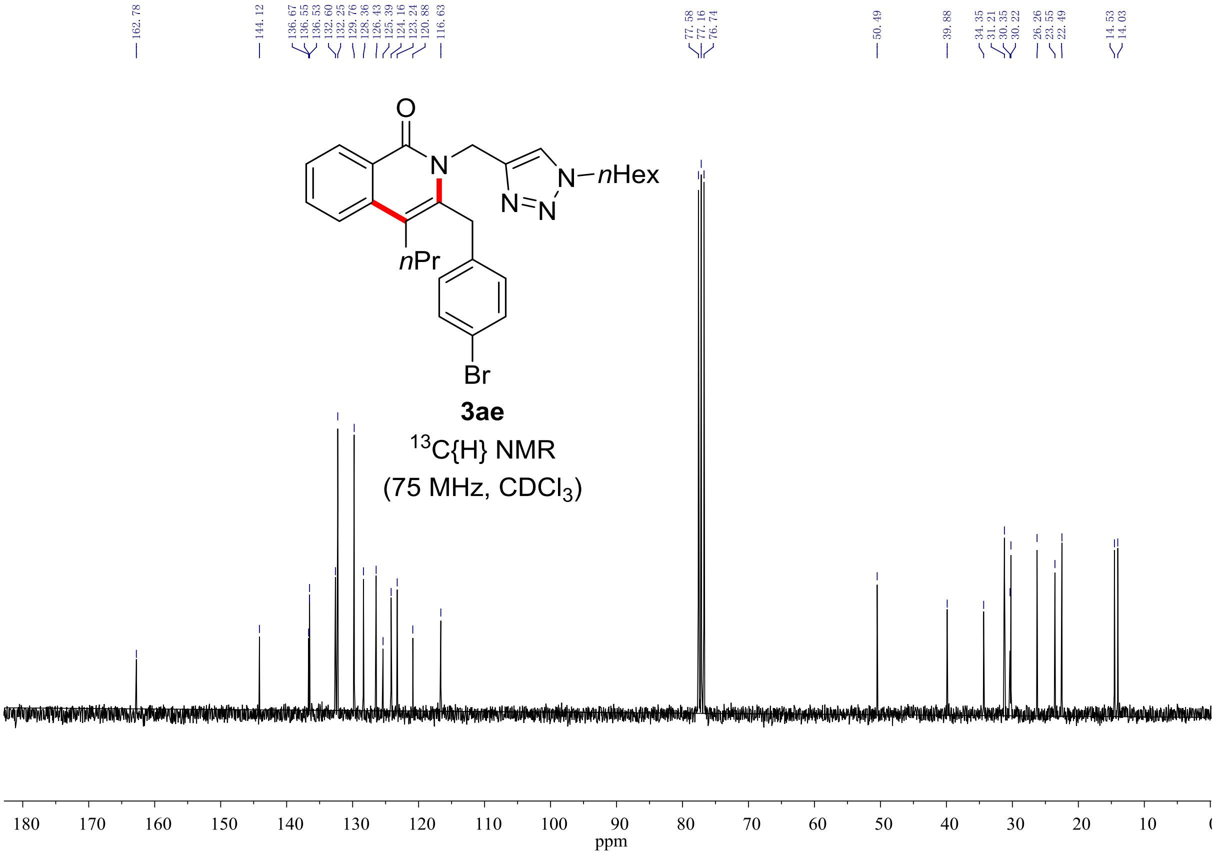
162.78
144.12
136.67
136.55
136.53
132.60
132.25
129.76
128.36
126.43
125.39
124.16
123.24
120.88
116.63
77.58
77.16
76.74
50.49
39.88
34.35
31.21
30.35
30.22
26.26
23.55
22.49
14.53
14.03
O
N
N
N
N
N−nHex
nPr
Br
3ae
13C{H} NMR
(75 MHz, CDCl3)
180 170 160 150 140 130 120 110 100 90 80 70 60 50 40 30 20 10
ppm

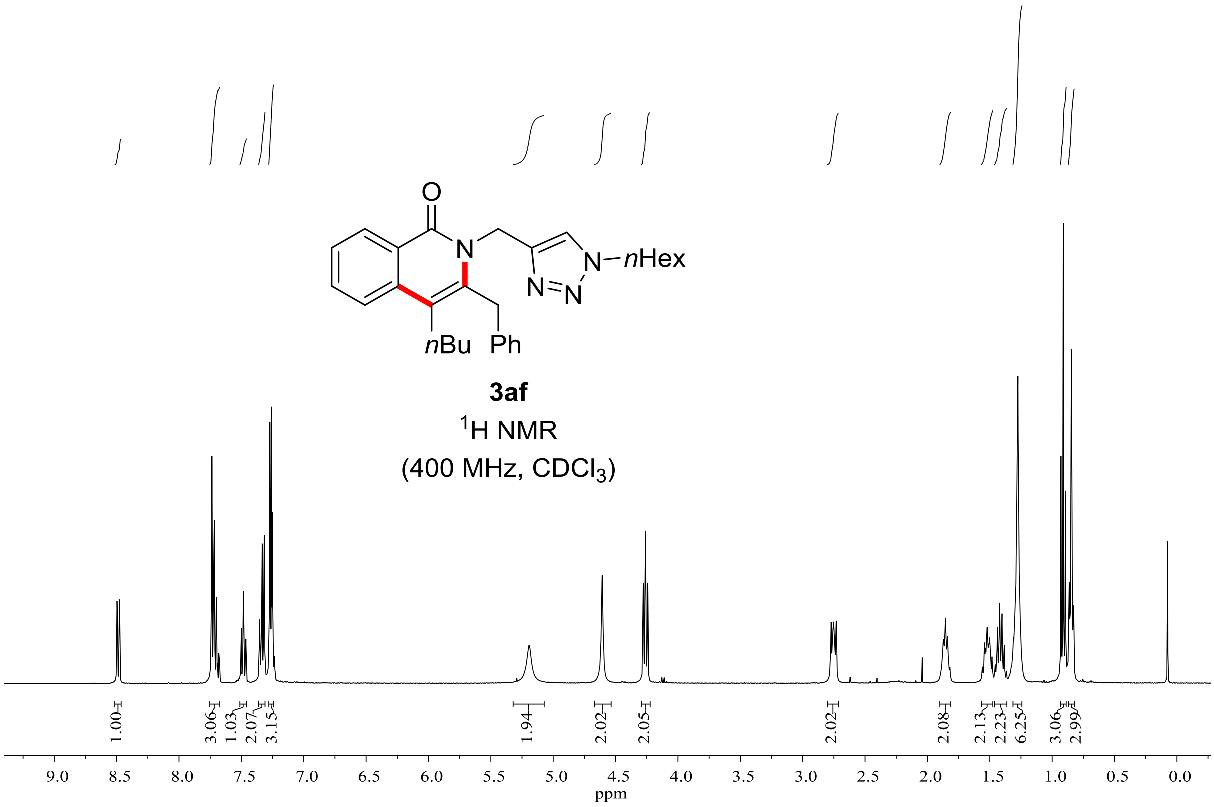

3af

^{1}H NMR

(400 MHz, $CDCl_3$)

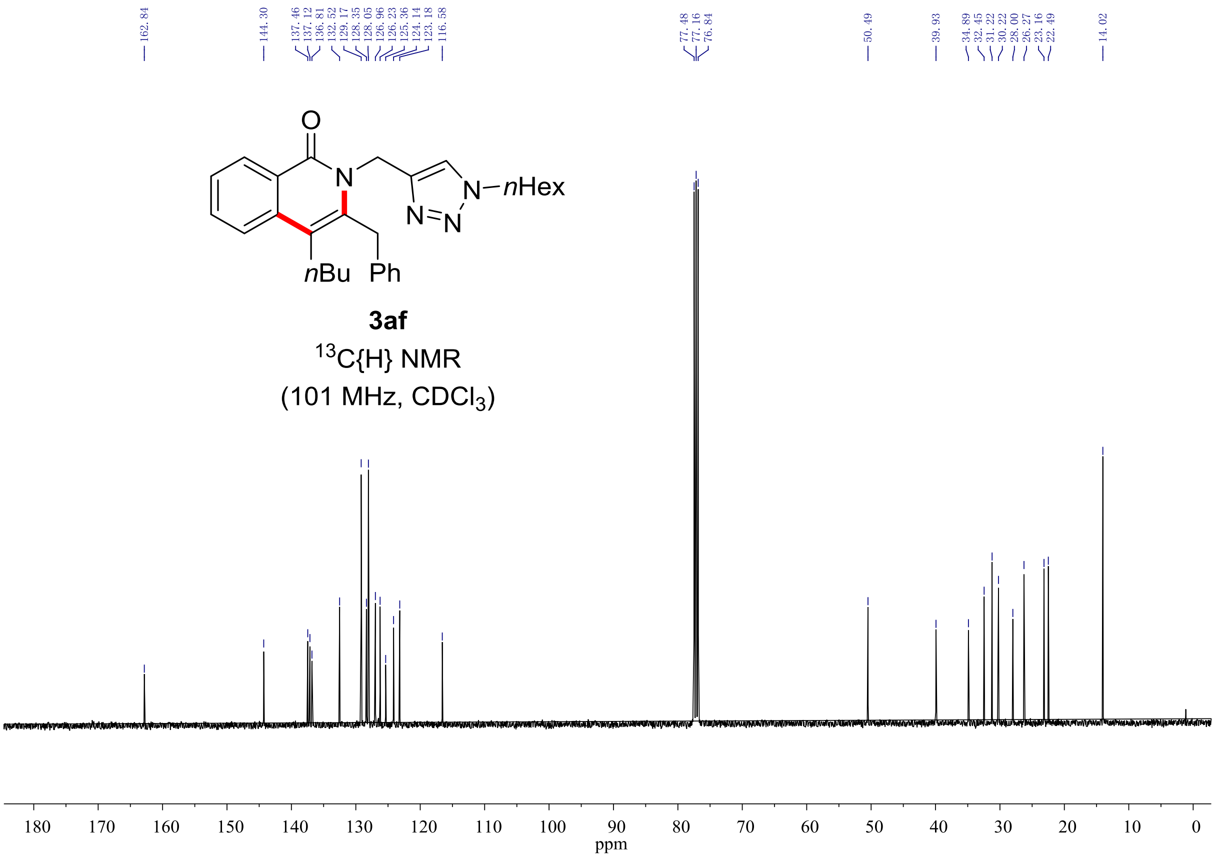

3af

^{13}C{H} NMR

(101 MHz, $CDCl_3$)

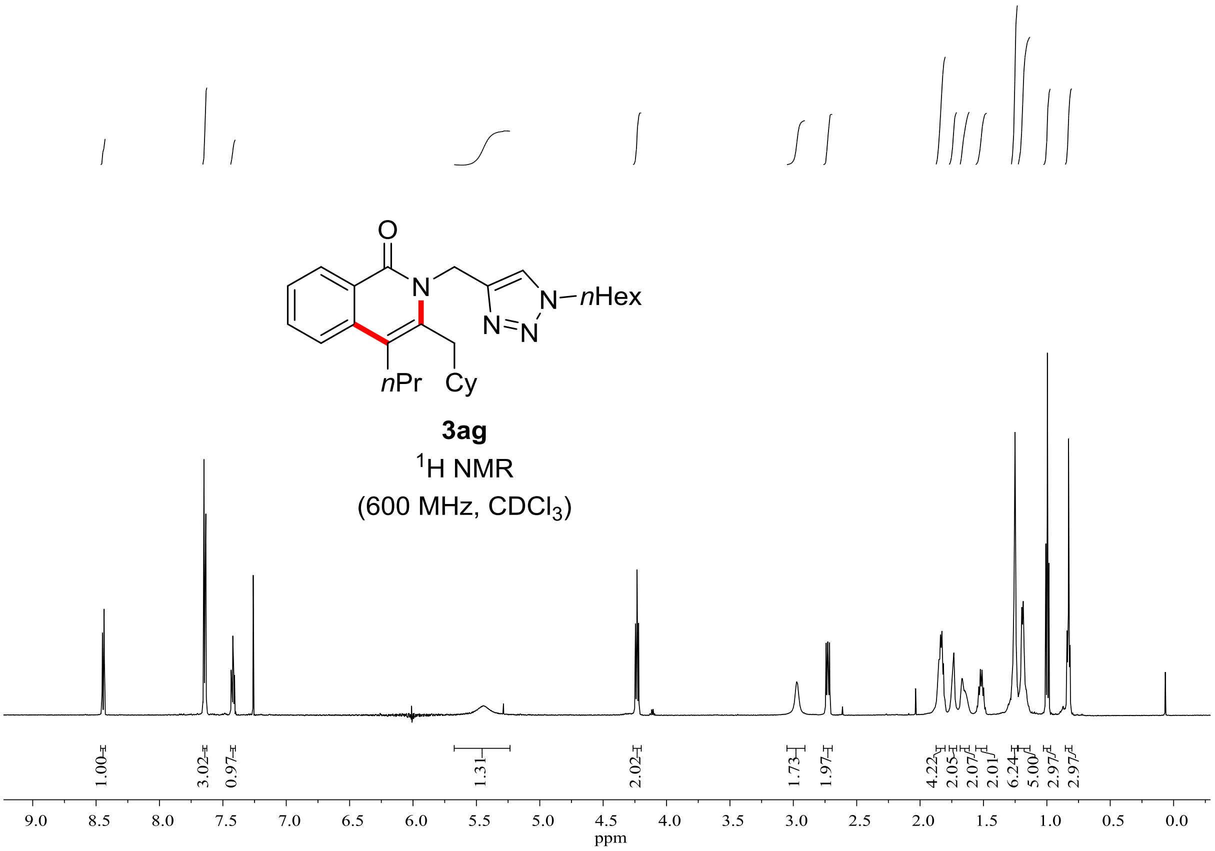

O
N
N—nHex
N=N
nPr
Cy
3ag
1H NMR
(600 MHz, CDCl3)
1.00
3.02
0.97
1.31
2.02
1.73
1.97
4.22
2.05
2.07
2.01
6.24
5.00
2.97
2.97
9.0 8.5 8.0 7.5 7.0 6.5 6.0 5.5 5.0 4.5 4.0 3.5 3.0 2.5 2.0 1.5 1.0 0.5 0.0
ppm

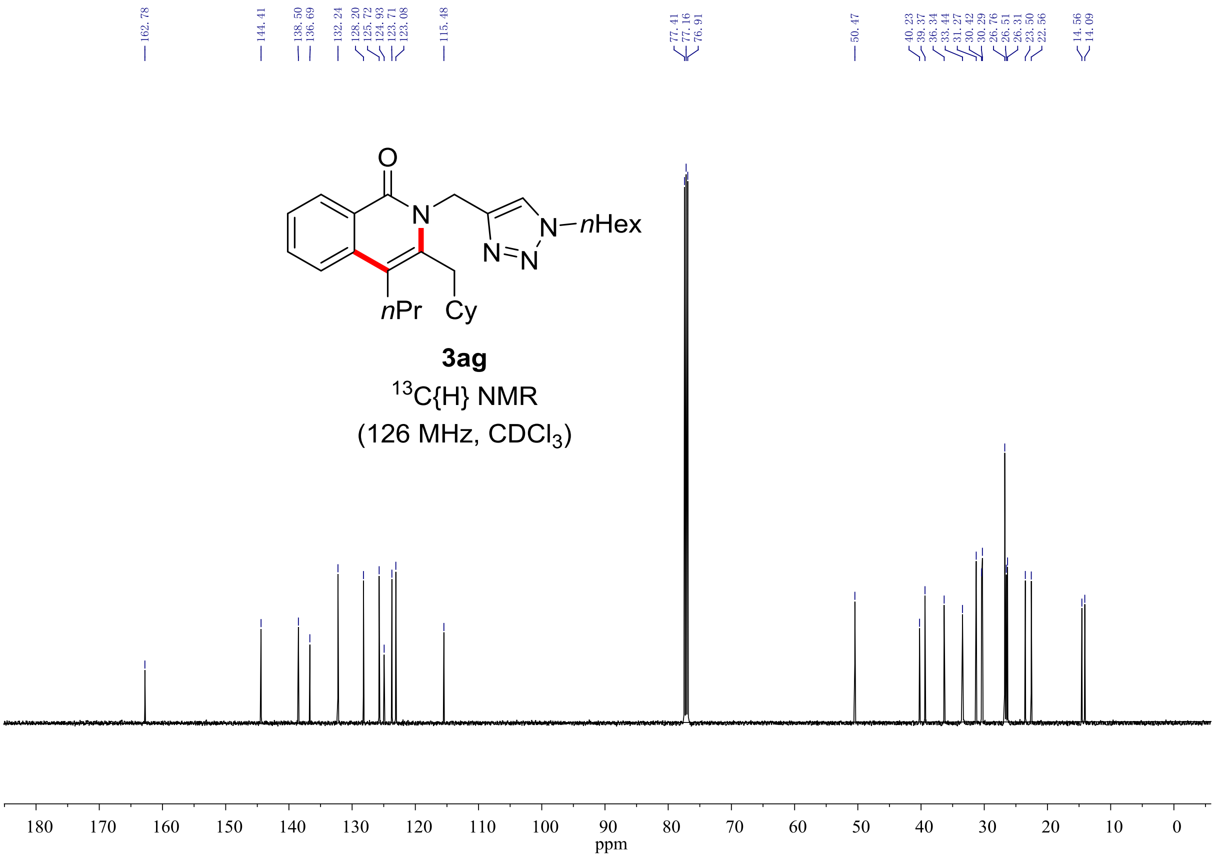

162.78
144.41
138.50
136.69
132.24
128.20
125.72
124.93
123.71
123.08
115.48
77.41
77.16
76.91
50.47
40.23
39.37
36.34
33.44
31.27
30.42
30.29
26.76
26.51
26.31
23.50
22.56
14.56
14.09
O
N
N—nHex
N=N
nPr
Cy
3ag
13C{H} NMR
(126 MHz, CDCl3)
180 170 160 150 140 130 120 110 100 90 80 70 60 50 40 30 20 10 0
ppm

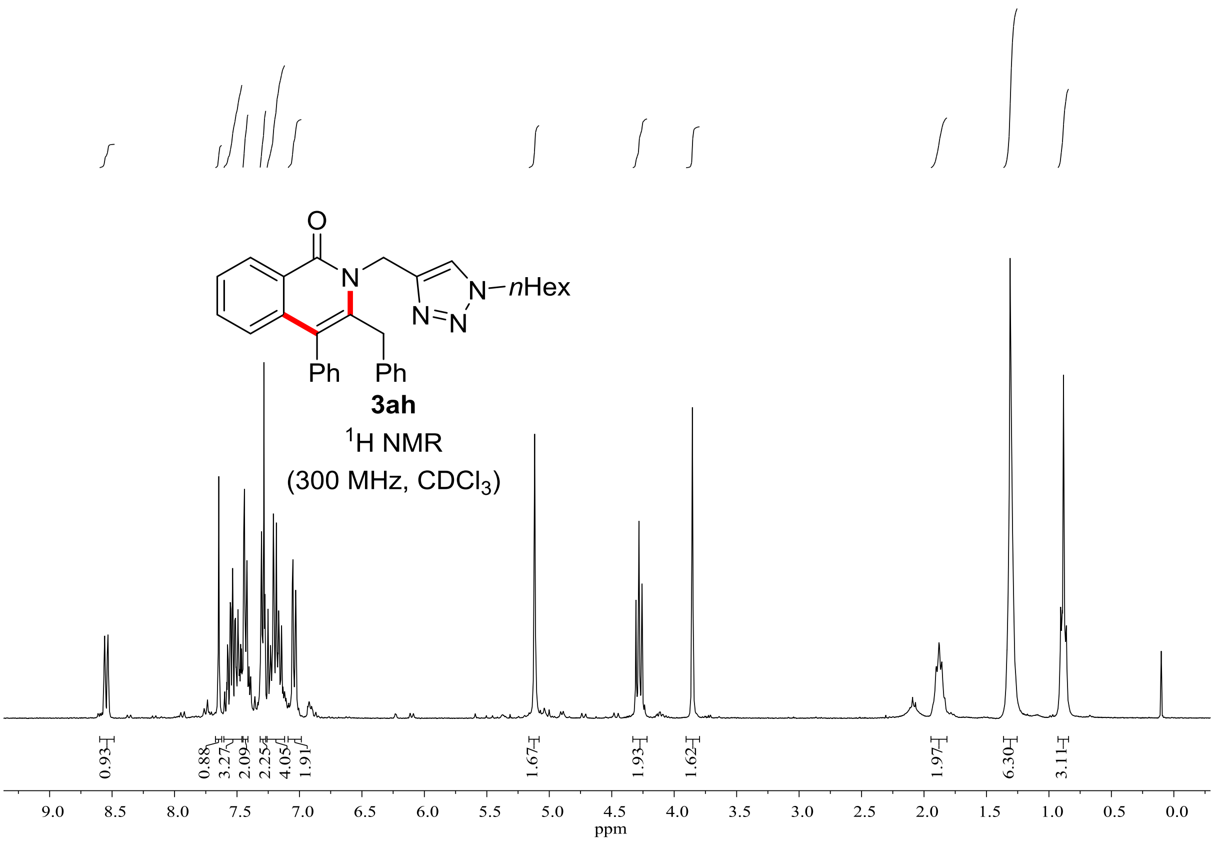

O
N
N−nHex
N=N
Ph
Ph
3ah
1H NMR
(300 MHz, CDCl3)
0.93
0.88
3.27
2.09
2.25
4.05
1.91
1.67
1.93
1.62
1.97
6.30
3.11
9.0 8.5 8.0 7.5 7.0 6.5 6.0 5.5 5.0 4.5 4.0 3.5 3.0 2.5 2.0 1.5 1.0 0.5 0.0
ppm

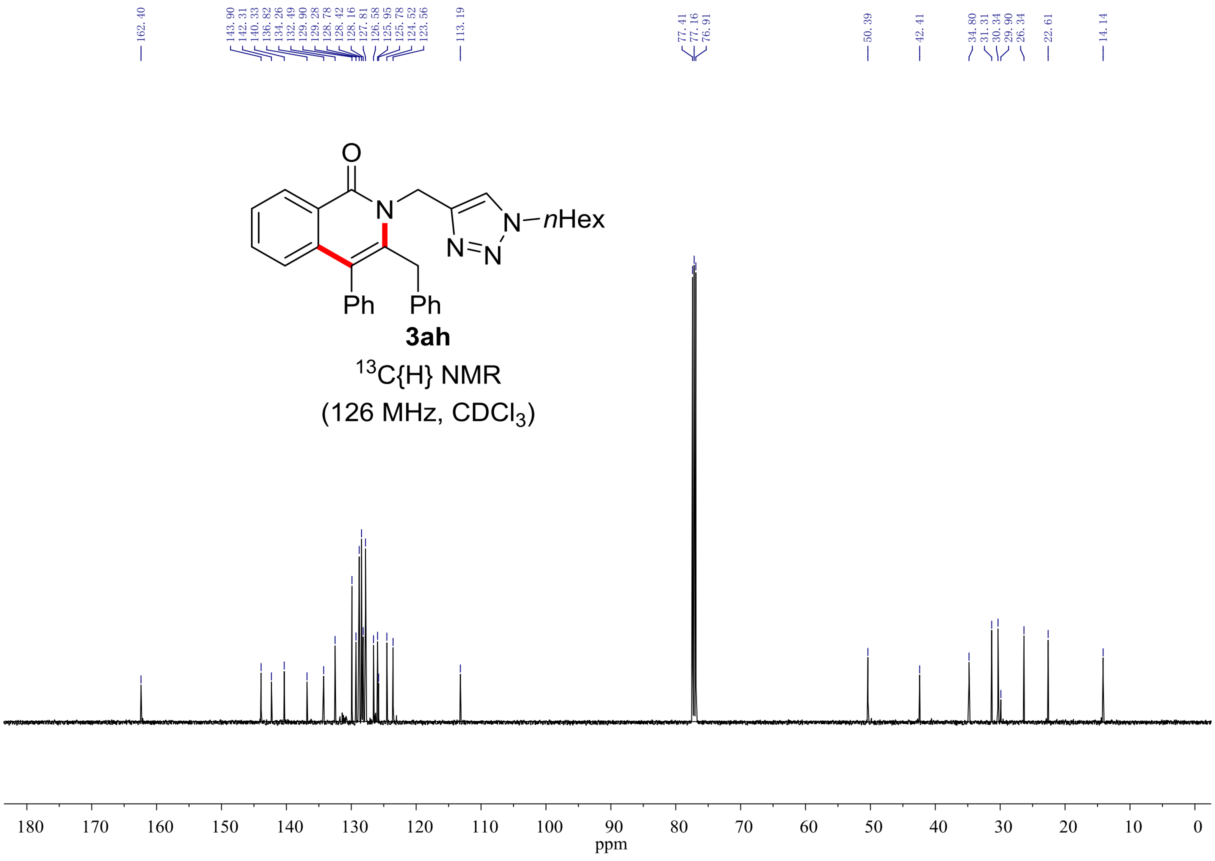

162.40
143.90
142.31
140.33
136.82
134.26
132.49
129.90
129.28
128.78
128.42
128.16
127.81
126.58
125.95
125.78
124.52
123.56
113.19
77.41
77.16
76.91
50.39
42.41
34.80
31.31
30.34
29.90
26.34
22.61
14.14
O
N
N−nHex
N=N
Ph
Ph
3ah
13C{H} NMR
(126 MHz, CDCl3)
180 170 160 150 140 130 120 110 100 90 80 70 60 50 40 30 20 10 0
ppm

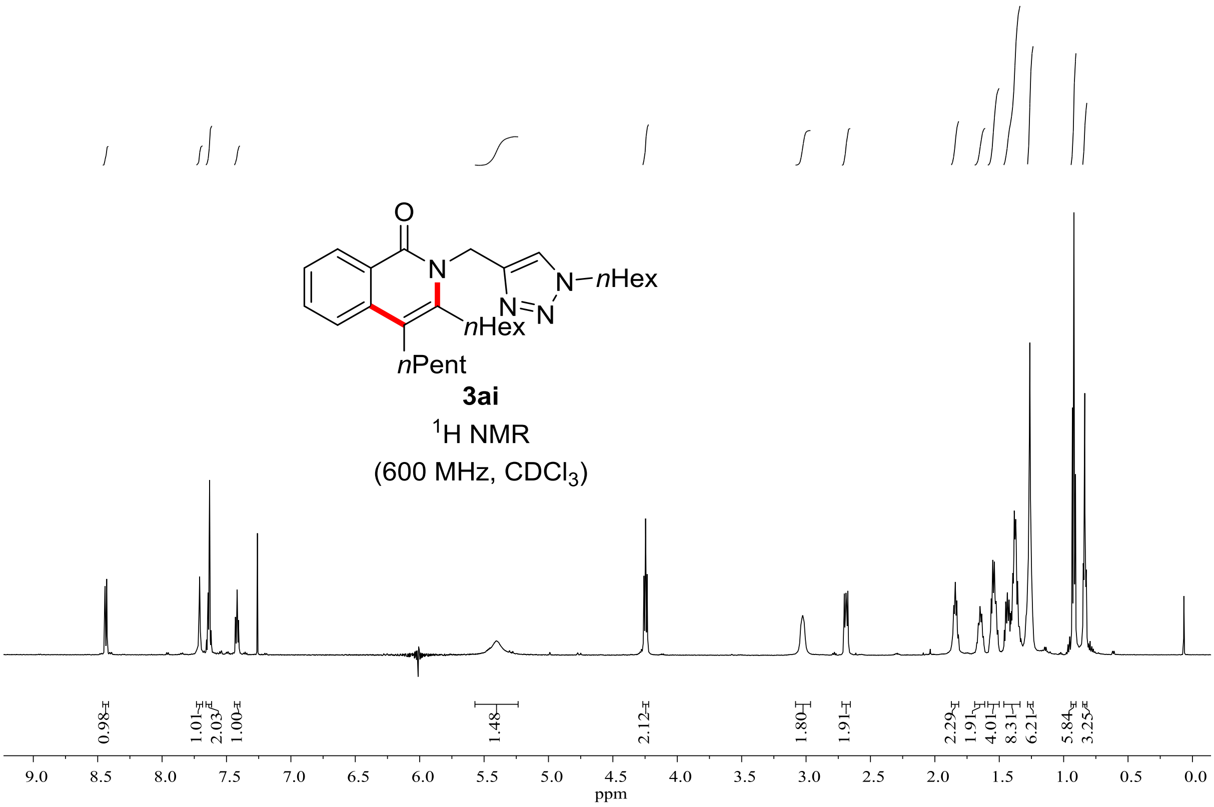
O
N
N
N
N
nHex
nHex
nPent
3ai
1H NMR
(600 MHz, CDCl3)
0.98
1.01
2.03
1.00
1.48
2.12
1.80
1.91
2.29
1.91
4.01
8.31
6.21
5.84
3.25
9.0
8.5
8.0
7.5
7.0
6.5
6.0
5.5
5.0
4.5
4.0
3.5
3.0
2.5
2.0
1.5
1.0
0.5
0.0
ppm

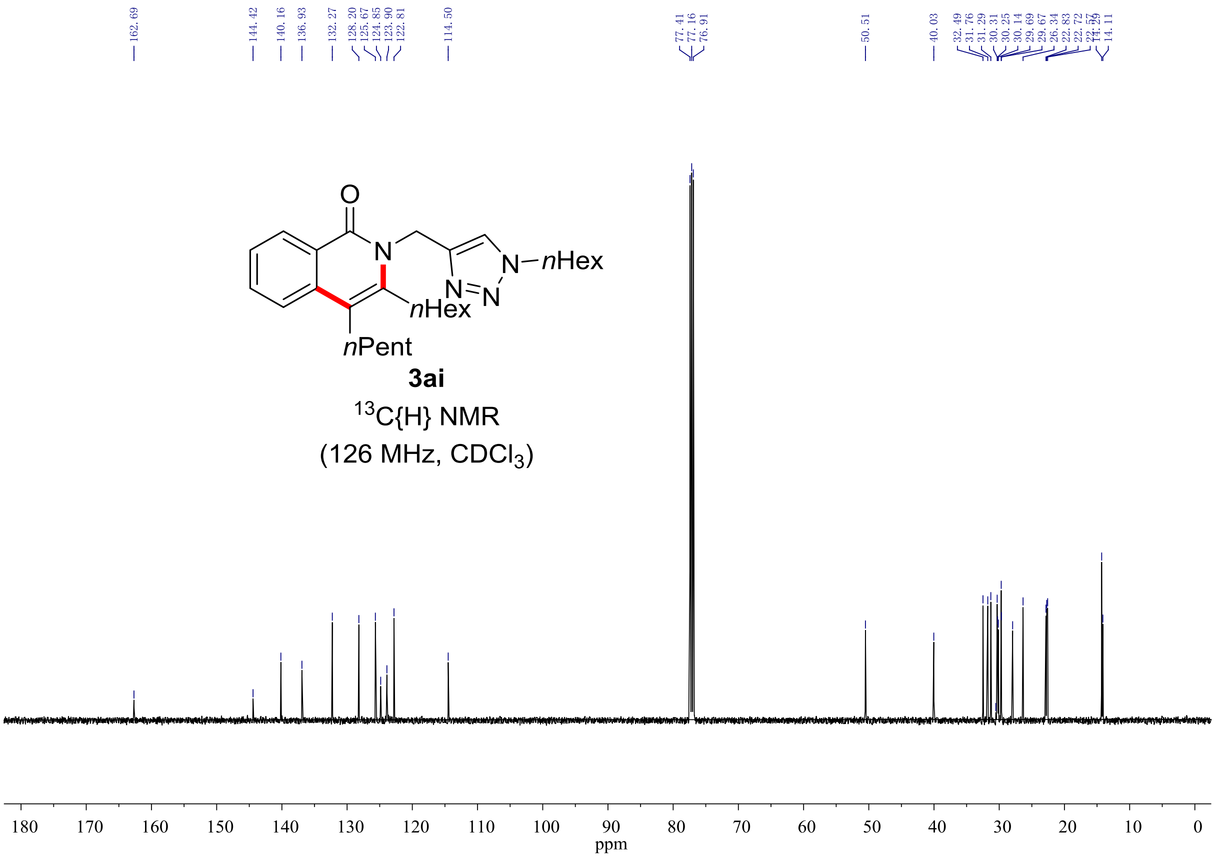
162.69
144.42
140.16
136.93
132.27
128.20
125.67
124.85
123.90
122.81
114.50
77.41
77.16
76.91
50.51
40.03
32.49
31.76
31.29
30.31
30.25
30.14
29.69
29.67
26.34
22.83
22.72
14.11
O
N
N
N
N
nHex
nHex
nPent
3ai
13C{H} NMR
(126 MHz, CDCl3)
180
170
160
150
140
130
120
110
100
90
80
70
60
50
40
30
20
10
0
ppm

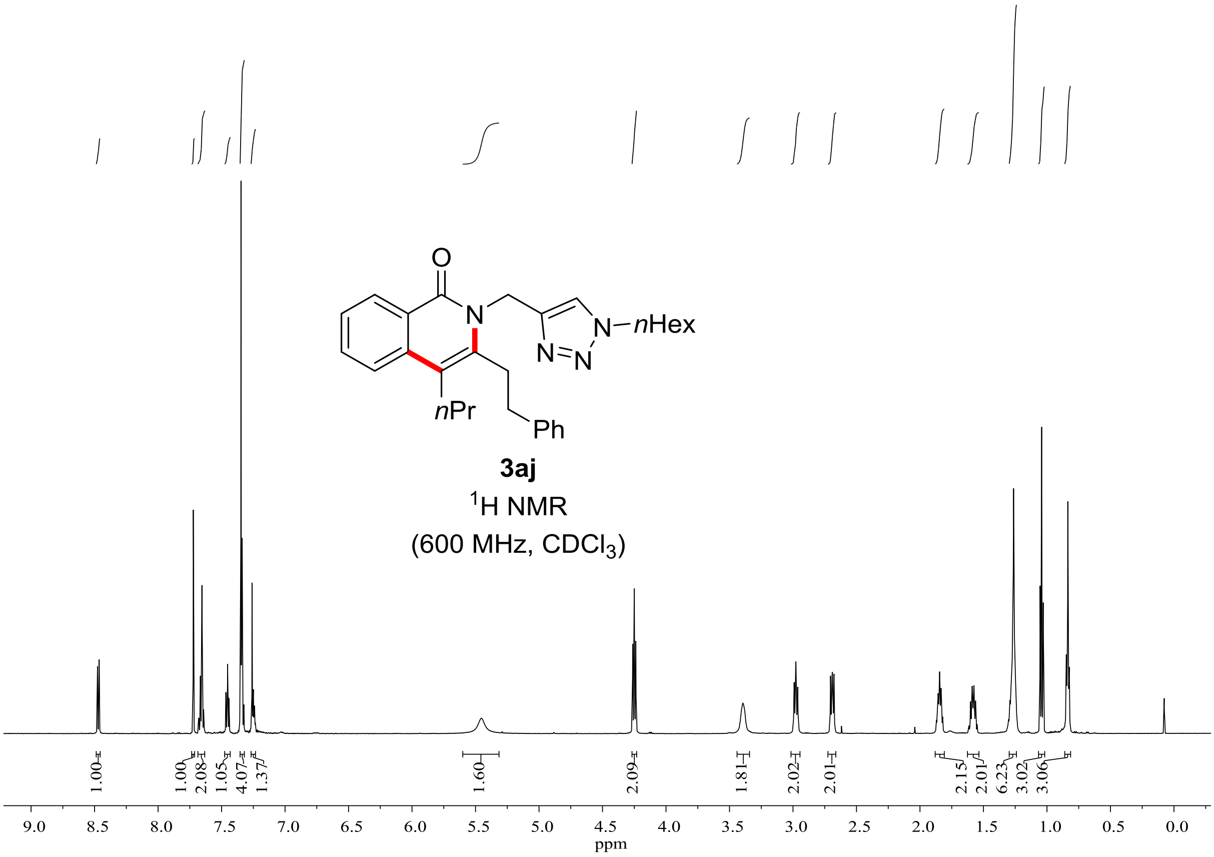

O
N
N–nHex
N=N
nPr
Ph
3aj
1H NMR
(600 MHz, CDCl3)
1.00
1.00
2.08
1.05
4.07
1.37
1.60
2.09
1.81
2.02
2.01
2.15
2.01
6.23
3.02
3.06
9.0
8.5
8.0
7.5
7.0
6.5
6.0
5.5
5.0
4.5
4.0
3.5
3.0
2.5
2.0
1.5
1.0
0.5
0.0
ppm

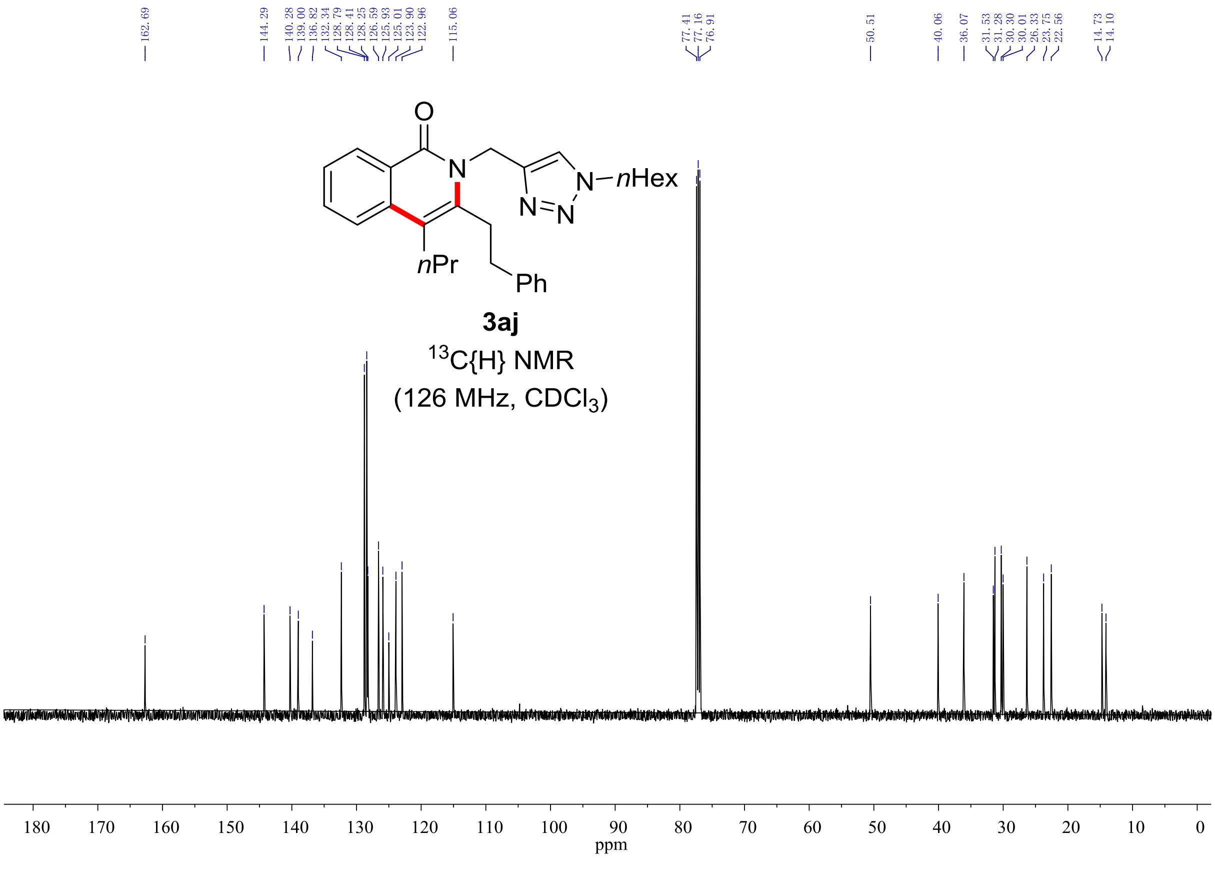

162.69
144.29
140.28
139.00
136.82
132.34
128.79
128.41
128.25
126.59
125.93
125.01
123.90
122.96
115.06
77.41
77.16
76.91
50.51
40.06
36.07
31.53
31.28
30.30
30.01
26.33
23.75
22.56
14.73
14.10
O
N
N–nHex
N=N
nPr
Ph
3aj
13C{H} NMR
(126 MHz, CDCl3)
180
170
160
150
140
130
120
110
100
90
80
70
60
50
40
30
20
10
0
ppm

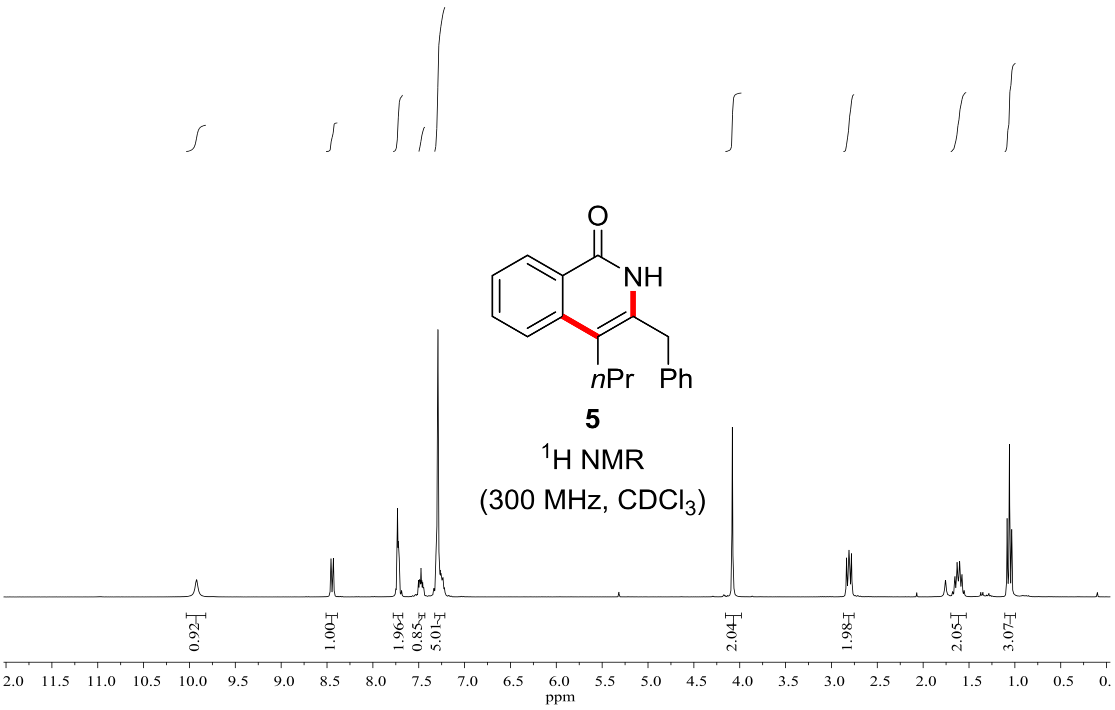
O
NH
nPr
Ph
5
¹H NMR
(300 MHz, CDCl₃)
0.92
1.00
1.96
0.85
5.01
2.04
1.98
2.05
3.07
ppm

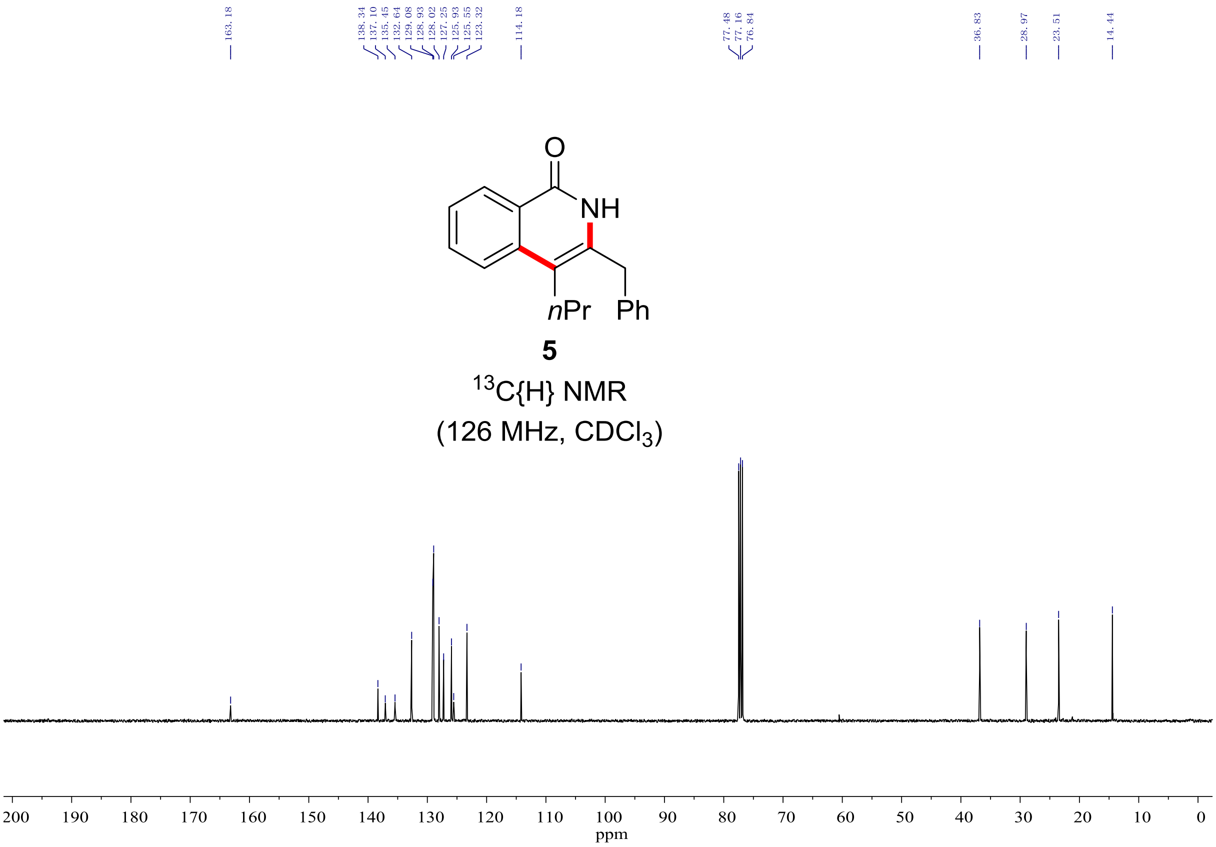
163.18
138.34
137.10
135.45
132.64
129.08
128.93
128.02
127.25
125.93
125.55
123.32
114.18
77.48
77.16
76.84
36.83
28.97
23.51
14.44
O
NH
nPr
Ph
5
¹³C{H} NMR
(126 MHz, CDCl₃)
ppm
